# Addition of dithi(ol)anylium tetrafluoroborates to α,β-unsaturated ketones

**DOI:** 10.3762/bjoc.14.37

**Published:** 2018-02-26

**Authors:** Yu-Chieh Huang, An Nguyen, Simone Gräßle, Sylvia Vanderheiden, Nicole Jung, Stefan Bräse

**Affiliations:** 1 Institute of Toxicology and Genetics, Karlsruhe Institute of Technology, Campus North, Hermann-von-Helmholtz-Platz 1, 76344 Eggenstein-Leopoldshafen, Germany; 2 Institute of Organic Chemistry, Karlsruhe Institute of Technology, Fritz-Haber-Weg 6, 76131 Karlsruhe, Germany

**Keywords:** addition to α,β-unsaturated carbonyls, dithiane chemistry, dithianylium tetrafluoroborate (TFB), ketene dithiane

## Abstract

In the presented study, dithi(ol)anylium tetrafluoroborates are added to α,β-unsaturated ketones in a Michael-type reaction yielding diverse substituted ketene diothi(ol)anes. The reactions proceed at room temperature in 1 or 13 h without the need of further additives. The presented procedure is in particular useful for dithi(ol)anylium tetrafluoroborates without electron-withdrawing groups in α-position. This is advantageous with respect to previous approaches, which were limited to the use of ketene dithioacetals substituted with electron-withdrawing groups. Aiming for the systematic investigation of possible steric and electronic influences on the outcome of the reaction, various combinations of electrophiles and nucleophiles were used and the results of the reactions were compared based on the type of the used dithioacetal. The scope of the presented procedure is shown with four additional transformations including the use of additional electrophiles and nucleophiles, the use of a chiral auxiliary and subsequent reduction of selected products. Additionally, we extended the reaction to the synthesis of diene dithiolanes by addition of an ynone to α-alkyl or aryl-substitued dithiolanylium TFBs.

## Introduction

1,3-Dithioacetals are a common motif in organic chemistry. They are part of dithiolane and dithiane protecting groups which are irreplaceable intermediates for the introduction of, e.g., fluorine via gem-difluorination [1–2]. They also allow the formation of valuable building blocks that can be used for diverse transformations in organic chemistry (e.g., Umpolung) [3]. Ketene 1,3-dithioacetals [4] are of particular interest as attractive nucleophiles for the addition to halonium ions [5–6], acyl chlorides [7] and other electrophiles [8–10] and are broadly used as precursors for [2 + 2]-cycloaddition [11–12], (aza)-Diels–Alder reaction [13–14], and [3 + 2]-cycloaddition reactions [15–16]. The products of these reactions are diversely substituted (ketene) dithiolanes or dithianes which allow a wide range of transformations like oxidation [17], fluorination [18] and cyclative reaction with [19–20] and without conservation [21–22] of the dithioacetal functionality. Besides many other well-known transformations of the α-position of ketene 1,3-dithioacetals, the reaction with azo-building blocks has been shown to yield α-azo ketene dithioacetals [23]. This finding was further studied by our group recently in order to show the potential of dithi(ol)anylium salts as an equivalent to ketene dithioacetals [24]. The disadvantages of the current synthetic use of ketene 1,3-dithioacetals are caused by the preparative effort for their isolation (via dithi(ol)anylium species) [25] and their substitution-dependent reactivity which often limits their use to ketenes with electron-withdrawing substituents in α-position [9,26]. Our recent approach to the synthesis of α-arylazo-carbonyl compounds showed that easily available dithi(ol)anylium tetrafluoroborates [27–28] (following shortened to TFBs) serve as stable precursors for ketene dithioacetals [29]. In accordance with previous work of others [30], they allow the addition of electrophiles in α-position independent of further presence of activating electron-withdrawing groups (EWGs). In the present study we will show the successful use of dithi(ol)anylium TFBs for the Michael-type addition to α,β-unsaturated ketones. A few examples for additions of activated (EWG-substituted) ketene 1,3-dithioacetals to α,β-unsaturated carbonyl compounds are literature known [31–32] but concepts for a versatile (metal-free) application of this reaction to starting materials lacking the EWG in α-position are still missing.

## Results and Discussion

### Addition to α,β-unsaturated ketones

**Scheme 1:**
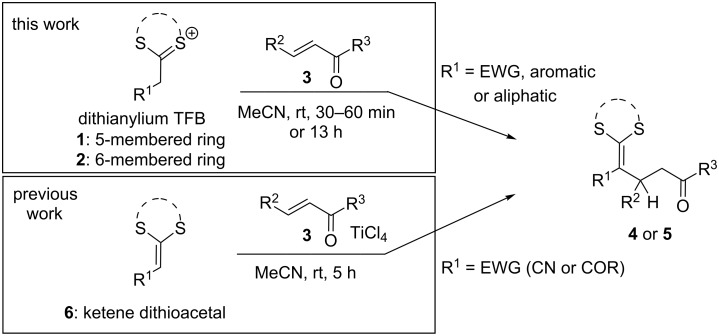
Previously reported procedure for the addition of ketene dithioacetals to α,β-unsaturated ketones [33] in comparison to our dithi(ol)anylium TFB-based approach.

Since we could show the benefits of a use of dithi(ol)anylium TFBs instead of ketene 1,3-dithioacetals for the addition of diazo components [31], we were interested in a more general use of dithi(ol)anylium TFBs for the addition to electrophiles in α-position to the thioacetal-masked carbonyl group. Five- and six-membered thioacetal species (compounds of type **1** and **2**, Scheme 1) have been selected as nucleophiles among other non-cyclic thioacetal TFBs because of their fast and easy availability from reactions of propanedithiol or ethanedithiol with carbonyl chlorides. Many dithi(ol)anylium TFBs form stable solids that can be crystallized and stored for several days or even weeks, others form oils that can be isolated from their reaction mixtures via separation of the reaction supernatant without further purification. Dithi(ol)anylium TFBs combine a high nucleophilicity of the α-position (as a result of the strong electron donating nature of the dithio group) with enhanced reactivity of the TFBs in comparison to the corresponding ketenes. Therefore, dithi(ol)anyl TFBs have been identified as interesting starting material for a feasible synthesis of organic building blocks. Herein we investigate the scope and limitations of dithi(ol)anyl TFBs for a fast generation of masked 1,5-dicarbonyl compounds similar to Michael additions using ketene dithioacetals without the use of stoichiometric amounts of metals or additional catalysts which have been proposed in former approaches to the synthesis of the target compounds **4** or **5** (see Scheme 1).

**Scheme 2:**
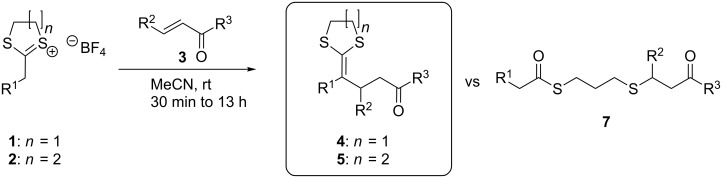
Addition of dithi(ol)anylium TFBs to α,β-unsaturated non-cyclic ketones.

The systematic analysis of the effect of the acetal ring size and substituents on the electrophilic and the nucleophilic component was used to reveal the influence of steric but also electronic changes on the outcome of the reaction. We synthesized eight different dithi(ol)anylium TFBs **1a–d** and **2a–d** from commercial carbonyl chlorides, isolated them via crystallization or separation and extraction and added them to a set of eight α,β-unsaturated non-cyclic ketones (Scheme 2 and Table 1). It was shown that dithi(ol)anylium TFBs, with different substitution patterns in α-position (R^1^), could be converted successfully to yield either ketene dithiolanes **4** or ketene dithianes **5**. The reactions were successful without the need of additional reagents or catalysts. We were able to show that the ring size of the dithioacetal group in **1** or **2**, the nature of the nucleophile (**1a–d, 2a–d**), and the nature of the electrophile (**3a–h**) have influence not only on the reaction yield but also on the success of the reaction and the obtained products. Almost all combinations of **1a** or **2a** and **3** giving the products **4** or **5** bearing a phenyl group in position R^1^ are possible and result in the isolation of **4a**–**5h** in up to very good yields (entries 1–8, Table 1). The only exception is compound **5e**, which could not be obtained due to the influence of the sterically bulky iPr group of the electrophile **3e**. The conversion of dithiane **2a** with electrophile **3e** resulted instead in a ring opening of the dithiane via hydrolysis and gave compound **7e** in 62% yield (for further details, please see Supporting Information File 1). The use of dithioacetals with other substituents in position R^1^ (R^1^ = Me, Et) again resulted in some cases in the formation of compounds of type **7**. This was observed in reactions using the ketene dithiane **2** as a starting material, while the use of ketene dithiolanes **1** did not cause ring opening during the reaction. The occurrence of the not desired hydrolysed compound of type **7** was generally observed if a sterically demanding combination of nucleophile (depends on ring size and R^1^) and electrophile (depends on R^2^) was chosen (e.g., entries 10, 13 and 14, Table 1). For compounds with low steric hindrance (**5i**, **5r** and all compounds **4**), the conversion to the desired products **4a**–**5r** was successful and no byproduct of type **7** was observed. For all reactions that resulted in the successful formation of **4** or **5**, the nature of the substituent R^2^ has a considerable effect on the reaction time. While reactions using electrophiles **3a–d,** and **3f** (R^2^ = H, Me, Bu) were finished with complete conversion of the starting material after a few minutes at rt [33], reactions with **3e** and **3g** (R^2^ = iPr, Ph) required a reaction time of 13 h. As addition reactions of ketene dithioacetals bearing EWGs in α-position have been described earlier, we used the electrophile **3d** for a comparison of those literature-known procedures with our experiments using non-EWG bearing reagents. Interestingly, the dithiolanylium TFB **2d** with α-EWG gave a lower yield compared to other examples of our approach (**5r** gave 46% yield, entry 18, Table 1). The outcome of this reaction is also only poor in comparison to examples known from the literature (where actually only CN groups in α-position have been investigated so far) [33].

**Table 1:** Addition of dithi(ol)anylium TFBs **1a**,**2a**–**1d**,**2d** to ketones **3a**–**h**.

Entry	Dithi(ol)ane	**3**	Product	R^1^	R^2^	R^3^	Time [h]	Yield [%]^a^	
								**4**	**5**

1	**1a** or **2a**	**3a**	**4a** or **5a**	Ph	H	Me	1	64	32
2	**1a** or **2a**	**3b**	**4b** or **5b**	Ph	H	Et	1	62	31
3	**1a** or **2a**	**3c**	**4c** or **5c**	Ph	Me	Me	1	94	36
4	**1a** or **2a**	**3d**	**4d** or **5d**	Ph	Me	Et	1	97	37
5	**1a** or **2a**	**3e**	**4e** or **5e**	Ph	iPr	Me	13	85	–^b^
6	**1a** or **2a**	**3f**	**4f** or **5f**	Ph	Bu	Me	1	90	64
7	**1a** or **2a**	**3g**	**4g** or **5g**	Ph	Ph	Me	13	56	43
8	**1a** or **2a**	**3h**	**4h** or **5h**	Ph	Ph	Ph	13	79	87
9	**1b** or **2b**	**3d**	**4i** or **5i**	Me	Me	Et	1	71	60
10	**1b** or **2b**	**3e**	**4j** or **5j**	Me	iPr	Me	13	52	–^b^
11	**1b** or **2b**	**3f**	**4k** or **5k**	Me	Bu	Me	1	66	–
12	**1b** or **2b**	**3g**	**4l** or **5l**	Me	Ph	Me	13	73	–
13	**1c** or **2c**	**3b**	**4m** or **5m**	Et	H	Et	1	55	–^b^
14	**1c** or **2c**	**3d**	**4n** or **5n**	Et	Me	Et	1	77	–^b^
15	**1c** or **2c**	**3e**	**4o** or **5o**	Et	iPr	Me	13	44	–
16	**1c** or **2c**	**3f**	**4p** or **5p**	Et	Bu	Me	1	79	–
17	**1c** or **2c**	**3h**	**4q** or **5q**	Et	Ph	Ph	13	65	–
18	**1d** or **2d**	**3d**	**4r** or **5r**	CO_2_Me	Me	Et	1	–	46

^a^No entry (–) means the combination was not tested if not otherwise stated with ^b^; ^b^compound **7** was formed instead of **5** (for further information and yields of **7** see Supporting Information File 1).

**Scheme 3:**
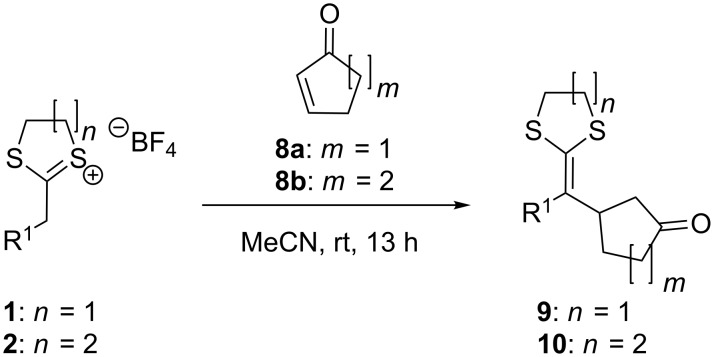
Addition of dithi(ol)anylium TFBs to α,β-unsaturated cyclic ketones.

The approach was further used for the addition of dithi(ol)anylium TFBs **1a**–**d** and **2a**,**b**,**d** to cyclic ketones, namely cyclopent-2-en-1-one (**8a**) and cyclohex-2-en-1-one (**8b**, Scheme 3). In those reactions, the use of dithiolanylium TFBs (**1**) was favoured in comparison to the use of dithianylium TFBs (**2**), which gave, especially in combination with cyclohex-2-en-1-one (**8b**), poorer yields. For the formation of compound **10b**, a conversion of **2a** with **8b** gave only 52% yield under the standard conditions at rt whereas an increase of the temperature to 60 °C resulted in the isolation of the desired product with 81% yield. In opposite to this finding, the addition of dithiolanylium TFBs **1** to cyclic unsaturated ketones was, especially concerning the nucleophile **1b**, very fast and the formation of byproducts could only be prevented by performing the reaction at lower temperatures (−40 °C). The reactions summarized in Table 2 show again (in comparison with Table 1) a different behaviour of EWG substituted and unsubstituted dithi(ol)anylium TFBs. The reactions using EWG substituted dithiolanylium TFB **1d** gave no yield of the desired product and the reaction of dithianylium TFB **2d** resulted in the successful formation of the desired target compounds **10g** and **10h**. This result is against the trend observed for entries 1–6 (Table 2), where the conversion of compounds **1** worked better than those with **2**.

**Table 2:** Addition of dithi(ol)anylium TFBs **1a–d** or **2a**,**b**,**d** to ketones **8a** or **8b**.

Entry	Dithi(ol)ane	**8**	Product	R^1^	*m*	Yield [%]^a^	
						**9**	**10**

1	**1a** or **2a**	**8a**	**9a** or **10a**	Ph	1	93	92
2	**1a** or **2a**	**8b**	**9b** or **10b**	Ph	2	94	52 (81^b^)
3	**1b** or **2b**	**8a**	**9c** or **10c**	Me	1	34 (59^c^)	26
4	**1b** or **2b**	**8b**	**9d** or **10d**	Me	2	0 (42^c^)	–
5	**1c** or **2c**	**8a**	**9e** or **10e**	Et	1	79	–
6	**1c** or **2c**	**8b**	**9f** or **10f**	Et	2	40	–
7	**1d** or **2d**	**8a**	**9g** or **10g**	CO_2_Me	1	0	68
8	**1d** or **2d**	**8b**	**9h** or **10h**	CO_2_Me	2	0	42

^a^No entry (–) means the combination was not tested; ^b^reaction at 60 °C for 15 h; ^c^reaction at −40 °C for 4 h.

**Scheme 4:**
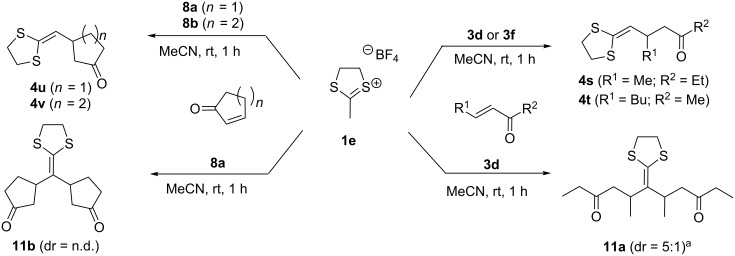
Single versus double addition of ketones to dithiolanylium TFB **1e**. ^a^dr was calculated from the ^1^H NMR results.

The presented procedure can also be used for the modification of α-unsubstituted dithiolanylium TFBs, yielding either single (products **4s**–**v**, Scheme 4) or double substitution (products **11a** and **11b**, Scheme 4) of the ketene dithiolane in α-position. The reaction can be directed towards single or double substitution via the amount of ketone used in the reaction (Table 3).

**Table 3:** Reaction of dithiolanylium TFB **1e** with ketones **3d**,**f** or **8a**,**b**.

Entry	**3** or **8**	Ratio [**1e**:**3**/**8**]	Product	R^1^	R^2^	Time [h]	Yield [%] **4** or **11**

1	**3d**	**1:1**	**4s**	Me	Et	1	55
2	**3f**	**1:1**	**4t**	Bu	Me	1	57
3	**8a**	**1:1**	**4u**	–	–	1	50
4	**8b**	**1:1**	**4v**	–	–	1	23
5	**3d**	**1:3**	**11a**	Me	Et	1	13
6	**8a**	**1:3**	**11b**	–	–	1	67

### Mechanistic aspects of the reaction

The mechanism of the reaction was investigated by changing the reaction conditions during the conversion of dithiolanylium TFB into compound **4c** exemplarily. As described in a previous work of our group on the addition of dithi(ol)anylium TFBs to electrophiles [29] the reaction of dithi(ol)anylium TFBs occurs via their decomposition into ketene dithi(ol)anes and HBF_4_. The improved conversion of the resulting ketene dithi(ol)anes in comparison to reactions where only ketene dithi(ol)anes are used is assumed to be a result of an activating effect of the released acid HBF_4_ during the reaction. This assumption was proven via experiments where different amounts of trimethylamine were added to capture the nascent HBF_4_. It was shown that if a certain amount of HBF_4_ is removed from the reaction by addition of NEt_3_, the reaction happens slower but still can be driven to completion within 3 hours if 10% of HBF_4_ remains in the reaction mixture (see table for comparative experiments in the Supporting Information File 1). This result indicates the catalytic activity of HBF_4_ in the reaction. The same product **4c** can be obtained if an isolated ketene dithi(ol)ane is subjected to HBF_4_.

### The scope of the dithi(ol)anylium TFB addition to α,β-unsaturated carbonyl compounds

**Scheme 5:**
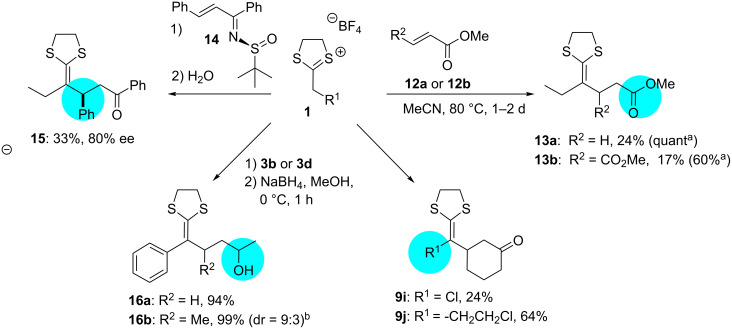
The scope of the presented protocol demonstrated by examples including the use of additional electrophiles (**13a**, **13b**) or nucleophiles (**9i**, **9j**), the use of a chiral auxiliary (**15**) or reductions of the obtained products **4** (**16a**, **16b**). ^a^CH_2_Cl_2_, 1 equiv Me_2_AlCl, rt; **13a**: 1 h, **13b**: 18 h. ^b^dr was calculated from the ^1^H NMR results.

In order to determine the scope of the herein presented reaction, we used compound **1c** for further conversions indicating the possible challenges and aims of forthcoming projects (Scheme 5). With two selected examples, we showed that it is possible to extend the presented procedure to the reaction of dithiolanylium TFB with other Michael acceptors as, e.g., α,β-unsaturated esters **12a** or **12b** (yielding **13a** and **13b**, Scheme 5). The reaction proceeded under similar conditions as shown in Table 1 (the reaction temperature was adjusted to 80 °C because of the low conversion at room temperature), but the initially obtained yield of the reaction was very low even though the reaction time was increased (**13a** = 24% (48 h), **13b** = 17% (24 h)). The addition of a Lewis acid (Me_2_AlCl) improved the results for the selected examples and the products **13a** and **13b** could be isolated in 60% and quantitative yield, respectively, via the adapted protocol. Further reactions have been conducted with other dithiolanylium TBFs to show that derivatives with functionalization in the α-position are tolerated. The successful use of an α-chlorinated dithiolanylium TFB (isolation of **9i**, Scheme 5) as well as an α-alkyl chloride substitution (isolation of **9j**, Scheme 5) demonstrate that the introduction of additional functionality is possible and that starting materials as well as products are stable enough for their use and purification. As the versatility of the presented synthetic targets as precursors in novel reactions depends on the stability of the obtained compounds and methods for their selective transformation, we tested exemplarily the reduction of the carbonyl group with sodium borohydride. We could show the conversion into the corresponding alcohol in very good yields for two examples (**16a** = 94%; **16b** = 99%, Scheme 5), demonstrating the possible reductive transformation of the ketone in the presence of the ketene dithioacetal function. While our initial procedure did not include investigations towards a stereoselective addition of the dithiolanylium TFB to α,β-unsaturated ketones, the derivative **4q** (see Table 1) has been selected to prove at least the general applicability of the presented procedure in the context of stereoselective conversion. In accordance with a literature-known protocol for the formation of sulfonylimines from α,β-unsaturated ketones [34], we attached the chiral Ellman auxiliary to the electrophile prior to its conversion with the dithiolanylium TFB **1c**. The resulting chiral ketene dithiolane **15** could be obtained in 33% yield and 80% ee.

**Scheme 6:**
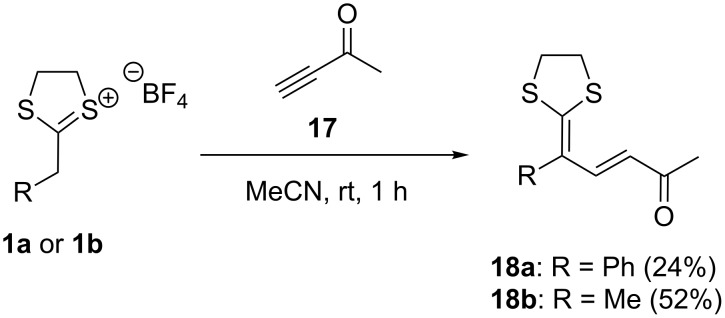
Synthesis of diene dithioacetals **18a** and **18b** by addition of ynone **17** to α-alkyl or aryl-substitued dithiolanylium TFBs.

In two additional experiments, we investigated the potential of the herein shown procedure for the synthesis of diene dithioacetals. A similar reaction using α-carbonyl substituted ketene dithioacetals for an addition to alkynes under iron catalysis was shown by Liu et al. before and was used for the synthesis of δ-lactams and lactones by 6-*endo* annulation [35]. Our approach reveals that comparable reactions of α-alkyl or aryl-substitued dithiolanylium TFBs can be used for the reaction with ynones giving diene dithioacetals as compounds **18a** and **18b** in a related manner (Scheme 6).

## Conclusion

The presented study shows that some of the current challenges concerning the addition of ketene dithioacetals or their synthetic equivalents to electrophiles can be overcome by the use of dithi(ol)anylium TFBs. Dithi(ol)anylium TFBs were used in combination with α,β-unsaturated ketones as Michael-type acceptors yielding ketene diothiolanes and ketene dithianes with various substitution patterns. The reactions worked without the need of further stoichiometric or catalytic amounts of additives and the concept was demonstrated to be successful for diverse dithi(ol)anylium derivatives. The presented procedure is in particular useful for dithi(ol)anylium TFBs without EWGs in α-position being advantageous in comparison to previous approaches which were limited to the use of ketene dithioacetals substituted with electron-withdrawing groups. The scope of the presented procedure was shown with four additional transformations including the use of additional electrophiles and nucleophiles, the use of a chiral auxiliary and subsequent reduction of selected products. Furthermore, we extended the reaction to the addition of ynones to α-alkyl or aryl-substitued dithiolanylium TFBs showing their successful transformation to diene dithiolanes in two examples. One limitation of the procedure concerning the use of possible Michael acceptors was identified so far as the conversion of dithi(ol)anylium TFBs with 3,3-disubstituted alk-2-en-1-ones did not occur.

## Supporting Information

File 1 Experimental part.

File 2 Collection of spectra and summary of IR data of starting materials.
