# Supplementary material for: Addition of dithi(ol)anylium tetrafluoroborates to α,β-unsaturated ketones
Source: Beilstein J Org Chem. 2018 Feb 26;14:515–22. doi: 10.3762/bjoc.14.37 (PMC5852463; doi:10.3762/bjoc.14.37)
Supplement: File 1 — Experimental part. [file Beilstein_J_Org_Chem-14-515-s001.pdf]

Beilstein J. Org. Chem. **2018,** *14,* 515–522. doi:10.3762/bjoc.14.37

Supporting Information File 1

for

Addition of dithi(ol)anylium tetrafluoroborates to α,β-unsaturated ketones

Yu-Chieh Huang[1], An Nguyen[1], Simone Gräßle[1], Sylvia Vanderheiden[2], Nicole Jung*[1,2] and Stefan Bräse*[1,2]

Address: [1]Institute of Toxicology and Genetics, Karlsruhe Institute of Technology, Campus North, Hermann-von-Helmholtz-Platz 1, 76344 Eggenstein-Leopoldshafen, Germany and [2]Institute of Organic Chemistry, Karlsruhe Institute of Technology, Fritz-Haber-Weg 6, 76131 Karlsruhe, Germany

Email: Nicole Jung - nicole.jung@kit.edu; Stefan Bräse - braese@kit.edu

* Corresponding author

Experimental part

Readme: This Supplemental Information was created automatically by an export function of the Software Chemotion-ELN (ELN = electronic lab notebook). All listed information was retrieved via the electronic lab notebook (please see point 3).

We introduced particular changes to the common way of reporting in order to shorten the textual part and to add additional information that is necessary to explicitly identify the described procedures and molecules.

(1) We introduce a novel manner to label molecules. The labeling in the form of {A|**1a**} refers, indicated by the "A" to a more general sorting of the molecule according to its function in the reaction (assigned to the General Procedure). The alphabetic sorting of starting materials, reagents and products is given for all general procedures (e.g., from A to D), allowing the labeling of the molecules in the distinct reaction according to the generic label given in the general procedure.

The common alpha numeric label which refers to the unique number of the molecules as given in the article is added in addition as usual in bold after the slash "|**1a**".

(2) We added general identifiers and properties which can be calculated with a given molecular structure at the beginning of each analysis of the molecules. This procedure should facilitate the proof reading and the identification of the molecules (also automatically).

(3) Changes that were made after the automatic export from the ELN: change of style type to justified, improvement of figure size, addition of residues R^1–R^4 in the general procedures and additional material after reviewing.

1 Versions

We used OpenBabel Version 2.4.1 including InChI 1.04 for the generation of the given identifiers.

2 General remarks

^{1}H NMR spectra were recorded on a BRUKER AM 400 (400 MHz) and BRUKER AM 500 (500 MHz) spectrometer. Chemical shifts are given in parts per million (δ/ppm), downfield from tetramethylsilane (TMS) are referenced to chloroform (7.26 ppm) as internal standard. All coupling constants are absolute values and *J* values are expressed in Hertz (Hz). The description of signals include: s = singlet, br. s = broad singlet, d = doublet, bd = broad doublet, t = triplet, dd = doublet of doublets, ddd = doublet of doublet of doublet, dt = doublet of triplets, q = quartet, quin = quintet, sxt – sextet, sept = septet, m = multiplet. The spectra were analyzed according to first order.
^{13}C NMR spectra were recorded on Bruker AM 400 (100 MHz) and Bruker AM 500 (125 MHz) spectrometers. Chemical shifts are expressed in parts per million (ppm, δ) downfield from tetramethylsilane (TMS) and are referenced to chloroform (77.4 ppm) as internal standard.
MS (EI) (electron impact mass spectrometry): Finnigan MAT 90 (70 eV). The molecular fragments are quoted as the relation between mass and charge (m/z), the intensities as a percentage value relative to the intensity of the base signal (100%). HRMS: all HRMS data are recorded with the Finnigan MAT 90 (EI-method).
IR (infrared spectroscopy): ATR spectra were recorded by diamond crystal on Bruker ALPHA-IR. Routine monitoring of reactions were performed using silica gel coated aluminium plates (Merck, silica gel 60, F254) which were analyzed under UV-light at 254 nm and/or dipped into a solution of Seebach reagent (2.5% phosphor molybdic acid, 1.0% cerium(IV) sulfate tetrahydrate and 6.0% sulfuric acid in H_2O, dipping solution) and heated with a heat gun. Solvent mixtures are understood as v/v. Solvents, reagents and chemicals were purchased from Sigma Aldrich, Alfa Aesar, ABCR and VWR and used without further purification unless stated otherwise.

3 General procedures

3.1 General Procedure 1

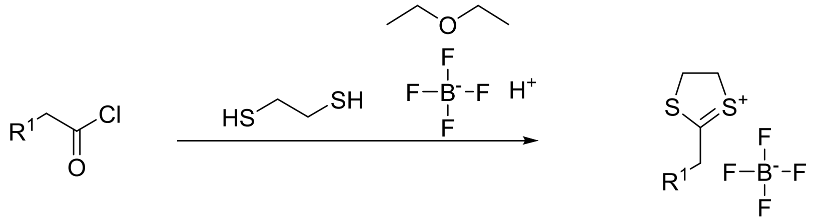

The carbon chloride starting material (1.00 equiv) was dissolved in dry diethyl ether at 0 °C in a flame dried and nitrogen gas flushed flask. Ethanedithol (1.00 equiv) or propanedithiol (1.00 equiv) was added to the reaction mixture followed by tetrafluoroboric acid diethyl ether

complex (1.00 equiv). The reaction was allowed to warm to room temperature and was then refluxed for 30 min. If a precipitate was formed, it was isolated via filtration and washed with dry diethyl ether. If the reaction resulted in the formation of an oily target compound, the reaction mixture was poured into a separation funnel and the oil was separated from the supernatant ether layer. The oil was washed with Et_2O and dried in vacuo.

3.2 General Procedure 2a

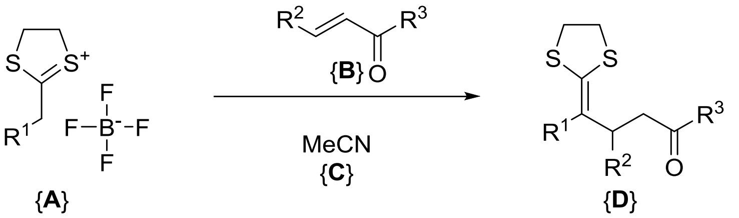

The dithi(ol)anylium tetrafluoroborate {**A**} (1.00 equiv) was dissolved in dry acetonitrile {**C**} in a glass vial at room temperature if not otherwise stated. The α,β-unsaturated ketone {**B**} (1.20 equiv) was added in one portion, the reaction was stirred at room temperature if not otherwise stated and was observed via TLC control. To all reactions, silica gel (3 g) was added after 1 h of reaction time and the solvent was removed via evaporation under reduced pressure. Even though some of the reactions were observed to be finished faster, all of them were reacted for 1 h to allow a good comparison of the results.

3.3 General Procedure 2b

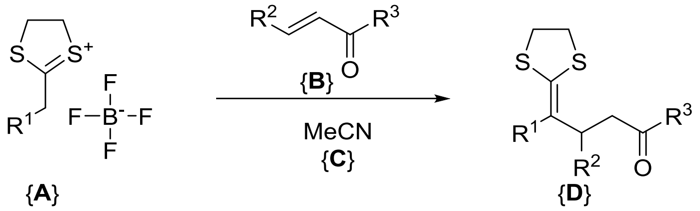

The dithi(ol)anylium tetrafluoroborate {**A**} (1.00 equiv) was dissolved in dry acetonitrile {**C**} in a glass vial at room temperature. The α,β-unsaturated ketone {**B**} (1.20 equiv or 1.50 equiv) was added in one portion, the reaction was stirred at room temperature if not otherwise stated and was observed via TLC control. To all reactions, silica gel (3 g) was added after 13 h of reaction time and the solvent was removed via evaporation under reduced pressure. Even though some of the reactions were observed to be finished faster, all of them were reacted for 13 h to allow a good comparison of the results.

3.4 General Procedure 3

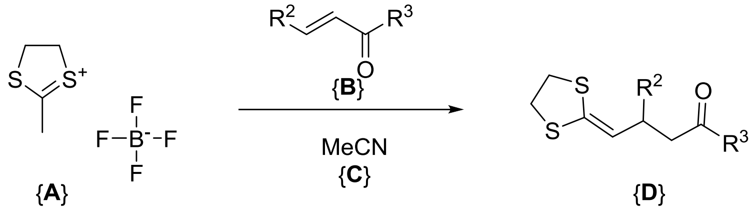

The dithiolanylium tetrafluoroborate {**A|1e**} (1.00 equiv) was dissolved in dry acetonitrile {**C**} in a glass vial at room temperature. The α,β-unsaturated ketone {**B**} (1.00 equiv) was added slowly at room temperature, the reaction was stirred at room temperature and its progress was

observed via TLC control. Silica gel (3 g) was added after 1 h of reaction time and the solvent was removed via evaporation under reduced pressure.

3.5 General Procedure 4

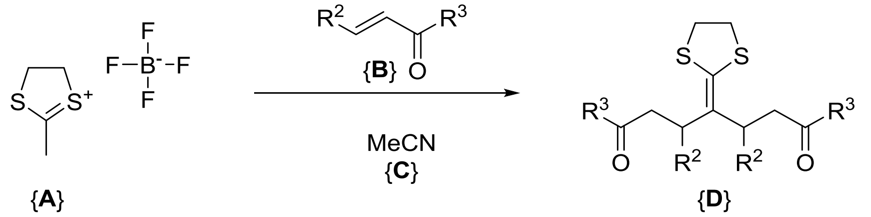

The dithiolanylium tetrafluoroborate {**A|1e**} (1.00 equiv) was dissolved in dry acetonitrile {**C**} in a glass vial at room temperature. The α,β-unsaturated ketone {**B**} (3.00 equiv) was added at room temperature, the reaction was stirred at room temperature and its progress was observed via TLC control. Silica gel (3 g) was added after 1 h of reaction time and the solvent was removed via evaporation under reduced pressure.

3.6 General Procedure 5

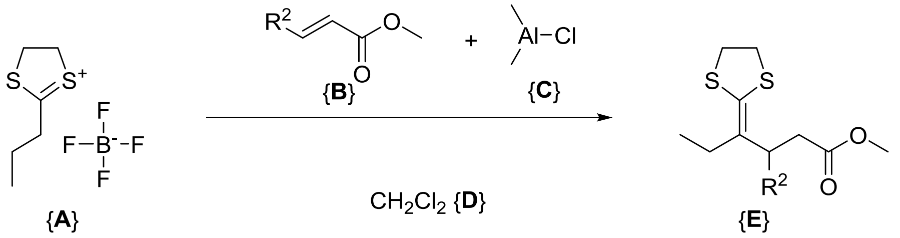

The dithiolanylium tetrafluoroborate {**A|1e**} (1.00 equiv) was dissolved in dry methylene chloride {**D**} in a flame dried glass vial (N_2 atmosphere). The unsaturated ester {**B**} and Me_2AlCl {**C**} were added and the mixture was stirred at rt until full conversion of the starting material was detected. Saturated $NaHCO_3$ (aq) was added and the aqueous phase was extracted with ethyl acetate twice. The combined organic layers were washed with water and brine, then dried over sodium sulfate and the crude product coated onto Celite.

3.7 General Procedure 6

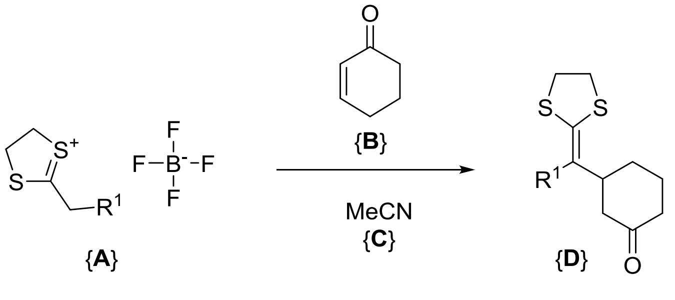

The dithiolanylium tetrafluoroborate {**A**} (1.00 equiv) was dissolved in dry acetonitrile {**C**} in a flame dried glass vial at room temperature and the cyclohexenone {**B|8b**} (1.20 equiv) was added in one portion. The reaction was stirred at room temperature until complete conversion of the starting material was detected, saturated $NaHCO_3$ (aq) was added and the aqueous phase was extracted with ethyl acetate twice. The combined organic layers were washed with water and brine, dried over sodium sulfate and coated onto Celite.

3.8 General Procedure 7

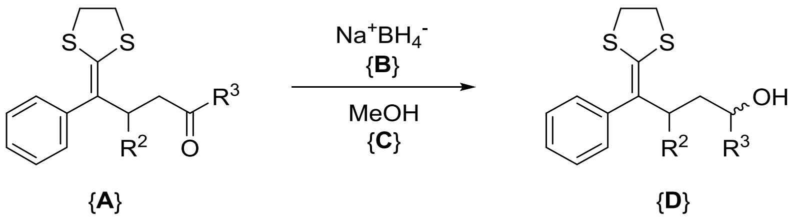

The ketene dithioacetal {**A**} was dissolved in methanol {**C**} and $NaBH_4$ {**B**} was added in small portions until full conversion of the starting material was detected via TLC control. The reaction mixture was quenched by slow addition of water, was extracted twice with ethyl actetate was dried over Na_2SO_4 and was coated on silica gel.

3.9 General Procedure 8

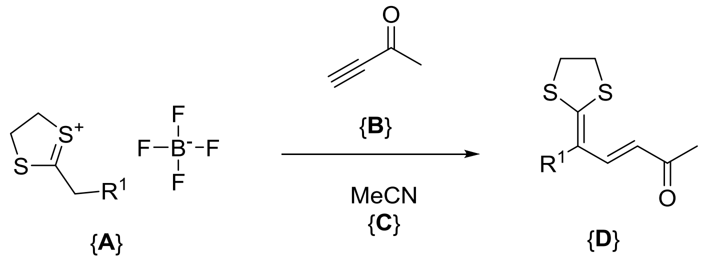

The dithiolanylium tetrafluoroborate {**A**} (1.00 equiv) was dissolved in dry acetonitrile {**C**} in a flame dried glass vial at room temperature and but-3-yn-2-one {**B**|**17**} (1.20 equiv) was added in one portion. The reaction was stirred at room temperature until complete conversion of the starting material was detected, saturated $NaHCO_3$ (aq) was added and the aqueous phase was extracted with ethyl acetate twice. The combined organic layers were washed with water and brine, dried over sodium sulfate and coated onto Celite.

4 Synthesis

4.1 5-(1,3-Dithiolan-2-ylidene)-5-phenylpentan-2-one (**4a**)

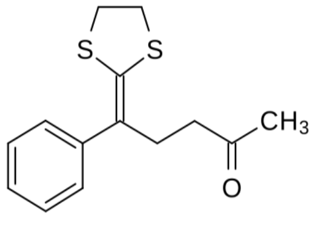

Name: 5-(1,3-dithiolan-2-ylidene)-5-phenylpentan-2-one; Formula: $C_{14}H_{16}OS_2$; CAS: - ; Smiles: CC(=O)CCC(=C1SCCS1)c1ccccc1; InCHI: LXKWSUYFYQCLRX-UHFFFAOYSA-N; Molecular Mass: 264.4062; Exact Mass: 264.0643; EA: C, 63.6; H, 6.1; O, 6.05; S, 24.25.

According to General Procedure 2a: {A|**1a**} 2-benzyl-4,5-dihydro-1,3-dithiol-1-ium tetrafluoroborate (1.000 g, 3.544 mmol, 1.00 equiv); {B|**3a**} but-3-en-2-one (0.298 g, 4.253 mmol, 1.20 equiv); {C} acetonitrile (5.00 mL); Yield {D|**4a**} = 64% (0.600 g, 2.268 mmol).

The obtained crude product was purified via flash-chromatography on silica gel using cyclohexane/ethyl acetate 10:1. R_f = 0.44 (cyclohexane/ethyl acetate 4:1).
^{1}H NMR (400 MHz, $CDCl_3$, ppm) δ = 1.99 (s, 3H), 2.40–2.45 (m, 2H), 2.73–2.79 (m, 2H), 3.18–3.23 (m, 2H), 3.32–3.37 (m, 2H), 7.15–7.21 (m, 3H), 7.23–7.29 (m, 2H); ^{13}C NMR (100 MHz, $CDCl_3$, ppm) δ = 29.8, 32.8, 37.8, 37.9, 41.5, 126.3, 127.1, 128.0 (2C), 128.3 (2C), 134.3, 142.0, 208.1; EI (*m/z*, 70 eV, 50 °C): 264 (70) $[M]^+$, 207 (100), 181 (33), 131 (33), 69 (51); HRMS–EI (*m/z*): [M] calcd for $C_{14}H_{16}OS_2$, 264.0643; found, 264.0642; IR (ATR, ṽ): 2923, 1710, 1595, 1489, 1439, 1420, 1357, 1278, 1158, 1073, 1027, 899, 849, 791, 761, 700, 642, 597, 534, 472, 430 cm^{-1}.

4.2 5-(1,3-Dithian-2-ylidene)-5-phenylpentan-2-one (**5a**)

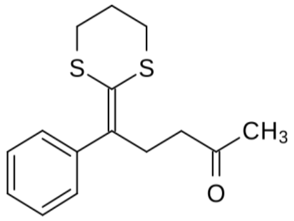

Name: 5-(1,3-dithian-2-ylidene)-5-phenylpentan-2-one; Formula: $C_{15}H_{18}OS_2$; CAS: - ; Smiles: CC(=O)CCC(=C1SCCCS1)c1ccccc1; InCHI: HLVFYRZSXYKKMF-UHFFFAOYSA-N; Molecular Mass: 278.4328; Exact Mass: 278.0799; EA: C, 64.71; H, 6.52; O, 5.75; S, 23.03.

According to General Procedure 2a: {A|**2a**} 2-benzyl-5,6-dihydro-4H-1,3-dithiin-1-ium tetrafluoroborate (0.150 g, 0.506 mmol, 1.00 equiv); {B|**3a**} but-3-en-2-one (0.043 g, 0.608 mmol, 1.20 equiv); {C} acetonitrile (5.00 mL); Yield {D|**5a**} = 32% (0.045 g, 0.161 mmol).
The obtained crude product was purified via flash-chromatography on silica gel using cyclohexane/ethyl acetate 10:1. R_f = 0.63 (cyclohexane/ethyl acetate 2:1).
^{1}H NMR (500 MHz, $CDCl_3$, ppm) δ = 2.06 (s, 3H), 2.11 (quin, *J* = 6.3 Hz, 2H), 2.40–2.45 (m, 2H), 2.80 (t, *J* = 6.2 Hz, 2H), 2.90–2.95 (m, 2H), 2.98 (t, *J* = 6.2 Hz, 2H), 7.14–7.18 (m, 2H), 7.26–7.31 (m, 1H), 7.33–7.38 (m, 2H); ^{13}C NMR (125 MHz, $CDCl_3$, ppm) δ = 24.2, 29.4, 29.7 (2C), 30.3, 41.5, 125.6, 127.2, 128.1 (2C), 128.6 (2C), 139.9, 140.6, 207.9; EI (*m/z*, 70 eV, 70 °C): 278 (72) $[M]^+$, 221 (100), 147 (31), 115 (15), 103 (23); HRMS–EI (*m/z*): $[M]^+$ calcd for $C_{15}H_{18}OS_2$, 278.0794; found, 278.0795; IR (ATR, ṽ): 2895, 1703, 1598, 1488, 1438, 1417, 1359, 1279, 1240, 1157, 1113, 1072, 1023, 998, 962, 910, 818, 786, 759, 699, 653, 616, 594, 542, 502, 421 cm^{-1}.

4.3 6-(1,3-Dithiolan-2-ylidene)-6-phenylhexan-3-one (**4b**)

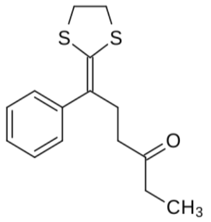

Name: 6-(1,3-dithiolan-2-ylidene)-6-phenylhexan-3-one; Formula: $C_{15}H_{18}OS_2$; CAS: - ; Smiles: CCC(=O)CCC(=C1SCCS1)c1ccccc1; InCHI: MQHLDGLJNBYOQQ-UHFFFAOYSA-N; Molecular Mass: 278.4328; Exact Mass: 278.0799; EA: C, 64.71; H, 6.52; O, 5.75; S, 23.03.

According to General Procedure 2a: {A|**1a**} 2-benzyl-4,5-dihydro-1,3-dithiol-1-ium tetrafluoroborate (1.000 g, 3.544 mmol, 1.00 equiv); {B|**3b**} pent-1-en-3-one (0.358 g, 4.254 mmol, 1.20 equiv); {C} acetonitrile (5.00 mL); Yield {D|**4b**} = 62% (0.615 g, 2.207 mmol).
The obtained crude product was purified via flash-chromatography on silica gel using cyclohexane/ethyl acetate 10:1. R_f = 0.53 (cyclohexane/ethyl acetate 4:1).
^{1}H NMR (400 MHz, $CDCl_3$, ppm) δ = 0.92 (t, *J* = 7.3 Hz, 3H), 2.26 (q, *J* = 7.3 Hz, 2H), 2.36–2.42 (m, 2H), 2.73–2.80 (m, 2H), 3.18–3.24 (m, 2H), 3.32–3.37 (m, 2H), 7.14–7.21 (m, 3H), 7.23–7.29 (m, 2H); ^{13}C NMR (100 MHz, $CDCl_3$, ppm) δ = 7.7, 32.9, 35.8, 37.8, 37.8, 40.2, 126.5, 127.0, 128.0 (2C), 128.3 (2C), 134.2, 142.0, 210.7; EI (*m/z*, 70 eV, 50 °C): 278 (68) $[M]^+$, 221 (21), 207 (100), 181 (33), 131 (32), 69 (51), 57 (23); HRMS–EI (*m*/*z*): [M] calcd for $C_{15}H_{18}OS_2$, 278.0799; found, 278.0800; IR (ATR, ṽ): 2972, 2925, 1709, 1596, 1490, 1440, 1419, 1360, 1278, 1149, 1111, 1054, 979, 919, 849, 813, 762, 700, 609, 544, 421 cm^{-1}.

4.4 6-(1,3-Dithian-2-ylidene)-6-phenylhexan-3-one (**5b**)

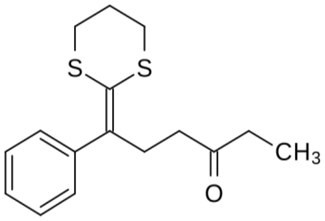

Name: 6-(1,3-dithian-2-ylidene)-6-phenylhexan-3-one; Formula: $C_{16}H_{20}OS_2$; CAS: - ; Smiles: CCC(=O)CCC(=C1SCCCS1)c1ccccc1; InCHI: YMTMOMPRUVRPTP-UHFFFAOYSA-N; Molecular Mass: 292.4594; Exact Mass: 292.0956; EA: C, 65.71; H, 6.89; O, 5.47; S, 21.93.

According to General Procedure 2a: {A|**2a**} 2-benzyl-5,6-dihydro-4H-1,3-dithiin-1-ium tetrafluoroborate (0.150 g, 0.506 mmol, 1.00 equiv); {B|**3b**} pent-1-en-3-one (0.051 g, 0.608 mmol, 1.20 equiv); {C} acetonitrile (5.00 mL); Yield {D|**5b**} = 31% (0.046 g, 0.158 mmol).
The obtained crude product was purified via flash-chromatography on silica gel using cyclohexane/ethyl acetate 10:1. R_f = 0.60 (cyclohexane/ethyl acetate 2:1).
^{1}H NMR (500 MHz, $CDCl_3$, ppm) δ = 0.98 (t, *J* = 7.3 Hz, 3H), 2.07–2.13 (m, 2H), 2.33 (q, *J* = 7.3 Hz, 2H), 2.36–2.42 (m, 2H), 2.75–2.82 (m, 2H), 2.90–2.95 (m, 2H), 2.95–2.99 (m, 2H), 7.12–7.16 (m, 2H), 7.25–7.30 (m, 1H), 7.32–7.37 (m, 2H); ^{13}C NMR (125 MHz, $CDCl_3$, ppm) δ = 7.73, 24.3, 29.5, 29.8, 30.5, 35.7, 40.2, 125.5, 127.3, 128.2 (2C), 128.6 (2C), 140.2, 140.7, 210.6; EI (*m/z*, 70 eV): 292 (76) $[M]^+$, 235 (23), 221 (100), 185 (8), 161 (17), 147 (23); HRMS–EI (*m*/*z*): $[M]^+$ calcd for $C_{16}H_{20}OS_2$, 292.0950; found, 292.0950; IR (ATR, ṽ): 2970, 2931, 1709, 1596, 1575, 1488, 1438, 1415, 1359, 1299, 1276, 1240, 1111, 1048, 1026, 977, 912, 811, 762, 734, 700, 603, 552, 433 cm^{-1}.

4.5 5-(1,3-Dithiolan-2-ylidene)-4-methyl-5-phenylpentan-2-one (**4c**)

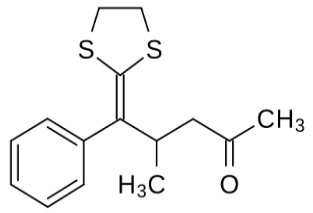

Name: 5-(1,3-dithiolan-2-ylidene)-4-methyl-5-phenylpentan-2-one; Formula: $C_{15}H_{18}OS_2$; CAS: - ; Smiles: CC(C(=C1SCCS1)c1ccccc1)CC(=O)C; InCHI: RCALJRZMLQRHLX-UHFFFAOYSA-N; Molecular Mass: 278.4328; Exact Mass: 278.0799; EA: C, 64.71; H, 6.52; O, 5.75; S, 23.03.

According to General Procedure 2a: {A|**1a**} 2-benzyl-4,5-dihydro-1,3-dithiol-1-ium tetrafluoroborate (0.150 g, 0.532 mmol, 1.00 equiv); {B|**3b**} pent-1-en-3-one (0.049 g, 0.583 mmol, 1.10 equiv); {C} acetonitrile (5.00 mL); Yield {D|**4c**} = 94% (0.140 g, 0.501 mmol).
The obtained crude product was purified via flash-chromatography on silica gel using cyclohexane/ethyl acetate 10:1. R_f = 0.64 (cyclohexane/ethyl acetate 2:1).
^{1}H NMR (500 MHz, $CDCl_3$, ppm) δ = 1.06 (d, *J* = 7.0 Hz, 3H), 2.10 (s, 3H), 2.28 (dd, *J* = 16.4 Hz, *J* = 8.0 Hz, 1H), 2.53 (dd, *J* = 16.3 Hz, *J* = 6.3 Hz, 1H), 3.19–3.25 (m, 2H), 3.36–3.43 (m, 3H), 7.07–7.15 (m, 2H), 7.26–7.39 (m, 3H); ^{13}C NMR (125 MHz, $CDCl_3$, ppm) δ = 18.9, 30.0, 37.1, 37.3, 37.8, 48.9, 127.1, 128.1 (2C), 129.1 (2C), 130.7, 134.4, 140.6, 207.4; EI (*m/z*, 70 eV, 90 °C): 278 (41) $[M]^+$, 221 (100), 185 (8); HRMS–EI (*m*/*z*): $[M]^+$ calcd for $C_{15}H_{18}OS_2$, 278.0794; found, 278.0792; IR (ATR, ṽ): 2961, 2924, 1711, 1597, 1582, 1488, 1438, 1420, 1358, 1279, 1241, 1161, 1120, 1072, 1029, 978, 926, 869, 845, 750, 702, 665, 643, 605, 539, 473 cm^{-1}.

4.6 5-(1,3-Dithian-2-ylidene)-4-methyl-5-phenylpentan-2-one (**5c**)

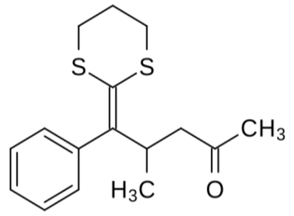

Name: 5-(1,3-dithian-2-ylidene)-4-methyl-5-phenylpentan-2-one; Formula: $C_{16}H_{20}OS_2$; CAS: - ; Smiles: CC(C(=C1SCCCS1)c1ccccc1)CC(=O)C; InCHI: MSEWRYWMSSBPBP-UHFFFAOYSA-N; Molecular Mass: 292.4594; Exact Mass: 292.0956; EA: C, 65.71; H, 6.89; O, 5.47; S, 21.93.

According to General Procedure 2a: {A|**2a**} 2-benzyl-5,6-dihydro-4H-1,3-dithiin-1-ium tetrafluoroborate (0.150 g, 0.506 mmol, 1.00 equiv); {B|**3b**} pent-1-en-3-one (0.051 g, 0.608 mmol, 1.20 equiv); {C} acetonitrile (5.00 mL); Yield {D|**5c**} = 36% (0.053 g, 0.182 mmol).
The obtained crude product was purified via flash-chromatography on silica gel using cyclohexane/ethyl acetate 10:1. R_f = 0.64 (cyclohexane/ethyl acetate 2:1).
^{1}H NMR (500 MHz, $CDCl_3$, ppm) δ = 1.00 (d, *J* = 6.9 Hz, 3H), 2.05–2.14 (m, 5H), 2.24 (dd, *J* = 16.2 Hz, *J* = 7.8 Hz, 1H), 2.43 (dd, *J* = 16.2 Hz, *J* = 6.6 Hz, 1H), 2.74–2.85 (m, 2H), 2.91–3.05 (m, 2H), 3.85–3.94 (m, 1H), 7.01–7.02 (m, 2H), 7.27–7.38 (m, 3H); ^{13}C NMR (125 MHz, $CDCl_3$, ppm) δ = 19.1, 24.4, 29.4, 29.5, 30.1, 33.4, 49.0, 126.1, 127.1, 128.1 (2C),

129.3 (2C), 138.9, 143.9, 207.6; EI (*m/z*, 70 eV, 50 °C): 292 (47) $[M]^+$, 235 (100), 218 (15), 185 (13); HRMS–EI (*m*/*z*): $[M]^+$ calcd for $C_{16}H_{20}OS_2$, 292.0950; found, 292.0951; IR (ATR, ṽ): 2960, 2925, 1711, 1597, 1560, 1488, 1438, 1417, 1357, 1299, 1278, 1240, 1161, 1119, 1072, 1029, 912, 866, 817, 783, 745, 703, 641, 541 cm^{-1}.

4.7 6-(1,3-Dithiolan-2-ylidene)-5-methyl-6-phenylhexan-3-one (**4d**)

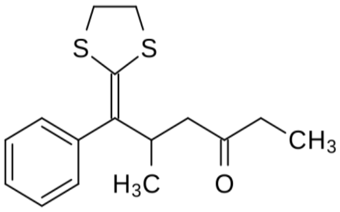

Name: 6-(1,3-dithiolan-2-ylidene)-5-methyl-6-phenylhexan-3-one; Formula: $C_{16}H_{20}OS_2$; CAS: - ; Smiles: CCC(=O)CC(C(=C1SCCS1)c1ccccc1)C; InCHI: GQDXLOBJYIWLJL-UHFFFAOYSA-N; Molecular Mass: 292.4594; Exact Mass: 292.0956; EA: C, 65.71; H, 6.89; O, 5.47; S, 21.93.

According to General Procedure 2a: {A|**1a**} 2-benzyl-4,5-dihydro-1,3-dithiol-1-ium tetrafluoroborate (0.150 g, 0.532 mmol, 1.00 equiv); {B|**3d**} (E)-hex-4-en-3-one (0.060 g, 0.611 mmol, 1.15 equiv); {C} acetonitrile (5.00 mL); Yield {D|**4d**} = 97% (0.151 g, 0.517 mmol).
The obtained crude product was purified via flash-chromatography on silica gel using cyclohexane/ethyl acetate 10:1. R_f = 0.53 (cyclohexane/ethyl acetate 2:1).
^{1}H NMR (500 MHz, $CDCl_3$, ppm) δ = 1.02 (t, *J* = 7.3 Hz, 3H), 1.05 (d, *J* = 6.9 Hz, 3H), 2.26 (dd, *J* = 16.3 Hz, *J* = 7.9 Hz, 1H), 2.30–2.40 (m, 2H), 2.50 (dd, *J* = 16.2 Hz, *J* = 6.4 Hz, 1H), 3.20–3.25 (m, 2H), 3.36–3.45 (m, 3H), 7.08–7.13 (m, 2H), 7.26–7.38 (m, 3H); ^{13}C NMR (125 MHz, $CDCl_3$, ppm) δ = 7.6, 18.9, 35.9, 37.1, 37.4, 37.8, 47.7, 127.1, 128.1 (2C), 129.2 (2C), 130.8, 134.3, 140.7, 209.9; EI (*m/z*, 70 eV, 40 °C): 292 (3) $[M]^+$, 221 (7), 84 (100); HRMS–EI (*m*/*z*): $[M]^+$ calcd for $C_{16}H_{20}OS_2$, 292.0950; found, 292.0949; IR (ATR, ṽ): 2966, 2925, 1708, 1597, 1581, 1488, 1454, 1439, 1409, 1358, 1278, 1149, 1113, 1072, 1024, 977, 912, 844, 796, 751, 732, 703, 645, 550 cm^{-1}.

4.8 6-(1,3-Dithian-2-ylidene)-5-methyl-6-phenylhexan-3-one (**5d**)

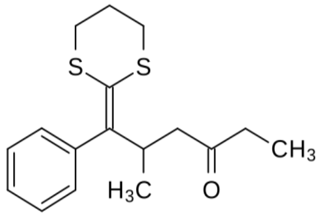

Name: 6-(1,3-dithian-2-ylidene)-5-methyl-6-phenylhexan-3-one; Formula: $C_{17}H_{22}OS_2$; CAS: - ; Smiles: CCC(=O)CC(C(=C1SCCCS1)c1ccccc1)C; InCHI: GIVITTWYMCQGJB-UHFFFAOYSA-N; Molecular Mass: 306.4860; Exact Mass: 306.1112; EA: C, 66.62; H, 7.24; O, 5.22; S, 20.92.

According to General Procedure 2a: {A|**2a**} 2-benzyl-5,6-dihydro-4H-1,3-dithiin-1-ium tetrafluoroborate (0.150 g, 0.506 mmol, 1.00 equiv); {B|**3d**} (E)-hex-4-en-3-one (0.060 g,

0.608 mmol, 1.20 equiv); {C} acetonitrile (5.00 mL); Yield {D|**5d**} = 37% (0.058 g, 0.190 mmol).
The obtained crude product was purified via flash-chromatography on silica gel using cyclohexane/ethyl acetate 10:1. R_f = 0.64 (cyclohexane/ethyl acetate 2:1).
^{1}H NMR (500 MHz, $CDCl_3$, ppm) δ = 0.99 (d, *J* = 7.0 Hz, 3H), 1.02 (t, *J* = 7.3 Hz, 3H), 2.09 (quin, *J* = 6.3 Hz, 2H), 2.22 (dd, *J* = 16.2 Hz, *J* = 7.8 Hz, 1H), 2.27–2.44 (m, 3H), 2.79 (td, *J* = 6.3 Hz, *J* = 1.53 Hz, 2H), 2.91–3.05 (m, 2H), 3.85–3.93 (m, 1H), 6.98–7.04 (m, 2H), 7.28–7.38 (m, 3H); ^{13}C NMR (125 MHz, $CDCl_3$, ppm) δ = 7.7, 19.2, 24.4, 29.4, 29.5, 33.4, 36.0, 47.7, 125.9, 127.1, 128.0 (2C), 129.3 (2C), 139.0, 144.1, 210.1; EI (*m/z*, 70 eV): 306 (56) $[M]^+$, 235 (100), 199 (15); HRMS–EI (*m/z*): $[M]^+$ calcd for $C_{17}H_{22}OS_2$, 306.1107; found, 306.1106; IR (ATR, ṽ): 2965, 2930, 1709, 1597, 1487, 1454, 1413, 1359, 1299, 1277, 1113, 1023, 913, 857, 787, 749, 702, 552 cm^{-1}.

4.9 4-((1,3-Dithiolan-2-ylidene)(phenyl)methyl)-5-methylhexan-2-one (**4e**)

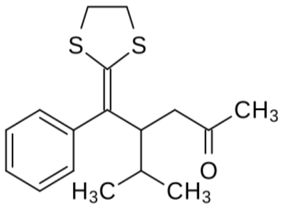

Name: 4-((1,3-Dithiolan-2-ylidene)(phenyl)methyl)-5-methylhexan-2-one; Formula: $C_{17}H_{22}OS_2$; CAS: - ; Smiles: CC(C(C(=C1SCCS1)c1ccccc1)CC(=O)C)C; InChI: AJUPULBPXRSZPK-UHFFFAOYSA-N; Molecular Mass: 306.4860; Exact Mass: 306.1112; EA: C, 66.62; H, 7.24; O, 5.22; S, 20.92.

According to General Procedure 2b: {A|**1a**} 2-benzyl-4,5-dihydro-1,3-dithiol-1-ium tetrafluoroborate (0.156 g, 0.551 mmol, 1.00 equiv); {B|**3e**} (E)-5-methylhex-3-en-2-one (0.074 g, 0.660 mmol, 1.20 equiv); {C} acetonitrile (5.00 mL); Yield {D|**4e**} = 85% (0.144 g, 0.471 mmol).
The obtained crude product was purified via flash-chromatography on silica gel using cyclohexane/ethyl acetate 10:1. R_f = 0.63 (cyclohexane/ethyl acetate 2:1).
^{1}H NMR (500 MHz, $CDCl_3$, ppm) δ = 0.90 (d, *J* = 6.6 Hz, 3H), 1.12 (d, *J* = 6.6 Hz, 3H), 1.60–1.69 (m, 1H), 2.16 (s, 3H), 2.37 (dd, *J* = 16.6 Hz, *J* = 8.9 Hz, 1H), 2.53 (dd, *J* = 16.6 Hz, *J* = 4.8 Hz, 1H), 3.04 (td, *J* = 9.3 Hz, *J* = 4.7 Hz, 1H), 3.20–3.24 (m, 2H), 3.36–3.40 (m, 2H), 7.13–7.16 (m, 2H), 7.27–7.31 (m, 1H), 7.33–7.37 (m, 2H); ^{13}C NMR (125 MHz, $CDCl_3$, ppm) δ = 20.6, 21.2, 29.8, 31.1, 37.1, 37.7, 45.8, 50.3, 127.1, 128.1 (2C), 129.1, 129.3 (2C), 136.8, 141.2, 207.9; EI (*m/z*, 70 eV, 60 °C): 306 (38) $[M]^+$, 263 (100), 221 (52), 191 (11), 113 (13), 69 (19); HRMS–EI (*m/z*): $[M]^+$ calcd for $C_{17}H_{22}OS_2$, 306.1107; found, 306.1108; IR (ATR, ṽ): 2964, 2924, 1710, 1586, 1472, 1440, 1404, 1369, 1301, 1278, 1169, 1110, 1071, 1026, 917, 889, 843, 816, 793, 749, 702, 652, 590, 563, 501, 425, 400 cm^{-1}.

4.10 4-((1,3-Dithiolan-2-ylidene)(phenyl)methyl)octan-2-one (**4f**)

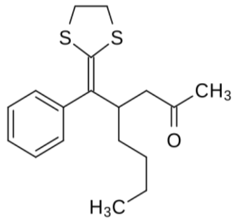

Name: 4-((1,3-dithiolan-2-ylidene)(phenyl)methyl)octan-2-one; Formula: $C_{18}H_{24}OS_2$; CAS: - ; Smiles: CCCCC(C(=C1SCCS1)c1ccccc1)CC(=O)C; InCHI: XLPWXCGXHOGTNX-UHFFFAOYSA-N; Molecular Mass: 320.5126; Exact Mass: 320.1269; EA: C, 67.45; H, 7.55; O, 4.99; S, 20.01.

According to General Procedure 2a: {A|**1a**} 2-benzyl-4,5-dihydro-1,3-dithiol-1-ium tetrafluoroborate (0.156 g, 0.553 mmol, 1.00 equiv); {B|**3f**} (E)-oct-3-en-2-one (0.084 g, 0.666 mmol, 1.21 equiv); {C} acetonitrile (5.00 mL); Yield {D|**4f**} = 90% (0.160 g, 0.498 mmol).
The obtained crude product was purified via flash-chromatography on silica gel using cyclohexane/ethyl acetate 10:1 + NEt_3 (1%). R_f = 0.45 (cyclohexane/ethyl acetate 2:1).
^{1}H NMR (500 MHz, $CDCl_3$, ppm) δ = 1.04–1.09 (t, *J* = 7.2 Hz, 3H), 1.44 -1.63 (m, 6H), 2.29 (s, 3H), 2.49 (dd, *J* = 16.6 Hz, *J* = 7.0 Hz ,1H), 2.63(dd, *J* = 16.4 Hz, *J* = 7.0 Hz, 1H), 3.37–3.41 (m, 2H), 3.41–3.48 (m, 1H), 3.54–3.57 (m, 2H), 7.24–7.28 (m, 2H), 7.43–7.48 (m, 1H), 7.49–7.54 (m, 2H); ^{13}C NMR (125 MHz, $CDCl_3$, ppm) δ = 13.9, 22.6, 29.6, 30.0, 33.2, 37.1, 37.8, 43.2, 47.9, 127.1, 128.2 (2C), 129.3 (2C), 129.4, 135.9, 140.7, 207.6; EI (*m/z*, 70 eV, 120 °C): 320 (58) $[M]^+$, 263 (100), 221 (22), 195 (20), 152 (49); HRMS–EI (*m*/*z*): $[M]^+$ calcd for $C_{18}H_{24}OS_2$, 320.1263; found, 320.1265; IR (ATR, ṽ): 2953, 2923, 2855, 1711, 1597, 1580, 1489, 1438, 1420, 1356, 1278, 1159, 1105, 1072, 1031, 978, 847, 750, 702, 645, 541 cm^{-1}.

4.11 4-((1,3-Dithian-2-ylidene)(phenyl)methyl)octan-2-one (**5f**)

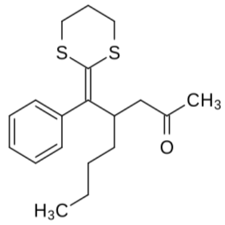

Name: 4-((1,3-dithian-2-ylidene)(phenyl)methyl)octan-2-one; Formula: $C_{19}H_{26}OS_2$; CAS: - ; Smiles: CCCCC(C(=C1SCCCS1)c1ccccc1)CC(=O)C; InCHI: UOHJUSHNULFLRZ-UHFFFAOYSA-N; Molecular Mass: 334.5391; Exact Mass: 334.1425; EA: C, 68.21; H, 7.83; O, 4.78; S, 19.17.

According to General Procedure 2a: {A|**2a**} 2-benzyl-5,6-dihydro-4H-1,3-dithiin-1-ium tetrafluoroborate (0.156 g, 0.525 mmol, 1.00 equiv); {B|**3f**} (E)-oct-3-en-2-one (0.080 g, 0.630 mmol, 1.20 equiv); {C} acetonitrile (5.00 mL); Yield {D|**5f**} = 64% (0.113 g, 0.337 mmol).
The obtained crude product was purified via flash-chromatography on silica gel using cyclohexane/ethyl acetate 10:1. R_f = 0.73 (cyclohexane/ethyl acetate 2:1 + 1% NEt_3).
^{1}H NMR (500 MHz, $CDCl_3$, ppm) δ = 0.90–0.96 (m, 3H), 1.26–1.45 (m, 6H), 2.05–2.15 (m, 2H), 2.12–2.15 (m, 3H), 2.27–2.42 (m, 2H), 2.75–2.86 (m, 2H), 2.91–2.98 (m, 1H), 2.99–

3.06 (m, 1H), 3.80–3.88 (m, 1H), 6.99–7.04 (m, 2H), 7.28–7.39 (m, 3H); ^{13}C NMR (125 MHz, $CDCl_3$, ppm) δ = 14.0, 22.6, 24.4, 29.5, 29.5, 29.6, 30.0, 33.0, 38.7, 47.7, 127.0, 127.6, 128.0 (2C), 129.2 (2C), 138.8, 142.6, 207.6; EI (*m/z*, 70 eV, 60 °C): 334 (33) $[M]^+$, 277 (61), 235 (22), 208 (70), 195 (26), 169 (22), 152 (100), 134 (80), 119 (29), 109 (33), 105 (37), 95 (28), 91 (21), 81 (26), 69 (55), 55 (28); HRMS–EI (*m*/*z*): $[M]^+$ calcd for $C_{19}H_{26}OS_2$, 334.1420; found, 334.1421; IR (ATR, ṽ): 2921, 2852, 1703, 1596, 1560, 1487, 1419, 1354, 1301, 1279, 1160, 1121, 1072, 1031, 914, 878, 783, 751, 727, 707, 645, 609, 541, 444 cm^{-1}.

4.12 5-(1,3-Dithiolan-2-ylidene)-4,5-diphenylpentan-2-one (**4g**)

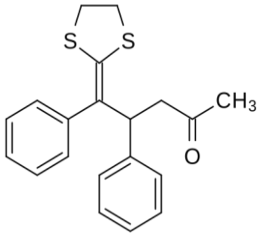

Name: 5-(1,3-dithiolan-2-ylidene)-4,5-diphenylpentan-2-one; Formula: $C_{20}H_{20}OS_2$; CAS: - ; Smiles: CC(=O)CC(C(=C1SCCS1)c1ccccc1)c1ccccc1; InCHI: LRVYWKBWOILOMV-UHFFFAOYSA-N; Molecular Mass: 340.5022; Exact Mass: 340.0956; EA: C, 70.55; H, 5.92; O, 4.7; S, 18.83.

According to General Procedure 2b: {A|**1a**} 2-benzyl-4,5-dihydro-1,3-dithiol-1-ium tetrafluoroborate (0.156 g, 0.551 mmol, 1.00 equiv); {B|**3g**} (E)-4-phenylbut-3-en-2-one (0.121 g, 0.827 mmol, 1.50 equiv); {C} acetonitrile (5.00 mL); Yield {D|**4g**} = 56% (0.105 g, 0.310 mmol).

The obtained crude product was purified via flash-chromatography on silica gel using cyclohexane/ethyl acetate 10:1. R_f = 0.72 (cyclohexane/ethyl acetate 2:1).

^{1}H NMR (500 MHz, $CDCl_3$, ppm) δ = 2.14 (s, 3H), 2.88 (dd, *J* = 7.5 Hz, *J* = 2.1 Hz, 2H), 3.27–3.31 (m, 2H), 3.49 (td, *J* = 5.9 Hz, *J* = 2.5 Hz, 2H), 4.79 (t, *J* = 7.5 Hz, 1H), 6.81–6.84 (m, 2H), 7.18–7.21 (m, 2H), 7.23–7.32 (m, 6H); ^{13}C NMR (125 MHz, $CDCl_3$, ppm) δ = 30.2, 37.4, 38.1, 46.3, 47.7, 126.5, 127.2, 127.6 (2C), 128.0 (2C), 128.1 (2C), 129.1, 129.7 (2C), 136.5, 140.4, 141.4, 206.6; EI (*m/z*, 70 eV, 90 °C): 340 (27) $[M]^+$, 283 (39), 223 (21), 146 (56), 131 (81), 105 (100), 84 (63), 77 (66), 69 (30); HRMS–EI (*m*/*z*): $[M]^+$ calcd for $C_{20}H_{20}OS_2$, 340.0950; found, 340.0949; IR (ATR, ṽ): 3023, 1702, 1598, 1577, 1490, 1423, 1365, 1281, 1252, 1164, 1069, 1016, 847, 828, 783, 754, 739, 698, 669, 557, 510, 429 cm^{-1}.

4.13 5-(1,3-Dithian-2-ylidene)-4,5-diphenylpentan-2-one (**5g**)

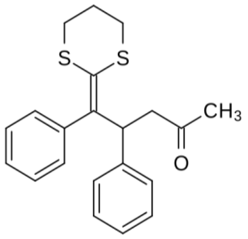

Name: 5-(1,3-dithian-2-ylidene)-4,5-diphenylpentan-2-one; Formula: $C_{21}H_{22}OS_2$; CAS: - ; Smiles: CC(=O)CC(C(=C1SCCCS1)c1ccccc1)c1ccccc1; InCHI: KDMUVXXDJMVQGI-UHFFFAOYSA-N; Molecular Mass: 354.5288; Exact Mass: 354.1112; EA: C, 71.14; H, 6.25; O, 4.51; S, 18.09.

According to General Procedure 2b: {A|**2a**} 2-benzyl-5,6-dihydro-4H-1,3-dithiin-1-ium tetrafluoroborate (0.168 g, 0.566 mmol, 1.00 equiv); {B|**3g**} (E)-4-phenylbut-3-en-2-one (0.124 g, 0.848 mmol, 1.50 equiv); {C} acetonitrile (5.00 mL); Yield {D|**5g**} = 43% (0.086 g, 0.241 mmol).
The obtained crude product was purified via flash-chromatography on silica gel using cyclohexane/ethyl acetate 10:1. R_f = 0.66 (cyclohexane/ethyl acetate 2:1).
^{1}H NMR (500 MHz, $CDCl_3$, ppm) δ = 2.12 (s, 3H), 2.10 – 2.16 (m, 2H), 2.73–2.89 (m, 4H), 3.02 (dt, *J* = 13.7 Hz, *J* = 6.1 Hz, 1H), 3.08–3.17 (m, 1H), 5.29 (t, *J* = 7.6 Hz, 1H), 6.65 (d, *J* = 6.4 Hz, 2H), 7.11 – 7.13 (m, 2H), 7.21 – 7.30 (m, 6H); ^{13}C NMR (125 MHz, $CDCl_3$, ppm) δ = 24.3, 29.6, 29.7, 30.2, 43.5, 46.0, 126.5, 127.3, 127.8 (2C), 127.9 (2C), 128.0, 128.2 (2C), 129.8 (2C), 138.4, 141.2, 141.9, 206.8; EI (*m/z*, 70 eV, 80 °C): 354 (48) $[M]^+$, 297 (45), 223 (21), 137 (35), 123 (100), 105 (63), 84 (63), 79 (20), 77 (27), 69 (34); HRMS–EI (*m*/*z*): [M] calcd for $C_{21}H_{22}OS_2$, 354.1107; found, 354.1108; IR (ATR, ṽ): 2903, 1700, 1595, 1495, 1414, 1360, 1276, 1226, 1159, 1070, 1032, 912, 886, 844, 815, 782, 766, 743, 700, 638, 592, 566, 552, 537, 429 cm^{-1}.

4.14 4-(1,3-Dithiolan-2-ylidene)-1,3,4-triphenylbutan-1-one (**4h**)

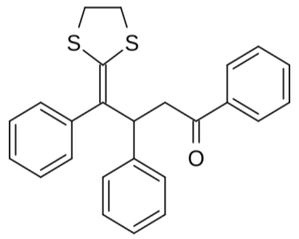

Name: 4-(1,3-Dithiolan-2-ylidene)-1,3,4-triphenylbutan-1-one; Formula: $C_{25}H_{22}OS_2$; CAS: - ; Smiles: O=C(c1ccccc1)CC(C(=C1SCCS1)c1ccccc1)c1ccccc1; InChI: GEPFNJDSCKCSTO-UHFFFAOYSA-N; Molecular Mass: 402.5716; Exact Mass: 402.1112; EA: C, 74.59; H, 5.51; O, 3.97; S, 15.93.

According to General Procedure 2b: {A|**1a**} 2-benzyl-4,5-dihydro-1,3-dithiol-1-ium tetrafluoroborate (0.150 g, 0.532 mmol, 1.00 equiv); {B|**3h**} (E)-1,3-diphenylprop-2-en-1-one (0.166 g, 0.798 mmol, 1.50 equiv); {C} acetonitrile (5.00 mL); Yield {D|**4h**} = 79% (0.170 g, 0.423 mmol).
The obtained crude product was purified via flash-chromatography on silica gel using cyclohexane/ethyl acetate 10:1. R_f = 0.38 (cyclohexane/ethyl acetate 4:1).
^{1}H NMR (500 MHz, $CDCl_3$, ppm) δ = 3.25–3.34 (m, 2H), 3.36–3.42 (m, 1H), 3.44–3.54 (m, 3H), 5.02 (t, *J* = 7.2 Hz, 1H), 6.82–6.85 (m, 2H), 7.22–7.33 (m, 8H), 7.44–7.48 (m, 2H), 7.55–7.59 (m, 1H), 7.92–7.95 (m, 2H); ^{13}C NMR (125 MHz, $CDCl_3$, ppm) δ = 37.4, 38.1, 41.1, 47.7, 126.4, 127.2, 127.7 (2C), 128.0 (2C), 128.0 (2C), 128.1 (2C), 128.4 (2C), 129.3, 129.8 (2C), 132.9, 136.5, 136.9, 140.6, 141.7, 197.9; EI (*m/z*, 70 eV, 170 °C): 402 (63) $[M]^+$, 309 (39), 283 (85), 223 (41), 209 (27), 178 (29), 121 (24), 105 (100), 77 (82), 69 (40); HRMS–EI (*m*/*z*): $[M]^+$ calcd for $C_{25}H_{22}OS_2$, 402.1107; found, 402.1108; IR (ATR, ṽ): 3022, 2930, 1674, 1595, 1577, 1487, 1448, 1324, 1277, 1251, 1208, 1067, 1000, 908, 849, 748, 689, 661, 613, 588, 568, 544, 512, 409 cm^{-1}.

4.15 4-(1,3-Dithian-2-ylidene)-1,3,4-triphenylbutan-1-one (**5h**)

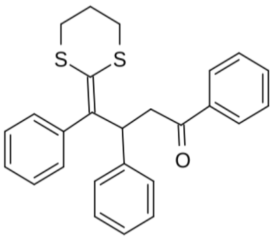

Name: 4-(1,3-Dithian-2-ylidene)-1,3,4-triphenylbutan-1-one; Formula: $C_{26}H_{24}OS_2$; CAS: - ; Smiles: O=C(c1ccccc1)CC(C(=C1SCCCS1)c1ccccc1)c1ccccc1; InCHI: ZUDKFLLVQMXJBO-UHFFFAOYSA-N; Molecular Mass: 416.5982; Exact Mass: 416.1269; EA: C, 74.96; H, 5.81; O, 3.84; S, 15.39.

According to General Procedure 2b: {A|**2a**} 2-benzyl-5,6-dihydro-4H-1,3-dithiin-1-ium tetrafluoroborate (0.183 g, 0.618 mmol, 1.00 equiv); {B|**3h**} (E)-1,3-diphenylprop-2-en-1-one (0.193 g, 0.927 mmol, 1.50 equiv); {C} acetonitrile (5.00 mL); Yield {D|**5h**} = 87% (0.224 g, 0.537 mmol).
The obtained crude product was purified via flash-chromatography on silica gel using cyclohexane/ethyl acetate 10:1. R_f = 0.63 (cyclohexane/ethyl acetate 4:1).
^{1}H NMR (500 MHz, $CDCl_3$, ppm) δ = 2.11–2.18 (m, 2H), 2.78–2.88 (m, 2H), 2.97–3.04 (m, 1H), 3.06–3.17 (m, 1H), 3.34–3.42 (m, 2H), 5.52 (t, *J* = 7.2 Hz, 1H), 6.68 (d, *J* = 7.0 Hz, 2H), 7.17–7.30 (m, 8H), 7.44–7.49 (m, 2H), 7.55–7.59 (m, 1H), 7.90–7.94 (m, 2H); ^{13}C NMR (125 MHz, $CDCl_3$, ppm) δ = 24.4, 29.6, 29.7, 40.8, 43.6, 126.4, 127.2, 127.8 (2C), 128.0 (4C), 128.1, 128.1 (2C), 128.5 (2C), 129.9 (2C), 132.9, 137.1, 138.6, 141.5, 142.1, 198.1; EI (*m/z*, 70 eV, 110 °C): 416 (4) $[M]^+$, 313 (33), 86 (64), 84 (100), 75 (42); HRMS–EI (*m*/*z*): $[M]^+$ calcd for $C_{26}H_{24}OS_2$,416.1263; found, 416.1264; IR (ATR, ṽ): 3024, 2914, 1681, 1596, 1579, 1492, 1447, 1416, 1360, 1298, 1241, 1202, 1179, 1072, 1028, 1000, 983, 909, 750, 733, 700, 615, 595, 568, 550 cm^{-1}.

4.16 6-(1,3-Dithiolan-2-ylidene)-5-methylheptan-3-one (**4i**)

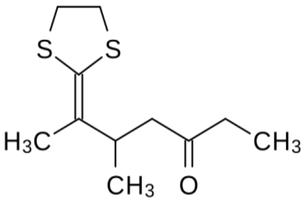

Name: 6-(1,3-dithiolan-2-ylidene)-5-methylheptan-3-one; Formula: $C_{11}H_{18}OS_2$; CAS: - ; Smiles: CCC(=O)CC(C(=C1SCCS1)C)C; InCHI: AGKKZIFNAVODRD-UHFFFAOYSA-N; Molecular Mass: 230.3900; Exact Mass: 230.0799; EA: C, 57.35; H, 7.87; O, 6.94; S, 27.84.

According to General Procedure 2a: {A|**1b**} 2-ethyl-4,5-dihydro-1,3-dithiol-1-ium tetrefluoroborate (0.150 g, 0.682 mmol, 1.00 equiv); {B|**3d**} (E)-hex-4-en-3-one (0.080 g, 0.818 mmol, 1.20 equiv); {C} acetonitrile (5.00 mL); Yield {D|**4i**} = 71% (0.111 g, 0.482 mmol).
The obtained crude product was purified via flash-chromatography on silica gel using cyclohexane/ethyl acetate 20:1 to 4:1.
^{1}H NMR (500 MHz, $CDCl_3$, ppm) δ = 1.00–1.04 (m, 6H), 1.71 (s, 3H), 2.34–2.50 (m, 4H), 3.09–3.18 (m, 1H), 3.32 (s, 4H); ^{13}C NMR (125 MHz, $CDCl_3$, ppm) δ = 7.7, 17.2, 18.2, 35.8,

37.2, 37.6, 38.0, 47.5, 125.0, 128.8, 210.4; EI (*m/z*, 70 eV, 40 °C): 230 (100) $[M]^+$, 159 (92), 57 (37); HRMS–EI (*m*/*z*): $[M]^+$ calcd for $C_{11}H_{18}OS_2$, 230.0794; found, 230.0795; IR (ATR, ṽ): 2964, 2925, 1707, 1601, 1454, 1410, 1368, 1277, 1109, 1025, 978, 923, 850, 685, 591 cm^{-1}.

4.17 6-(1,3-Dithian-2-ylidene)-5-methylheptan-3-one (**5i**)

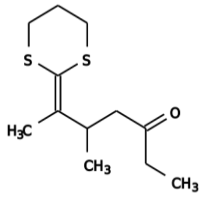

Name: 6-(1,3-dithian-2-ylidene)-5-methylheptan-3-one; Formula: $C_{12}H_{20}OS_2$; CAS: - ; Smiles: CCC(=O)CC(C(=C1SCCCS1)C)C; InCHI: UEBNFWJRYWYELY-UHFFFAOYSA-N; Molecular Mass: 244.4166; Exact Mass: 244.0956; EA: C, 58.97; H, 8.25; O, 6.55; S, 26.24.

According to General Procedure 2a: {A|**2b**} 2-ethyl-5,6-dihydro-4H-1,3-dithiin-1-ium tetrafluoroborate (0.150 g, 0.641 mmol, 1.00 equiv); {B|**3d**} (E)-hex-4-en-3-one (0.075 g, 0.769 mmol, 1.20 equiv); {C} acetonitrile (5.00 mL); Yield {D|**5i**} = 60% (0.093 g, 0.382 mmol).
The obtained crude product was purified via flash-chromatography on silica gel using cyclohexane/ethyl acetate 10:1. R_f = 0.58 (cyclohexane/ethyl acetate 2:1).
^{1}H NMR (500 MHz, $CDCl_3$, ppm) δ = 0.96 (d, *J* = 6.9, 3H), 1.01 (t, *J* = 7.3 Hz, 3H), 1.76 (s, 3H), 2.05–2.13 (m, 2H), 2.36–2.44 (m, 4H), 2.82–2.88 (m, 4H), 3.70–3.79 (m, 1H); ^{13}C NMR (125 MHz, $CDCl_3$, ppm) δ = 8.0, 15.2, 18.7, 25.1, 30.1, 30.4, 34.0, 36.0, 47.8, 120.6, 141.4, 210.5; EI (*m/z*, 70 eV, 30 °C): 244 (27) $[M]^+$, 178 (28), 173 (100), 163 (62), 131 (40), 121 (21), 93 (30), 57 (24); HRMS–EI (*m*/*z*): $[M]^+$ calcd for $C_{21}H_{20}OS_2$, 244.0950; found, 244.0951; IR (ATR, ṽ): 2965, 2932, 1709, 1455, 1414, 1374, 1275, 1110, 1024, 912, 817, 429 cm^{-1}.

4.18 4-(1-(1,3-Dithiolan-2-ylidene)ethyl)-5-methylhexan-2-one (**4j**)

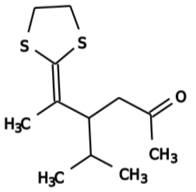

Name: 4-(1-(1,3-dithiolan-2-ylidene)ethyl)-5-methylhexan-2-one; Formula: $C_{12}H_{20}OS_2$; CAS: - ; Smiles: CC(C(C(=C1SCCS1)C)CC(=O)C)C; InCHI: LBBLNZCUATYWIS-UHFFFAOYSA-N; Molecular Mass: 244.4166; Exact Mass: 244.0956; EA: C, 58.97; H, 8.25; O, 6.55; S, 26.24.

According to General Procedure 2b: {A|**1b**} 2-ethyl-4,5-dihydro-1,3-dithiol-1-ium tetrafluoroborate (0.158 g, 0.717 mmol, 1.00 equiv); {B|**3e**} (E)-5-methylhex-3-en-2-one (0.121 g, 1.076 mmol, 1.50 equiv); {C} acetonitrile (5.00 mL); Yield {D|**4j**} = 52% (0.091 g, 0.374 mmol).

The obtained crude product was purified via flash-chromatography on silica gel using cyclohexane/ethyl acetate 10:1. R_f = 0.64 (cyclohexane/ethyl acetate 2:1).
^{1}H NMR (500 MHz, $CDCl_3$, ppm) δ = 0.81 (d, *J* = 6.7 Hz, 3H), 0.90 (d, *J* = 6.6 Hz, 3H), 1.50–1.60 (m, 1H), 1.64 (s, 3H), 2.09 (s, 3H), 2.34 (dd, *J* = 14.5 Hz, *J* = 9.8 Hz, 1H), 2.56 (dd, *J* = 14.5 Hz, *J* = 4.9 Hz, 1H), 2.67 (td, *J* = 9.7 Hz, *J* = 4.8 Hz, 1H), 3.21–3.30 (m, 4H); ^{13}C NMR (125 MHz, $CDCl_3$, ppm) δ = 17.5, 20.6, 20.7, 29.4, 31.5, 37.1, 37.4, 46.0, 51.0, 123.3, 131.1, 208.5; EI (*m/z*, 70 eV, 30 °C): 244 (37) $[M]^+$, 201 (100), 159 (38); HRMS–EI (*m/z*): [M] calcd for $C_{12}H_{20}OS_2$, 244.0950; found, 244.0952; IR (ATR, ṽ): 2956, 2924, 2869, 1705, 1601, 1466, 1420, 1355, 1278, 1229, 1161, 1112, 1030, 977, 904, 849, 731, 685, 566, 540, 465 cm^{-1}.

4.19 4-(1-(1,3-Dithiolan-2-ylidene)ethyl)octan-2-one (**4k**)

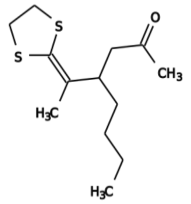

Name: 4-(1-(1,3-dithiolan-2-ylidene)ethyl)octan-2-one; Formula: $C_{13}H_{22}OS_2$; CAS: - ; Smiles: CCCCC(C(=C1SCCS1)C)CC(=O)C; InChI: OOWHPIJVOYHULD-UHFFFAOYSA-N; Molecular Mass: 258.4432; Exact Mass: 258.1112; EA: C, 60.42; H, 8.58; O, 6.19; S, 24.81.

According to General Procedure 2a: {A|**1b**} 2-ethyl-4,5-dihydro-1,3-dithiol-1-ium tetrafluoroborate (0.148 g, 0.673 mmol, 1.00 equiv); {B|**3f**} (E)-oct-3-en-2-one (0.102 g, 0.808 mmol, 1.20 equiv); {C} acetonitrile (5.00 mL); Yield {D|**4k**} = 66% (0.115 g, 0.445 mmol).
The obtained crude product was purified via flash-chromatography on silica gel using cyclohexane/ethyl acetate 10:1. R_f = 0.71 (cyclohexane/ethyl acetate 10:1).
^{1}H NMR (500 MHz, $CDCl_3$, ppm) δ = 0.83 (t, *J* = 7.2 Hz, 3H), 1.13–1.34 (m, 6H), 1.63 (s, 3H), 2.09 (s, 3H), 2.38 (d, *J* = 7.2 Hz, 2H), 2.94–3.02 (m, 1H), 3.26–3.31 (m, 4H); ^{13}C NMR (125 MHz, $CDCl_3$, ppm) δ = 14.1, 17.1, 22.8, 29.5, 30.0, 33.2, 37.4, 37.7, 43.8, 48.1, 123.5, 130.6, 208.3; EI (*m/z*, 70 eV, 70 °C): 258 (46) $[M]^+$, 201 (100), 159 (25), 145 (21); HRMS–EI (*m/z*): $[M]^+$ calcd for $C_{13}H_{22}OS_2$, 258.1107; found, 258.1108; IR (ATR, ṽ): 2953, 2923, 2855, 1708, 1601, 1420, 1355, 1277, 1159, 1105, 976, 907, 850, 729, 685, 566 cm^{-1}.

4.20 5-(1,3-Dithiolan-2-ylidene)-4-phenylhexan-2-one (**4l**)

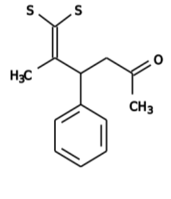

Name: 5-(1,3-dithiolan-2-ylidene)-4-phenylhexan-2-one; Formula: $C_{15}H_{18}OS_2$; CAS: - ; Smiles: CC(=C1SCCS1)C(c1ccccc1)CC(=O)C; InChI: VWZGXEMKFJLYLX-UHFFFAOYSA-N; Molecular Mass: 278.4328; Exact Mass: 278.0799; EA: C, 64.71; H, 6.52; O, 5.75; S, 23.03.

According to General Procedure 2b: {A|**1b**} 2-ethyl-4,5-dihydro-1,3-dithiol-1-ium tetrafluoroborate (0.168 g, 0.763 mmol, 1.00 equiv); {B|**3g**} (E)-4-phenylbut-3-en-2-one (0.168 g, 1.148 mmol, 1.50 equiv); {C} acetonitrile (5.00 mL); Yield {D|**4l**} = 73% (0.154 g, 0.555 mmol).
The obtained crude product was purified via flash-chromatography on silica gel using cyclohexane/ethyl acetate 10:1 + 1% triethylamine.
^{1}H NMR (500 MHz, $CDCl_3$, ppm) δ = 1.64–1.68 (m, 3H), 2.19 (s, 3H), 2.87 (dd, *J* = 15.6, *J* = 7.8 Hz, 1H), 3.01 (dd, *J* = 15.6, *J* = 7.6 Hz, 1H), 3.35–3.43 (m, 4H), 4.48 (t, *J* = 7.6 Hz, 1H), 7.18–7.24 (m, 3H), 7.27–7.31 (m, 2H); ^{13}C NMR (125 MHz, $CDCl_3$, ppm) δ = 18.4, 30.2, 37.8, 38.1, 46.0, 48.2, 123.6, 126.8, 127.4 (2C), 128.7 (2C), 131.5, 141.7, 207.4; EI (*m/z*, 70 eV, 60 °C): 278 (82) $[M]^+$, 221 (100), 198 (32), 170 (86), 161 (50), 131 (24), 58 (28); HRMS–EI (*m*/*z*): $[M]^+$ calcd for $C_{15}H_{18}O_2S_2$, 278.0794; found, 278.0796; IR (ATR, ṽ): 3024, 2921, 2849, 1707, 1599, 1493, 1448, 1418, 1354, 1278, 1249, 1157, 1085, 1029, 977, 912, 848, 773, 743, 699, 648, 567, 549, 494, 389 cm^{-1}.

4.21 6-(1,3-Dithiolan-2-ylidene)octan-3-one (**4m**)

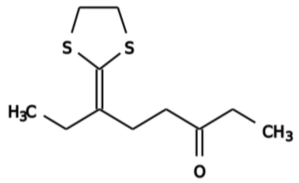

Name: 6-(1,3-dithiolan-2-ylidene)octan-3-one; Formula: $C_{11}H_{18}OS_2$; CAS: - ; Smiles: CCC(=C1SCCS1)CCC(=O)CC; InCHI: YDJFXLVHPNACDY-UHFFFAOYSA-N; Molecular Mass: 230.3900; Exact Mass: 230.0799; EA: C, 57.35; H, 7.87; O, 6.94; S, 27.84.

According to General Procedure 2a: {A|**1c**} 2-propyl-4,5-dihydro-1,3-dithiol-1-ium tetrafluoroborate (0.250 g, 1.068 mmol, 1.00 equiv); {B|**3b**} pent-1-en-3-one (0.108 g, 1.282 mmol, 1.20 equiv); {C} acetonitrile (5.00 mL); Yield {D|**4m**} = 55% (0.135 g, 0.585 mmol).
The obtained crude product was purified via flash-chromatography on silica gel using cyclohexane/ethyl acetate 10:1. R_f = 0.58 (cyclohexane/ethyl acetate 20:1 + 1% NEt_3).
^{1}H NMR (400 MHz, $CDCl_3$, ppm) δ = 1.01 (t, *J* = 7.5 Hz, 3H), 1.06 (t, *J* = 7.3 Hz, 3H), 2.16 (q, *J* = 7.5 Hz, 9H), 2.40–2.47 (m, 4H), 2.49–2.56 (m, 2H), 3.33 (s, 4H); ^{13}C NMR (100 MHz, $CDCl_3$, ppm) δ = 7.8, 12.0, 29.8, 30.2, 35.8, 37.6, 37.7, 40.0, 127.5, 129.3, 211.1; EI (*m/z*, 70 eV, 20 °C): 230.2 (65)$[M]^+$, 181 (31), 159 (100); HRMS–EI (*m*/*z*): [M] calcd for $C_{11}H_{18}OS_2$, 230.0799; found, 230.0800; IR (ATR, ṽ) = 2963, 2928, 1710, 1600, 1457, 1419, 1359, 1278, 1147, 1111, 1045, 987, 913, 848, 827, 685 cm^{-1}.

4.22 6-(1,3-Dithiolan-2-ylidene)-5-methyloctan-3-one (**4n**)

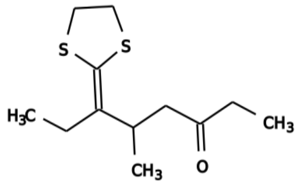

Name: 6-(1,3-dithiolan-2-ylidene)-5-methyloctan-3-one; Formula: $C_{12}H_{20}OS_2$; CAS: - ; Smiles: CCC(=C1SCCS1)C(CC(=O)CC)C; InCHI: HNEMFLNWZMGKBV-

UHFFFAOYSA-N; Molecular Mass: 244.4166; Exact Mass: 244.0956; EA: C, 58.97; H, 8.25; O, 6.55; S, 26.24.

According to General Procedure 2a: {A|**1c**} 2-propyl-4,5-dihydro-1,3-dithiol-1-ium tetrafluoroborate (0.176 g, 0.752 mmol, 1.00 equiv); {B|**3d**} (E)-hex-4-en-3-one (0.088 g, 0.901 mmol, 1.20 equiv); {C} acetonitrile (5.00 mL); Yield {D|**4n**} = 77% (0.141 g, 0.576 mmol).
The obtained crude product was purified via flash-chromatography on silica gel using cyclohexane/ethyl acetate 10:1 + 1% triethylamine. R_f = 0.61 (cyclohexane/ethyl acetate 2:1). ^{1}H NMR (500 MHz, $CDCl_3$, ppm) δ = 0.95–1.02 (m, 9H), 2.05–2.14 (m, 2H), 2.34–2.40 (m, 3H), 2.49 (dd, *J* = 15.6 Hz, *J* = 5.9 Hz, 1H), 3.03–3.12 (m, 1H), 3.25–3.29 (m, 4H); ^{13}C NMR (125 MHz, $CDCl_3$, ppm) δ = 7.6, 13.0, 18.4, 25.9, 35.8, 37.1, 37.3, 37.8, 47.6, 129.6, 131.3, 210.2; EI (*m/z,* 70 eV): 244 (34) $[M]^+$, 173 (100), 163 (28); HRMS–EI (*m*/*z*): $[M]^+$ calcd for $C_{12}H_{20}OS_2$, 244.0950; found, 244.0950; IR (ATR, ṽ): 2926, 1707, 1589, 1504, 1456, 1421, 1371, 1279, 1257, 1142, 990, 922, 872, 848, 785, 740, 711, 686, 573, 489 cm^{-1}.

4.23 5-(1,3-Dithiolan-2-ylidene)-4-isopropylheptan-2-one (**4o**)

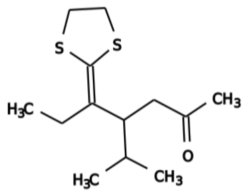

Name: 5-(1,3-dithiolan-2-ylidene)-4-isopropylheptan-2-one; Formula: $C_{13}H_{22}OS_2$; CAS: - ; Smiles: CCC(=C1SCCS1)C(C(C)C)CC(=O)C; InChI: FYEONQBYGJVCGM-UHFFFAOYSA-N; Molecular Mass: 258.4432; Exact Mass: 258.1112; EA: C, 60.42; H, 8.58; O, 6.19; S, 24.81.

According to General Procedure 2b: {A|**1c**} 2-propyl-4,5-dihydro-1,3-dithiol-1-ium tetrafluoroborate (0.150 g, 0.641 mmol, 1.00 equiv); {B|**3e**} (E)-5-methylhex-3-en-2-one (0.108 g, 0.961 mmol, 1.50 equiv); {C} acetonitrile (5.00 mL); Yield {D|**4o**} = 44% (0.073 g, 0.284 mmol).
The obtained crude product was purified via flash-chromatography on silica gel using cyclohexane/ethyl acetate 10:1 +1% triethylamine. R_f = 0.68 (cyclohexane/ethyl acetate 2:1). ^{1}H NMR (500 MHz, $CDCl_3$, ppm) δ = 0.87 (d, *J* = 6.6 Hz, 3H), 0.90 (d, *J* = 6.6 Hz, 3H), 1.03 (t, *J* = 7.6 Hz, 3H), 1.59–1.69 (m, 1H), 2.11 (s, 3H), 2.04–2.11 (m, 2H), 2.40–2.47 (m, 1H), 2.57–2.63 (m, 1H), 2.72 (td, *J* = 9.5 Hz, *J* = 5.0 Hz, 1H), 3.23–3.31 (m, 4H); ^{13}C NMR (125 MHz, $CDCl_3$, ppm) δ = 12.6, 20.9, 21.1, 26.1, 29.6, 31.6, 37.2, 37.2, 46.3, 51.2, 129.9, 131.8, 208.4; EI (*m/z*, 70 eV, 30 °C): 258 (29) $[M]^+$, 215 (100), 173 (47); HRMS–EI (*m*/*z*): $[M]^+$ calcd for $C_{13}H_{22}OS_2$, 258.1107; found, 258.1106; IR (ATR, ṽ): 2959, 2927, 2870, 1705, 1588, 1461, 1420, 1384, 1355, 1278, 1257, 1229, 1160, 1113, 1056, 1021, 978, 895, 848, 807, 685, 587, 543, 504, 431 cm^{-1}.

4.24 4-(1-(1,3-Dithiolan-2-ylidene)propyl)octan-2-one (**4p**)

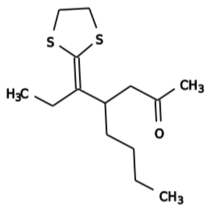

Name: 4-(1-(1,3-Dithiolan-2-ylidene)propyl)octan-2-one; Formula: $C_{14}H_{24}OS_2$; CAS: - ; Smiles: CCCCC(C(=C1SCCS1)CC)CC(=O)C; InCHI: KDAUXNCFMLRQRP-UHFFFAOYSA-N; Molecular Mass: 272.4698; Exact Mass: 272.1269; EA: C, 61.71; H, 8.88; O, 5.87; S, 23.54.

According to General Procedure 2a: {A|**1c**} 2-propyl-4,5-dihydro-1,3-dithiol-1-ium tetrafluoroborate (0.155 g, 0.662 mmol, 1.00 equiv); {B|**3f**} (E)-oct-3-en-2-one (0.125 g, 0.993 mmol, 1.50 equiv); {C} acetonitrile (5.00 mL); Yield {D|**4p**} = 79% (0.143 g, 0.524 mmol).
The obtained crude product was purified via flash-chromatography on silica gel using cyclohexane/ethyl acetate 10:1 + 1% triethylamine. R_f = 0.71 (cyclohexane/ethyl acetate 2:1). ^{1}H NMR (500 MHz, $CDCl_3$, ppm) δ = 0.82 (t, *J* = 7.1 Hz, 3H), 0.99 (t, *J* = 7.6 Hz, 3H), 1.13–1.37 (m, 6H), 1.87–2.09 (m, 2H), 2.09 (s, 3H), 2.39–2.49 (m, 2H), 2.94–3.02 (m, 1H), 3.22–3.29 (m, 4H); ^{13}C NMR (125 MHz, $CDCl_3$, ppm) δ = 12.6, 13.6, 22.3, 25.2, 29.2, 29.5, 32.9, 36.9, 36.9, 43.5, 47.9, 129.5, 130.8, 207.7; EI (*m/z*, 70 eV, 100 °C): 272 (10) $[M]^+$, 173 (60), 159 (100), 145 (57); HRMS–EI (*m*/*z*): $[M]^+$ calcd for $C_{14}H_{24}O_3S_2$, 272.1263; found, 272.1263; IR (ATR, ṽ): 2956, 2924, 2855, 1708, 1589, 1458, 1420, 1355, 1277, 1242, 1158, 1107, 1057, 977, 895, 848, 729, 685, 533 cm^{-1}.

4.25 4-(1,3-Dithiolan-2-ylidene)-1,3-diphenylhexan-1-one (**4q**)

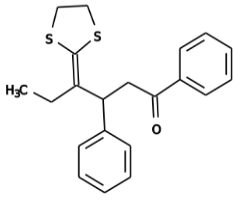

Name: 4-(1,3-dithiolan-2-ylidene)-1,3-diphenylhexan-1-one; Formula: $C_{21}H_{22}OS_2$; CAS: - ; Smiles: CCC(=C1SCCS1)C(c1ccccc1)CC(=O)c1ccccc1; InCHI: FFNUZHPVLBYLFF-UHFFFAOYSA-N; Molecular Mass: 354.5288; Exact Mass: 354.1112; EA: C, 71.14; H, 6.25; O, 4.51; S, 18.09.

According to General Procedure 2b: {A|**1c**} 2-propyl-4,5-dihydro-1,3-dithiol-1-ium tetrafluoroborate (0.156 g, 0.666 mmol, 1.00 equiv); {B|**3h**} (E)-1,3-diphenylprop-2-en-1-one (0.209 g, 1.002 mmol, 1.50 equiv); {C} acetonitrile (5.00 mL); Yield {D|**4q**} = 65% (0.154 g, 0.433 mmol).
The obtained crude product was purified via flash-chromatography on silica gel using cyclohexane/ethyl acetate 10:1 + 1% triethylamine. R_f = 0.73 (cyclohexane/ethyl acetate 2:1). ^{1}H NMR (500 MHz, $CDCl_3$, ppm) δ = 0.80 (t, *J* = 7.6 Hz, 3H), 2.11–2.19 (m, 2H), 3.34–3.40 (m, 4H), 3.48 (dd, *J* = 16.6 Hz, *J* = 6.4 Hz, 1H), 3.70 (dd, *J* = 16.6 Hz, *J* = 8.2 Hz, 1H), 4.72 (dd, *J* = 8.2 Hz, 6.5 Hz, 1H), 7.19–7.24 (m, 1H), 7.27–7.32 (m, 4H), 7.44–7.52 (m, 2H),

7.57–7.61 (m, 1H), 7.99–8.04 (m, 2H); ^{13}C NMR (125 MHz, $CDCl_3$, ppm) δ = 12.7, 26.9, 37.5, 37.7, 40.5, 47.8, 126.4, 127.4 (2C), 128.1 (2C), 128.3 (2C), 128.5 (2C), 130.1, 131.6, 132.9, 137.0, 141.8, 198.1; EI (*m/z*, 70 eV, 90 °C): 354 (10) $[M]^+$, 313 (72), 147 (34), 105 (38), 84 (100), 75 (96); HRMS–EI (*m*/*z*): $[M]^+$ calcd for $C_{21}H_{22}OS_2$, 354.1107; found, 354.1107; IR (ATR, ṽ): 3057, 3024, 2961, 2924, 2869, 1681, 1595, 1579, 1493, 1447, 1419, 1359, 1263, 1214, 1179, 1058, 1029, 1001, 977, 910, 845, 770, 751, 739, 690, 654, 617, 588, 549 cm^{-1}.

4.26 Methyl 2-(1,3-dithian-2-ylidene)-3-methyl-5-oxoheptanoate (**5r**)

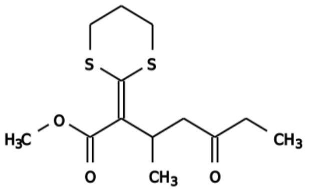

Name: Methyl 2-(1,3-dithian-2-ylidene)-3-methyl-5-oxoheptanoate; Formula: $C_{13}H_{20}O_3S_2$; CAS: - ; Smiles: CCC(=O)CC(C(=C1SCCCS1)C(=O)OC)C; InCHI: SLOZNFFLELWBBW-UHFFFAOYSA-N; Molecular Mass: 288.4261; Exact Mass: 288.0854; EA: C, 54.13; H, 6.99; O, 16.64; S, 22.23.

According to General Procedure 2a: {A|**2d**} 2-(2-methoxy-2-oxoethyl)-5,6-dihydro-4H-1,3-dithiin-1-ium tetrafluoroborate (0.150 g, 0.539 mmol, 1.00 equiv); {B|**3d**} (E)-hex-4-en-3-one (0.064 g, 0.647 mmol, 1.20 equiv); {C} acetonitrile (5.00 mL); Yield {D|**5r**} = 46% (0.071 g, 0.246 mmol).
The obtained crude product was purified via flash-chromatography on silica gel using cyclohexane/ethyl acetate 10:1. R_f = 0.38 (cyclohexane/ethyl acetate 2:1).
^{1}H NMR (500 MHz, $CDCl_3$, ppm) δ = 1.02 (t, *J* = 7.3 Hz, 3H), 1.07 (d, *J* = 6.9 Hz, 3H), 2.10 (quin, *J* = 6.9 Hz, 2H), 2.37 (qd, *J* = 7.3 Hz, *J* = 3.0 Hz, 2H), 2.58–2.69 (m, 2H), 2.82–2.91 (m, 2H), 2.93–3.02 (m, 2H), 3.65–3.72 (m, 1H), 3.74 (s, 3H); ^{13}C NMR (125 MHz, $CDCl_3$, ppm) δ = 7.7, 18.6, 23.5, 29.0, 29.0, 31.6, 36.2, 47.5, 51.1, 130.7, 147.8, 166.3, 210.2; EI (*m/z*, 70 eV, 120 °C): 288 (78) $[M]^+$, 257 (31), 228 (55), 217 (89), 199 (80), 185 (100), 181 (64), 131 (47); HRMS–EI (*m*/*z*): $[M]^+$ calcd for $C_{13}H_{20}O_3S_2$, 288.0848; found, 288.0849; IR (ATR, ṽ): 2968, 2933, 2874, 1706, 1504, 1430, 1372, 1246, 1189, 1145, 1110, 988, 921, 782, 715 cm^{-1}.

4.27 3-((1,3-Dithiolan-2-ylidene)(phenyl)methyl)cyclopentan-1-one (**9a**)

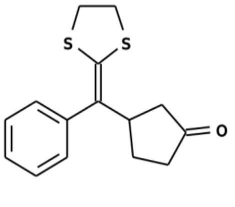

Name: 3-((1,3-dithiolan-2-ylidene)(phenyl)methyl)cyclopentan-1-one; Formula: $C_{15}H_{16}OS_2$; CAS: - ; Smiles: O=C1CCC(C1)C(=C1SCCS1)c1ccccc1; InCHI: LOEMJXFENGAIRJ-UHFFFAOYSA-N; Molecular Mass: 276.4169; Exact Mass: 276.0643; EA: C, 65.18; H, 5.83; O, 5.79; S, 23.2.

According to General Procedure 2a: {A|**1a**} 2-benzyl-4,5-dihydro-1,3-dithiol-1-ium tetrafluoroborate (0.150 g, 0.532 mmol, 1.00 equiv); {B|**8a**} cyclopent-2-en-1-one (0.052 g, 0.638 mmol, 1.20 equiv); {C} acetonitrile (5.00 mL); Yield {D|**9a**} = 93% (0.137 g, 0.494 mmol).
The obtained crude product was purified via flash-chromatography on silica gel using cyclohexane/ethyl acetate 10:1. R_f = 0.36 (cyclohexane/ethyl acetate 2:1).
^{1}H NMR (500 MHz, $CDCl_3$, ppm) δ = 1.62–1.72 (m, 1H), 1.95 (dd, *J* = 18.1 Hz, *J* = 11.8 Hz, 1H), 2.09–2.30 (m, 3H), 2.41 (dd, *J* = 18.1 Hz, *J* = 8.3 Hz, 1H), 3.23–3.26 (m, 2H), 3.40–3.49 (m, 3H), 7.09–7.12 (m, 2H), 7.29–7.33 (m, 1H), 7.35–7.40 (m, 2H); ^{13}C NMR (125 MHz, $CDCl_3$, ppm) δ = 27.5, 37.2, 37.9, 38.0, 42.8, 44.8, 127.4, 128.0, 128.4 (2C), 129.5 (2C), 135.9, 139.9, 217.7; EI (*m/z*, 70 eV, 160 °C): 276 (57) $[M]^+$, 191 (100), 171 (26), 115 (30); HRMS–EI (*m*/*z*): $[M]^+$ calcd for $C_{15}H_{16}OS_2$, 276.0637; found, 276.0636; IR (ATR, ṽ): 2924, 1733, 1596, 1580, 1485, 1436, 1399, 1279, 1243, 1166, 1152, 1138, 1080, 1024, 980, 907, 845, 792. 767, 732, 704, 665, 604, 570, 484, 432, 400 cm^{-1}.

4.28 3-((1,3-Dithian-2-ylidene)(phenyl)methyl)cyclopentan-1-one (**10a**)

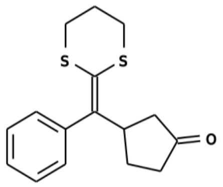

Name: 3-((1,3-dithian-2-ylidene)(phenyl)methyl)cyclopentan-1-one; Formula: $C_{16}H_{18}OS_2$; CAS: - ; Smiles: O=C1CCC(C1)C(=C1SCCCS1)c1ccccc1; InCHI: RUNYTOSEZSUNGD-UHFFFAOYSA-N; Molecular Mass: 290.4435; Exact Mass: 290.0799; EA: C, 66.16; H, 6.25; O, 5.51; S, 22.08.

According to General Procedure 2a: {A|**2a**} 2-benzyl-5,6-dihydro-4H-1,3-dithiin-1-ium tetrafluoroborate (0.150 g, 0.506 mmol, 1.00 equiv); {B|**8a**} cyclopent-2-en-1-one (0.050 g, 0.608 mmol, 1.20 equiv); {C} acetonitrile (5.00 mL); Yield {D|**10a**} = 92% (0.135 g, 0.465 mmol).
The obtained crude product was purified via flash-chromatography on silica gel using cyclohexane/ethyl acetate 10:1. R_f = 0.47 (cyclohexane/ethyl acetate 2:1).
^{1}H NMR (500 MHz, $CDCl_3$, ppm) δ = 1.57–1.70 (m, 1H), 1.90 (dd, *J* = 18.0 Hz, *J* = 12.2 Hz, 1H), 2.07–2.18 (m, 5H), 2.34 (dd, *J* = 18.0 Hz, *J* = 7.3 Hz, 1H), 2.77–2.83 (m, 2H), 2.92–3.04 (m, 2H), 3.92 (tdd, *J* = 12.0 Hz, *J* = 7.0 Hz, *J* = 5.5 Hz, 1H), 6.99–7.05 (m, 2H), 7.29–7.34 (m, 1H), 7.35–7.40 (m, 2H); ^{13}C NMR (125 MHz, $CDCl_3$, ppm) δ = 24.5, 27.8, 29.5, 29.6, 38.2, 41.1, 43.1, 127.6, 127.7, 128.5 (2C), 129.7 (2C), 138.4, 141.0, 218.0; EI (*m/z*, 70 eV, 100 °C): 290 (100) $[M]^+$, 215 (25), 187 (74), 173 (41), 115 (39); HRMS–EI (*m*/*z*): $[M]^+$ calcd for $C_{16}H_{18}OS_2$, 290.0794; found, 290.0792; IR (ATR, ṽ): 2911, 1727, 1485, 1419, 1395, 1276, 1233, 1138, 1070, 1027, 984, 911, 858, 767, 766, 725, 708, 605, 575, 484, 417 cm^{-1}.

4.29 3-((1,3-Dithiolan-2-ylidene)(phenyl)methyl)cyclohexan-1-one (**9b**)

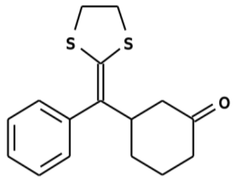

Name: 3-((1,3-dithiolan-2-ylidene)(phenyl)methyl)cyclohexan-1-one; Formula: $C_{16}H_{18}OS_2$; CAS: - ; Smiles: O=C1CCCC(C1)C(=C1SCCS1)c1ccccc1; InCHI: XDXILYAJVPWYNR-UHFFFAOYSA-N; Molecular Mass: 290.4435; Exact Mass: 290.0799; EA: C, 66.16; H, 6.25; O, 5.51; S, 22.08.

According to General Procedure 2a: {A|**1a**} 2-benzyl-4,5-dihydro-1,3-dithiol-1-ium tetrafluoroborate (0.150 g, 0.532 mmol, 1.00 equiv); {B|**8b**} cyclohex-2-en-1-one (0.061 g, 0.638 mmol, 1.20 equiv); {C} acetonitrile (5.00 mL); Yield {D|**9b**} = 94% (0.145 g, 0.501 mmol).
The obtained crude product was purified via flash-chromatography on silica gel using cyclohexane/ethyl acetate 10:1. R_f = 0.63 (cyclohexane/ethyl acetate 2:1).
^{1}H NMR (500 MHz, $CDCl_3$, ppm) δ = 1.42–1.51 (m, 1H), 1.65–1.77 (m, 1H), 1.97–2.16 (m, 4H), 2.29–2.35 (m, 1H), 2.44–2.50 (m, 1H), 3.06 (tt, *J* = 12.6 Hz, *J* = 3.6 Hz, 1H), 3.24 (dd, *J* = 6.6 Hz, *J* = 5.1 Hz, 2H), 3.37–3.44 (m, 2H), 7.10–7.15 (m, 2H), 7.31–7.41 (m, 3H); ^{13}C NMR (125 MHz, $CDCl_3$, ppm) δ = 25.0, 29.7, 37.2, 37.9, 40.8, 46.2, 46.9, 127.4, 128.3 (2C), 129.4 (2C), 129.5, 135.4, 140.1, 210.6; EI (*m/z*, 70 eV, 120 °C): 290 (100) $[M]^+$, 233 (15), 192 (25); HRMS–EI (*m/z*): $[M]^+$ calcd for $C_{16}H_{18}OS_2$, 290.0794; found, 290.0795; IR (ATR, ṽ): 2925, 2860, 1704, 1598, 1489, 1439, 1419, 1343, 1313, 1278, 1252, 1221, 1181, 1149, 1070, 1026, 964, 910, 867, 849, 763, 729, 703, 646, 565, 508, 420, 392 cm^{-1}.

4.30 3-((1,3-Dithian-2-ylidene)(phenyl)methyl)cyclohexan-1-one (**10b**)

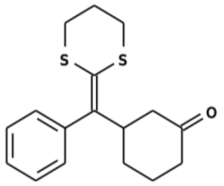

Name: 3-((1,3-dithian-2-ylidene)(phenyl)methyl)cyclohexan-1-one; Formula: $C_{17}H_{20}OS_2$; CAS: - ; Smiles: O=C1CCCC(C1)C(=C1SCCCS1)c1ccccc1; InCHI: DBMRDQGRCWFNNO-UHFFFAOYSA-N; Molecular Mass: 304.4701; Exact Mass: 304.0956; EA: C, 67.06; H, 6.62; O, 5.25; S, 21.06.

According to General Procedure 2a: {A|**2a**} 2-benzyl-5,6-dihydro-4H-1,3-dithiin-1-ium tetrafluoroborate (0.150 g, 0.506 mmol, 1.00 equiv); {B|**8b**} cyclohex-2-en-1-one (0.058 g, 0.608 mmol, 1.20 equiv); {C} acetonitrile (5.00 mL); Yield {D|**10b**} = 52% (0.080 g, 0.262 mmol).
The obtained crude product was purified via flash-chromatography on silica gel using cyclohexane/ethyl acetate 10:1. The reaction was repeated at 60 °C and was stirred for 15 h. The product could be obtained in 81% yield. R_f = 0.65 (cyclohexane/ethyl acetate 2:1).

^{1}H NMR (500 MHz, $CDCl_3$, ppm) δ = 1.37–1.47 (m, 1H), 1.64–1.76 (m, 1H), 1.83–1.91 (m, 1H), 1.99–2.12 (m, 5H), 2.27–2.39 (m, 2H), 2.74–2.84 (m, 2H), 2.89–3.01 (m, 2H), 3.56 (tt, *J* = 12.7 Hz, *J* = 3.6 Hz, 1H), 7.03 (d, *J* = 7.02 Hz, 2H), 7.31–7.41 (m, 3H); ^{13}C NMR (125 MHz, $CDCl_3$, ppm) δ = 24.4, 25.3, 29.4, 29.5, 30.0, 41.0, 42.9, 46.4, 127.2, 127.5, 128.4 (2C), 129.5 (2C), 138.6, 142.2, 210.9; EI (*m/z*, 70 eV, 110 °C): 304 (100) $[M]^+$, 247 (14), 229 (20), 173 (18); HRMS ($C_{17}H_{20}OS_2^+$): Calcd 304.0950. Found. 304.0949. HRMS–EI (*m*/*z*): $[M]^+$ calcd for $C_{17}H_{20}OS_2$, 304.0950; found, 304.0949; IR (ATR, ṽ): 2935, 1697, 1564, 1488, 1439, 1419, 1317, 1281, 1245, 1226, 1181, 1072, 1024, 960, 937, 864, 766, 703, 669, 635, 562, 507, 459, 420 cm^{-1}.

4.31 3-(1-(1,3-Dithiolan-2-ylidene)ethyl)cyclopentan-1-one (**9c**)

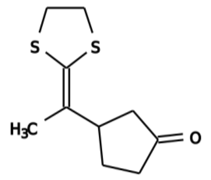

Name: 3-(1-(1,3-dithiolan-2-ylidene)ethyl)cyclopentan-1-one; Formula: $C_{10}H_{14}OS_2$; CAS: - ; Smiles: O=C1CCC(C1)C(=C1SCCS1)C; InCHI: QPWUQJGOQDXGSQ-UHFFFAOYSA-N; Molecular Mass: 214.3476; Exact Mass: 214.0486; EA: C, 56.03; H, 6.58; O, 7.46; S, 29.92.

According to General Procedure 2a: {A|**1b**} 2-ethyl-4,5-dihydro-1,3-dithiol-1-ium tetrafluoroborate (0.300 g, 1.363 mmol, 1.00 equiv); {B|**8a**} cyclopent-2-en-1-one (0.134 g, 1.636 mmol, 1.20 equiv); {C} acetonitrile (5.00 mL); Yield {D|**9c**} = 59% (0.173 g, 0.807 mmol).
The reaction mixture was stirred at –40 °C for 4 h and the obtained crude product was purified via flash-chromatography on silica gel using cyclohexane/ethyl acetate 10:1. According to the adapted protocol, the product was obtained in 59% yield. The reaction was repeated at room temperature for 1 h which resulted in the isolation of the product {D|**9c**} in 34% yield. R_f = 0.43 (cyclohexane/ethyl acetate 20:1).
^{1}H NMR (500 MHz, $CDCl_3$, ppm) δ = 1.79 (s, 3H), 1.76–1.85 (m, 1H), 2.05–2.25 (m, 3H), 2.31–2.39 (m, 2H), 3.20–3.29 (m, 1H), 3.34–3.39 (m, 4H); ^{13}C NMR (125 MHz, $CDCl_3$, ppm) δ = 17.6, 27.0, 37.4, 37.6, 38.5, 41.9, 44.9, 121.6, 130.7, 218.5; EI (*m/z*, 70 eV, 80 °C): 214 (100) $[M]^+$, 186 (13); HRMS–EI (*m*/*z*): $[M]^+$ calcd for $C_{10}H_{14}OS_2$, 214.0483; found, 214.0481; IR (ATR, ṽ): 3433, 2923, 1734, 1672, 1601, 1401, 1278, 1242, 1133, 976, 895, 850, 683, 568, 483 cm^{-1}.

4.32 3-(1-(1,3-Dithian-2-ylidene)ethyl)cyclopentan-1-one (**10c**)

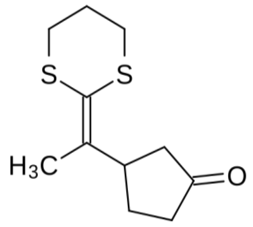

Name: 3-(1-(1,3-Dithian-2-ylidene)ethyl)cyclopentan-1-one; Formula: $C_{11}H_{16}OS_2$; CAS: - ; Smiles: O=C1CCC(C1)C(=C1SCCCS1)C; InCHI: WQJQUKJBHBQXMD-UHFFFAOYSA-

N; Molecular Mass: 228.3741; Exact Mass: 228.0643; EA: C, 57.85; H, 7.06; O, 7.01; S, 28.08.

According to General Procedure 6: {A|**2b**} 2-ethyl-5,6-dihydro-4H-1,3-dithiin-1-ium tetrafluoroborate (0.204 g, 0.871 mmol, 1.00 equiv); {B|**8a**} cyclopent-2-en-1-one (0.086 g, 1.046 mmol, 1.20 equiv); {C} acetonitrile (5.00 mL); Yield {D|**10c**} = 26% (0.052 g, 0.226 mmol).
The reaction was stirred at room temperature for 1 h. The obtained crude product was purified via flash-chromatography on silica gel using cyclohexane/ethyl acetate 100:0 to 95:5. R_f = 0.42 (cyclohexane/ethyl acetate 4:1).
^{1}H NMR (400 MHz, $CDCl_3$, ppm) δ = 1.71–1.83 (m, 1H), 1.84–1.86 (m, 3H), 1.98–2.39 (m, 8H), 2.84–2.96 (m, 4H), 3.78–3.90 (m, 1H); ^{13}C NMR (100 MHz, $CDCl_3$, ppm) δ = 15.3, 24.8, 27.2, 29.7, 30.0, 38.5, 40.9, 42.1, 122.4, 137.3, 218.4; EI (*m/z*, 70 eV, 50 °C): 228 (100) $[M]^+$, 181 (14), 15 (11), 153 (16), 131 (11), 130 (13), 125 (44), 111 (19), 98 (12), 97 (14), 86 (43), 84 (63), 69 (21); HRMS–EI (*m*/*z*): [M] calcd for $C_{11}H_{16}OS_2$, 228.0643; found, 228.0644; IR (ATR, ṽ): 2906, 1736, 1401, 1373, 1299, 1275, 1242, 1167, 1132, 1025, 976, 898, 816, 730, 550, 484 cm^{-1}.

4.33 3-(1-(1,3-Dithiolan-2-ylidene)ethyl)cyclohexan-1-one (**9d**)

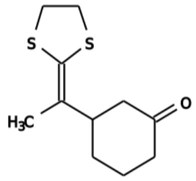

Name: 3-(1-(1,3-dithiolan-2-ylidene)ethyl)cyclohexan-1-one; Formula: $C_{11}H_{16}OS_2$; CAS: - ; Smiles: O=C1CCCC(C1)C(=C1SCCS1)C; InCHI: ZCSYABZYYYQJDH-UHFFFAOYSA-N; Molecular Mass: 228.3741; Exact Mass: 228.0643; EA: C, 57.85; H, 7.06; O, 7.01; S, 28.08.

The dithiolanylium tetrafluoroborate {A|**1b**} (1.00 equiv) was weighed into a dried Schlenk flask, evaporated for 10 min, then flushed with nitrogen. The tetrafluoroborate was dissolved by addition of dry acetonitrile {C} and the mixture was cooled to –40 °C. Cyclohexenone {B|**8b**} was disssolved in 1 mL of acetonitrile and was added in one portion. After 4 h, saturated $NaHCO_3$(aq) was added and the aqueous phase was extracted with ethyl acetate twice, the combined organic layers were washed with water and brine then dried over sodium sulfate.
{A|**1b**} 2-ethyl-4,5-dihydro-1,3-dithiol-1-ium tetrafluoroborate (0.302 g, 1.372 mmol, 1.00 equiv); {B|**8b**} cyclohex-2-en-1-one (0.158 g, 1.647 mmol, 1.20 equiv); {C} acetonitrile (1.00 mL); {D} acetonitrile (10.00 mL); Yield {E|**9d**} = 42% (0.130 g, 0.571 mmol).
The obtained crude product was purified via flash-chromatography on silica gel using cyclohexane/ethyl acetate 98:2 to 85:15. R_f = 0.42 (cyclohexane/ethyl acetate 4:1).
^{1}H NMR (400 MHz, $CDCl_3$, ppm) δ = 1.54–1.71 (m, 2H), 1.73–1.81 (m, 4H), 2.04–2.11 (m, 1H), 2.16–2.40 (m, 4H), 2.75–2.87 (m, 1H), 3.27–3.36 (m, 4H); ^{13}C NMR (100 MHz, $CDCl_3$, ppm) δ = 17.5, 25.2, 28.9, 37.2, 37.6, 41.0, 45.2, 47.0, 76.7, 77.3, 123.3, 129.9, 210.8; EI (*m/z*, 70 eV, 40 °C): 228 (100) $[M]^+$, 171 (23), 143 (25), 142 (100), 130 (42); HRMS–EI (*m*/*z*): $[M]^+$ calcd for $C_{11}H_{16}OS_2$, 228.0637; found, 228.0635; IR (ATR, ṽ): 2954, 2913, 1695,

1603, 1421, 1364, 1345, 1318, 1266, 1224, 1181, 1105, 1061, 1038, 929, 891, 867, 755, 684, 651, 615, 499, 469 cm^{-1}.

4.34 3-(1-(1,3-Dithiolan-2-ylidene)propyl)cyclopentan-1-one (**9e**)

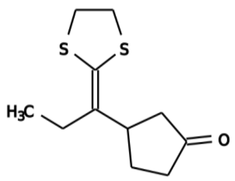

Name: 3-(1-(1,3-dithiolan-2-ylidene)propyl)cyclopentan-1-one; Formula: $C_{11}H_{16}OS_2$; CAS: - ; Smiles: CCC(=C1SCCS1)C1CCC(=O)C1; InCHI: BTUSJJHTMUJDHS-UHFFFAOYSA-N; Molecular Mass: 228.3741; Exact Mass: 228.0643; EA: C, 57.85; H, 7.06; O, 7.01; S, 28.08.

According to General Procedure 2a: {A|**1c**} 2-propyl-4,5-dihydro-1,3-dithiol-1-ium tetrafluoroborate (0.150 g, 0.641 mmol, 1.00 equiv); {B|**8a**} cyclopent-2-en-1-one (0.063 g, 0.769 mmol, 1.20 equiv); {C} acetonitrile (5.00 mL); Yield {D|**9e**} = 79% (0.115 g, 0.504 mmol).
The obtained crude product was purified via flash-chromatography on silica gel using cyclohexane/ethyl acetate 10:1. R_f = 0.63 (cyclohexane/ethyl acetate 2:1).
^{1}H NMR (500 MHz, $CDCl_3$, ppm) δ = 1.00 (t, *J* = 7.6 Hz, 3H), 1.68–1.82 (m, 1H), 2.01–2.19 (m, 5H), 2.25–2.36 (m, 2H), 3.15 (dt, *J* = 12.2 Hz, *J* = 6.0 Hz, 1H), 3.31 (s, 4H); ^{13}C NMR (125 MHz, $CDCl_3$, ppm), δ = 12.9, 25.7, 27.3, 37.2, 37.3, 38.3, 42.1, 44.7, 127.6, 131.2, 218.3; EI (*m/z*, 70 eV, 30 °C): 228 (23) $[M]^+$, 88 (10), 86 (61), 84 (100); HRMS–EI (*m*/*z*): $[M]^+$ calcd for $C_{11}H_{16}OS_2$, 228.0637; found, 228.0638; IR (ATR, ṽ): 2960, 2925, 2870, 1735, 1590, 1460, 1400, 1371, 1278, 1235, 1167, 1133, 1061, 976, 912, 873, 848, 828, 790, 730, 684, 647, 572, 544, 485 cm^{-1}.

4.35 3-(1-(1,3-Dithiolan-2-ylidene)propyl)cyclohexan-1-one (**9f**)

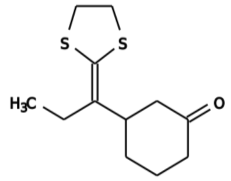

Name: 3-(1-(1,3-dithiolan-2-ylidene)propyl)cyclohexan-1-one; Formula: $C_{12}H_{18}OS_2$; CAS: - ; Smiles: CCC(=C1SCCS1)C1CCCC(=O)C1; InCHI: LDEHBRRLPOXYNP-UHFFFAOYSA-N; Molecular Mass: 242.4007; Exact Mass: 242.0799; EA: C, 59.46; H, 7.48; O, 6.6; S, 26.46.

According to General Procedure 2a: {A|**1c**} 2-propyl-4,5-dihydro-1,3-dithiol-1-ium tetrafluoroborate (0.161 g, 0.688 mmol, 1.00 equiv); {B|**8b**} cyclohex-2-en-1-one (0.079 g, 0.825 mmol, 1.20 equiv); {C} acetonitrile (5.00 mL); Yield {D|**9f**} = 40% (0.066 g, 0.274 mmol).
The obtained crude product was purified via flash-chromatography on silica gel using cyclohexane/ethyl acetate 10:1. R_f = 0.38 (cyclohexane/ethyl acetate 20:1).

^{1}H NMR (400 MHz, $CDCl_3$, ppm) δ = 1.07 (t, *J* = 7.6 Hz, 3H), 1.65–1.72 (m, 2H), 1.83–1.88 (m, 1H), 2.10 (dd, *J* = 5.8 Hz, *J* = 4.2 Hz, 1H), 2.22 (q, *J* = 7.6 Hz, 2H), 2.26–2.32 (m, 1H), 2.36–2.43 (m, 3H), 2.83 (d, *J* = 8.8 Hz, 1H), 3.34 (s, 4H); ^{13}C NMR (100 MHz, $CDCl_3$, ppm) δ = 13.1, 25.4, 26.1, 29.4, 37.2, 37.5, 41.2, 45.7, 47.4, 129.8, 130.8, 211.0; EI (*m/z*, 70 eV, 20 °C): 242 (100) $[M]^+$, 214 (47), 181 (21), 147 (21), 131 (71); HRMS–EI (*m*/*z*): [M] calcd for $C_{12}H_{18}OS_2$, 242.0799; found, 242.0798; IR (ATR, ṽ): 2929, 2869, 1725, 1458, 1404, 1378, 1335, 1295, 1275, 1214, 1155, 1104, 1021, 978, 910, 851, 817, 775, 681, 642, 499 cm^{-1}.

4.36 Methyl 2-(1,3-dithian-2-ylidene)-2-(3-oxocyclopentyl)acetate (**10g**)

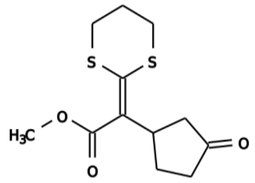

Name: Methyl 2-(1,3-dithian-2-ylidene)-2-(3-oxocyclopentyl)acetate; Formula: $C_{12}H_{16}O_3S_2$; CAS: - ; Smiles: COC(=O)C(=C1SCCCS1)C1CCC(=O)C1; InCHI: LXIYELLFKPNDKG-UHFFFAOYSA-N; Molecular Mass: 272.3836; Exact Mass: 272.0541; EA: C, 52.91; H, 5.92; O, 17.62; S, 23.54.

According to General Procedure 2a: {A|**2d**} 2-(2-methoxy-2-oxoethyl)-5,6-dihydro-4H-1,3-dithiin-1-ium tetrafluoroborate (0.150 g, 0.539 mmol, 1.00 equiv); {B|**8a**} cyclopent-2-en-1-one (0.053 g, 0.647 mmol, 1.20 equiv); {C} acetonitrile (5.00 mL); Yield {D|**10g**} = 68% (0.100 g, 0.366 mmol).
The obtained crude product was purified via flash-chromatography on silica gel using cyclohexane/ethyl acetate 10:1. R_f = 0.20 (cyclohexane/ethyl acetate 2:1).
^{1}H NMR (500 MHz, $CDCl_3$, ppm) δ = 1.97–2.08 (m, 1H), 2.09–2.22 (m, 4H), 2.28–2.44 (m, 3H), 2.85–2.95 (m, 2H), 2.99 (td, *J* = 7.0 Hz, *J* = 3.1 Hz, 2H), 3.72 (s, 3H), 3.73–3.81 (m, 1H); ^{13}C NMR (125 MHz, $CDCl_3$, ppm) δ = 23.7, 27.7, 29.2, 29.2, 38.4, 38.5, 42.5, 51.3, 127.7, 150.5, 165.9, 218.6; EI (*m/z*, 70 eV, 100 °C): 272 (68) $[M]^+$, 213 (22), 169 (26), 47 (100); HRMS–EI (*m*/*z*): $[M]^+$ calcd for $C_{12}H_{16}O_3S_2$, 272.0535; found, 272.0536; IR (ATR, ṽ): 2945, 2914, 1729, 1685, 1504, 1454, 1423, 1398, 1294, 1281, 1255, 1241, 1184, 1140, 989, 973, 871, 831, 785, 740, 710, 691, 658, 589, 505, 489, 424, 389 cm^{-1}.

4.37 Methyl 2-(1,3-dithian-2-ylidene)-2-(3-oxocyclohexyl)acetate (**10h**)

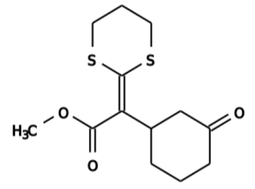

Name: Methyl 2-(1,3-dithian-2-ylidene)-2-(3-oxocyclohexyl)acetate; Formula: $C_{13}H_{18}O_3S_2$; CAS: - ; Smiles: COC(=O)C(=C1SCCCS1)C1CCCC(=O)C1; InCHI: AJGNXKNGUYYEFE-UHFFFAOYSA-N; Molecular Mass: 286.4102; Exact Mass: 286.0697; EA: C, 54.52; H, 6.33; O, 16.76; S, 22.39.

According to General Procedure 2a: {A|**2d**} 2-(2-methoxy-2-oxoethyl)-5,6-dihydro-4H-1,3-dithiin-1-ium tetrafluoroborate (0.150 g, 0.539 mmol, 1.00 equiv); {B|**8b**} cyclohex-2-en-1-one (0.062 g, 0.647 mmol, 1.20 equiv); {C} acetonitrile (5.00 mL); Yield {D|**10h**} = 42% (0.065 g, 0.228 mmol).
The obtained crude product was purified via flash-chromatography on silica gel using cyclohexane/ethyl acetate 10:1. R_f = 0.28 (cyclohexane/ethyl acetate 2:1).
^{1}H NMR (500 MHz, $CDCl_3$, ppm) δ = 1.63 (qt, *J* = 13.1 Hz, *J* = 4.1 Hz, 1H), 1.74 (dt, *J* = 13.1 Hz, *J* = 1.5 Hz, 1H), 1.90–1.99 (m, 1H), 2.04 (ddd, *J* = 13.3 Hz, *J* = 6.3 Hz, *J* = 3.1 Hz, 1H), 2.12 (quin, *J* = 6.9 Hz, 2H), 2.22–2.38 (m, 3H), 2.74 (t, *J* = 13.4 Hz, 1H), 2.88 (t, *J* = 6.5 Hz, 2H), 2.96 (td, *J* = 6.9 Hz, *J* = 1.1 Hz, 2H), 3.28–3.37 (m, 1H), 3.75 (s, 3H); ^{13}C NMR (125 MHz, $CDCl_3$, ppm) δ = 23.5, 25.0, 28.9, 29.0, 29.0, 41.0, 41.0, 45.4, 51.2, 128.8, 148.5, 166.1, 210.6; EI (*m/z*, 70 eV, 100 °C): 286 (100) $[M]^+$, 255 (21), 227 (33), 211 (34), 169 (8); HRMS–EI (*m*/*z*): $[M]^+$ calcd for $C_{13}H_{18}O_3S_2$, 286.0692; found, 286.0691; IR (ATR, ṽ): 2945, 2862, 1687, 1510, 1432, 1285, 1265, 1240, 1214, 1186, 1135, 1098, 995, 967, 935, 870, 779, 741, 710, 670, 559, 516 421, 380 cm^{-1}.

4.38 6-(1,3-Dithiolan-2-ylidene)-5-methylhexan-3-one (**4s**)

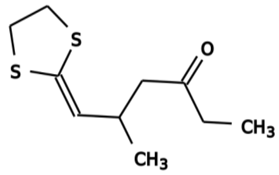

Name: 6-(1,3-dithiolan-2-ylidene)-5-methylhexan-3-one; Formula: $C_{10}H_{16}OS_2$; CAS: - ; Smiles: CCC(=O)CC(C=C1SCCS1)C; InCHI: UAXCQPMMEQTBAO-UHFFFAOYSA-N; Molecular Mass: 216.3634; Exact Mass: 216.0643; EA: C, 55.51; H, 7.45; O, 7.39; S, 29.64.

According to General Procedure 3: {A|**1e**} 2-methyl-4,5-dihydro-1,3-dithiol-1-ium tetrafluoroborate (0.500 g, 2.427 mmol, 1.00 equiv); {B|**3d**} (E)-hex-4-en-3-one (0.238 g, 2.427 mmol, 1.00 equiv); {C} acetonitrile (5.00 mL); Yield {D|**4s**} = 55% (0.288 g, 1.331 mmol).
The obtained crude product was purified via flash-chromatography on silica gel using cyclohexane/ethyl acetate 10:1. R_f = 0.50 (cyclohexane/ethyl acetate 20:1).
^{1}H NMR (500 MHz, $CDCl_3$, ppm) δ = 0.98–1.02 (m, 6H), 2.28–2.48 (m, 4H), 2.79 (dq, *J* = 9.27 Hz, *J* = 6.8 Hz, 1H), 3.24–3.30 (m, 2H), 3.32–3.37 (m, 2H), 5.31 (d, *J* = 9.3 Hz, 1H); ^{13}C NMR (125 MHz, $CDCl_3$, ppm) δ = 7.6, 20.2, 34.6, 36.1, 36.8, 37.9, 49.1, 120.5, 134.9, 210.3; EI (*m/z*, 70 eV, 50 °C): 216 (43) $[M]^+$, 145 (100); HRMS–EI (*m*/*z*): [M] calcd for $C_{10}H_{16}OS_2$, 216.0637; found, 216.0635; IR (ATR, ṽ): 2964, 2931, 2873, 1702, 1574, 1554, 1404, 1372, 1274, 1226, 1124, 1023, 980, 906, 859, 842, 798, 741, 688, 580, 529, 412 cm^{-1}.

4.39 4-((1,3-Dithiolan-2-ylidene)methyl)octan-2-one (**4t**)

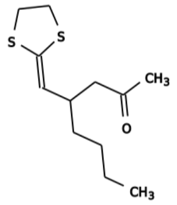

Name: 4-((1,3-dithiolan-2-ylidene)methyl)octan-2-one; Formula: $C_{12}H_{20}OS_2$; CAS: - ; Smiles: CCCCC(C=C1SCCS1)CC(=O)C; InCHI: UYKAJJCGWWOEHW-UHFFFAOYSA-N; Molecular Mass: 244.4166; Exact Mass: 244.0956; EA: C, 58.97; H, 8.25; O, 6.55; S, 26.24.

According to General Procedure 3: {A|**1e**} 2-methyl-4,5-dihydro-1,3-dithiol-1-ium tetrafluoroborate (0.151 g, 0.733 mmol, 1.00 equiv); {B|**3f**} (E)-oct-3-en-2-one (0.092 g, 0.731 mmol, 1.00 equiv); {C} acetonitrile (5.00 mL); Yield {D|**4t**} = 57% (0.102 g, 0.418 mmol).
The obtained crude product was purified via flash-chromatography on silica gel using cyclohexane/ethyl acetate 10:1. R_f = 0.61 (cyclohexane/ethyl acetate 2:1).
^{1}H NMR (500 MHz, $CDCl_3$, ppm) δ = 0.86 (t, *J* = 6.8 Hz, 3H), 1.19–1.42 (m, 6H), 2.12 (s, 3H), 2.41 (dd, *J* = 6.9 Hz, *J* = 2.6 Hz, 2H), 2.63–2.73 (m, 1H), 3.22–3.32 (m, 2H), 3.33–3.41 (m, 2H), 5.28 (d, *J* = 9.8 Hz, 1H); ^{13}C NMR (125 MHz, $CDCl_3$, ppm) δ = 14.0, 22.6, 29.3, 30.1, 35.1, 36.9, 37.9, 40.2, 49.2, 119.4, 136.0, 208.1; EI (*m/z*, 70 eV, 30 °C): 244 (43) $[M]^+$, 187 (100); HRMS–EI (*m*/*z*): $[M]^+$ calcd for $C_{12}H_{20}OS_2$, 244.0950; found, 244.0951; IR (ATR, ṽ): 2953, 2924, 2856, 1709, 1684, 1596, 1457, 1417, 1356, 1277, 1162, 1050, 827, 729, 683, 601, 547 cm^{-1}.

4.40 3-((1,3-Dithiolan-2-ylidene)methyl)cyclopentan-1-one (**4u**)

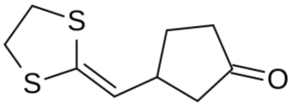

Name: 3-((1,3-Dithiolan-2-ylidene)methyl)cyclopentan-1-one; Formula: $C_9H_{12}OS_2$; CAS: - ; Smiles: O=C1CCC(C1)C=C1SCCS1; InCHI: OQIFLRJENUJSFT-UHFFFAOYSA-N; Molecular Mass: 200.3210; Exact Mass: 200.0330; EA: C, 53.96; H, 6.04; O, 7.99; S, 32.01.

According to General Procedure 3: {A|**1e**} 2-methyl-4,5-dihydro-1,3-dithiol-1-ium tetrafluoroborate (0.164 g, 0.796 mmol, 1.00 equiv); {B|**8a**} cyclopent-2-en-1-one (0.072 g, 0.876 mmol, 1.10 equiv); {C} acetonitrile (5.00 mL); Yield {D|**4u**} = 50% (0.081 g, 0.402 mmol).
The obtained crude product was purified via flash-chromatography on silica gel using cyclohexane/ethyl acetate 10:1. R_f = 0.61 (cyclohexane/ethyl acetate 2:1).
^{1}H NMR (500 MHz, $CDCl_3$, ppm) δ = 1.60–1.71 (m, 1H), 1.88–1.97 (m, 1H), 2.10–2.32 (m, 3H), 2.46 (dd, *J* = 18.2 Hz, *J* = 8.0 Hz, 1H), 2.88–2.99 (m, 1H), 3.28–3.34 (m, 2H), 3.36–3.41 (m, 2H), 5.46 (d, *J* = 8.7 Hz, 1H); ^{13}C NMR (125 MHz, $CDCl_3$, ppm) δ = 29.5, 37.0, 38.0, 38.1, 40.9, 44.5, 118.0, 136.8, 218.4; EI (*m/z*, 70 eV, 30 °C): 200 (55) $[M]^+$, 144 (17), 116 (50), 84 (100); HRMS–EI (*m*/*z*): $[M]^+$ calcd for $C_9H_{12}OS_2$, 200.0324; found, 200.0322; IR (ATR, ṽ): 2923, 1734, 1676, 1400, 1277, 1237, 1156, 986, 847, 767, 729, 575, 485 cm^{-1}.

4.41 3-((1,3-Dithiolan-2-ylidene)methyl)cyclohexan-1-one (**4v**)

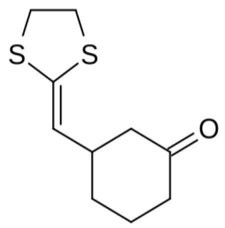

Name: 3-((1,3-dithiolan-2-ylidene)methyl)cyclohexan-1-one; Formula: $C_{10}H_{14}OS_2$; CAS: - ; Smiles: O=C1CCCC(C1)C=C1SCCS1; InCHI: SGGJGNOFBJFXPU-UHFFFAOYSA-N; Molecular Mass: 214.3476; Exact Mass: 214.0486; EA: C, 56.03; H, 6.58; O, 7.46; S, 29.92.

According to General Procedure 3: {A|**1e**} 2-methyl-4,5-dihydro-1,3-dithiol-1-ium tetrafluoroborate (0.150 g, 0.728 mmol, 1.00 equiv); {B|**8b**} cyclohex-2-en-1-one (0.070 g, 0.728 mmol, 1.00 equiv); {C} acetonitrile (5.00 mL); Yield {D|**4v**} = 23% (0.036 g, 0.166 mmol).
The obtained crude product was purified via flash-chromatography on silica gel using cyclohexane/ethyl acetate 10:1. R_f = 0.61 (cyclohexane/ethyl acetate 2:1).
^{1}H NMR (500 MHz, $CDCl_3$, ppm) δ = 1.45–1.55 (m, 1H), 1.70 (dtdd, *J* = 13.5 Hz, *J* = 11.9 Hz, *J* = 4.8 Hz, *J* = 3.5 Hz, 1H), 1.91–1.97 (m, 1H), 1.99–2.07 (m, 1H), 2.13 (ddd, *J* = 14.0 Hz, *J* = 11.3 Hz, *J* = 1.1 Hz, 1H), 2.22–2.29 (m, 1H), 2.32–2.38 (m, 1H), 2.48 (ddt, *J* = 14.0 Hz, *J* = 4.3 Hz, *J* = 1.9 Hz, 1H), 2.58–2.67 (m, 1H), 3.29–3.33 (m, 2H), 3.36–3.40 (m, 2H), 5.40 (d, *J* = 8.9 Hz, 1H); ^{13}C NMR (125 MHz, $CDCl_3$, ppm) δ = 24.9, 30.9, 36.9, 38.0, 41.1, 43.2, 47.1, 118.5, 136.1, 210.5; EI (*m/z*, 70 eV, 90 °C): 214 (100) $[M]^+$, 186 (22), 157 (40), 129 (21), 116 (46), 84 (78); HRMS–EI (*m*/*z*): $[M]^+$ calcd for $C_{10}H_{14}OS_2$, 214.0481; found, 214.0479; IR (ATR, ṽ): 2926, 2862, 1705, 1593, 1446, 1417, 1344, 1311, 1256, 1223, 1104, 1058, 992, 949, 922, 865, 829, 731, 701, 645, 583, 561, 504, 447, 387 cm^{-1}.

4.42 6-(1,3-Dithiolan-2-ylidene)-5,7-dimethylundecane-3,9-dione (**11a**)

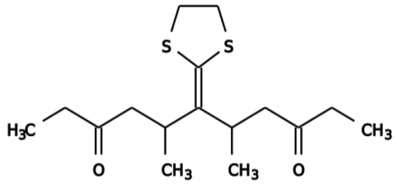

Name: 6-(1,3-dithiolan-2-ylidene)-5,7-dimethylundecane-3,9-dione; Formula: $C_{16}H_{26}O_2S_2$; CAS: - ; Smiles: CCC(=O)CC(C(=C1SCCS1)C(CC(=O)CC)C)C; InCHI: BAPCQQRWXYOJGB-UHFFFAOYSA-N; Molecular Mass: 314.5064; Exact Mass: 314.1374; EA: C, 61.1; H, 8.33; O, 10.17; S, 20.39.

According to General Procedure 4: {A|**1e**} 2-methyl-4,5-dihydro-1,3-dithiol-1-ium tetrafluoroborate (0.150 g, 0.728 mmol, 1.00 equiv); {B|**3d**} (E)-hex-4-en-3-one (0.214 g, 2.184 mmol, 3.00 equiv); {C} acetonitrile (5.00 mL); Yield {D|**11a**} = 13% (0.030 g, 0.096 mmol).
The obtained crude product was purified via flash-chromatography on silica gel using cyclohexane/ethyl acetate 10:1. R_f = 0.51 (cyclohexane/ethyl acetate 2:1).
^{1}H NMR (500 MHz, $CDCl_3$, ppm) δ = 1.03 (t, *J* = 7.2 Hz, 6H), 1.06 (d, *J* = 6.0 Hz, 1H, dia-1), 1.11 (d, *J* = 7.0 Hz, 5H, dia-2), 2.42 (q, *J* = 7.3 Hz, 4H), 2.52–2.58 (m, 2H), 2.65–2.76 (m,

2H), 3.02–3.10 (m, 2H), 3.27–3.32 (m, 4H); ^{13}C NMR (125 MHz, $CDCl_3$, ppm) δ = 7.8 (2C), 17.8 (dia-2, minor, 2C), 18.2 (dia-1, major, 2C), 36.3 (dia-1, major, 2C), 36.4 (dia-2, minor, 2C), 37.2 (bs, 2C), 46.7 (2C), 130.0, 133.6, 210.6 (2C); EI (*m/z*, 70 eV, 80 °C): 314 (68) $[M]^+$, 243 (55), 216 (36), 185 (24), 171 (100), 145 (52); HRMS–EI (*m*/*z*): $[M]^+$ calcd for $C_{16}H_{26}O_2S_2$, 314.1369; found, 314.1367; IR (ATR, ṽ): 2965, 2928, 1708, 1455, 1410, 1370, 1276, 1209, 1113, 1018, 939, 842, 684, 510 cm^{-1}.

4.43 3,3'-((1,3-Dithiolan-2-ylidene)methylene)bis(cyclopentan-1-one) (**11b**)

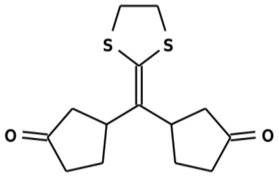

Name: 3,3'-((1,3-dithiolan-2-ylidene)methylene)bis(cyclopentan-1-one); Formula: $C_{14}H_{18}O_2S_2$; CAS: - ; Smiles: O=C1CCC(C1)C(=C1SCCS1)C1CCC(=O)C1; InCHI: DENARIJVSXWQSI-UHFFFAOYSA-N; Molecular Mass: 282.4215; Exact Mass: 282.0748; EA: C, 59.54; H, 6.42; O, 11.33; S, 22.71.

According to General Procedure 4: {A|**1e**} 2-methyl-4,5-dihydro-1,3-dithiol-1-ium tetrafluoroborate (0.252 g, 1.223 mmol, 1.00 equiv); {B|**8a**} cyclopent-2-en-1-one (0.301 g, 3.669 mmol, 3.00 equiv); {C} acetonitrile (5.00 mL); Yield {D|**11b**} = 67% (0.232 g, 0.822 mmol).
The obtained crude product was purified via flash-chromatography on silica gel using cyclohexane/ethyl acetate 10:1. R_f = 0.33 (cyclohexane/ethyl acetate 2:1).
^{1}H NMR (400 MHz, $CDCl_3$, ppm) δ = 1.88–2.35 (m, 10 H), 2.34–2.52 (m, 2 H), 3.00–3.14 (m, 2 H), 3.18–3.32 (m, 4 H); ^{13}C NMR (100 MHz, $CDCl_3$, ppm) δ = 2x26.4 (2C, 2 dia*), 37.1 (2C), 38.4 (2C), 40.8 (2C), 2x41.8 (2C, 2 dia*), 2x125.8 (1C, 2 dia*), 2x132.2 (1C, 2 dia*), 217.5+217.6 (2C, 2 dia*). *dia = 2 Cs as diastereomers; EI (*m/z*, 70 eV, 90 °C): 282 (100) $[M]^+$, 201 (25), 199 (47), 181 (27), 131 (35), 69 (38), 55 (20); HRMS–EI (*m*/*z*): [M] calcd for $C_{14}H_{18}O_2S_2$, 282.0748; found, 282.0749; IR (ATR, ṽ): 2894, 1731, 1585, 1456, 1399, 1280, 1238, 1153, 1153, 1008, 976, 927, 839, 810, 730, 682, 661, 569, 508, 483 cm^{-1}.

4.44 Methyl 4-(1,3-dithiolan-2-ylidene)hexanoate (**13a**)

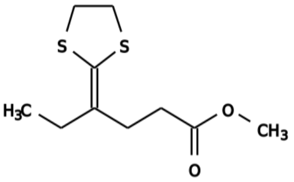

Name: Methyl 4-(1,3-dithiolan-2-ylidene)hexanoate; Formula: $C_{10}H_{16}O_2S_2$; CAS: - ; Smiles: CCC(=C1SCCS1)CCC(=O)OC; InCHI: WEWHOQGGMKXBGI-UHFFFAOYSA-N; Molecular Mass: 232.3628; Exact Mass: 232.0592; EA: C, 51.69; H, 6.94; O, 13.77; S, 27.6.

According to General Procedure 6: {A|**1c**} 2-propyl-4,5-dihydro-1,3-dithiol-1-ium tetrafluoroborate (0.300 g, 1.28 mmol, 1.00 equiv); {B|**12a** } methyl prop-2-enoate (0.110 g,

1.28 mmol, 1.00 equiv); {C} acetonitrile (3.0 mL); {D|**13a**} = 24% (0.0714 g, 0.307 mmol). The reaction was allowed to stir at 80 °C for 24 h.
According to General Procedure 5: {A|**1c**} 2-propyl-4,5-dihydro-1,3-dithiol-1-ium tetrafluoroborate (0.101 g, 0.431 mmol, 1.00 equiv); {B|**12a**} methyl prop-2-enoate (0.045 g, 0.517 mmol, 1.20 equiv); {C} dimethylalumanylium chloride (0.057 g, 0.621 mmol, 1.44 equiv); {D} methylene chloride (5.00 mL); Yield {E|**13a**} = 100% (0.100 g, 0.430 mmol).
Full conversion was detected after 2 h and the obtained crude product was purified via flash-chromatography on silica gel using cyclohexane/ethyl acetate 95:5. R_f = 0.60 (cyclohexane/ethyl acetate 4:1).
^{1}H NMR (400 MHz, $CDCl_3$, ppm) δ = 1.03 (t, *J* = 7.5 Hz, 3H), 2.19 (q, *J* = 7.6 Hz, 2H), 2.41–2.55 (m, 4H), 3.35 (s, 4H), 3.67–3.72 (m, 3H); ^{13}C NMR (100 MHz, $CDCl_3$, ppm) δ = 12.0, 29.6, 31.3, 32.0, 37.6, 37.7, 51.6, 126.9, 130.1, 173.6; EI (*m/z*, 70 eV, 20 °C): 232 (49) $[M]^+$, 217 (16), 159 (100), 145 (22), 105 (83); HRMS–EI (*m*/*z*): $[M]^+$ calcd for $C_{10}H_{16}O_2S_2$, 232.0586; found, 232.0588; IR (ATR, ṽ): 2960, 2926, 1731, 1600, 1434, 1361, 1247, 1193, 1156, 1084, 1057, 1028, 849, 645 cm^{-1}.

4.45 Dimethyl 2-(1-(1,3-dithiolan-2-ylidene)propyl)succinate (**13b**)

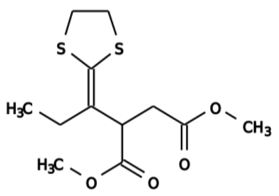

Name: Dimethyl 2-(1-(1,3-dithiolan-2-ylidene)propyl)succinate; Formula: $C_{12}H_{18}O_4S_2$; CAS: - ; Smiles: CCC(=C1SCCS1)C(C(=O)OC)CC(=O)OC; InCHI: MRPUMAFLNFWMQI-UHFFFAOYSA-N; Molecular Mass: 290.3989; Exact Mass: 290.0647; EA: C, 49.63; H, 6.25; O, 22.04; S, 22.08.

According to General Procedure 6: {A|**1c**} 2-propyl-4,5-dihydro-1,3-dithiol-1-ium tetrafluoroborate (0.300 g, 1.28 mmol, 1.00 equiv); {B|**12b**} dimethyl (E)-but-2-enedioate (0.185 g, 1.28 mmol, 1.00 equiv); {C} acetonitrile (3.0 mL); Yield {D|**13b**} = 17% (0.0646 g, 0.222 mmol). The reaction was allowed to stir at 80 °C for 48 h.
According to General Procedure 5: {A|**1c**} 2-propyl-4,5-dihydro-1,3-dithiol-1-ium tetrafluoroborate (0.300 g, 1.282 mmol, 1.00 equiv); {B|**12b**} dimethyl (E)-but-2-enedioate (0.222 g, 1.538 mmol, 1.20 equiv); {C} dimethylalumanylium chloride (0.119 g, 1.282 mmol, 1.00 equiv); {D} methylene chloride (5.00 mL); Yield {E|**13b**} = 60% (0.222 g, 0.764 mmol). Full conversion of the starting material was detected after 18 h.
The obtained crude product was purified via flash-chromatography on silica gel using cyclohexane/ethyl acetate, 98:2 to 95:5 to 90:10. R_f = 0.28 (cyclohexane/ethyl acetate 4:1).
^{1}H NMR (400 MHz, $CDCl_3$, ppm) δ = 0.98 (t, *J* = 7.5 Hz, 3H), 2.07–2.27 (m, 2H), 2.45 (dd, *J* = 16.8 Hz, *J* = 4.5 Hz, 1H), 3.03 (dd, *J* = 16.8 Hz, *J* = 10.4 Hz, 1H), 3.31–3.42 (m, 4H), 3.69 (s, 3H), 3.70 (s, 3H), 3.97 (dd, *J* = 10.4 Hz, *J* = 4.5 Hz, 1H); ^{13}C NMR (100 MHz, $CDCl_3$, ppm) δ = 12.1, 27.0, 34.1, 37.6, 37.7, 48.3, 51.7, 52.0, 123.4, 135.4, 172.0, 172.7; EI (*m/z*, 70 eV, 80 °C): 290 (48) $[M]^+$, 258 (30), 321 (100), 202 (23), 131 (25), 69 (30); HRMS–EI (*m*/*z*): $[M]^+$ calcd for $C_{12}H_{18}O_4S_2$, 290.0641; found, 290.0642; IR (ATR, ṽ): 2945, 1731, 1587, 1438, 1401, 1364, 1332, 1261, 1215, 1149, 1093, 1056, 995, 981, 961, 892, 846, 828, 756, 684, 633, 556, 420 cm^{-1}.

4.46 3-(Chloro(1,3-dithiolan-2-ylidene)methyl)cyclohexan-1-one (**9i**)

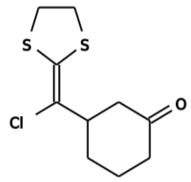

Name: 3-(Chloro(1,3-dithiolan-2-ylidene)methyl)cyclohexan-1-one; Formula: $C_{10}H_{13}ClOS_2$; CAS: - ; Smiles: O=C1CCCC(C1)C(=C1SCCS1)Cl; InCHI: NBLKWHHUXUGJIG-UHFFFAOYSA-N; Molecular Mass: 248.7926; Exact Mass: 248.0096; EA: C, 48.28; Cl, 14.25; H, 5.27; O, 6.43; S, 25.78.

According to General Procedure 6: {A|**1f**} 2-(chloromethyl)-4,5-dihydro-1,3-dithiol-1-ium tetrafluoroborate (0.308 g, 1.281 mmol, 1.00 equiv); {B|**8b**} cyclohex-2-en-1-one (0.148 g, 1.537 mmol, 1.20 equiv); {C} acetonitrile (5.00 mL); Yield {D|**9i**} = 24% (0.077 g, 0.309 mmol).
The reaction was stirred at room temperature for 24 h, and the obtained crude product was purified via flash-chromatography on silica gel using cyclohexane/ethyl acetate 100% to 19:1. R_f = 0.36 (cyclohexane/ethyl acetate 4:1).
^{1}H NMR (400 MHz, $CDCl_3$, ppm) δ = 1.60–1.89 (m, 3H), 2.09 (ddd, *J* = 9.8 Hz, *J* = 6.4 Hz, *J* = 2.9 Hz, 1H), 2.21–2.31 (m, 1H), 2.36 (dd, *J* = 5.1 Hz, *J* = 1.5 Hz, 2H), 2.50–2.59 (m, 1H), 2.98–3.07 (m, 1H), 3.34–3.48 (m, 4H); ^{13}C NMR (100 MHz, $CDCl_3$, ppm) δ = 24.4, 29.0, 37.5, 39.1, 40.8, 45.1, 46.6, 118.7, 134.0, 210.1; EI (*m/z*, 70 eV, 30 °C): 248/250 (2/0.3) $[M]^+$, 213 (2), 191/193 (1/0.5), 181 (2), 84 (100); HRMS–EI (*m*/*z*): $[M]^+$ calcd for $C_{21}H_{22}OS_2$, 248.0096; found, 248.0098; IR (ATR, ṽ): 2929, 2863, 1708, 1587, 1447, 1419, 1344, 1314, 1257, 1221, 1184, 1150, 1121, 1055, 1030, 978, 960, 935, 909, 867, 851, 733, 702, 503, 402 cm^{-1}.

4.47 3-(3-Chloro-1-(1,3-dithiolan-2-ylidene)propyl)cyclohexan-1-one (**9j**)

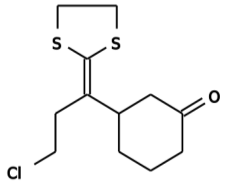

Name: 3-(3-Chloro-1-(1,3-dithiolan-2-ylidene)propyl)cyclohexan-1-one; Formula: $C_{12}H_{17}ClOS_2$; CAS: - ; Smiles: ClCCC(=C1SCCS1)C1CCCC(=O)C1; InCHI: XCZVBGQHMCFXSF-UHFFFAOYSA-N; Molecular Mass: 276.8458; Exact Mass: 276.0409; EA: C, 52.06; Cl, 12.81; H, 6.19; O, 5.78; S, 23.16.

According to General Procedure 6: {A|**1g**} 2-(3-chloropropyl)-4,5-dihydro-1,3-dithiol-1-ium tetrafluoroborate (0.300 g, 1.117 mmol, 1.00 equiv); {B|**8b**} cyclohex-2-en-1-one (0.129 g, 1.341 mmol, 1.20 equiv); {C} acetonitrile (5.00 mL); Yield {D|**9j**} = 64% (0.198 g, 0.714 mmol).
The reaction was finished after 5 min and workup was started after 30 min of reaction time. The obtained crude product was purified via flash-chromatography on silica gel using cyclohexane/ethyl acetate 100% to 19:1. R_f = 0.45 (cyclohexane/ethyl acetate 4:1).

^{1}H NMR (400 MHz, $CDCl_3$, ppm) δ = 1.60– 2.37 (m, 8H), 2.59–2.63 (m, 2H), 2.76 (dd, *J* = 8.7 Hz, *J* = 2.9 Hz, 1H), 3.32 (s, 4H), 3.47–3.53 (m, 2H); ^{13}C NMR (100 MHz, $CDCl_3$, ppm) δ = 25.1, 29.2, 36.3, 37.3, 37.5, 40.8, 41.2, 45.3, 46.8, 123.5, 136.0, 210.0; EI (*m/z*, 70 eV, 50 °C): 276 (13) $[M]^+$, 241(10), 243 (10), 227 (18), 218 (72), 181 (20), 122 (47); HRMS–EI (*m*/*z*): $[M]^+$ calcd for $C_{12}H_{17}OClS_2$, 276.0404; found, 276.0403; IR (ATR, ṽ): 2958, 2924, 2861, 1697, 1578, 1450, 1418, 1354, 1313, 1283, 1260, 1246, 1217, 1138, 1034, 980, 938, 909, 874, 850, 765, 725, 668, 627, 614, 514 cm^{-1}.

4.48 5-(1,3-Dithiolan-2-ylidene)-5-phenylpentan-2-ol (**16a**)

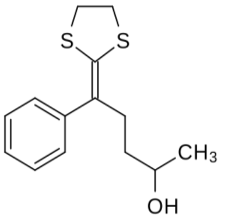

Name: 5-(1,3-Dithiolan-2-ylidene)-5-phenylpentan-2-ol; Formula: $C_{14}H_{18}OS_2$; CAS: - ; Smiles: CC(CCC(=C1SCCS1)c1ccccc1)O; InCHI: OOECJNICVMRHHH-UHFFFAOYSA-N; Molecular Mass: 266.4221; Exact Mass: 266.0799; EA: C, 63.11; H, 6.81; O, 6.01; S, 24.07.

According to General Procedure 7: {A|**4a**} 5-(1,3-dithiolan-2-ylidene)-5-phenylpentan-2-one (0.270 g, 1.021 mmol, 1.00 equiv); {B} sodium;boranuide (0.046 g, 1.225 mmol, 1.20 equiv); {C} methanol (5.00 mL); Yield {D|**16a**} = 94% (0.256 g, 0.962 mmol).
The obtained crude product was purified via flash-chromatography on silica gel using cyclohexane/ethyl acetate 4:1. R_f = 0.25 (cyclohexane/ethyl acetate 4:1).
^{1}H NMR (400 MHz, $CDCl_3$, ppm) δ = 1.08 (d, *J* = 6.2 Hz, 3H), 1.40–1.48 (m, 3H), 1.51 (br. s., 3H), 2.40–1.69 (m, 3H), 3.40–1.25 (m, 3H), 3.40–1.40 (m, 3H), 3.40–1.79 (m, 3H), 7.40–1.30 (m, 3H); ^{13}C NMR (100 MHz, $CDCl_3$, ppm) δ = 23.3, 35.2, 37.1, 37.8, 37.8, 67.3, 127.0, 127.9, 128.0 (2C), 128.3 (2C), 133.3, 142.5; EI (*m/z*, 70 eV, 60 °C): 266 (75) $[M]^+$, 207 (77), 181 (38), 161 (22), 147 (28), 131 (40), 105 (100), 84 (63), 69 (52); HRMS–EI (*m*/*z*): [M] calcd for $C_{14}H_{18}OS_2$, 266.0799; found, 266.0800; IR (ATR, ṽ): 3351, 3051, 2962, 2922, 1595, 1490, 1439, 1372, 1278, 1187, 1125, 1080, 1026, 961, 931, 848, 764, 700, 646, 609, 561, 492 cm^{-1}.

4.49 5-(1,3-Dithiolan-2-ylidene)-4-methyl-5-phenylpentan-2-ol (**16b**)

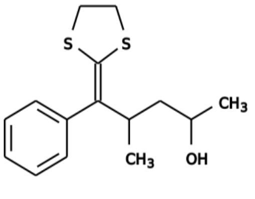

Name: 5-(1,3-dithiolan-2-ylidene)-4-methyl-5-phenylpentan-2-ol; Formula: $C_{15}H_{20}OS_2$; CAS: - ; Smiles: CC(C(=C1SCCS1)c1ccccc1)CC(O)C; InCHI: IECVTZNNDJQHMF-UHFFFAOYSA-N; Molecular Mass: 280.4487; Exact Mass: 280.0956; EA: C, 64.24; H, 7.19; O, 5.7; S, 22.87.

According to General Procedure 7: {A|**4c**} 5-(1,3-dithiolan-2-ylidene)-4-methyl-5-phenylpentan-2-one (0.070 g, 0.251 mmol, 1.00 equiv); {B} sodium;boranuide (0.011 g, 0.302 mmol, 1.20 equiv); {C} methanol (5.00 mL); Yield {D|**16b**} = 99% (0.070 g, 0.249 mmol).
The obtained crude product was purified via flash-chromatography on silica gel using cyclohexane/ethyl acetate 4:1. R_f = 0.28 (cyclohexane/ethyl acetate 4:1).
^{1}H NMR (400 MHz, $CDCl_3$, ppm) δ = 1.01–1.07 (m, 3H), 1.23 (d, *J* = 6.2 Hz, dia-minor, 0.75H), 1.26 (d, *J* = 6.1 Hz, dia-major, 2.25H), 1.29–1.35 (m, 1H), 1.62–1.70 (m, 1H), 2.97–3.15 (m, 1H), 3.22–3.28 (m, 2H), 3.39–3.47 (m, 2H), 3.89–4.06 (m, 1H), 7.08–7.10 (m, dia-minor, 0.5H), 7.19 (m, 1.5H, dia-major), 7.28–7.41 (m, 3H); ^{13}C NMR (100 MHz, $CDCl_3$, ppm) δ = 19.1 (dia-major), 19.9 (dia-minor), 23.3 (dia-minor), 24.0 (dia-major), 37.2 (s), 37.8 (s), 39.2 (s), 44.7 (dia-minor), 44.9 (dia-major), 66.1 (dia-minor), 66.4 (dia-major), 127.1 (dia-major), 127.2 (dia-minor), 128.1 (2C, dia-major), 128.2 (2C, dia-minor), 129.4 (2C, dia-minor), 129.5 (2C, dia-major), 131.8 (dia-minor), 132.6 (dia-major), 133.6 (dia-major), 133.9 (dia-minor), 140.4 (dia-minor), 140.8 (dia-major); EI (*m/z*, 70 eV, 50 °C): 280 (29) $[M]^+$, 221 (63), 118 (23), 105 (33), 86 (59), 84 (100), 51 (31); HRMS–EI (*m*/*z*): $[M]^+$ calcd for $C_{15}H_{20}OS_2$, 280.0956; found, 280.0957; IR (ATR, ṽ): 3367, 3051, 2960, 2924, 1598, 1581, 1488, 1439, 1420, 1371, 1278, 1121, 1029, 982, 948, 917, 886, 843, 794, 747, 701, 644, 570, 543, 495 cm^{-1}.

4.50 4-(1,3-Dithiolan-2-ylidene)-1,3-diphenylhexan-1-one (**15**)

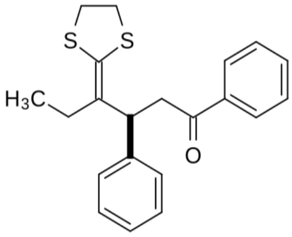

Name: 4-(1,3-Dithiolan-2-ylidene)-1,3-diphenylhexan-1-one; Formula: $C_{21}H_{22}OS_2$; CAS: - ; Smiles: CCC(=C1SCCS1)[C@H](c1ccccc1)CC(=O)c1ccccc1; InChI: FFNUZHPVLBYLFF-IBGZPJMESA-N; Molecular Mass: 354.5288; Exact Mass: 354.1112; EA: C, 71.14; H, 6.25; O, 4.51; S, 18.09.

The dithiolanylium tetrafluoroborate {**A**|**1c**} (1.00 equiv) was dissolved in dry acetonitrile {C} in a glass vial at room temperature (under N_2-atmosphere). The α,β-unsaturated imine {B|**14**} (1.00 equiv) was added in one portion at room temperature, the reaction was stirred at room temperature and its progress was observed via TLC control. Full conversion of the starting material was observed after 1 h and silica gel (3 g) was added. The the solvent was removed via evaporation under reduced pressure and the ee (80%) was determined via chiral HPLC (AD-column), n-heptane/isopropanol, 97:3.
{A|**1c**} 2-propyl-4,5-dihydro-1,3-dithiol-1-ium tetrafluoroborate (0.151 g, 0.645 mmol, 1.00 equiv); {B|**14**} (S)-N-((1Z,2E)-1,3-diphenylallylidene)-2-methylpropane-2-sulfinamide (0.240 g, 0.771 mmol, 1.20 equiv); {C} acetonitrile (5.00 mL); Yield {D|**15**} = 33% (0.074 g, 0.210 mmol).
The obtained crude product was purified via flash-chromatography on silica gel using cyclohexane/ethyl acetate 97:3 to 90:10. R_f = 0.73 (cyclohexane/ethyl acetate 2:1).
^{1}H NMR (500 MHz, $CDCl_3$, ppm) δ = 0.80 (t, *J* = 7.6 Hz, 3H), 2.09–2.19 (m, 3H), 3.09–2.40 (m, 3H), 3.48 (dd, *J* = 16.6 Hz, *J* = 6.4 Hz, 1H), 3.65–3.74 (m, 1H), 4.72 (dd, *J* = 8.2 Hz, *J* = 6.5 Hz, 1H), 7.18–7.23 (m, 1H), 7.18–7.31 (m, 1H), 7.18–7.51 (m, 1H), 7.18–7.61 (m, 1H),

7.18–7.03 (m, 1H); ^{13}C NMR (125 MHz, $CDCl_3$, ppm) δ = 12.7, 29.7, 37.5, 37.7, 40.5, 47.8, 126.4, 127.5 (2C), 128.1 (2C), 128.3 (2C), 128.5 (2C), 130.2, 131.6, 132.9, 137.0, 141.8, 198.1; EI (*m/z*, 70 eV, 80 °C): 354 (33) $[M]^+$, 235 (40), 210 (33), 105 (100), 86 (40), 84 (63), 57 (26); HRMS–EI (*m*/*z*): $[M]^+$ calcd for $C_{21}H_{22}OS_2$, 354.1107; found, 354.1105.

4.51 (*E*)-5-(1,3-Dithiolan-2-ylidene)-5-phenylpent-3-en-2-one (**18a**)

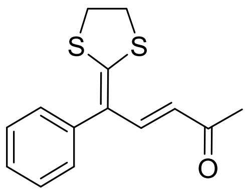

Name: (E)-5-(1,3-Dithiolan-2-ylidene)-5-phenylpent-3-en-2-one; Formula: $C_{14}H_{14}OS_2$; CAS: - ;Smiles: CC(=O)/C=C/C(=C1SCCS1)c1ccccc1; InCHI: UORDPIWKYNURLV-BQYQJAHWSA-N; Molecular Mass: 262.3904; Exact Mass: 262.0486; EA: C, 64.08; H, 5.38; O, 6.10; S, 24.44.

According to General Procedure 8: {A|**1a**} 2-benzyl-4,5-dihydro-1,3-dithiol-1-ium tetrafluoroborate (0.150 g, 0.531 mmol, 1.00 equiv); {B|**17**} but-3-yn-2-one (0.0457 g, 0.638 mmol, 1.20 equiv); {C} acetonitrile (1.50 mL); Yield {D|**18a**} = 24% (0.034 g, 0.129 mmol). The obtained crude product was purified via flash-chromatography on silica gel using cyclohexane/ethyl acetate 100% to 90:10. R_f = 0.45 (cyclohexane/ethyl acetate 4:1).
^{1}H NMR (400 MHz, $CDCl_3$, ppm) δ = 2.24 (s, 3H), 3.36–3.43 (m, 2H), 3.50–3.56 (m, 2H), 5.56 (d, *J* = 15.3 Hz, 1H), 7.18–7.21 (m, 2H), 7.32–7.46 (m, 3H), 7.69 (d, *J* = 15.3 Hz, 1H); ^{13}C NMR (100 MHz, $CDCl_3$, ppm) δ = 27.6, 38.0, 38.2, 125.1, 126.8, 128.0, 128.8 (s, 2C) 129.4 (s, 2C), 139.2, 142.2, 153.2, 198.4; APCI-MS (*m*/*z*): 263 (100) $[M+1]^+$, 223 (24), 203 (20).

4.52 (*E*)-5-(1,3-Dithiolan-2-ylidene)hex-3-en-2-one (**18b**)

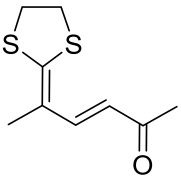

Name: (E)-5-(1,3-Dithiolan-2-ylidene)hex-3-en-2-one; Formula: $C_9H_{12}OS_2$; CAS: - ;Smiles: CC(=C1SCCS1)/C=C/C(=O)C; InCHI: LDGXSBDCBZHQOU-ONEGZZNKSA-N; Molecular Mass: 200.3210; Exact Mass: 200.0330; EA: C, 53.96; H, 6.04; O, 7.99; S, 32.01.

According to General Procedure 8: {A|**1b**} 2-ethyl-4,5-dihydro-1,3-dithiol-1-ium tetrefluoroborate (0.152 g, 0.691 mmol, 1.00 equiv); {B|**17**} but-3-yn-2-one (0.0564 g, 0.829 mmol, 1.20 equiv); {C} acetonitrile (1.50 mL), Yield {D|**18b**} = 52% (0.072 g, 0.357 mmol). The obtained crude product was purified via flash-chromatography on silica gel using cyclohexane/ethyl acetate 100% to 90:10. R_f = 0.45 (cyclohexane/ethyl acetate 2:1).
^{1}H NMR (400 MHz, $CDCl_3$, ppm) δ = 1.96 (m, 3H), 2.28 (m, 3H), 3.39–3.51 (m, 4H), 5.94 (d, *J* = 15.4 Hz, 1H), 7.51 (d, *J* = 15.4 Hz, 1H); ^{13}C NMR (100 MHz, $CDCl_3$, ppm) δ = 18.3, 27.2, 37.8, 37.9, 119.8, 122.9, 142.2, 150.6, 198.4; EI (*m/z*, 70 eV, 40 °C): 200 (89) $[M]^+$, 185(28), 180 (21), 159 (24), 144 (24), 132 (19), 131 (34), 130 (100), 129 (26), 97 (27), 96 (21), 95 (10), 85 (17), 69 (24), 58 (57), 53 (14); HRMS–EI (*m*/*z*): $[M]^+$ calcd for $C_9H_{12}OS_2$,

200.0330; found, 200.0332; IR (ATR, ṽ): 2920, 1656, 1627, 1589, 1532, 1422, 1378, 1354, 1255, 1242, 1152, 1106, 1040, 995, 981, 961, 928, 857, 835, 682, 641, 553, 528, 444 cm^{-1}.

4.53 *S*-(3-((2-Methyl-5-oxohexan-3-yl)thio)propyl) 2-phenylethanethioate (**7e**)

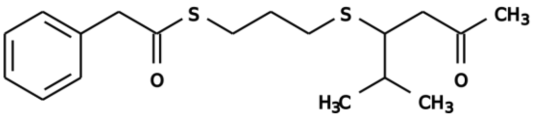

Name: S-(3-((2-methyl-5-oxohexan-3-yl)thio)propyl) 2-phenylethanethioate; Formula: $C_{18}H_{26}O_2S_2$; CAS: - ; Smiles: CC(C(CC(=O)C)SCCCSC(=O)Cc1ccccc1)C; InCHI: HAPMDIPOMSAKCB-UHFFFAOYSA-N; Molecular Mass: 338.5278; Exact Mass: 338.1374; EA: C, 63.86; H, 7.74; O, 9.45; S, 18.94.

According to General Procedure 2b: {A|**2a**} 2-benzyl-5,6-dihydro-4H-1,3-dithiin-1-ium tetrafluoroborate (0.150 g, 0.506 mmol, 1.00 equiv); {B|**3e**} (E)-5-methylhex-3-en-2-one (0.085 g, 0.760 mmol, 1.50 equiv); {C} acetonitrile (5.00 mL); Yield {D|**7e**} = 62% (0.106 g, 0.314 mmol).
The obtained crude product was purified via flash-chromatography on silica gel using cyclohexane/ethyl acetate 10:1. R_f = 0.48 (cyclohexane/ethyl acetate 4:1).
^{1}H NMR (400 MHz, $CDCl_3$, ppm) δ = 0.83 (d, *J* = 6.8 Hz, 3H), 0.89 (d, *J* = 6.7 Hz, 3H), 1.68–1.82 (m, 3H), 2.08 (s, 3H), 2.46 (t, *J* = 7.2 Hz, 2H), 2.51–2.61 (m, 2H), 2.85 (t, *J* = 7.1 Hz, 2H), 2.89–2.95 (m, 2H), 3.73 (s, 2H), 7.89–2.29 (m, 2H); ^{13}C NMR (100 MHz, $CDCl_3$, ppm) δ = 18.9, 19.4, 28.0, 29.3, 30.9, 31.4, 32.3, 46.8, 47.6, 50.4, 127.2, 128.5 (2C), 129.4 (2C), 133.5, 197.0, 207.0; EI (*m/z*, 70 eV, 80 °C): 338 (15) $[M]^+$, 219 (100), 113 (94), 95 (15), 91 (89); HRMS–EI (*m/z*): [M] calcd for $C_{18}H_{26}O_2S_2$, 338.1374; found, 338.1375; IR (ATR, ṽ): 3029, 2958, 2925, 2870, 1713, 1685, 1602, 1495, 1454, 1411, 1383, 1363, 1297, 1254, 1158, 1076, 1016, 999, 842, 754, 702, 590, 502, 430 cm^{-1}.

4.54 *S*-(3-((2-Methyl-5-oxohexan-3-yl)thio)propyl) propanethioate (**7j**)

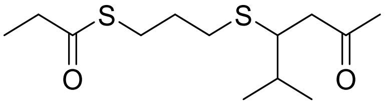

Name: S-(3-((2-methyl-5-oxohexan-3-yl)thio)propyl) propanethioate; Formula: $C_{13}H_{24}O_2S_2$; CAS: - ; Smiles: CCC(=O)SCCCSC(C(C)C)CC(=O)C; InCHI: LGBVJUDEOLYQDS-UHFFFAOYSA-N; Molecular Mass: 276.4585; Exact Mass: 276.1218; EA: C, 56.48; H, 8.75; O, 11.57; S, 23.2.

According to General Procedure 2b: {A|**2b**} 2-ethyl-5,6-dihydro-4H-1,3-dithiin-1-ium tetrafluoroborate (0.250 g, 1.068 mmol, 1.00 equiv); {B|**3e**} (E)-5-methylhex-3-en-2-one (0.180 g, 1.602 mmol, 1.50 equiv); {C} acetonitrile (5.00 mL); Yield {D|**7j**} = 30% (0.088 g, 0.319 mmol).
The obtained crude product was purified via flash-chromatography on silica gel using cyclohexane/ethyl acetate 10:1. R_f = 0.44 (cyclohexane/ethyl acetate 4:1).
^{1}H NMR (400 MHz, $CDCl_3$, ppm) δ = 0.89 (d, *J* = 6.7 Hz, 3H), 0.95 (d, *J* = 6.7 Hz, 3H), 1.14 (t, *J* = 7.5 Hz, 3H), 1.75–1.89 (m, 3H), 2.15 (s, 3H), 2.75–1.69 (m, 3H), 2.91 (t, *J* = 7.1 Hz, 2H) 2.96–3.01 (m, 2H); ^{13}C NMR (100 MHz, $CDCl_3$, ppm) δ = 9.6, 18.9, 19.4, 27.5, 29.5,

30.9, 31.4, 32.3, 37.3, 46.9, 47.7, 199.9, 207.0; EI (*m/z*, 70 eV, 30 °C): 276 (18) $[M]^+$, 219 (100), 181 (13), 113 (94); HRMS–EI (*m*/*z*): $[M]^+$ calcd for $C_{13}H_{24}O_2S_2$, 276.1218; found, 276.1219; IR (ATR, ṽ) = 2959, 1715, 1688, 1460, 1415, 1363, 1253, 1158, 1089, 1017, 933, 714, 590 cm^{-1}.

4.55 *S*-(3-((3-Oxopentyl)thio)propyl) butanethioate (**7m**)

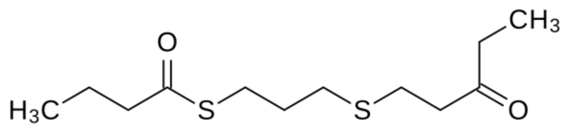

Name: S-(3-((3-oxopentyl)thio)propyl) butanethioate; Formula: $C_{12}H_{22}O_2S_2$; CAS: - ; Smiles: CCCC(=O)SCCCSCCC(=O)CC; InCHI: ZYLZEQDDPVIRMC-UHFFFAOYSA-N; Molecular Mass: 262.4319; Exact Mass: 262.1061; EA: C, 54.92; H, 8.45; O, 12.19; S, 24.44.

According to General Procedure 2a: {A|**2c**} 2-propyl-5,6-dihydro-4H-1,3-dithiin-1-ium tetrafluoroborate (0.200 g, 0.806 mmol, 1.00 equiv); {B|**3b**} pent-1-en-3-one (0.081 g, 0.967 mmol, 1.20 equiv); {C} acetonitrile (5.00 mL); Yield {D|**7m**} = 14% (0.030 g, 0.114 mmol).
The obtained crude product was purified via flash-chromatography on silica gel using cyclohexane/ethyl acetate 10:1. R_f = 0.48 (cyclohexane/ethyl acetate 4:1).
^{1}H NMR (400 MHz, $CDCl_3$, ppm) δ = 0.94 (t, *J* = 7.4 Hz, 3H), 1.06 (t, *J* = 7.3 Hz, 3H), 1.68 (sxt, *J* = 7.4 Hz, 2H), 1.85 (quin, *J* = 7.2 Hz, 2H), 2.44 (q, *J* = 7.3 Hz, 2H), 2.54 (dt, *J* = 17.7 Hz, 7.3 Hz, 4H), 2.66–2.77 (m, 4H), 2.95 (t, *J* = 7.2 Hz, 2H); ^{13}C NMR (100 MHz, $CDCl_3$, ppm) δ = 8.0, 13.8, 19.4, 26.1, 27.9, 29.7, 31.5, 36.5, 42.6, 46.2, 199.5, 209.8; EI (*m/z*, 70 eV, 40 °C): 262 (10) $[M]^+$, 191 (100); HRMS–EI (*m*/*z*): [M] calcd for $C_{12}H_{22}O_2S_2$, 262.1061; found, 262.1063; IR (ATR, ṽ) = 2964, 2933, 1712, 1685, 1457, 1411, 1353, 1255, 1111, 1050, 986, 886, 759, 698, 600 cm^{-1}.

4.56 *S*-(3-((4-Oxohexan-2-yl)thio)propyl) butanethioate (**7n**)

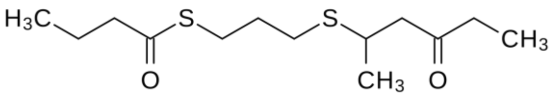

Name: S-(3-((4-oxohexan-2-yl)thio)propyl) butanethioate; Formula: $C_{13}H_{24}O_2S_2$; CAS: - ; Smiles: CCCC(=O)SCCCSC(CC(=O)CC)C; InCHI: YHZSWQASJCLPOE-UHFFFAOYSA-N; Molecular Mass: 276.4585; Exact Mass: 276.1218; EA: C, 56.48; H, 8.75; O, 11.57; S, 23.2.

According to General Procedure 2a: {A|**2c**} 2-propyl-5,6-dihydro-4H-1,3-dithiin-1-ium tetrafluoroborate (0.150 g, 0.605 mmol, 1.00 equiv); {B|**3d**} (E)-hex-4-en-3-one (0.071 g, 0.725 mmol, 1.20 equiv); {C} acetonitrile (5.00 mL); Yield {D|**7n**} = 46% (0.078 g, 0.281 mmol).
The obtained crude product was purified via flash-chromatography on silica gel using cyclohexane/ethyl acetate 10:1. R_f = 0.53 (cyclohexane/ethyl acetate 4:1).
^{1}H NMR (400 MHz, $CDCl_3$, ppm) δ = 0.92 (t, *J* = 7.4 Hz, 3H), 0.99–1.06 (m, 3H), 1.24 (d, *J* = 6.7 Hz, 3H), 1.65 (sxt, *J* = 7.4 Hz, 2H), 1.81 (quin, *J* = 7.2 Hz, 2H), 2.36–2.45 (m, 2H), 2.36–2.58 (m, 2H), 2.36–2.71 (m, 2H), 2.92 (t, *J* = 7.1 Hz, 2H), 3.21 (sxt, *J* = 6.8 Hz, 1H);

^{13}C NMR (100 MHz, $CDCl_3$, ppm) δ = 7.5, 13.4, 19.1, 21.6, 27.6, 29.5 (2C), 35.0, 36.7, 45.9, 49.6, 199.1, 209.1; EI (m/z, 70 eV): 276 (9) $[M]^+$, 205 (82), 57 (100); HRMS–EI (*m*/*z*): $[M]^+$ calcd for $C_{13}H_{24}O_2S_2$, 276.1218; found, 276.1216; IR (ATR, ṽ): 2964, 2933, 2875, 1712, 1686, 1456, 1410, 1358, 1297, 1256, 1112, 1050, 985, 886, 860, 794, 759, 697, 599 cm^{-1}.

5 Experiments to elucidate mechanistic aspects

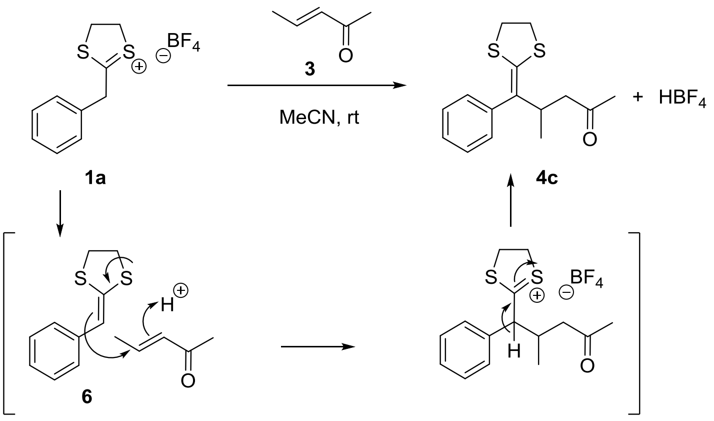

Scheme S1: Proposed mechanism of the HBF_4-catalyzed addition reaction.

Table S1: Experiments indicating the above shown mechanism.

Entry	Edukt	additive	Equiv additive	time	Yield 4c
1	**1a**	-		1 h	83%*
2	**1a**	NEt_3	1.2	24 h	-
3	**6**	-		24 h	-
4	**1a**	NEt_3	0.5	1 h	51%
5	**1a**	NEt_3	0.1	1 h	78%
6	**1a**	NEt_3	0.9	3 h	78%
7	**6**	HBF_4*Et_2O	1.2	1 h	77%
8	**6**	HBF_4*Et_2O	0.1	1 h	80%

*Workup of the reactions was different (aqueous workup) to the original table in the manuscript due to the need for a fast quenching of the reaction after 1 h. Therefore, the yield differs from the original experiment (entry 3, Table 1).
