# Supplementary material for: Addition of dithi(ol)anylium tetrafluoroborates to α,β-unsaturated ketones
Source: Beilstein J Org Chem. 2018 Feb 26;14:515–22. doi: 10.3762/bjoc.14.37 (PMC5852463; doi:10.3762/bjoc.14.37)
Supplement: File 2 — Collection of spectra and summary of IR data of starting materials. [file Beilstein_J_Org_Chem-14-515-s002.pdf]

Beilstein J. Org. Chem. **2018,** *14,* 515–522. doi:10.3762/bjoc.14.37

Supporting Information File 2

for

Addition of dithi(ol)anylium tetrafluoroborates to α,β-unsaturated ketones

Yu-Chieh Huang[1], An Nguyen[1], Simone Gräßle[1], Sylvia Vanderheiden[2], Nicole Jung*[1,2] and Stefan Bräse*[1,2]

Address: [1]Institute of Toxicology and Genetics, Karlsruhe Institute of Technology, Campus North, Hermann-von-Helmholtz-Platz 1, 76344 Eggenstein-Leopoldshafen, Germany and [2]Institute of Organic Chemistry, Karlsruhe Institute of Technology, Fritz-Haber-Weg 6, 76131 Karlsruhe, Germany

Email: Nicole Jung - nicole.jung@kit.edu; Stefan Bräse - braese@kit.edu

* Corresponding author

Collection of spectra and summary of IR data of starting materials

Contents

Spectra of compounds type **4** and **5** S2
Spectra of compounds type **9** and **10** S32
Spectra of compounds type **11a** and **11b** S45
Spectra of compounds **13a**, **13b**, **15**, **16a**, **16b** and **18a** and **18b** S47
Products of type **7**: ring opening by hydrolysis S54
Properties and IR (Raman) spectra of compounds **1** and **2** S58

Spectra of compounds type **4** and **5**

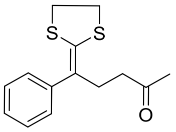

[4a] 5-(1,3-dithiolan-2-ylidene)-5-phenylpentan-2-one

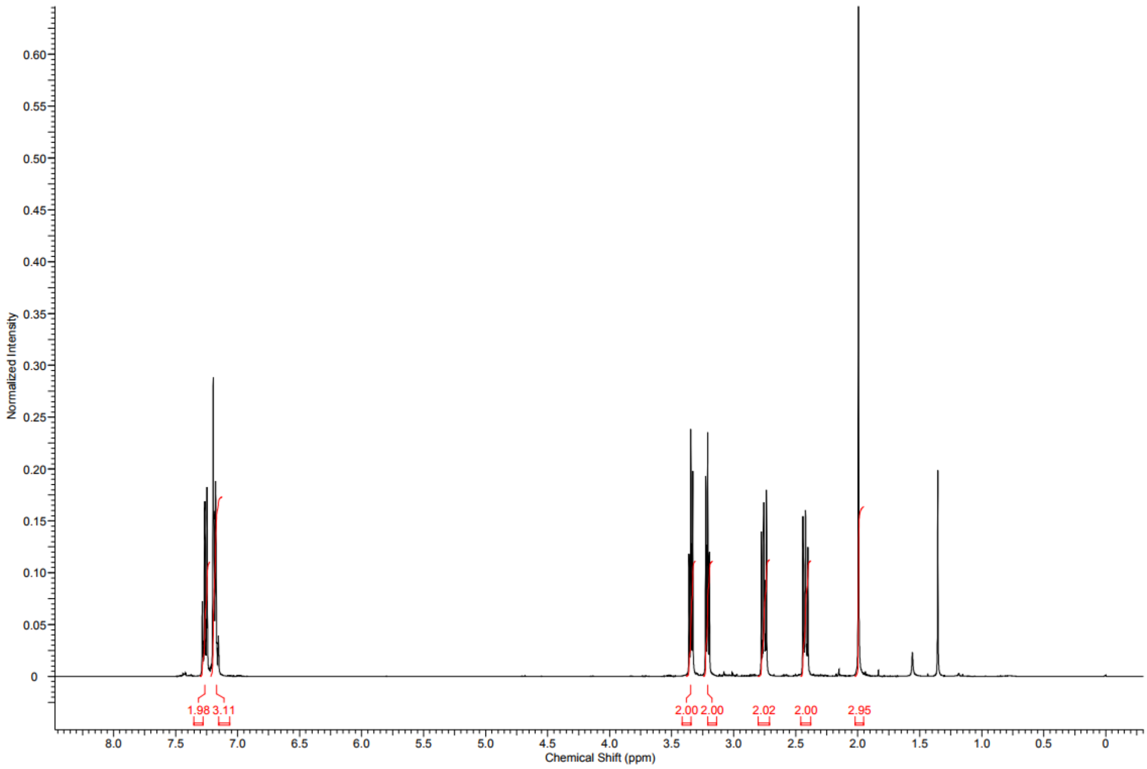

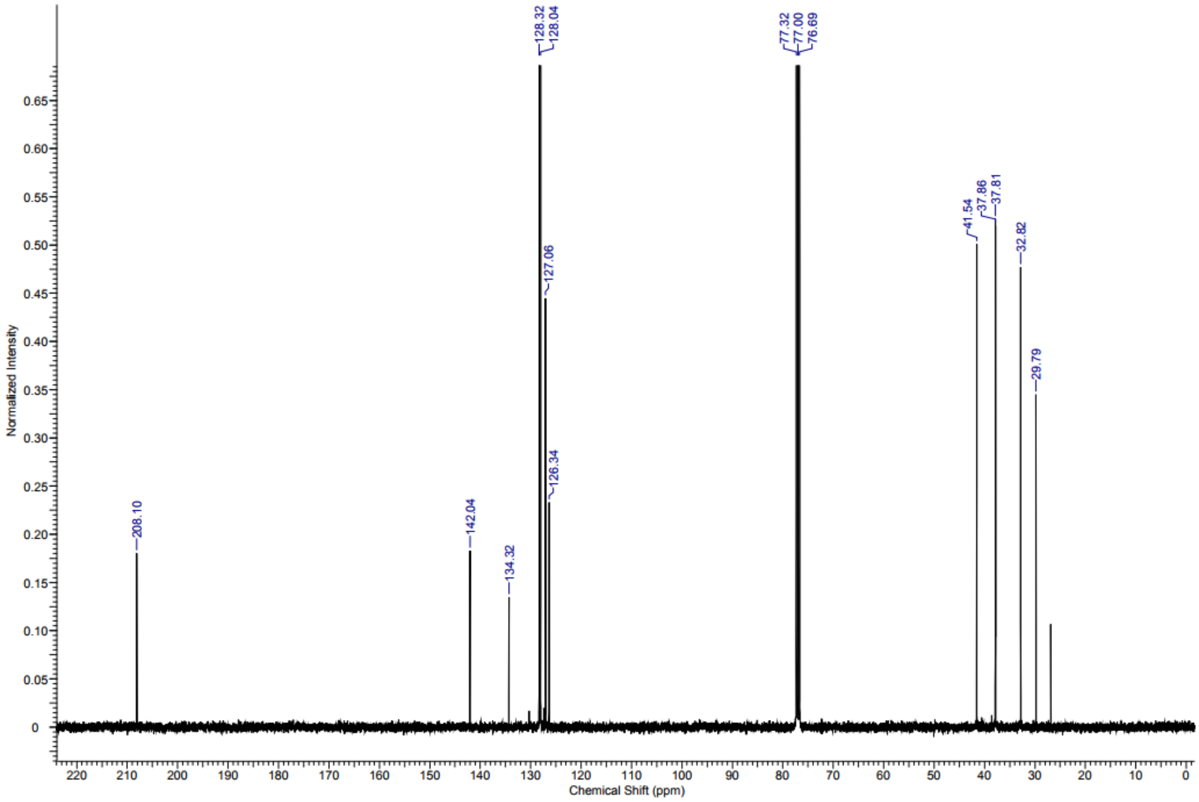

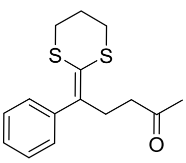

[5a] 5-(1,3-dithian-2-ylidene)-5-phenylpentan-2-one

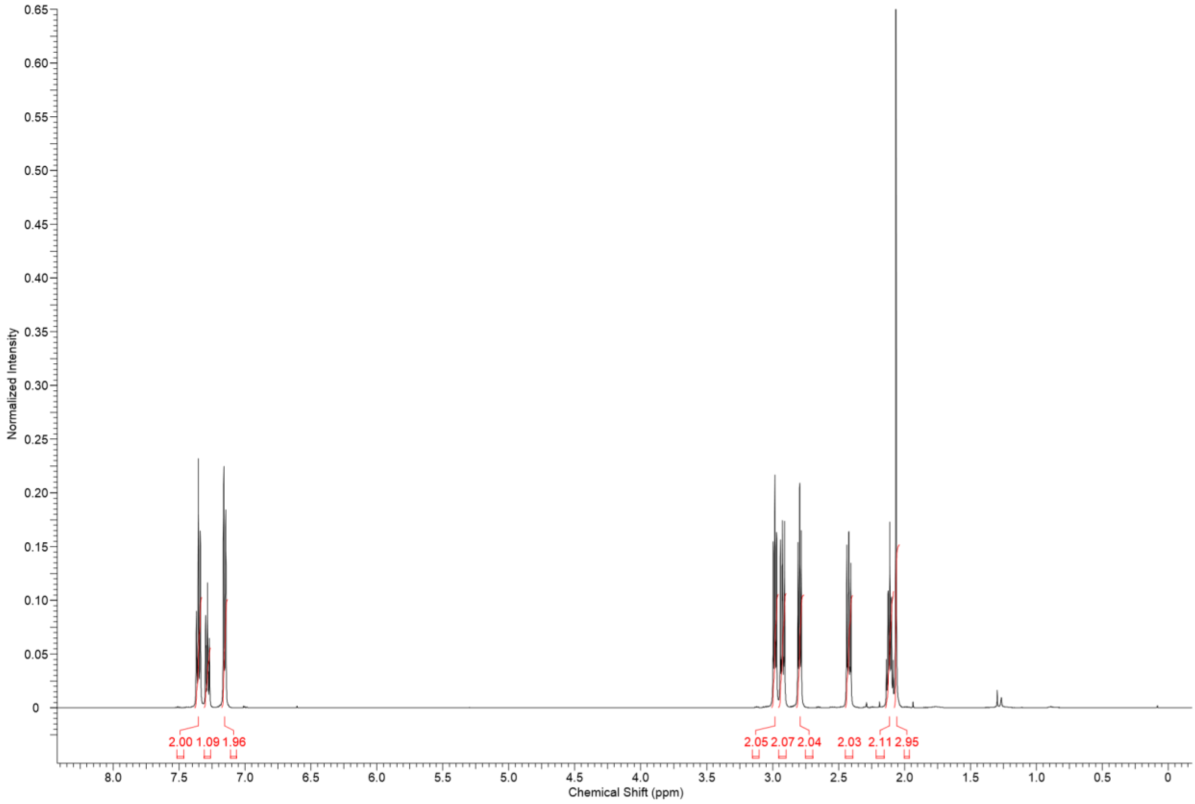

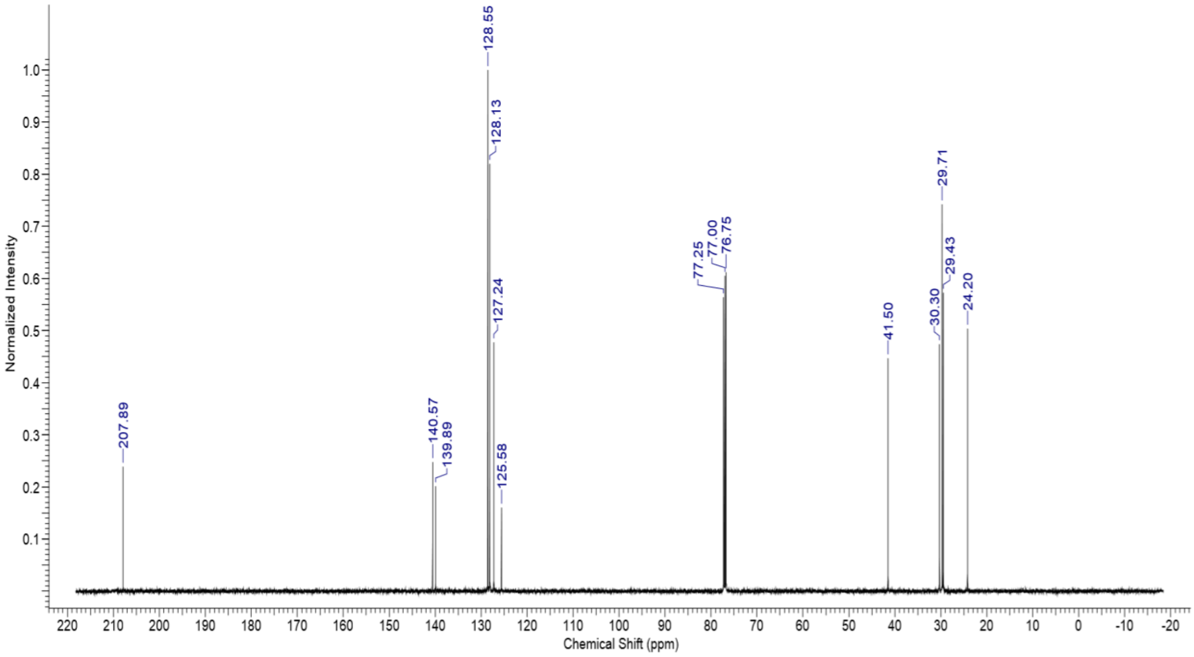

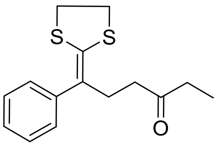

[4b] 6-(1,3-dithiolan-2-ylidene)-6-phenylhexan-3-one

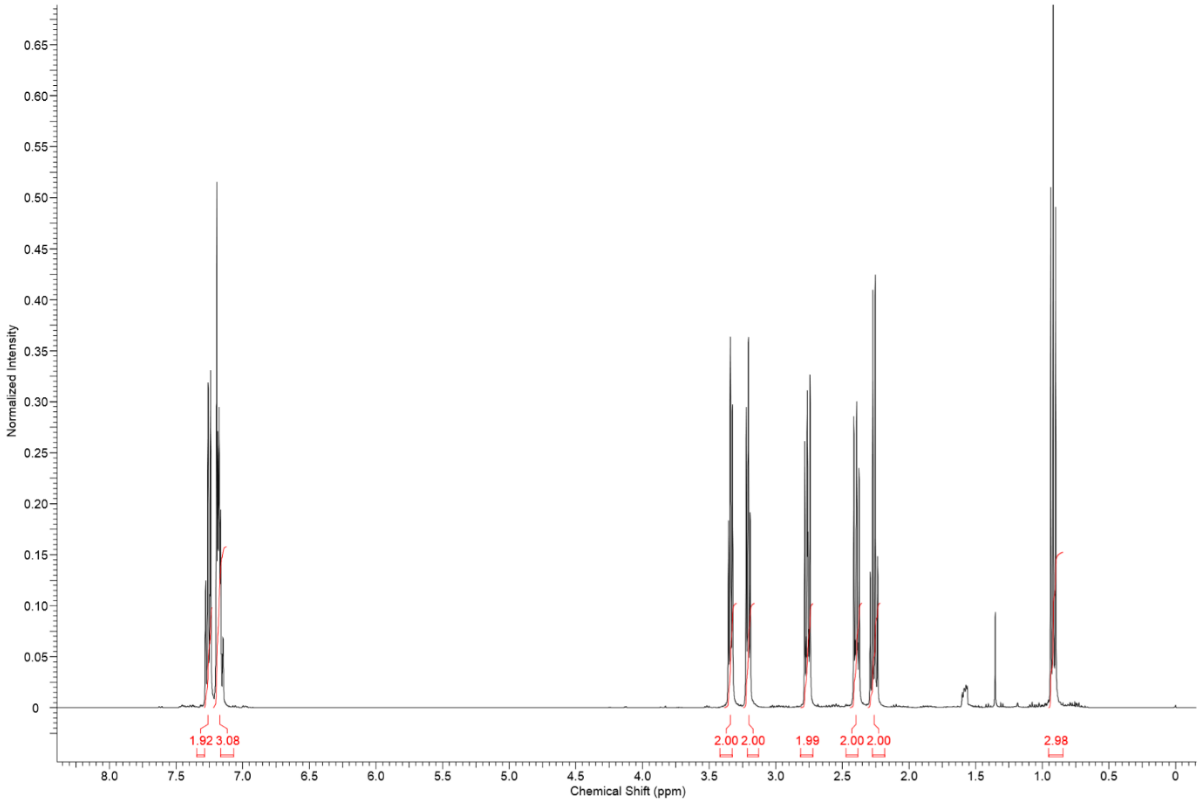

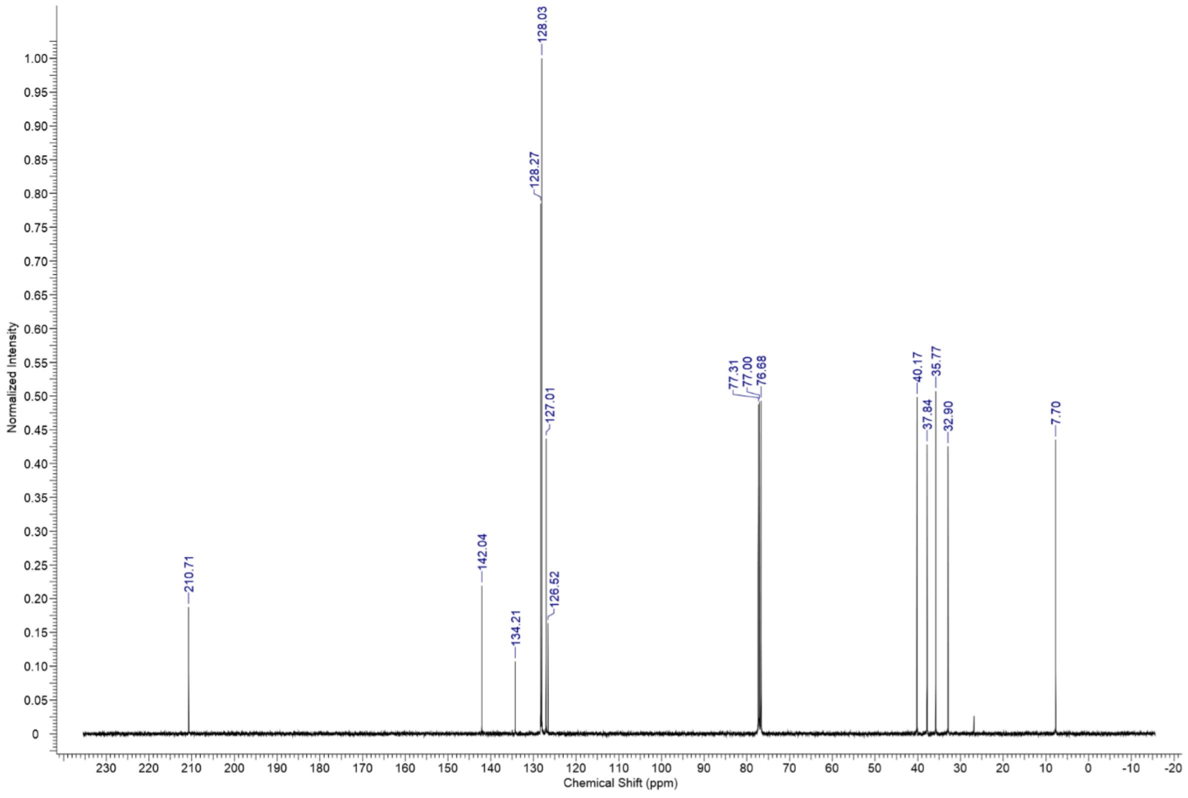

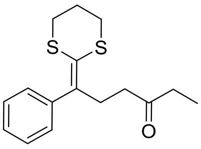

[5b] 6-(1,3-dithian-2-ylidene)-6-phenylhexan-3-one

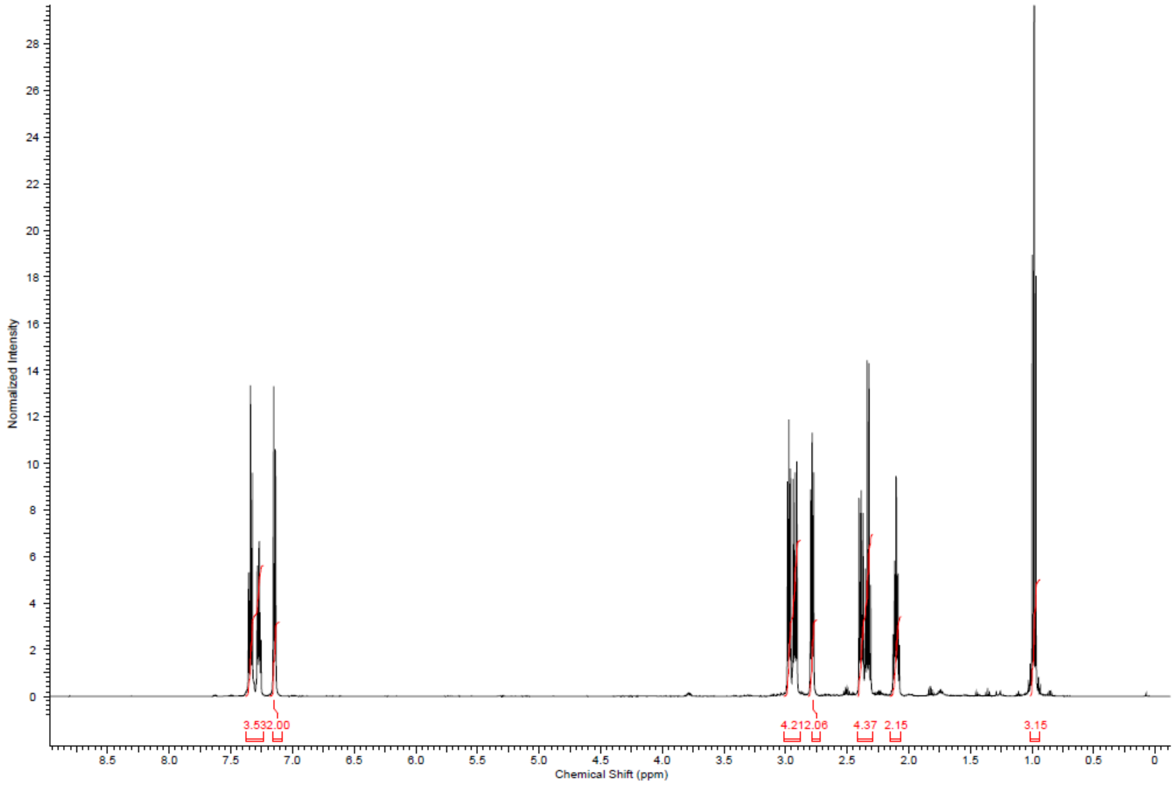

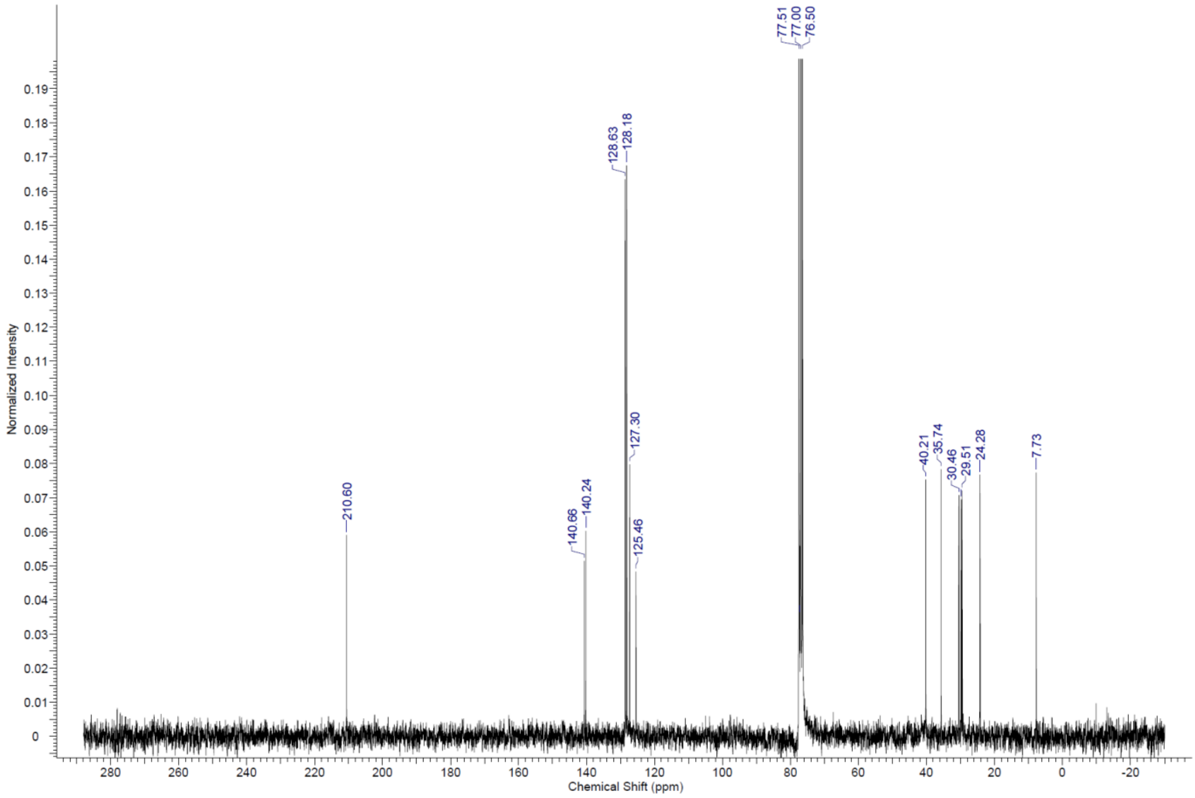

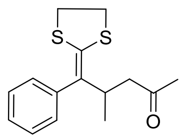

[4c] 5-(1,3-dithiolan-2-ylidene)-4-methyl-5-phenylpentan-2-one

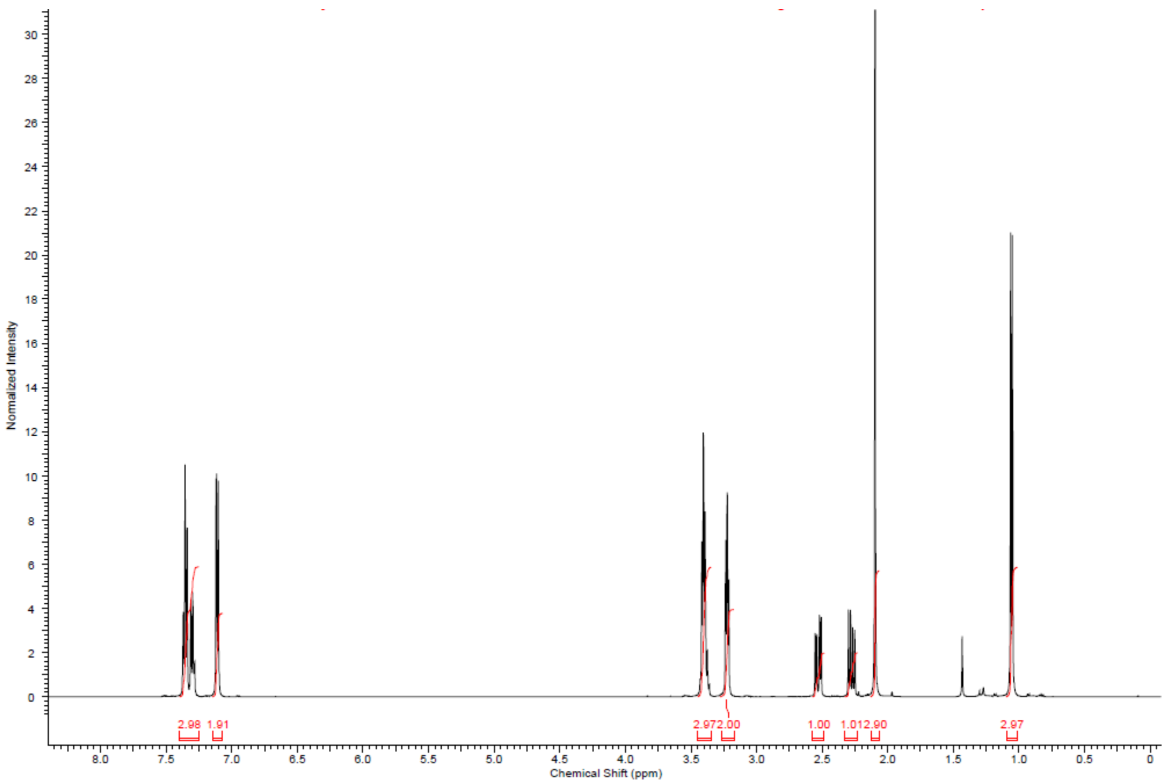

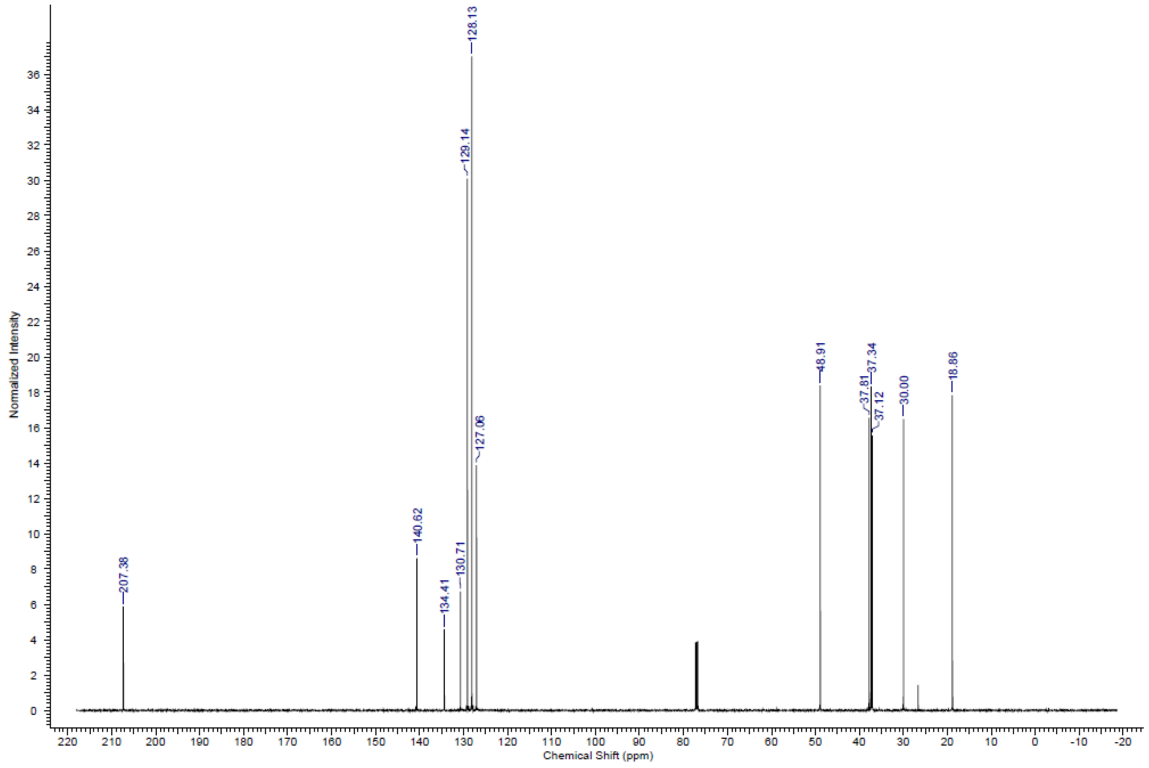

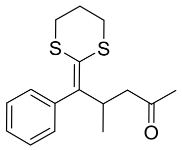

[5c] 5-(1,3-dithian-2-ylidene)-4-methyl-5-phenylpentan-2-one

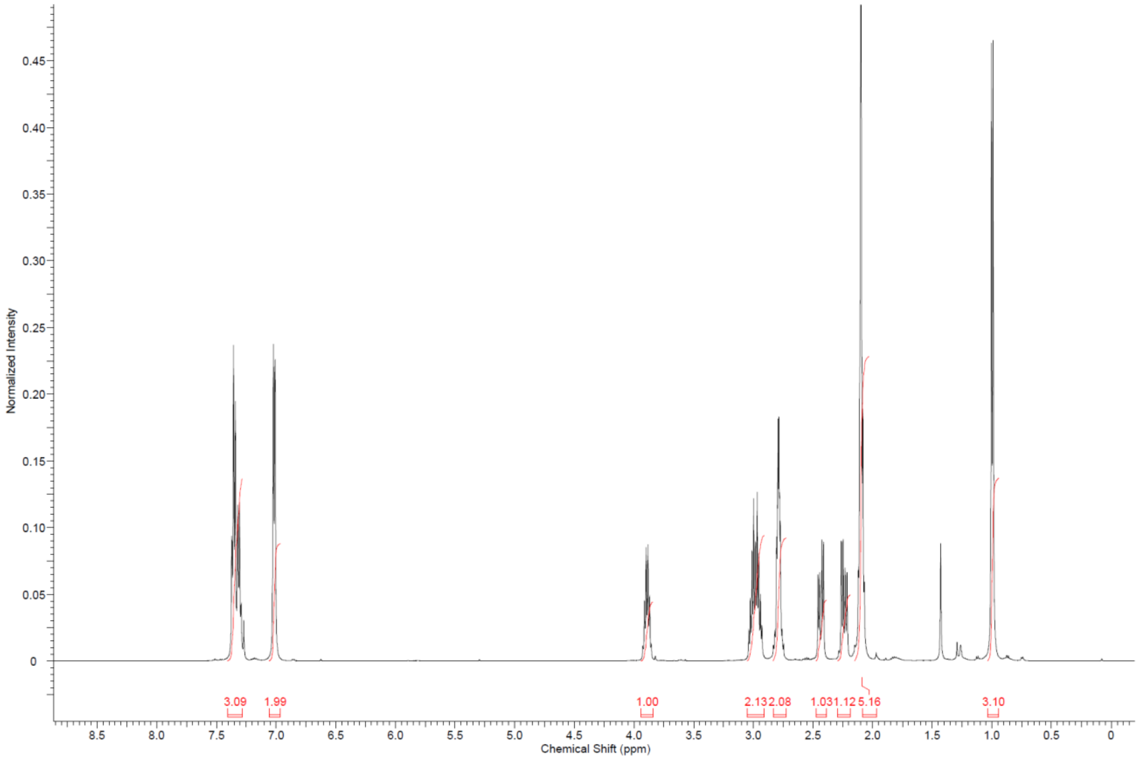

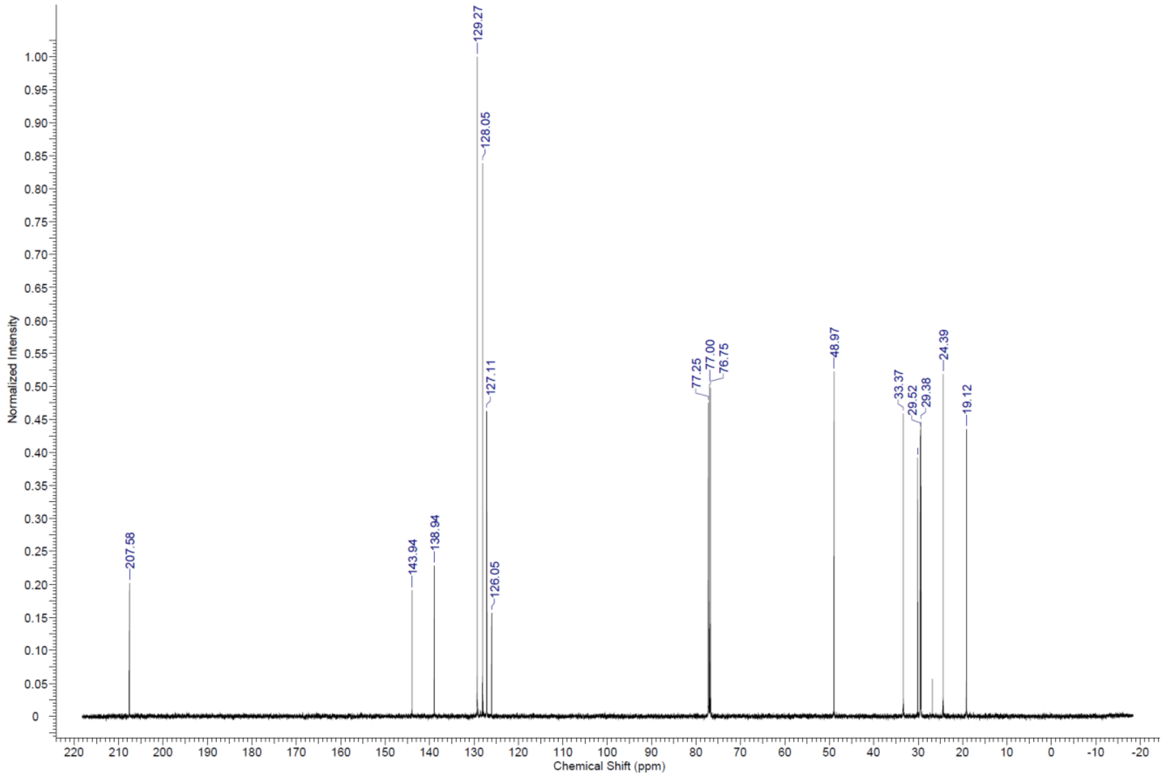

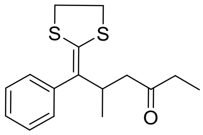

[4d] 6-(1,3-dithiolan-2-ylidene)-5-methyl-6-phenylhexan-3-one

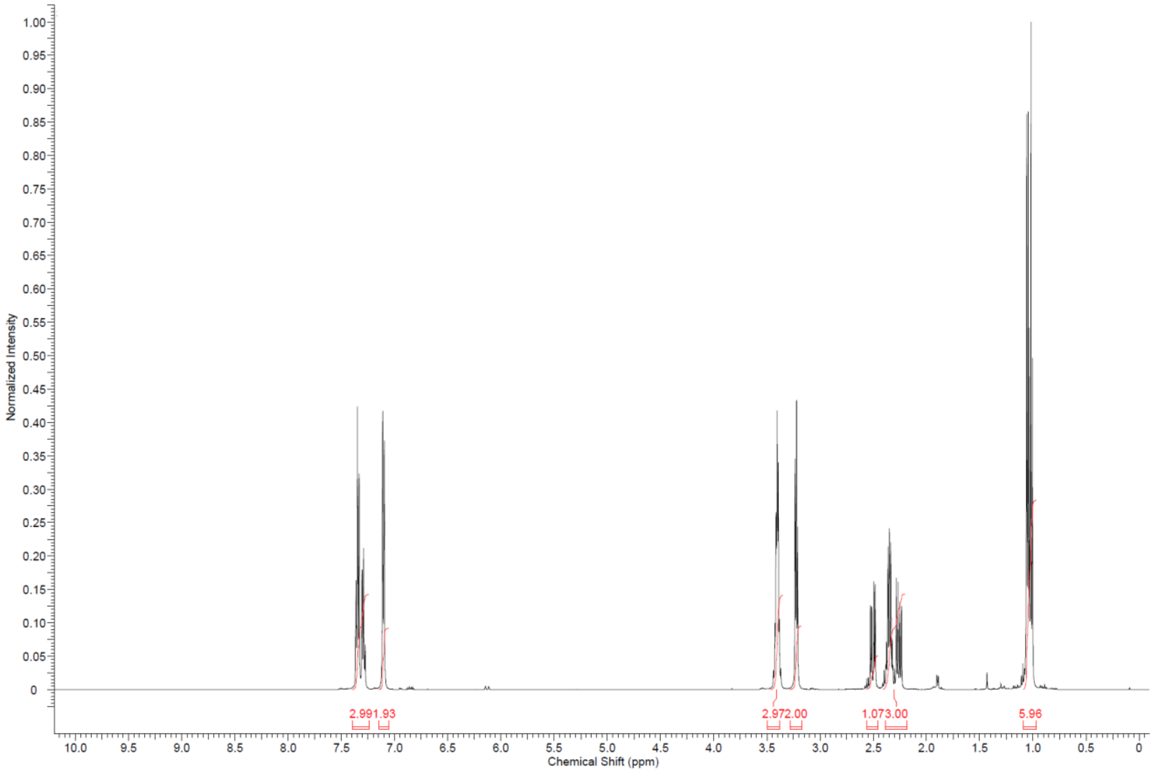

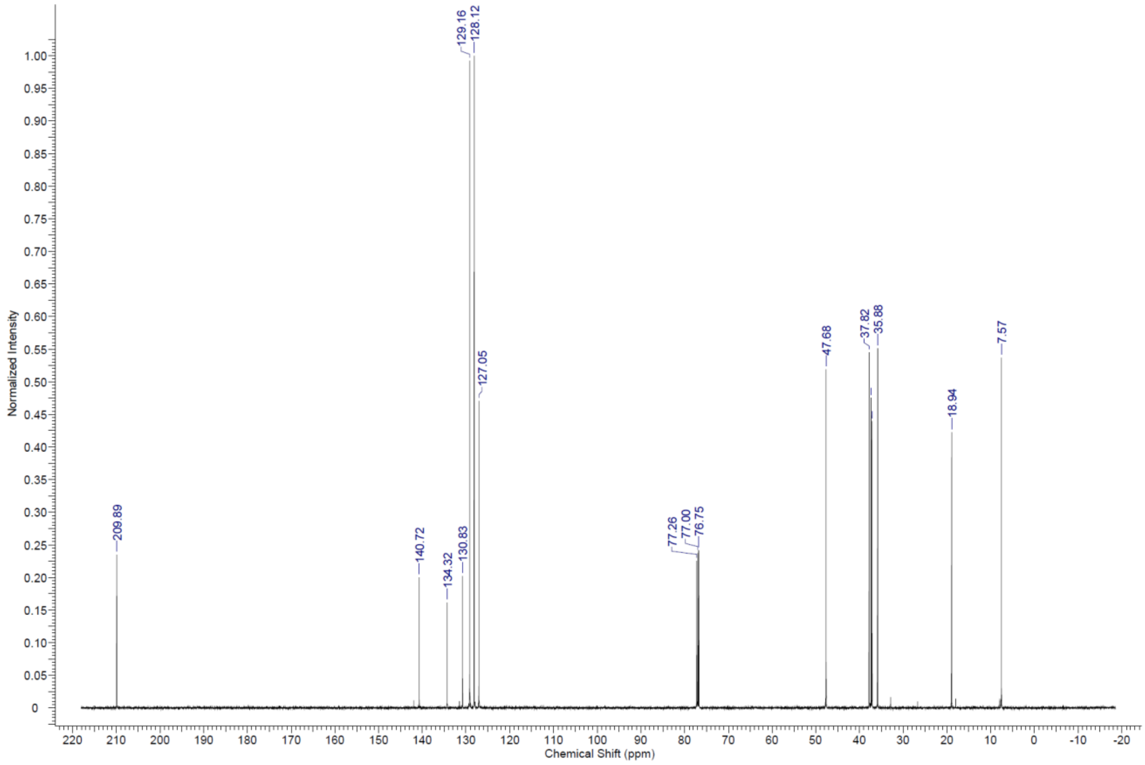

[5d] 6-(1,3-dithian-2-ylidene)-5-methyl-6-phenylhexan-3-one

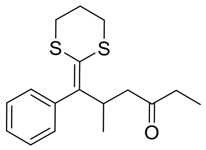

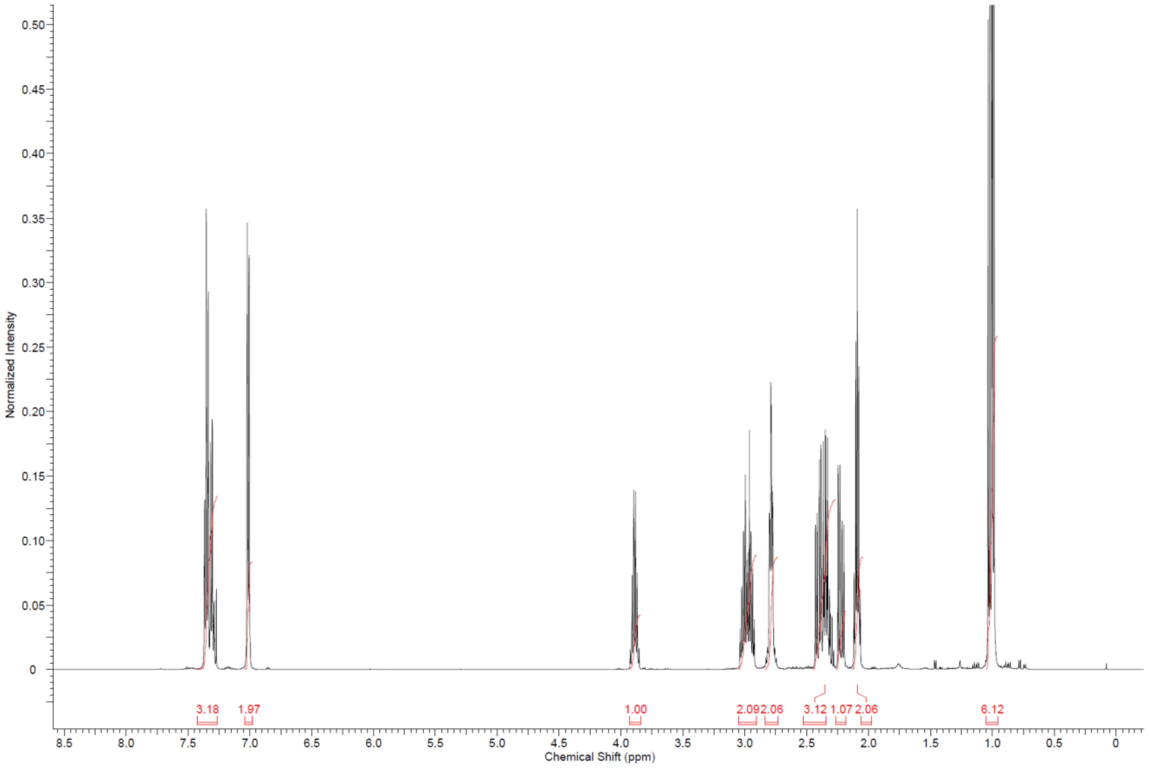

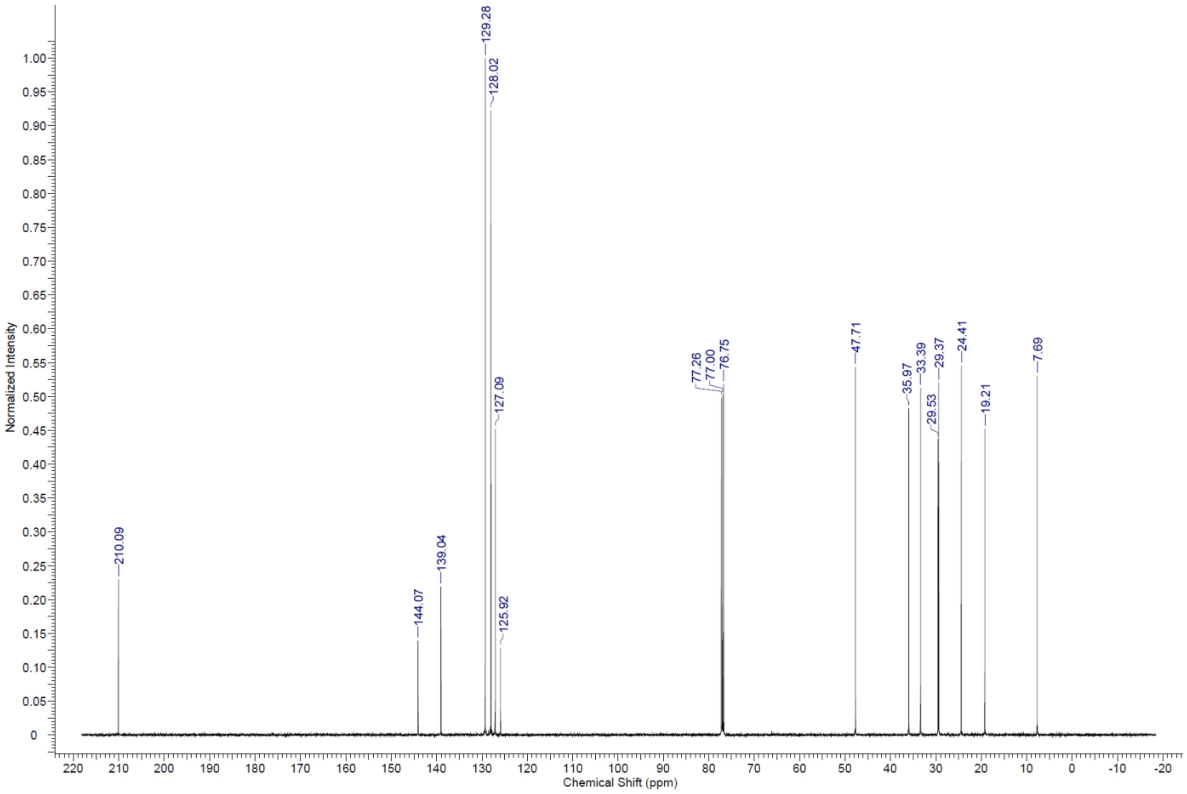

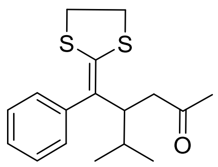

[4e] 4-((1,3-Dithiolan-2-ylidene)(phenyl)methyl)-5-methylhexan-2-one

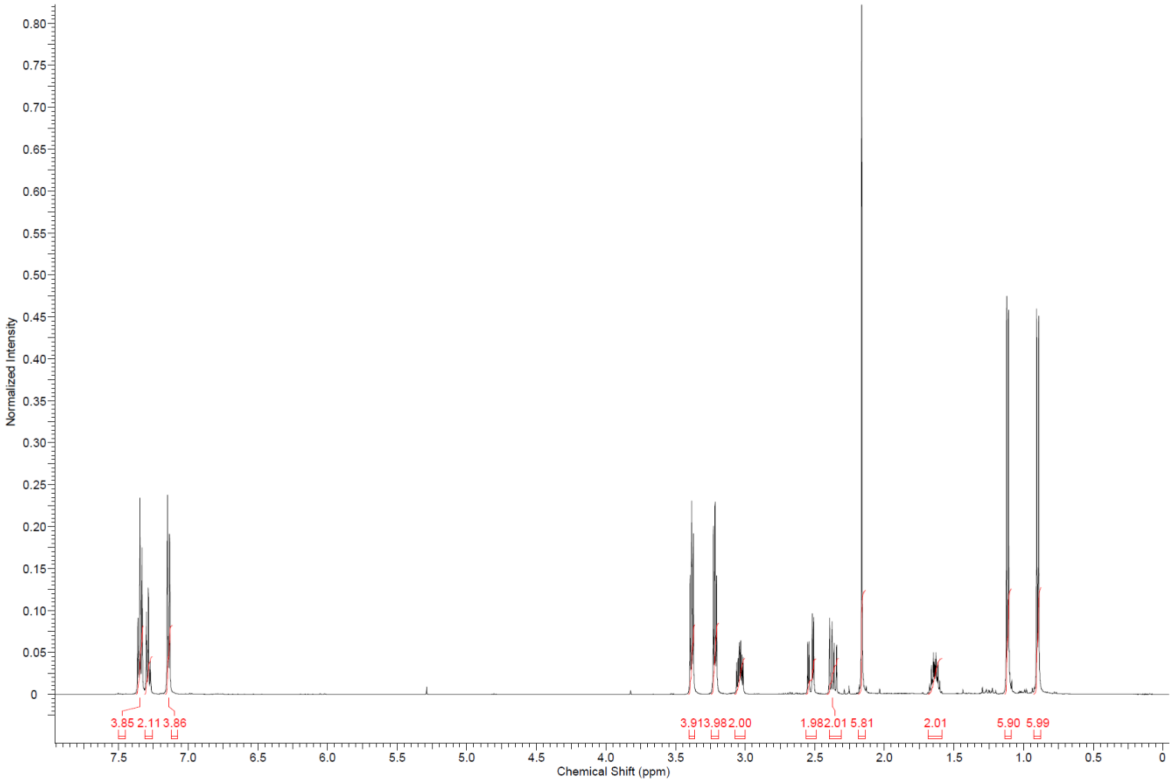

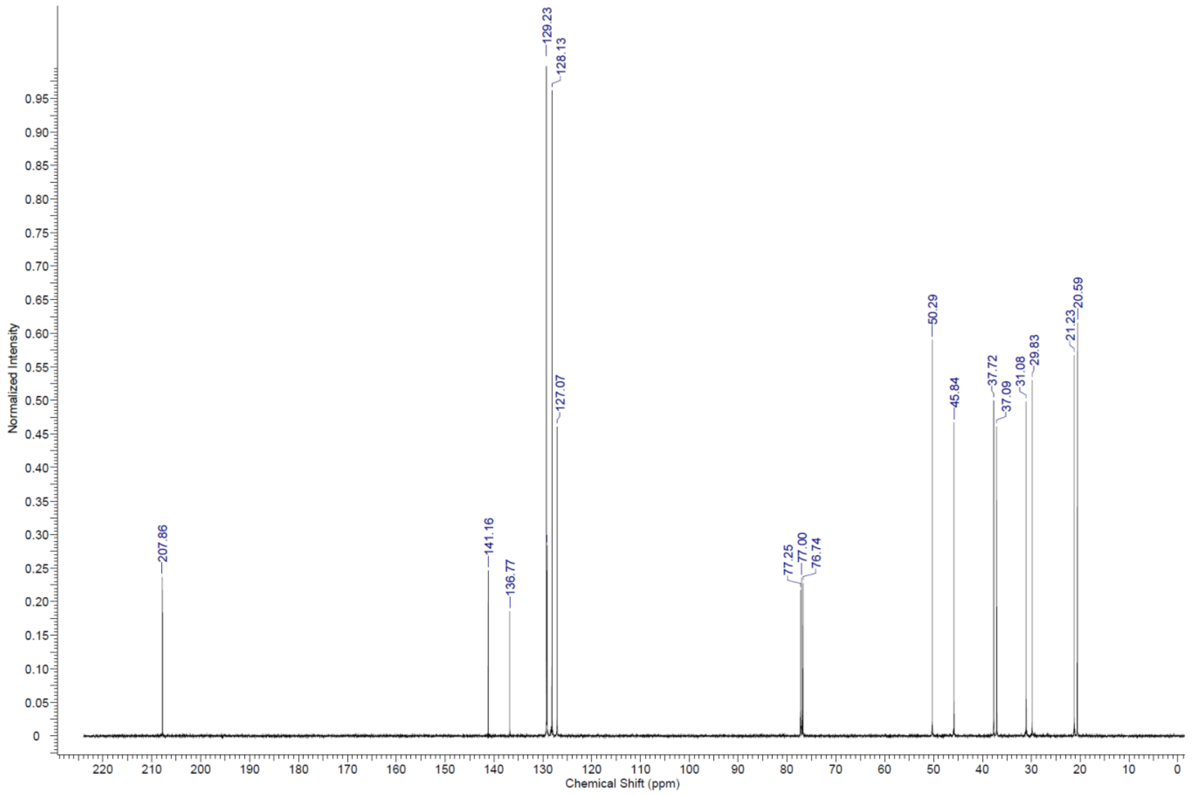

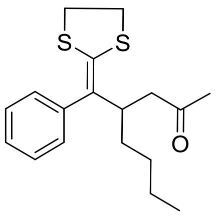

[4f] 4-((1,3-dithiolan-2-ylidene)(phenyl)methyl)octan-2-one

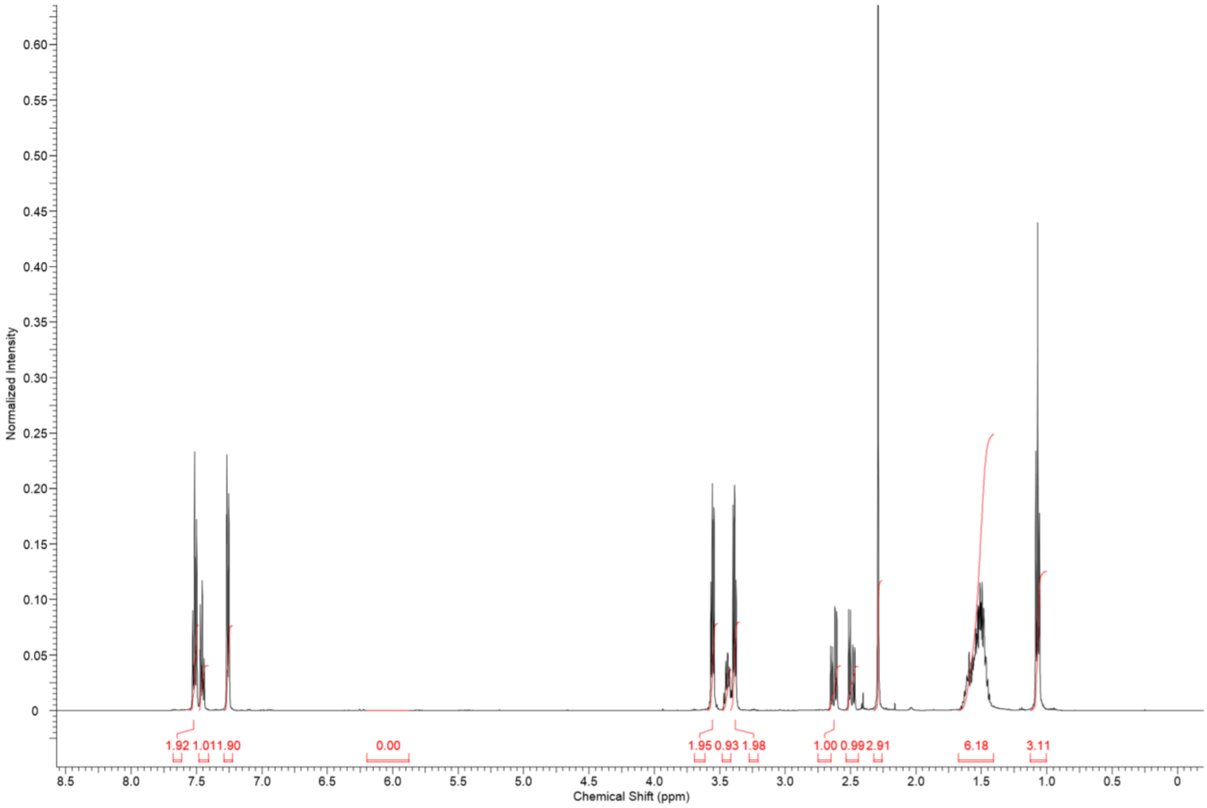

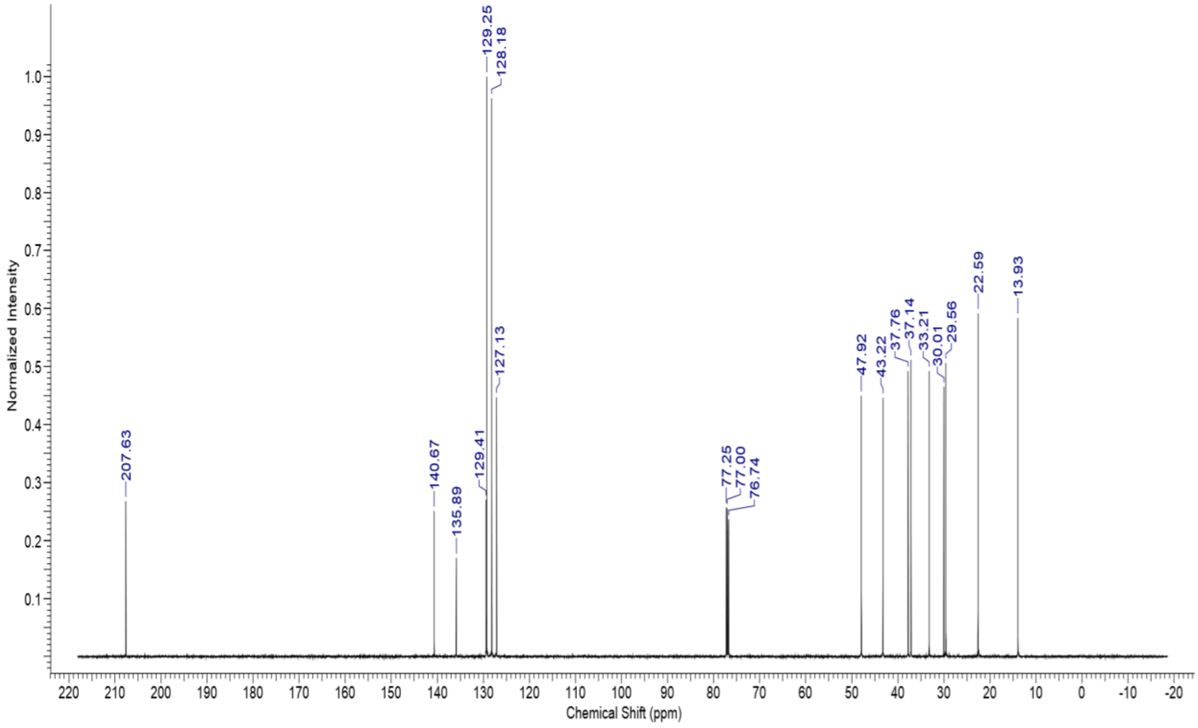

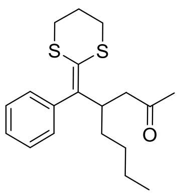

[5f] 4-((1,3-dithian-2-ylidene)(phenyl)methyl)octan-2-one

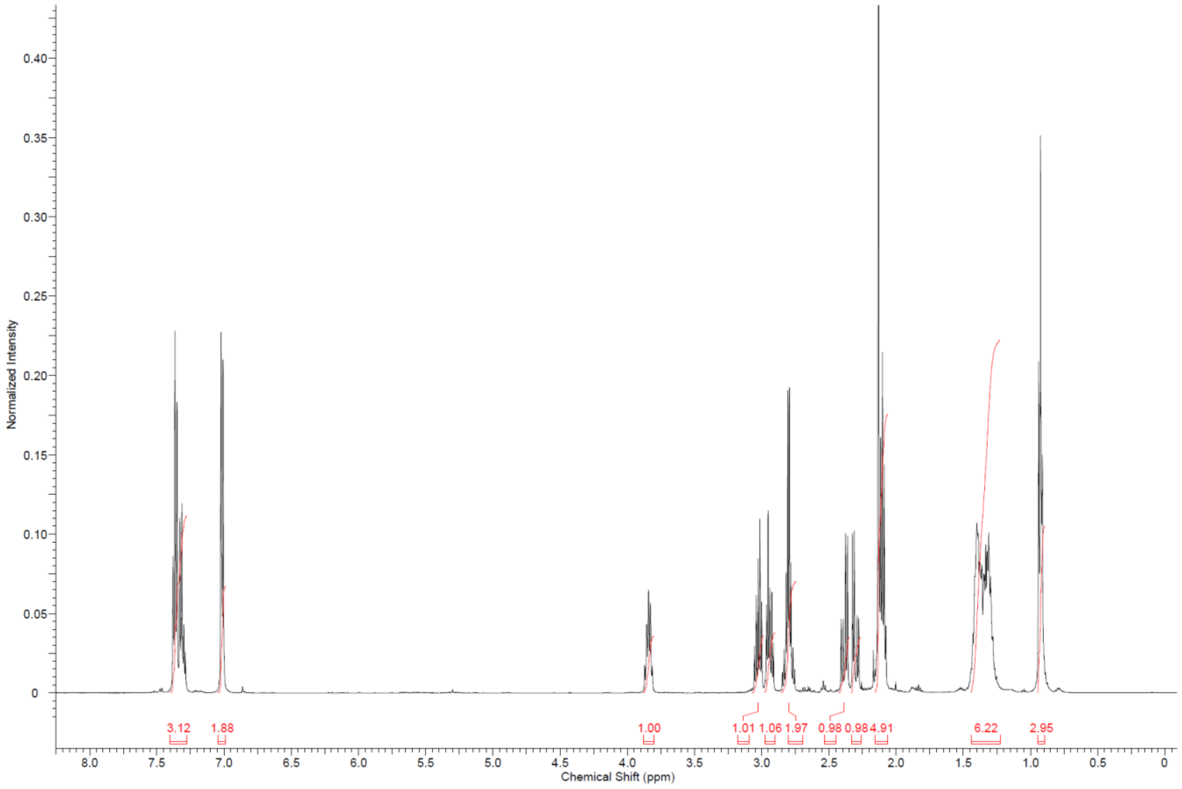

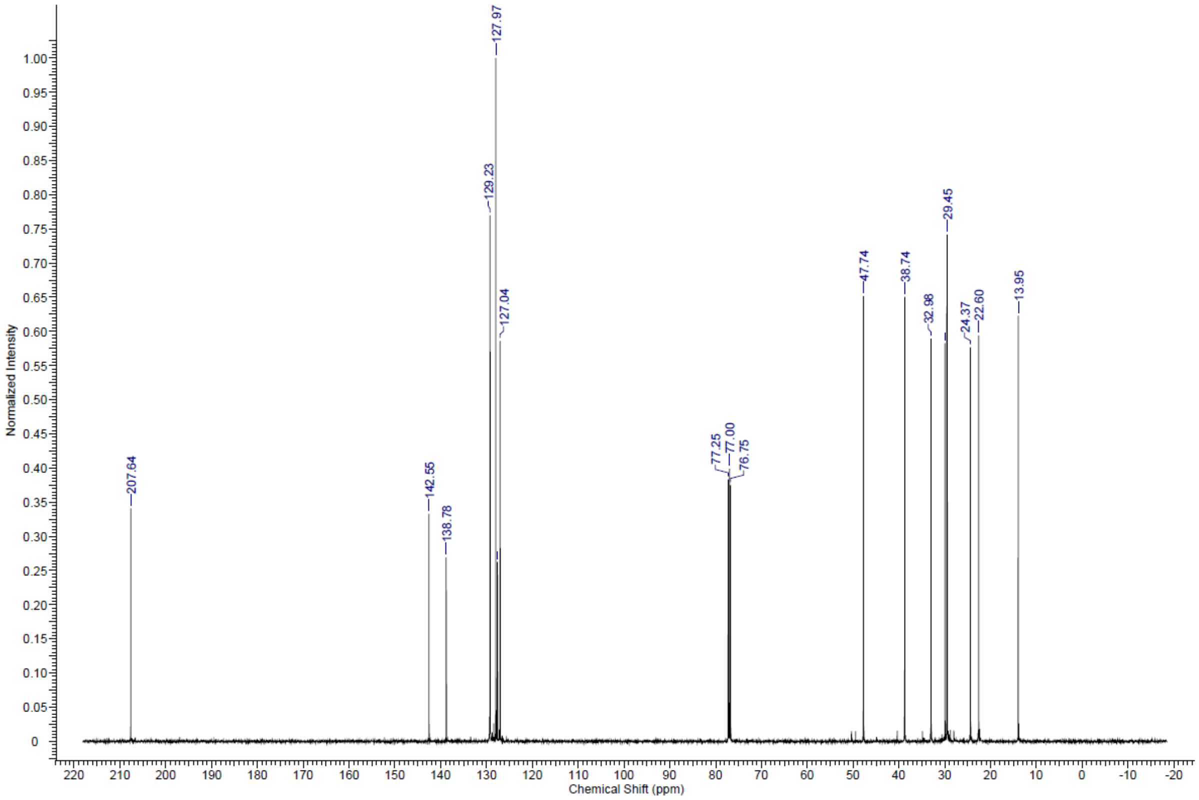

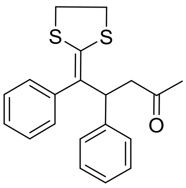

[4g] 5-(1,3-dithiolan-2-ylidene)-4,5-diphenylpentan-2-one

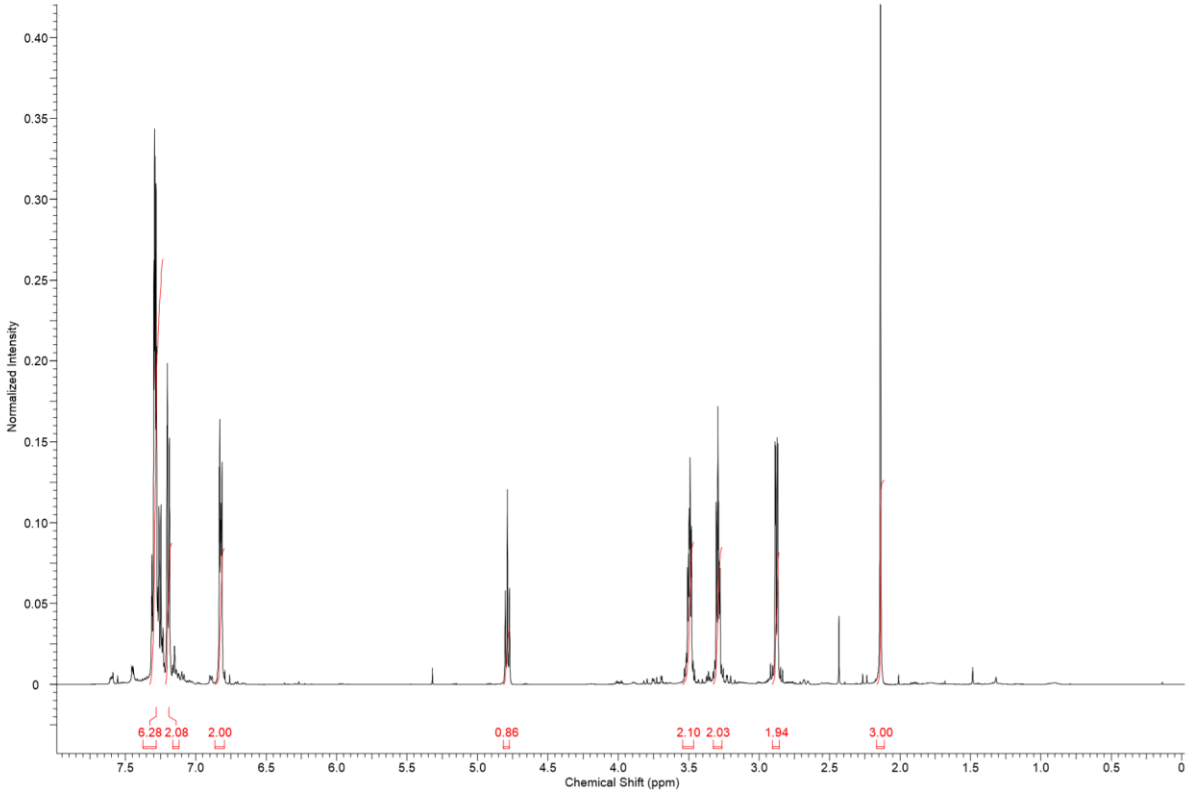

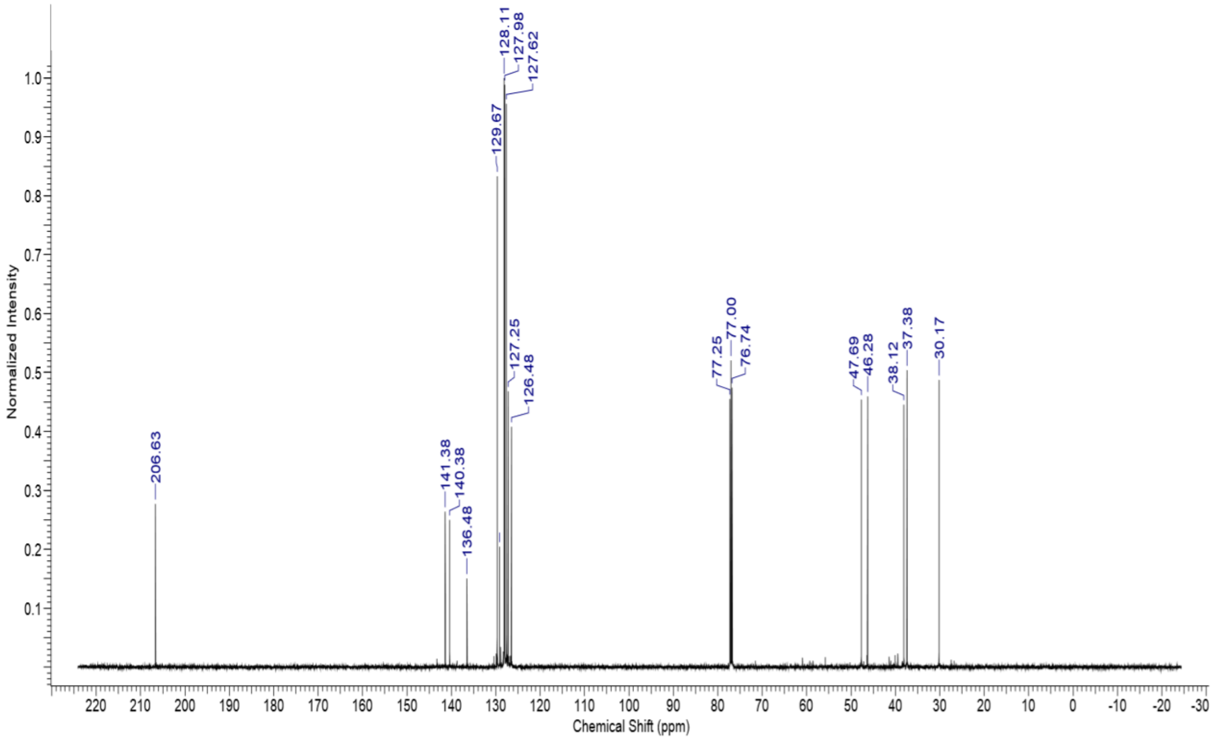

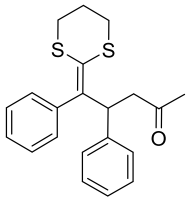

[5g] 5-(1,3-Dithian-2-ylidene)-4,5-diphenylpentan-2-one

.

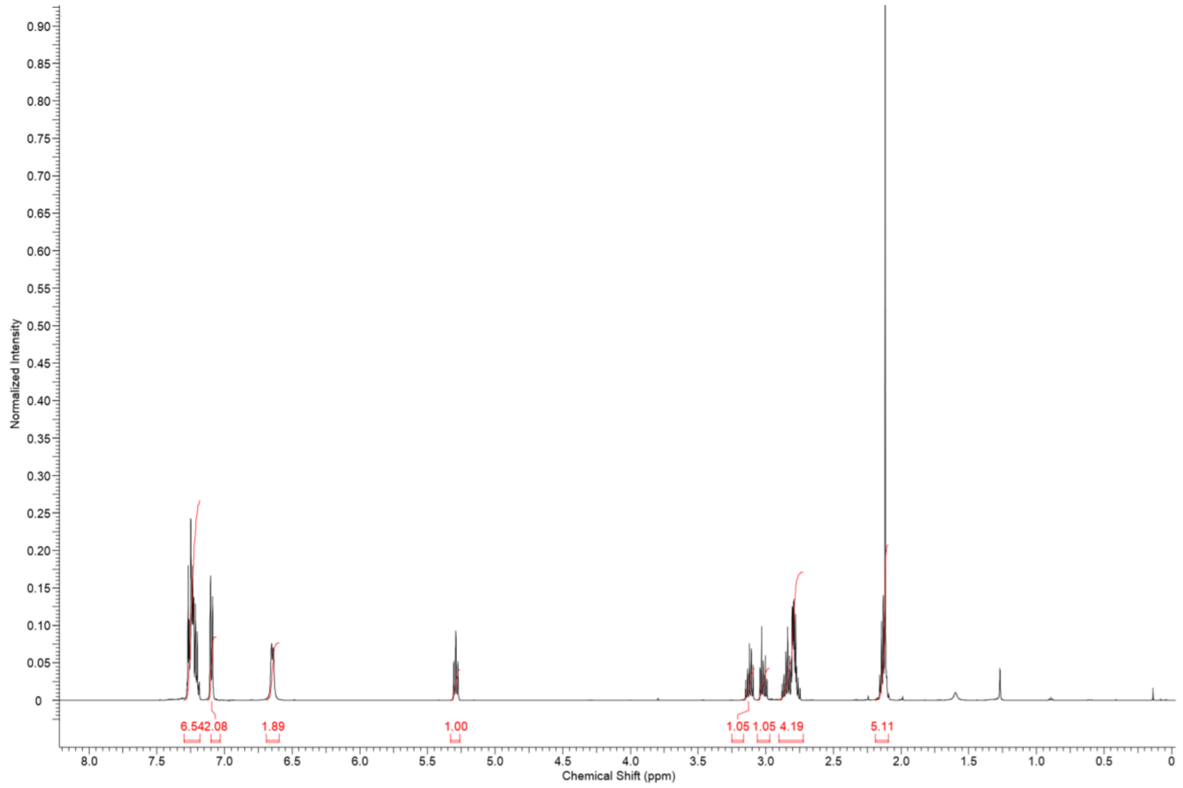

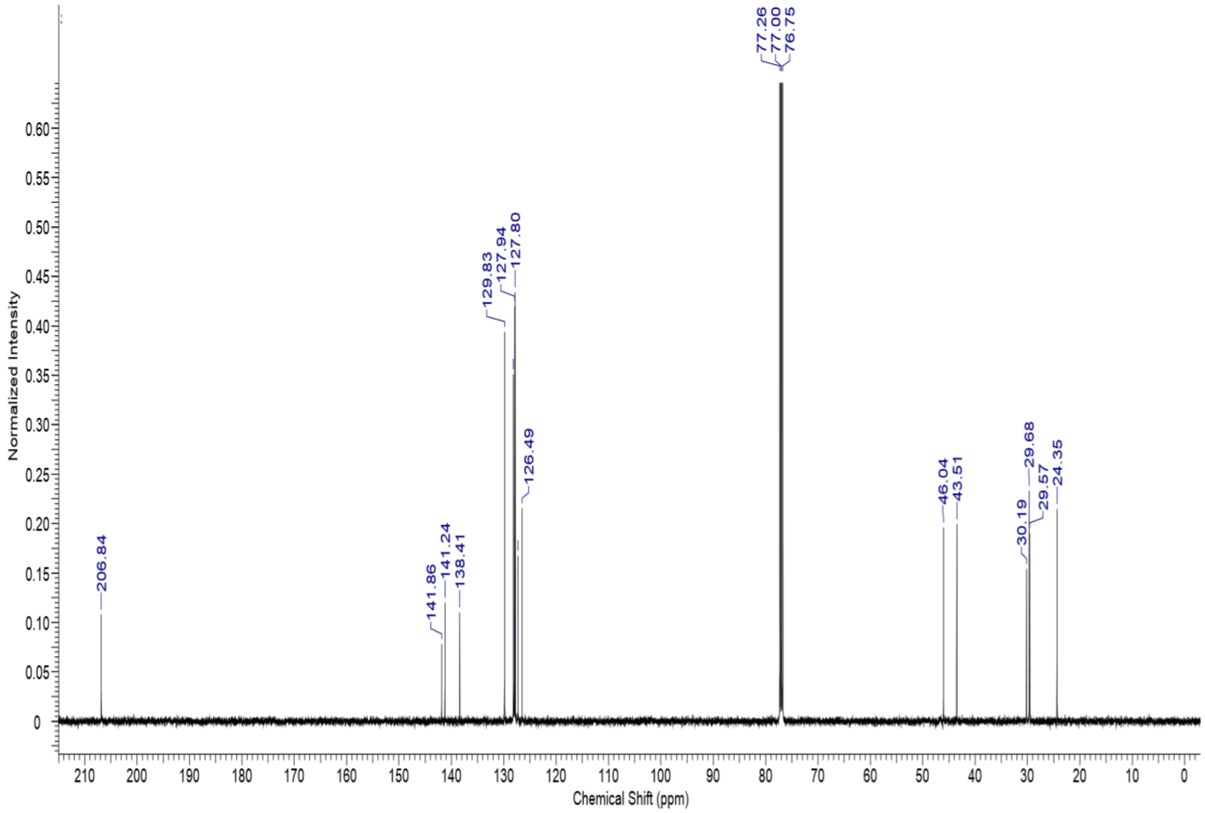

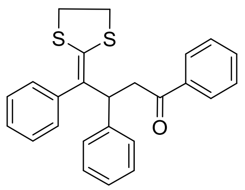

[4h] 4-(1,3-Dithiolan-2-ylidene)-1,3,4-triphenylbutan-1-one

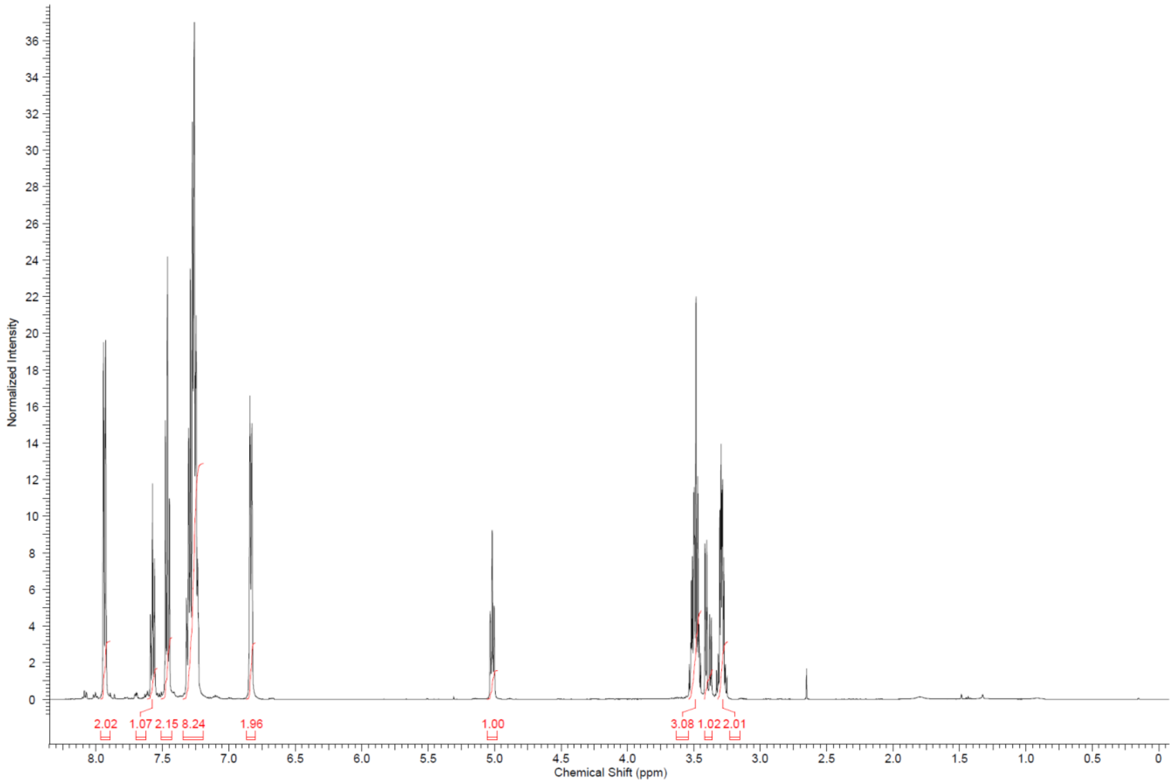

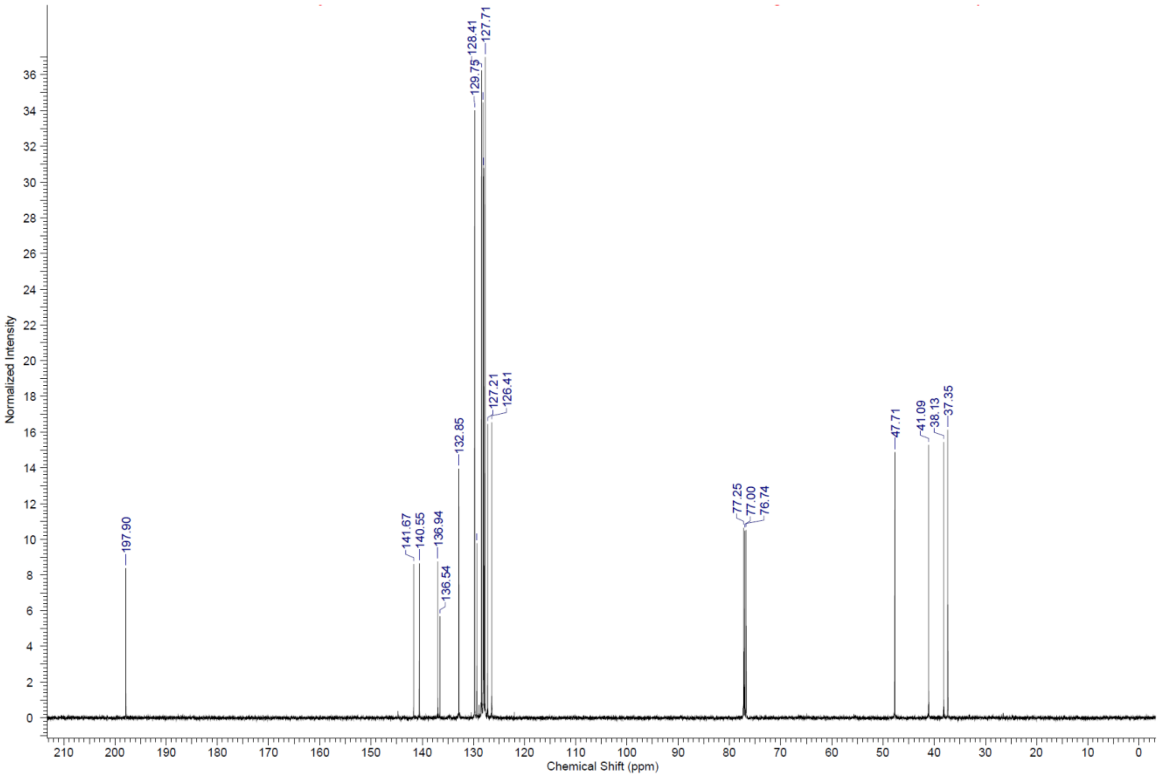

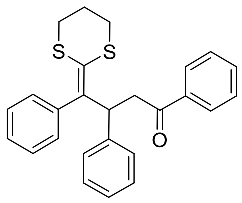

[5h] 4-(1,3-Dithian-2-ylidene)-1,3,4-triphenylbutan-1-one, impure (80%)

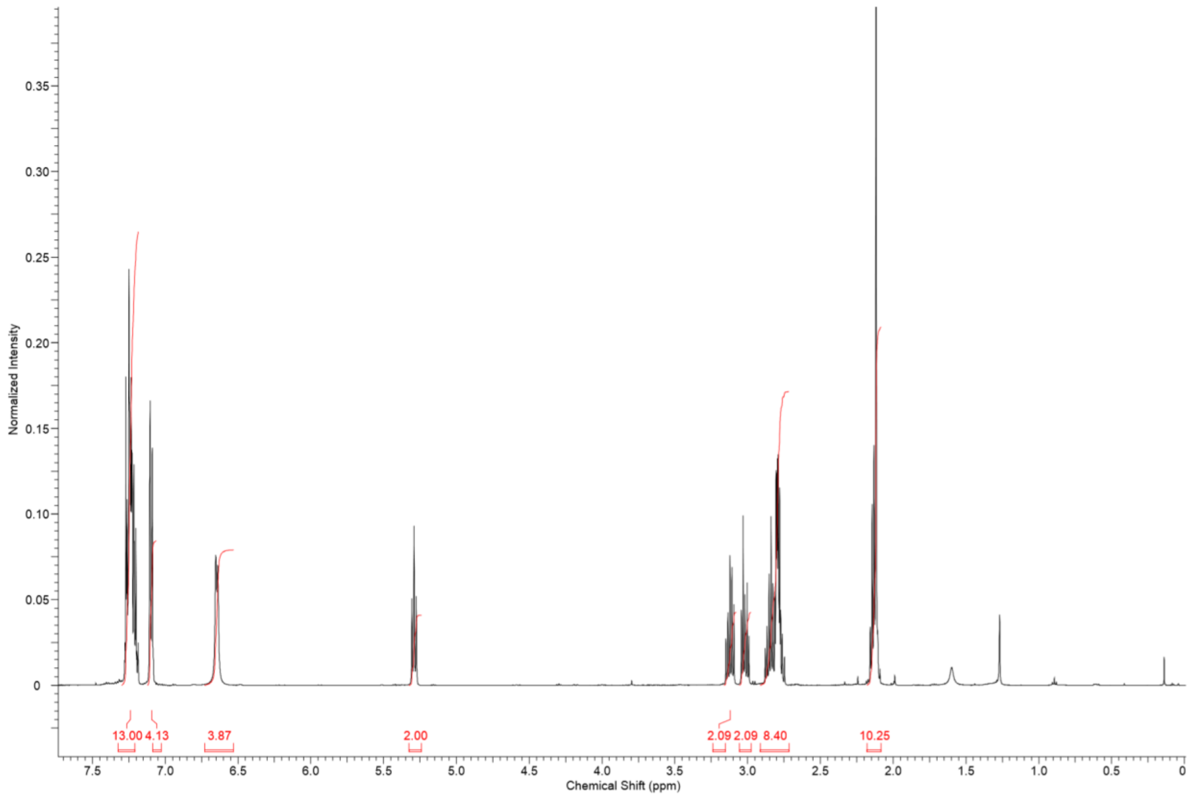

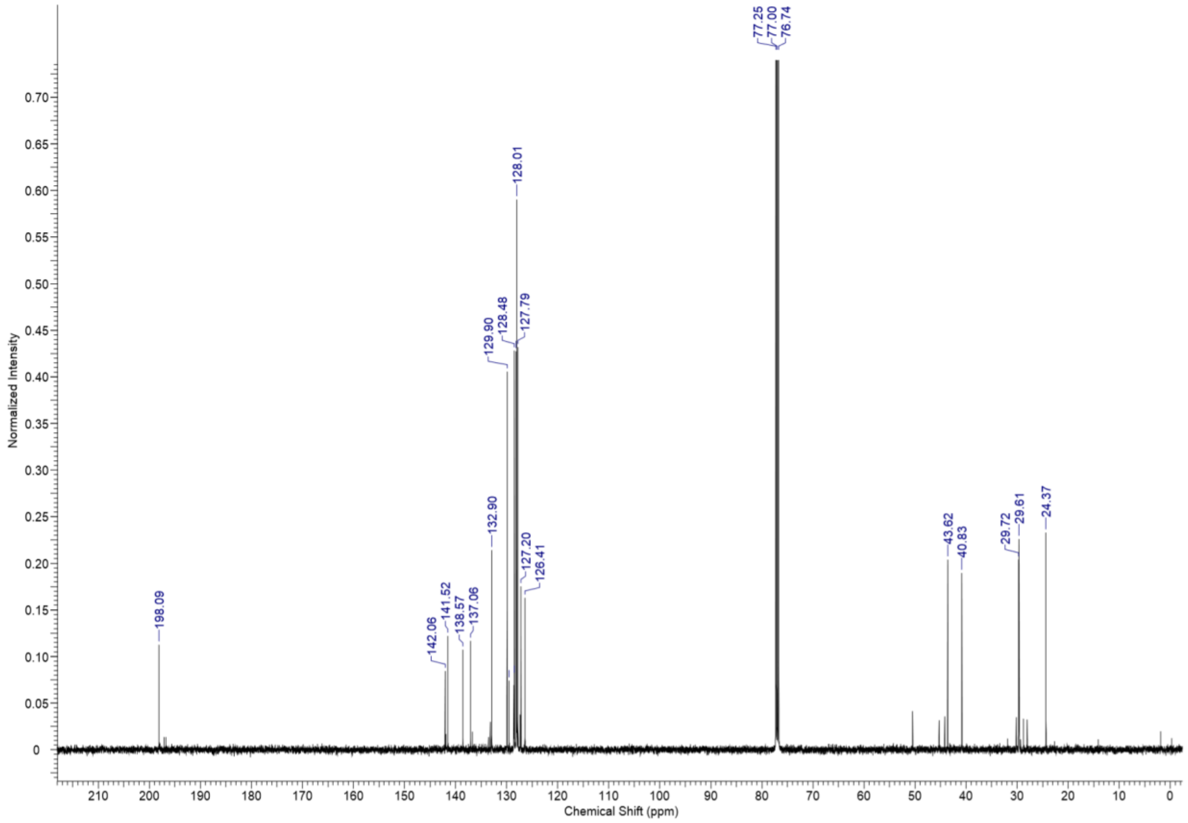

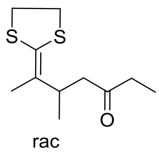

[4i] 6-(1,3-dithiolan-2-ylidene)-5-methylheptan-3-one (rac)

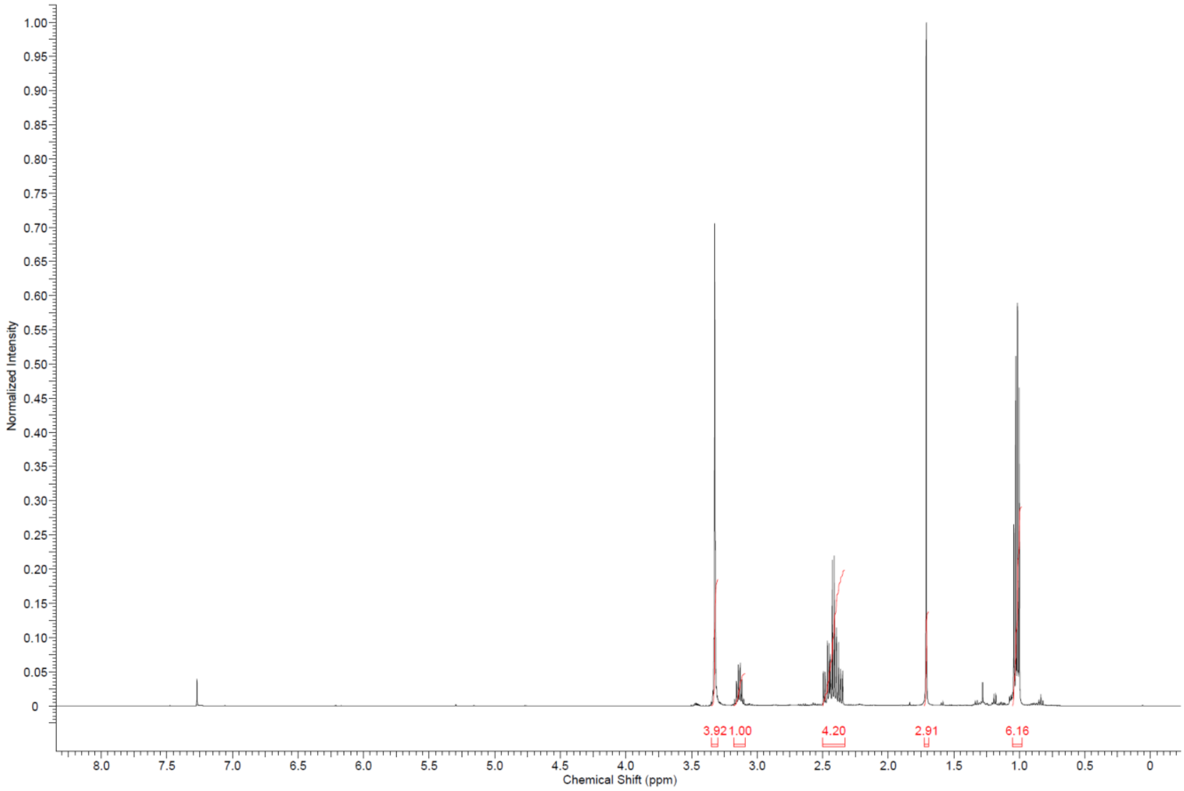

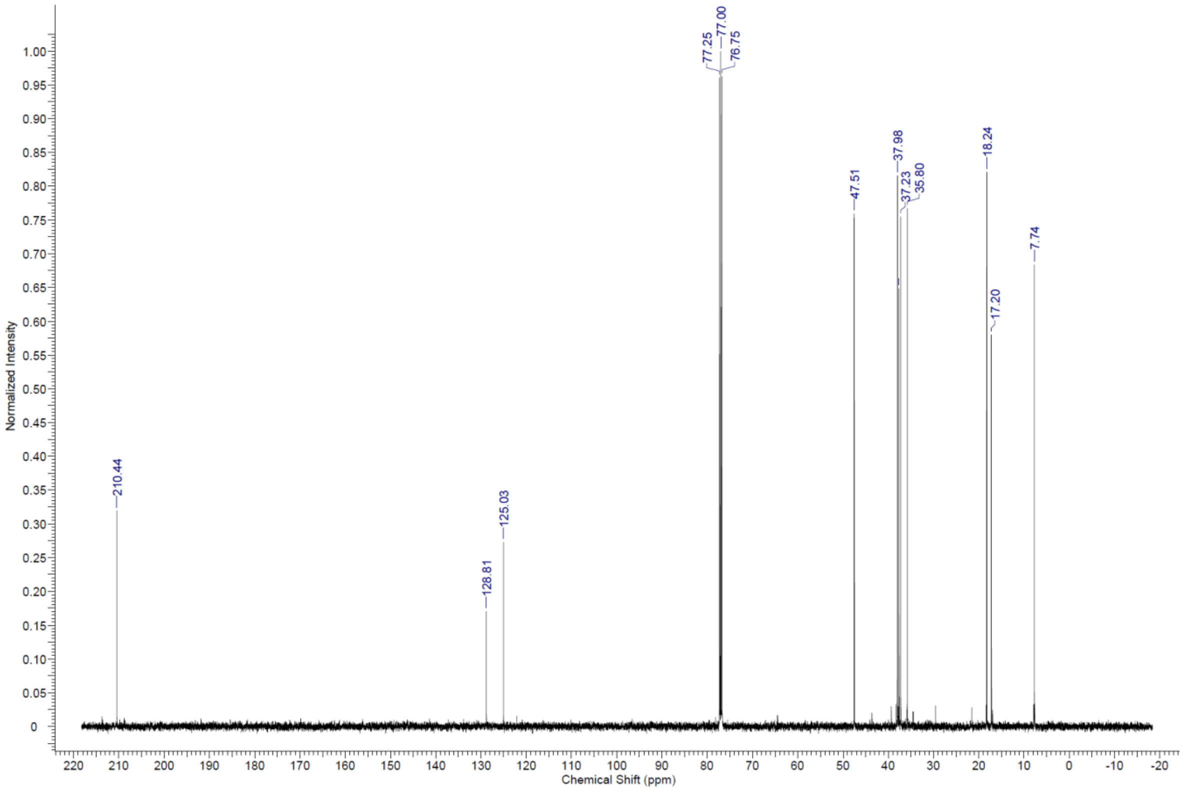

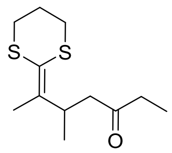

rac

[5i] 6-(1,3-dithian-2-ylidene)-5-methylheptan-3-one

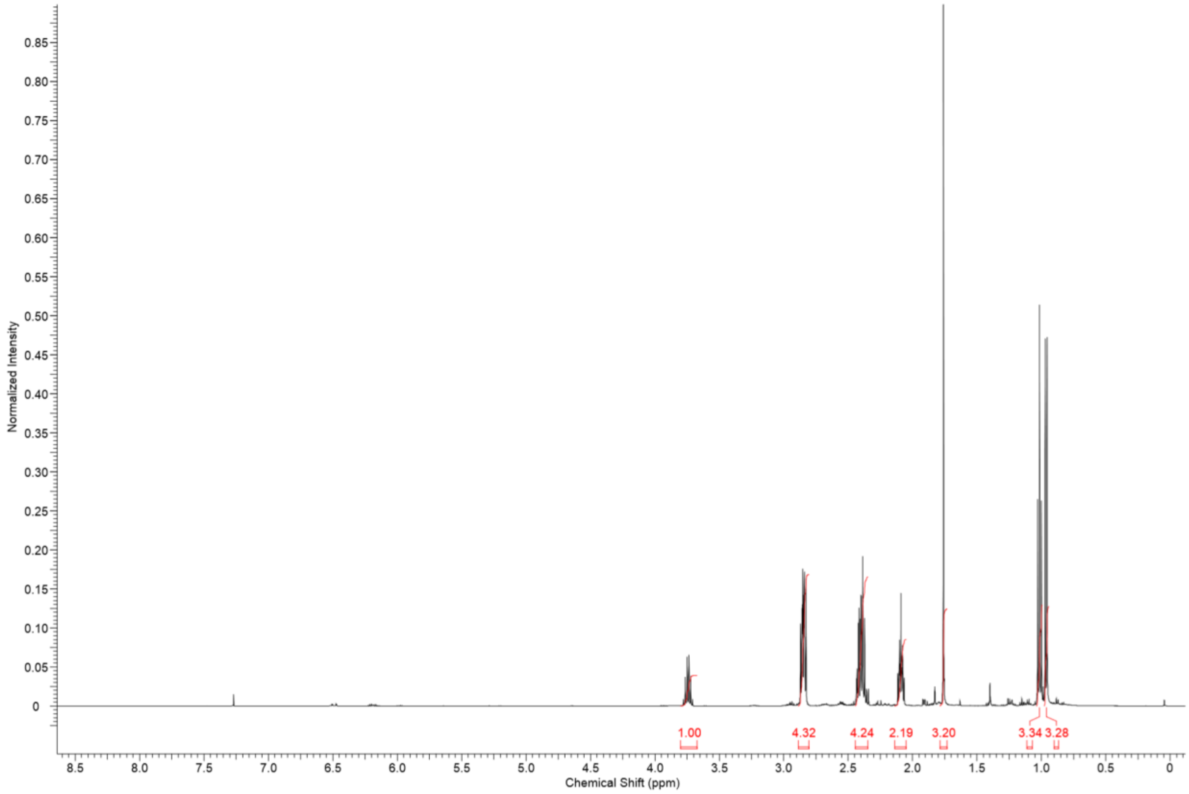

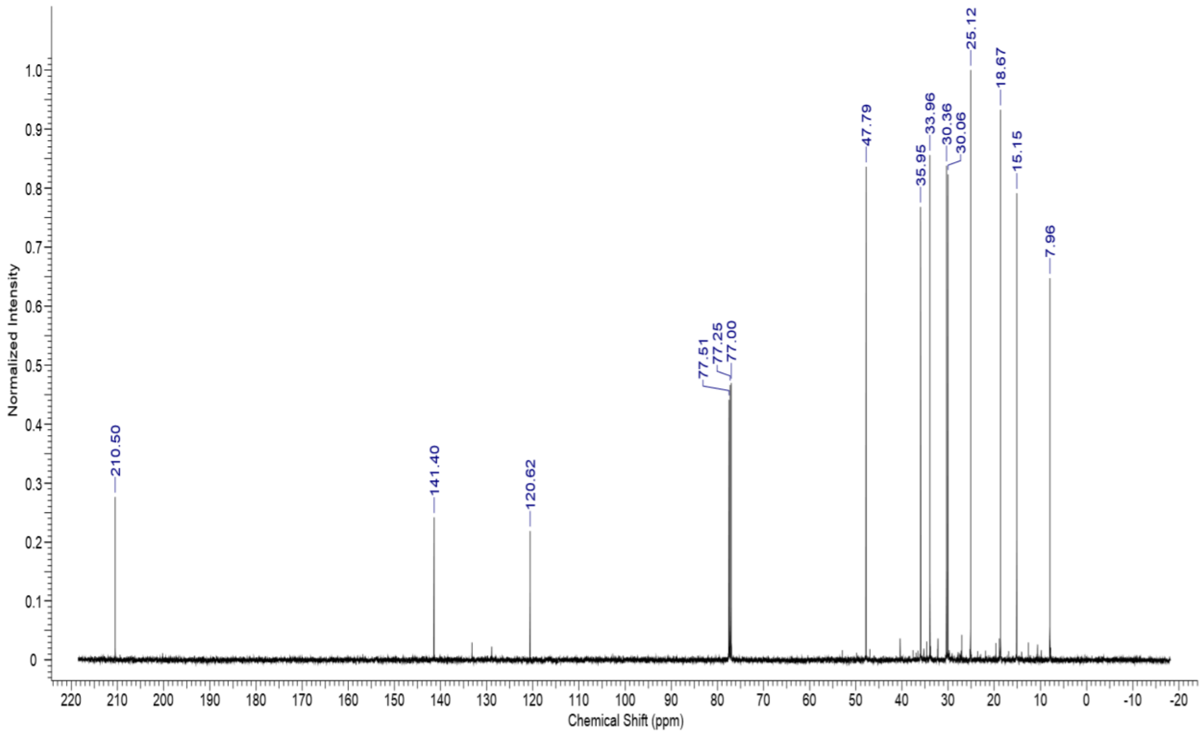

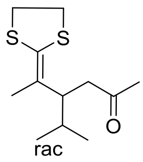

[4j] 4-(1-(1,3-dithiolan-2-ylidene)ethyl)-5-methylhexan-2-one

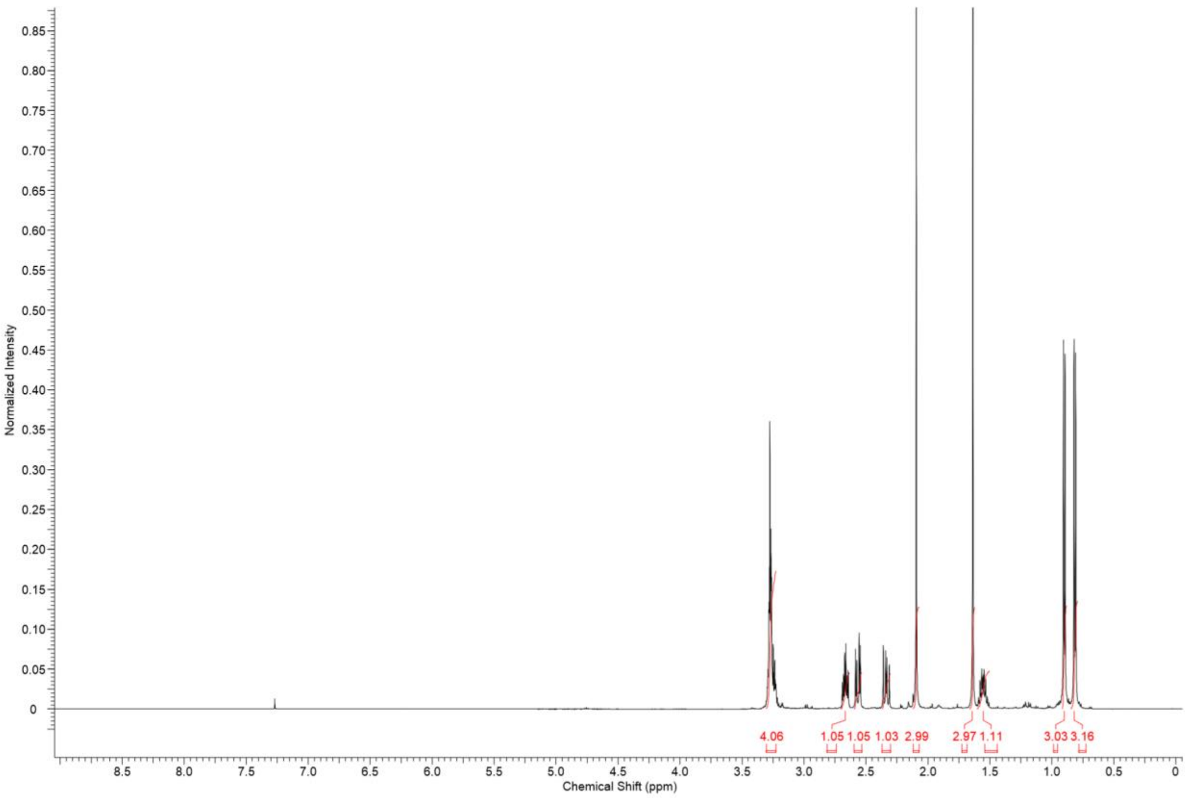

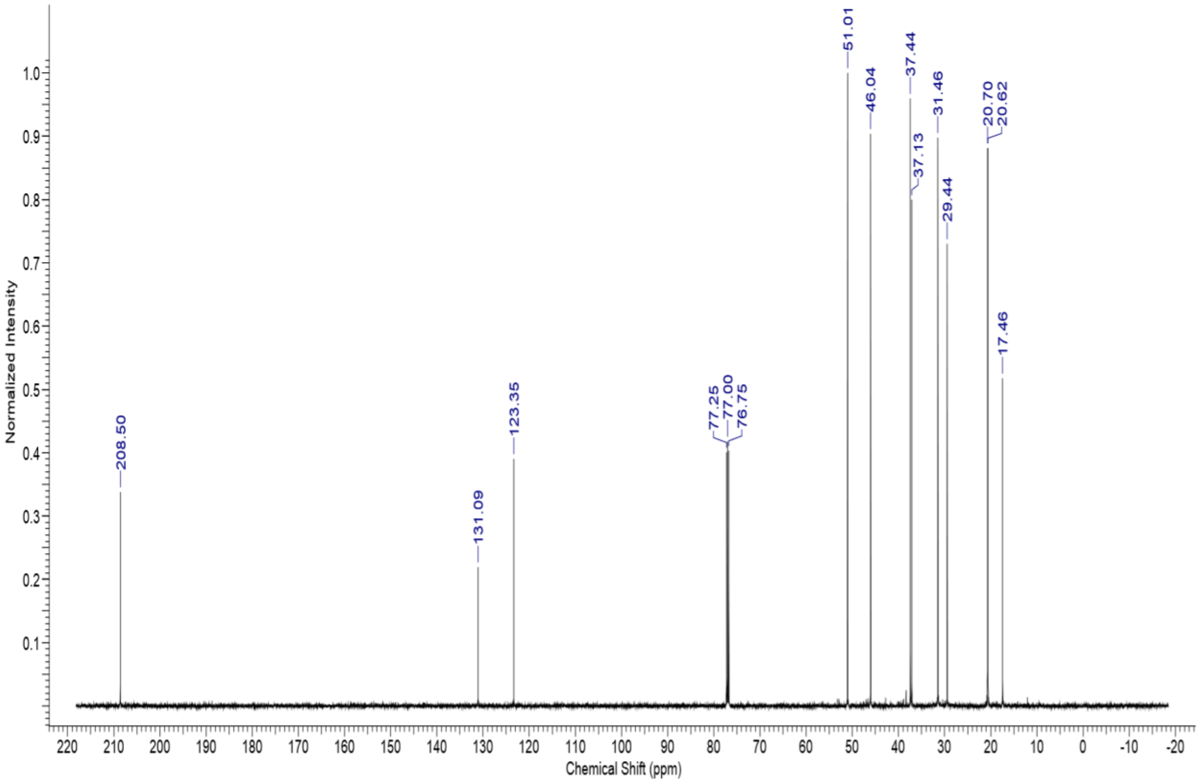

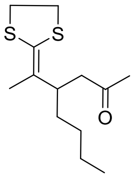

[4k] 4-(1-(1,3-dithiolan-2-ylidene)ethyl)octan-2-one

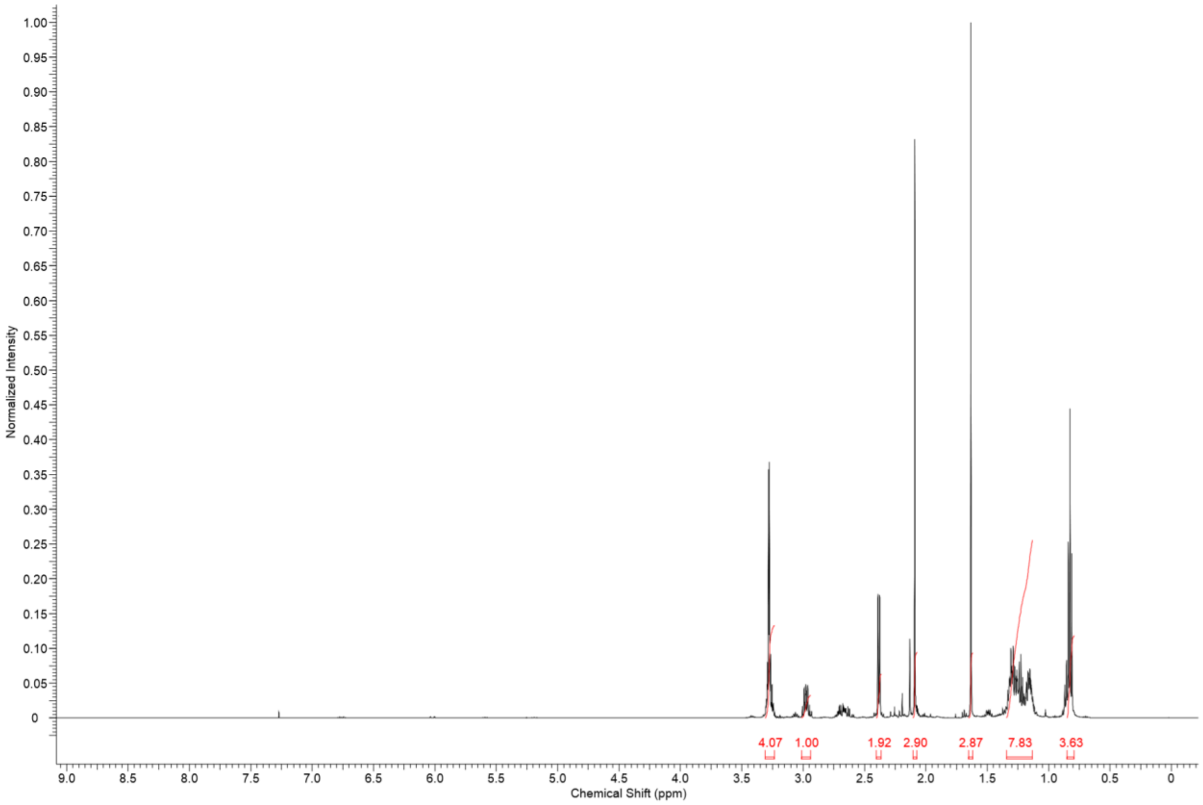

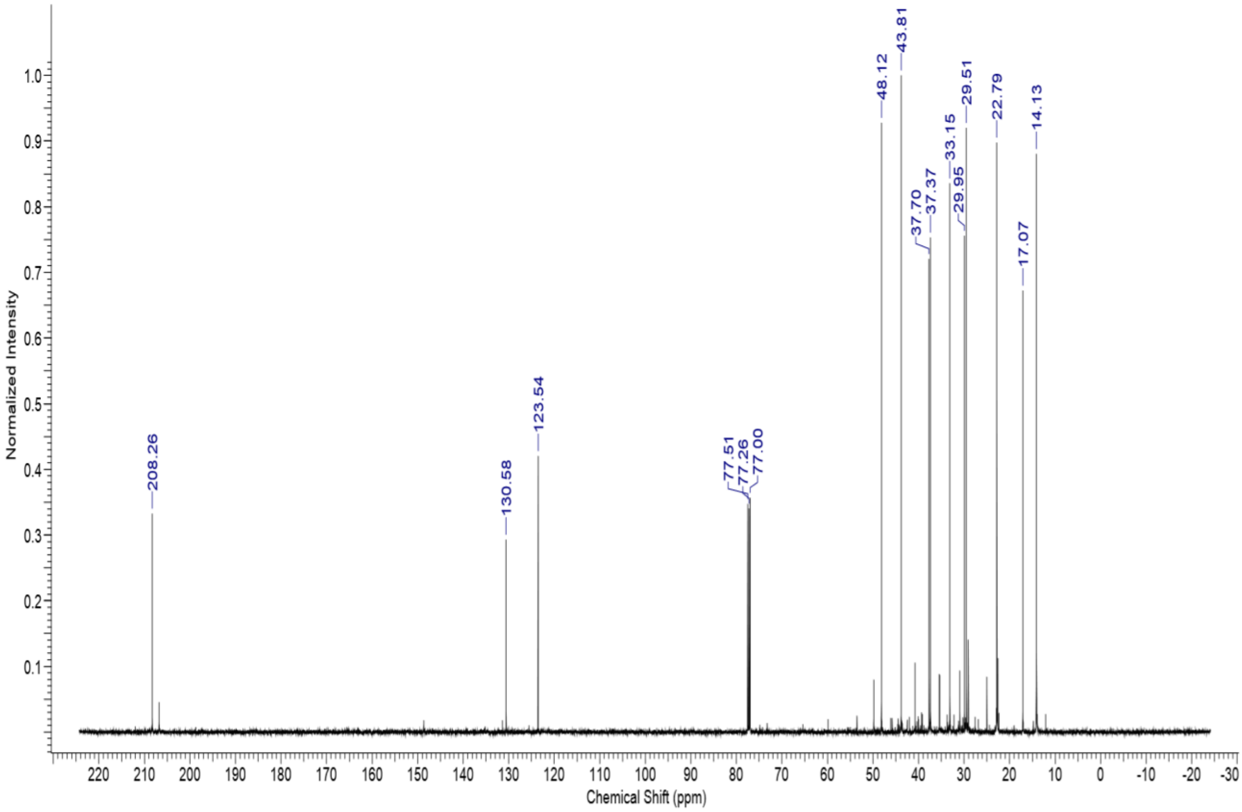

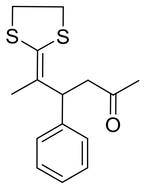

[4l] 5-(1,3-dithiolan-2-ylidene)-4-phenylhexan-2-one

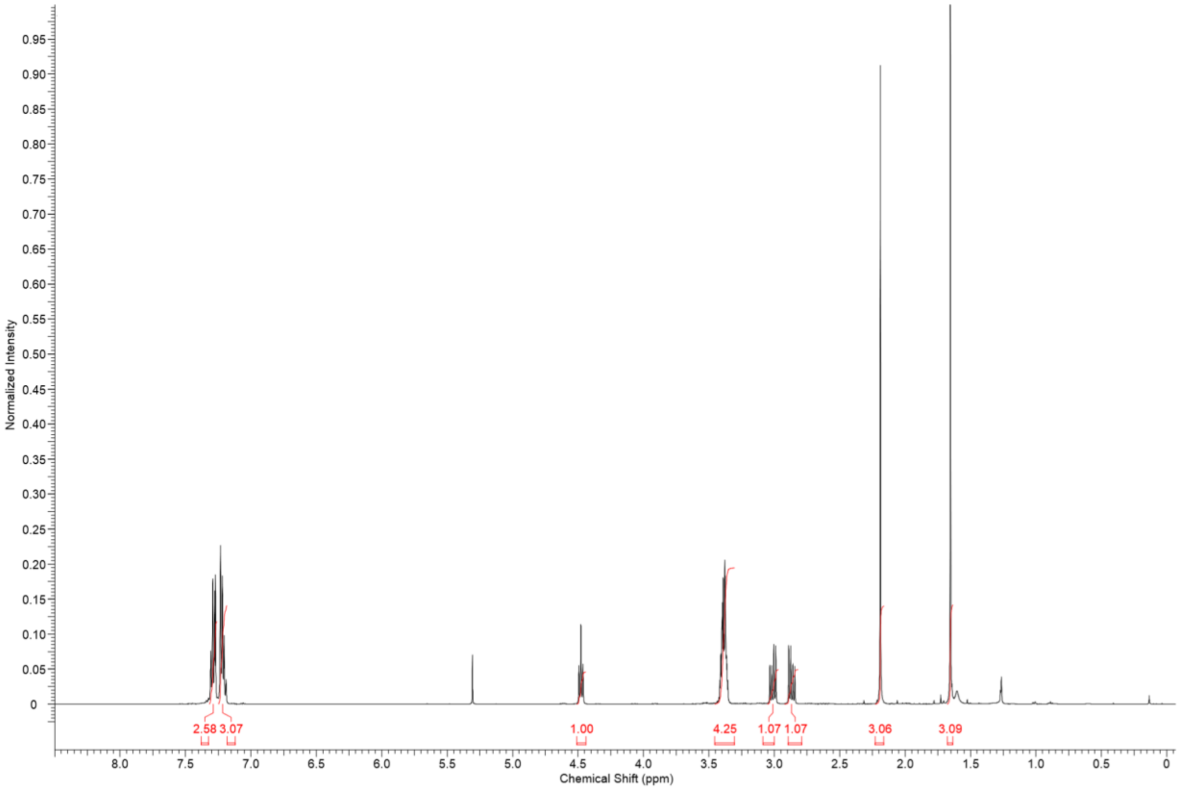

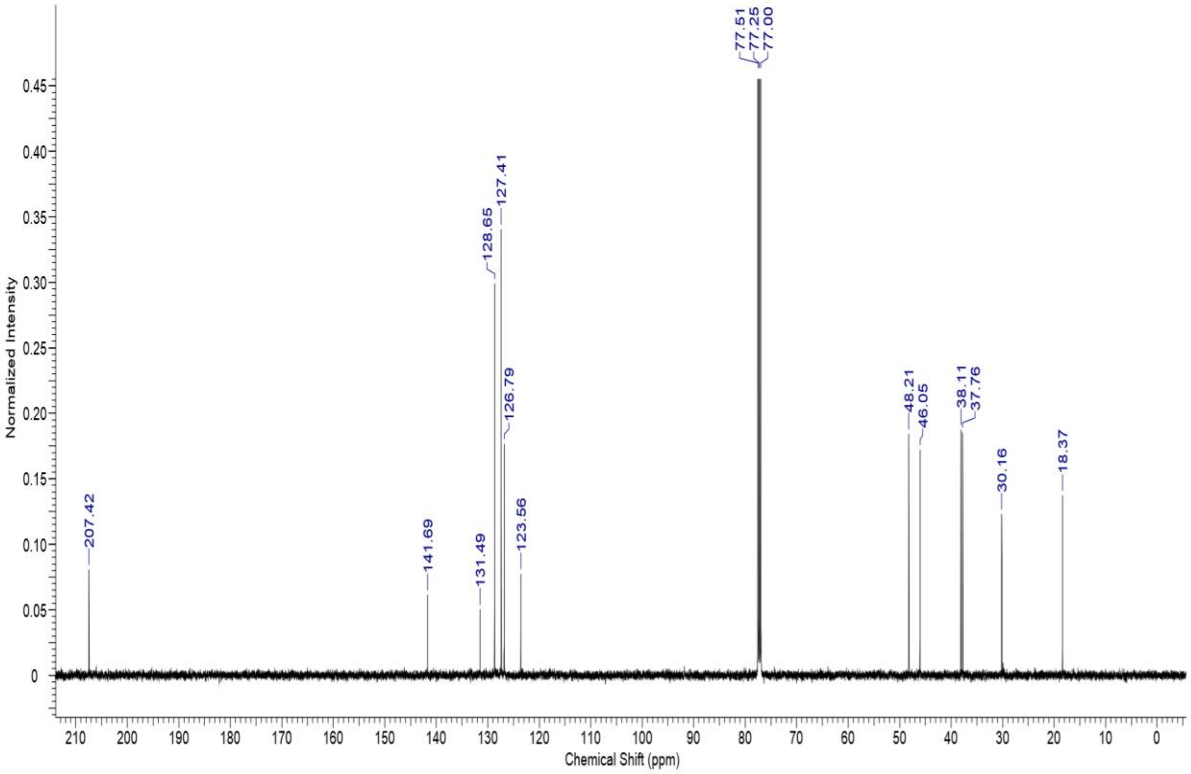

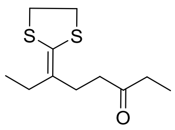

[4m] 6-(1,3-dithiolan-2-ylidene)octan-3-one

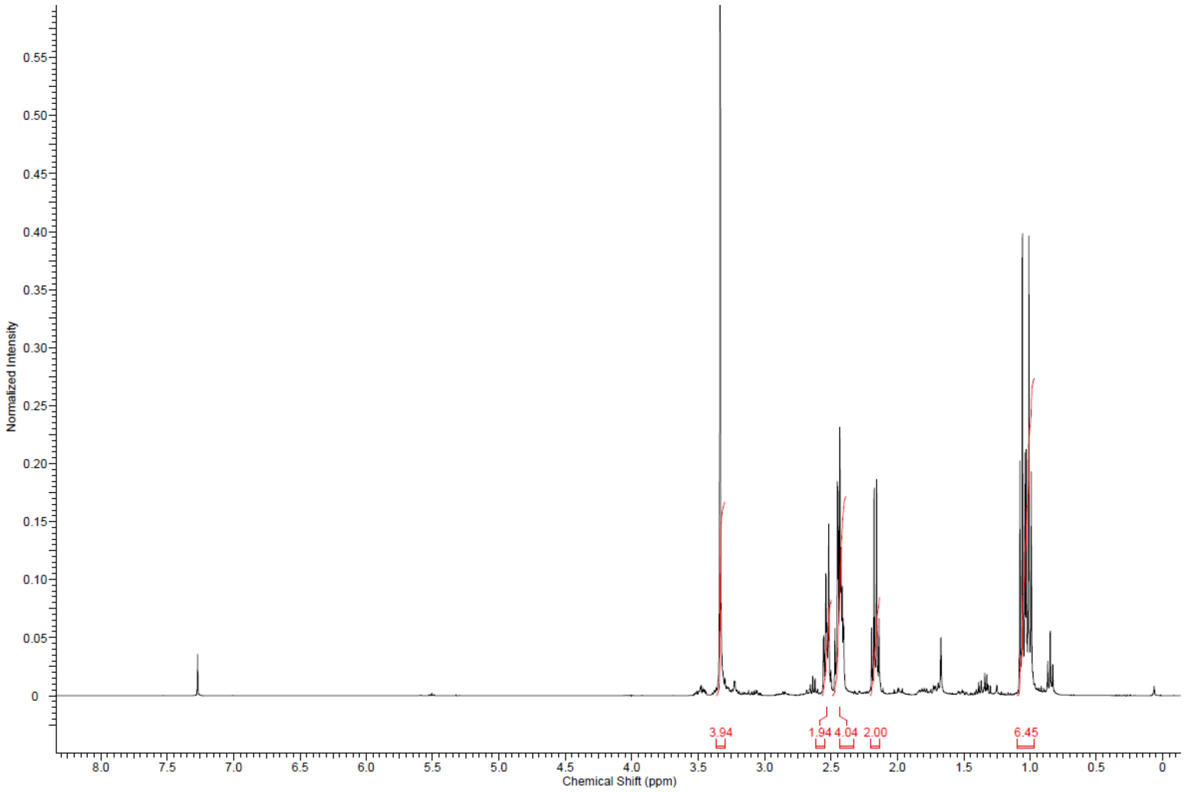

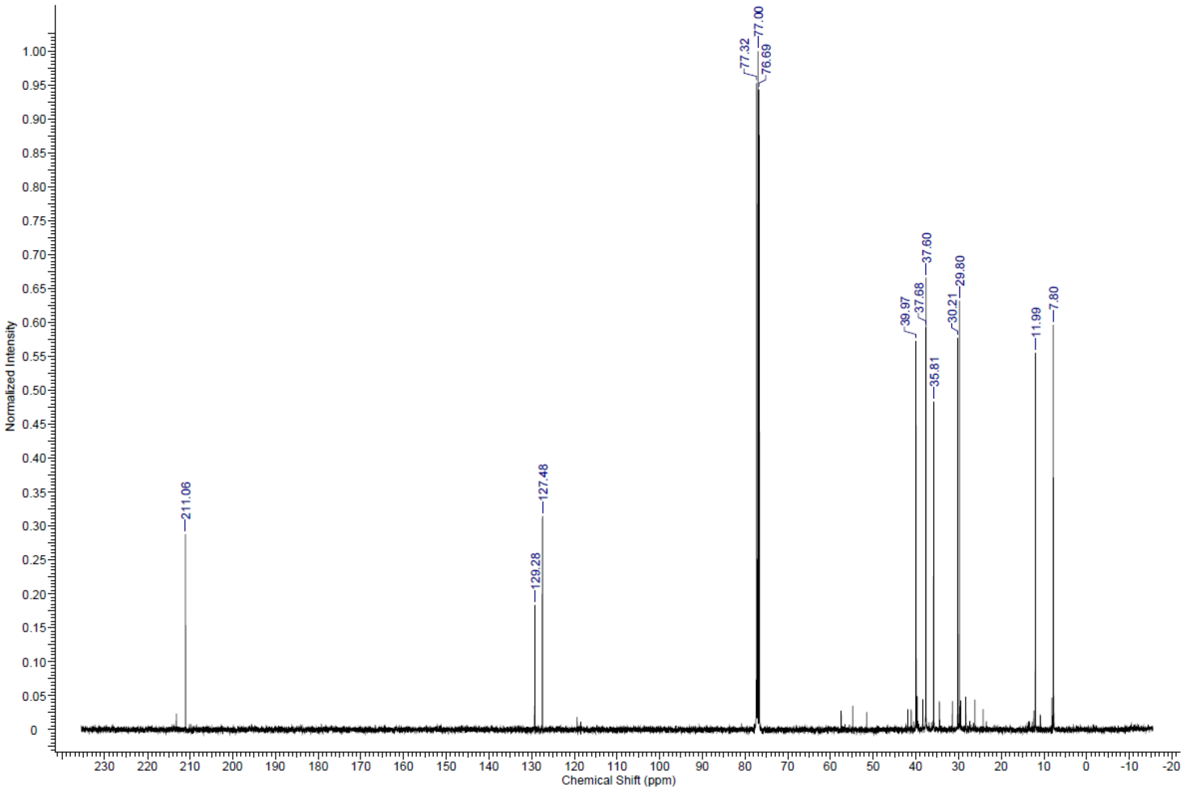

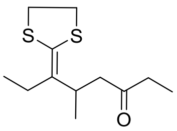

[4n] 6-(1,3-dithiolan-2-ylidene)-5-methyloctan-3-one

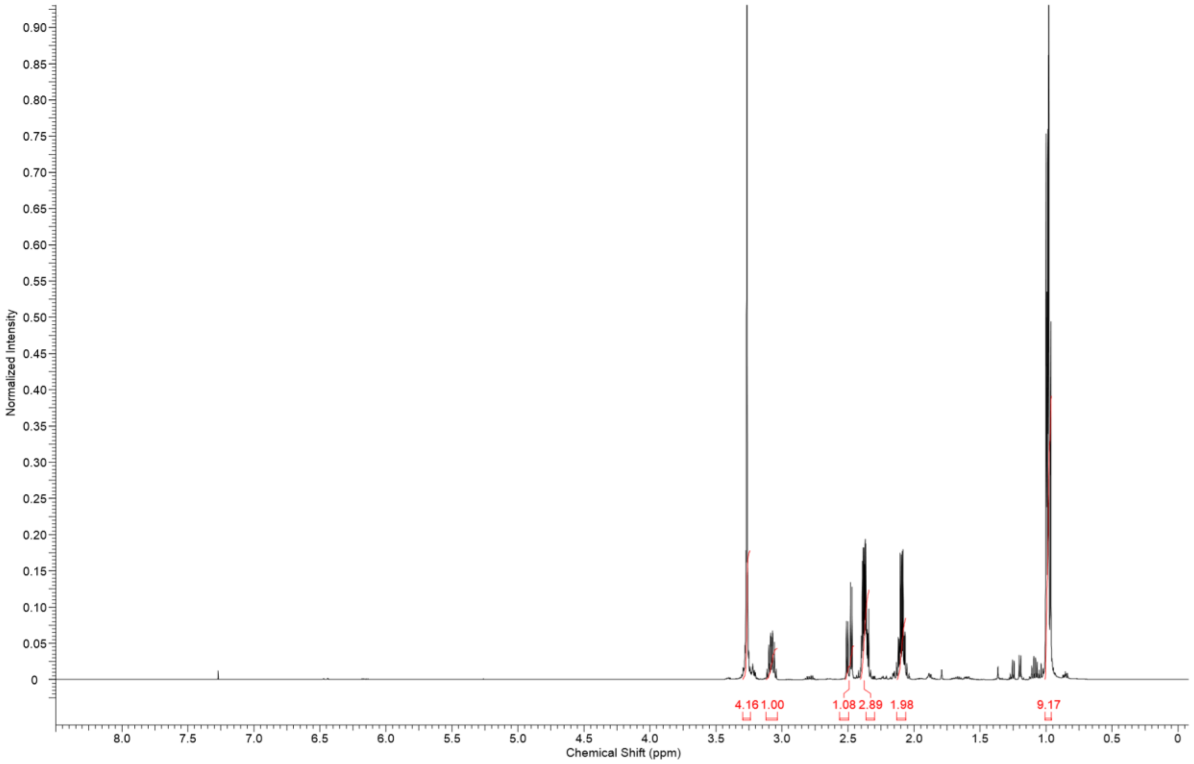

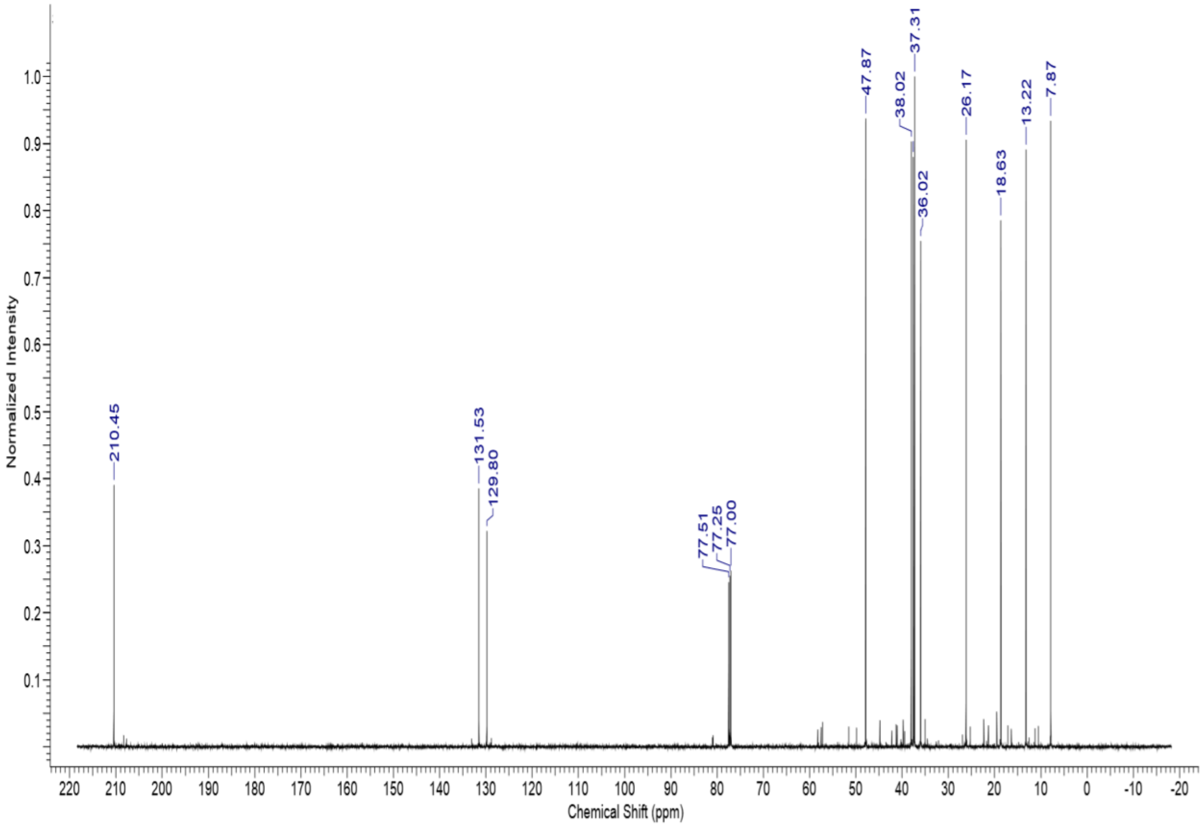

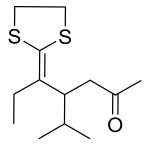

[4o] 5-(1,3-dithiolan-2-ylidene)-4-isopropylheptan-2-one

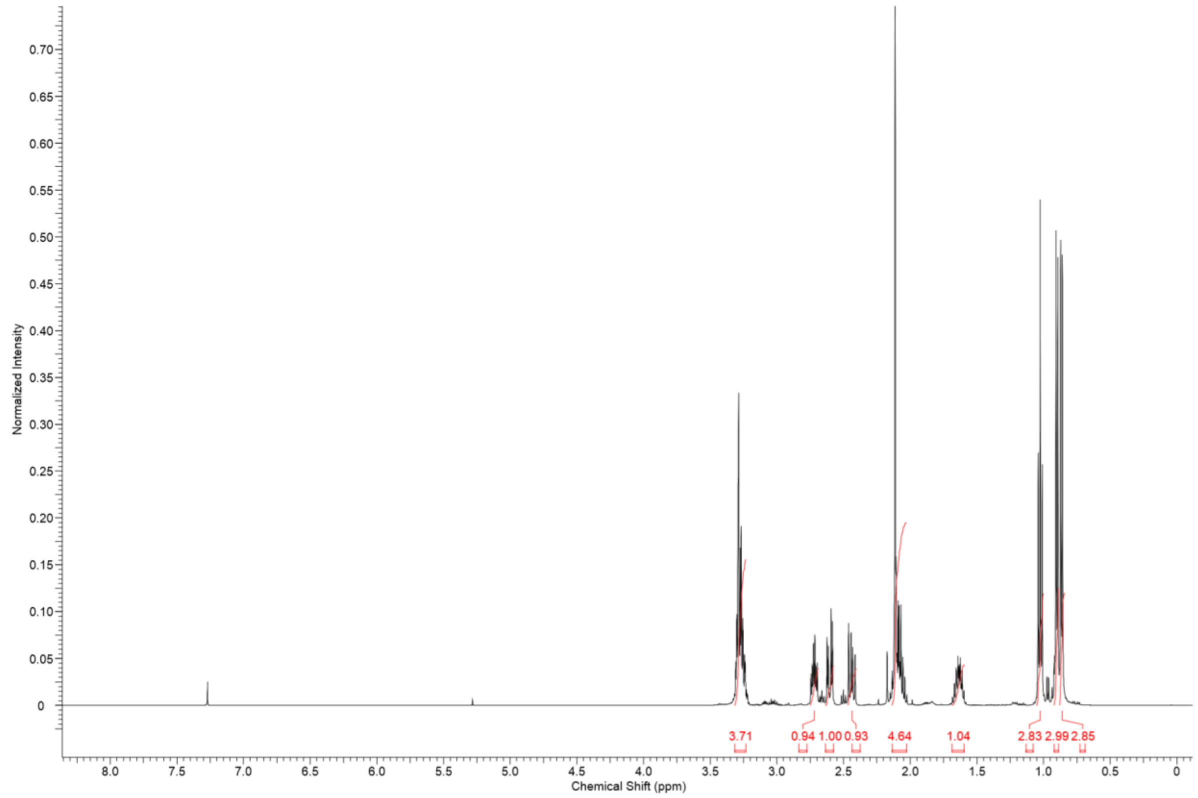

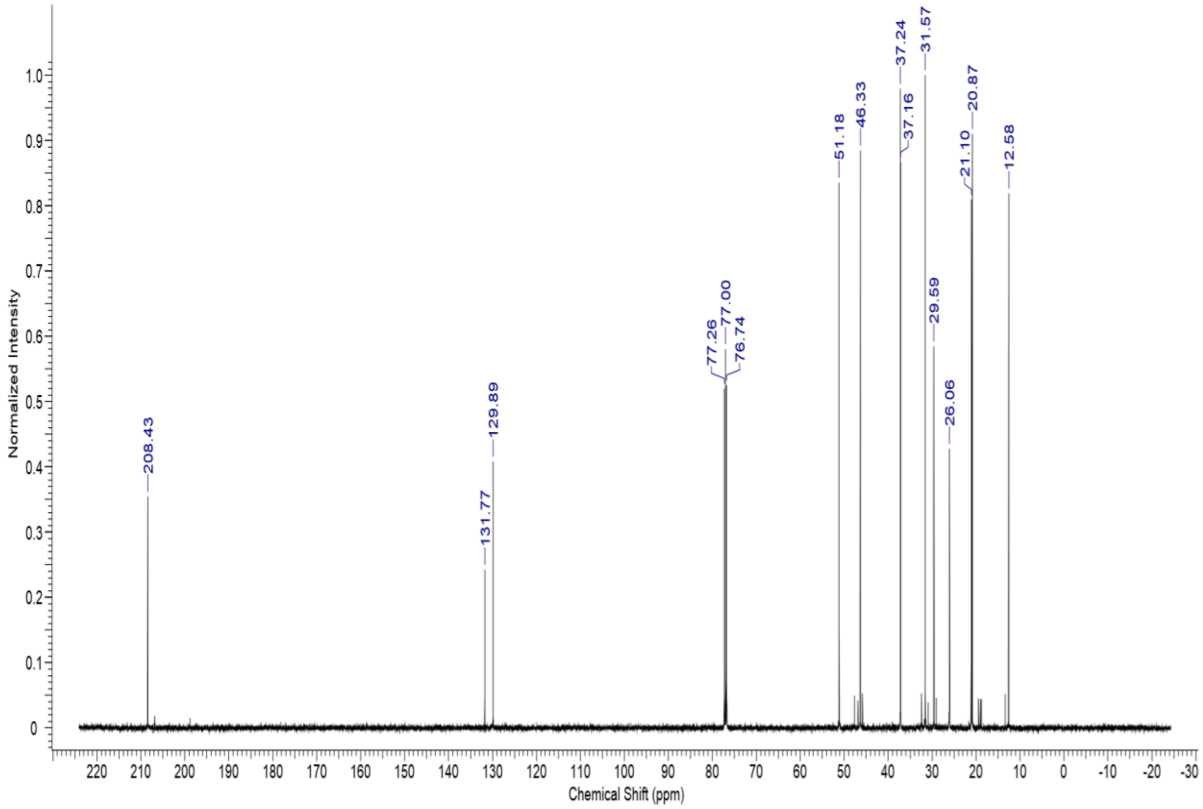

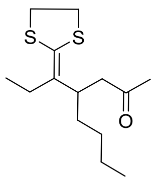

[4p] 4-(1-(1,3-Dithiolan-2-ylidene)propyl)octan-2-one

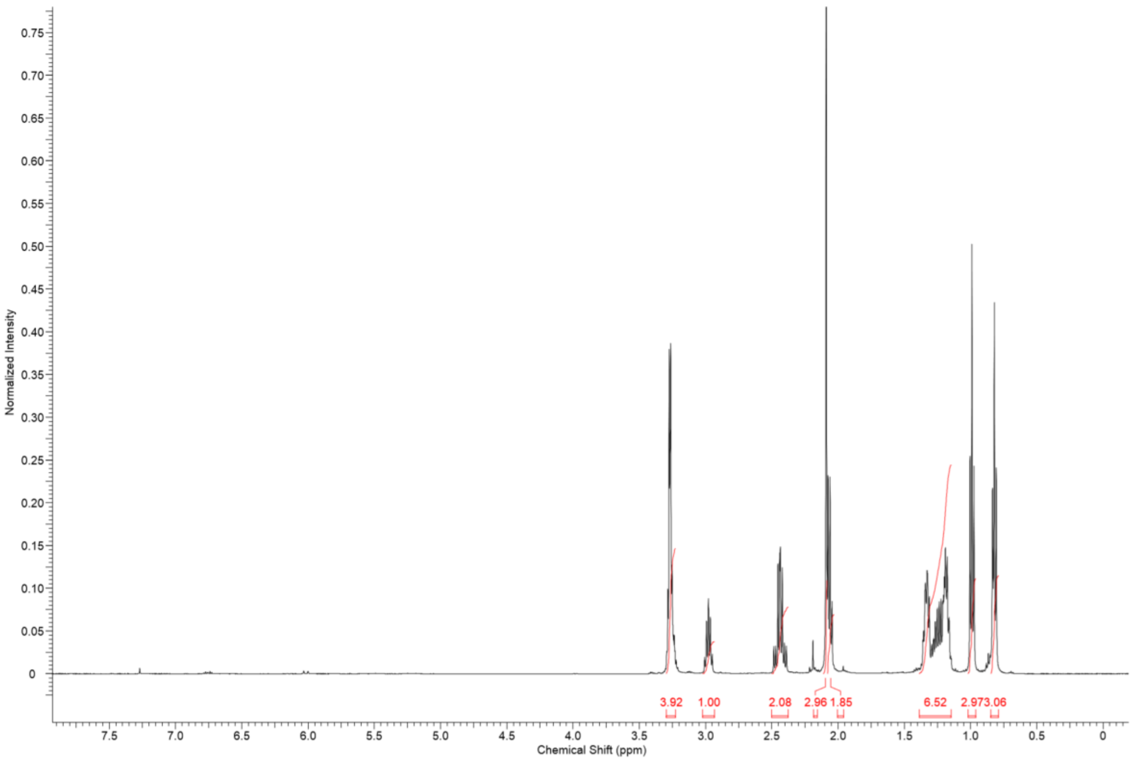

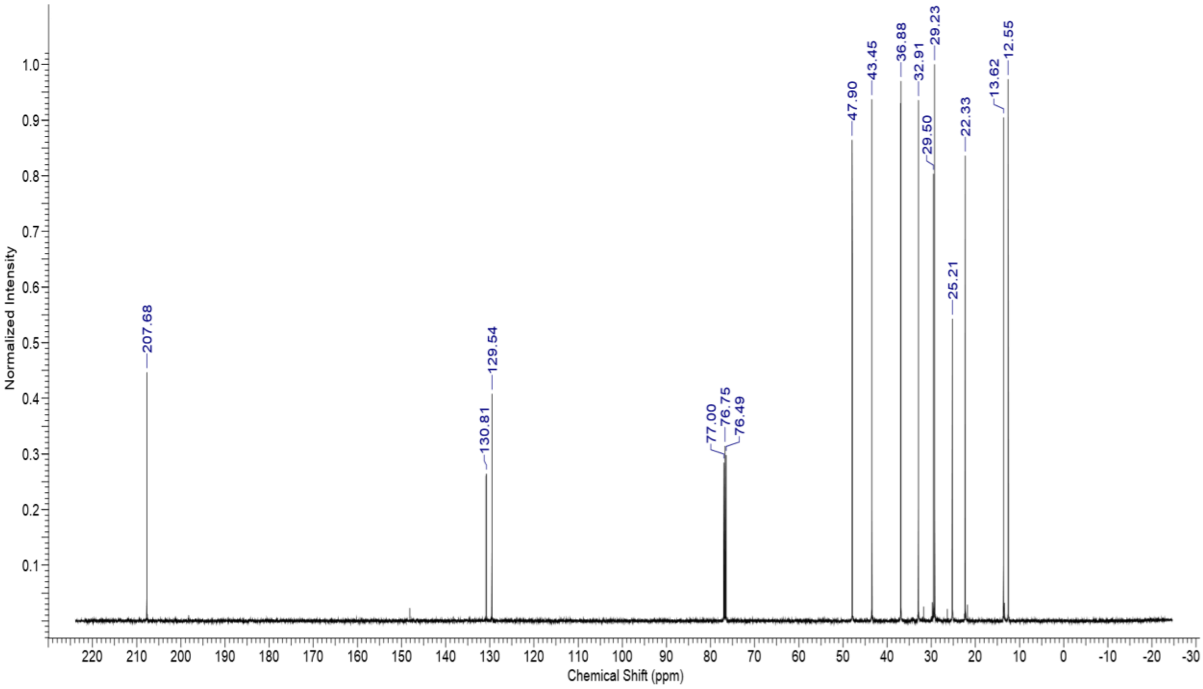

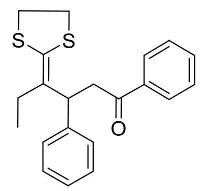

[4q] 4-(1,3-dithiolan-2-ylidene)-1,3-diphenylhexan-1-one

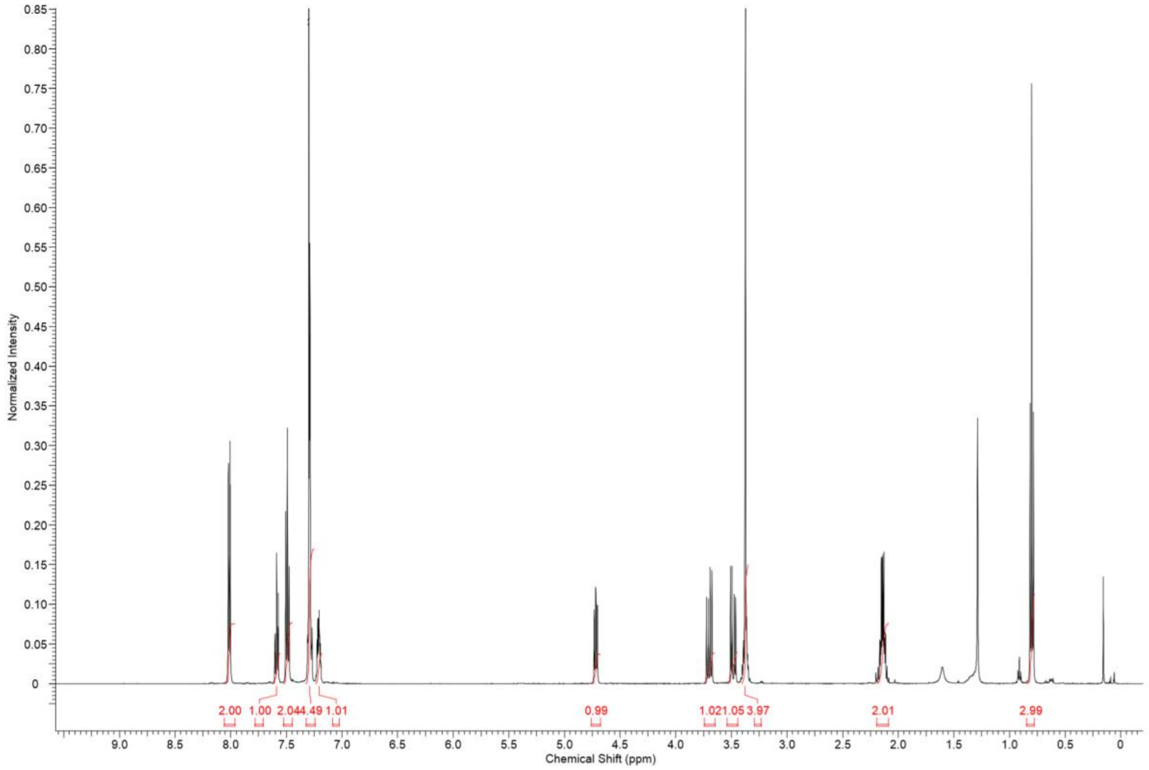

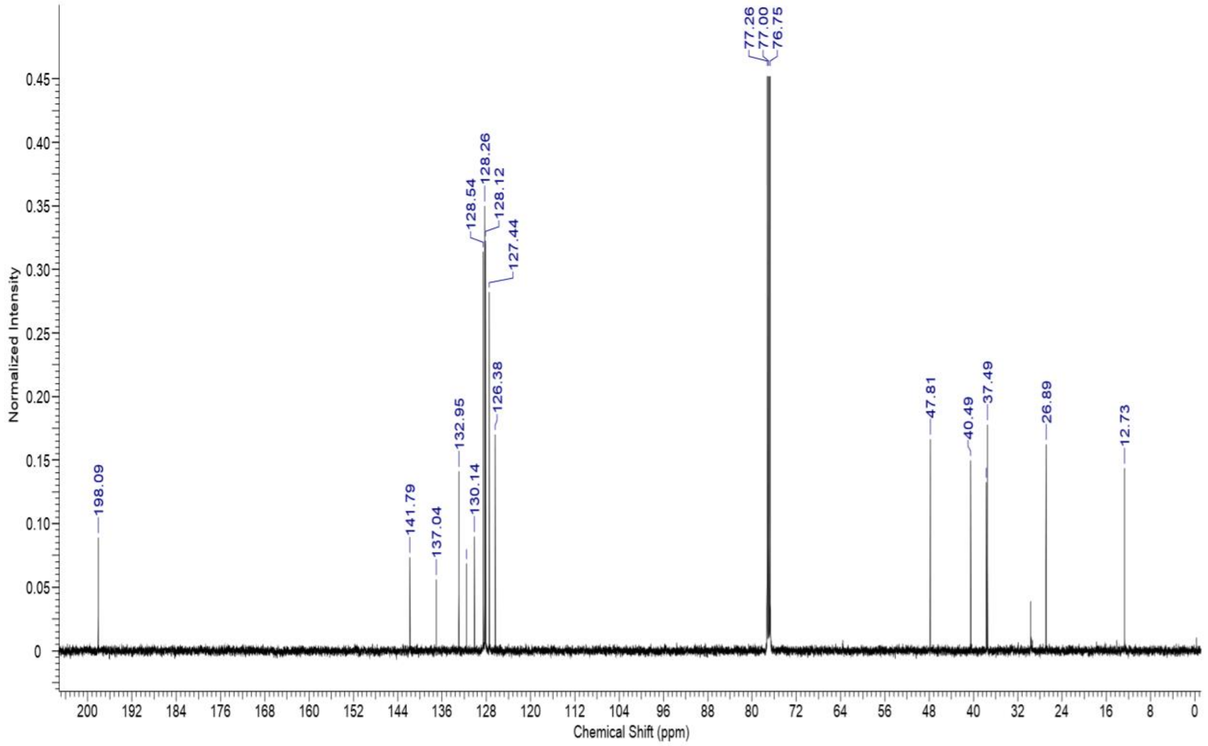

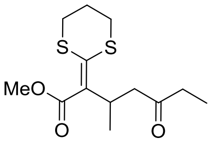

[5r] Methyl 2-(1,3-dithian-2-ylidene)-3-methyl-5-oxoheptanoate

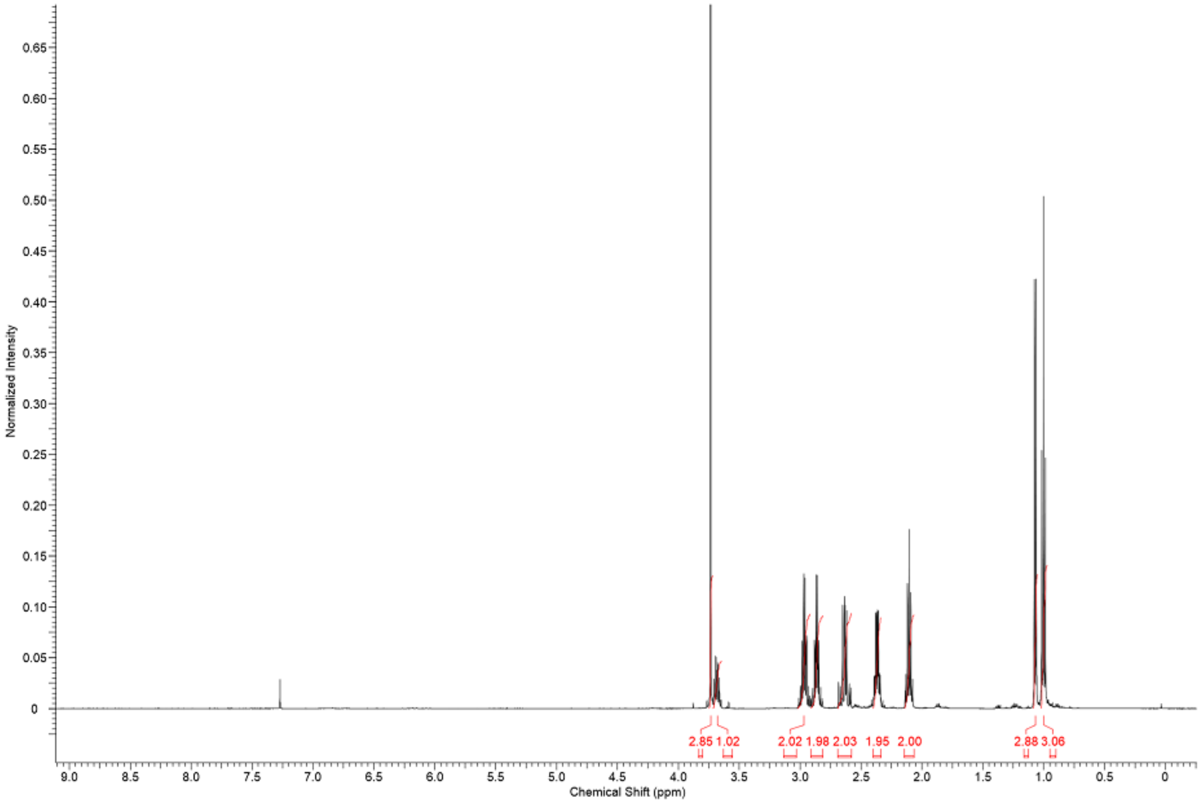

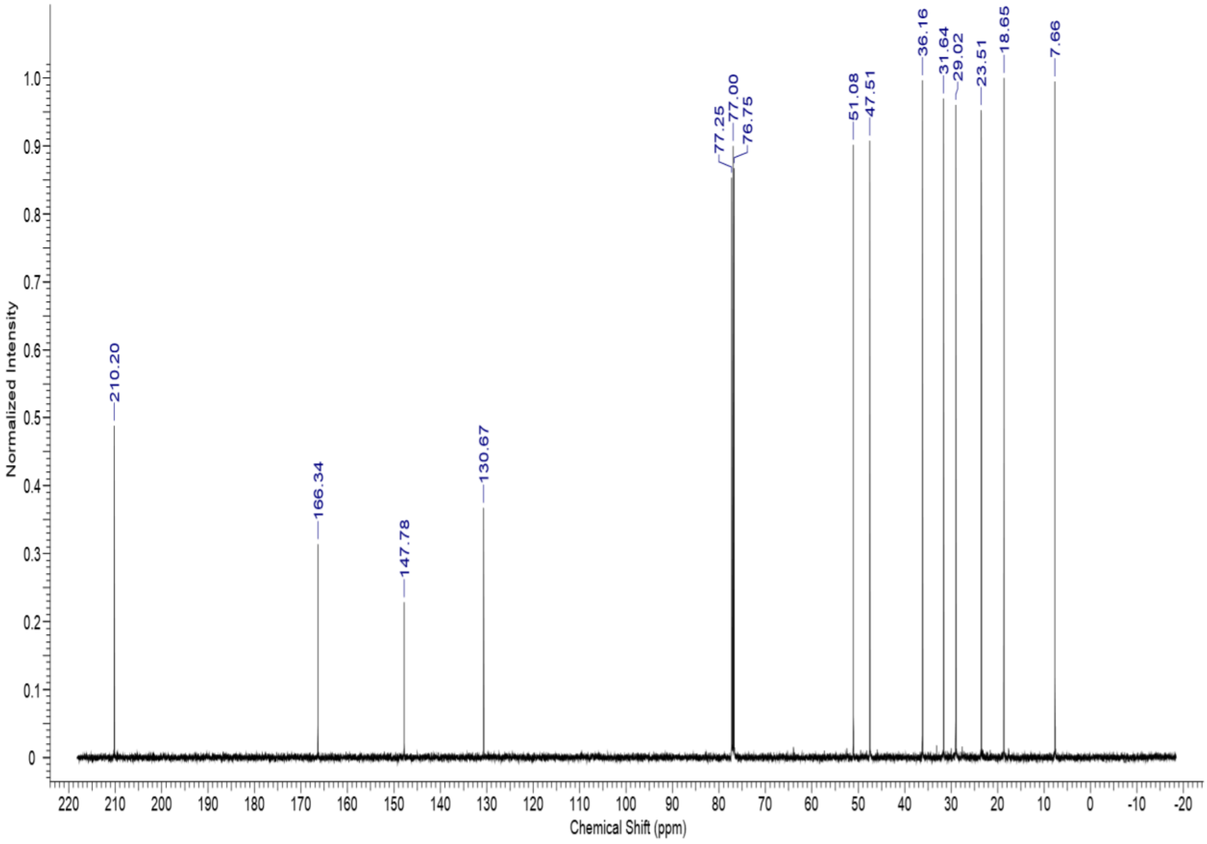

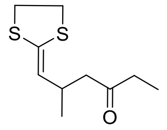

[4s] 6-(1,3-dithiolan-2-ylidene)-5-methylhexan-3-one

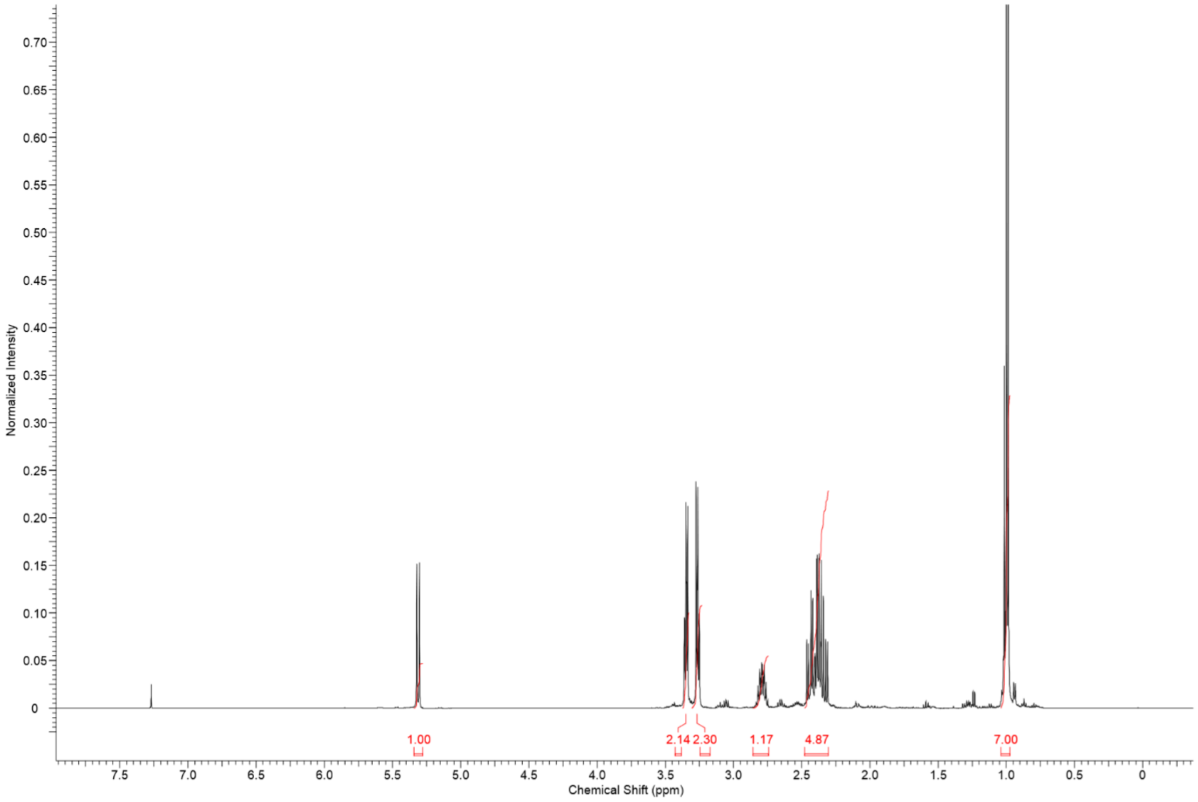

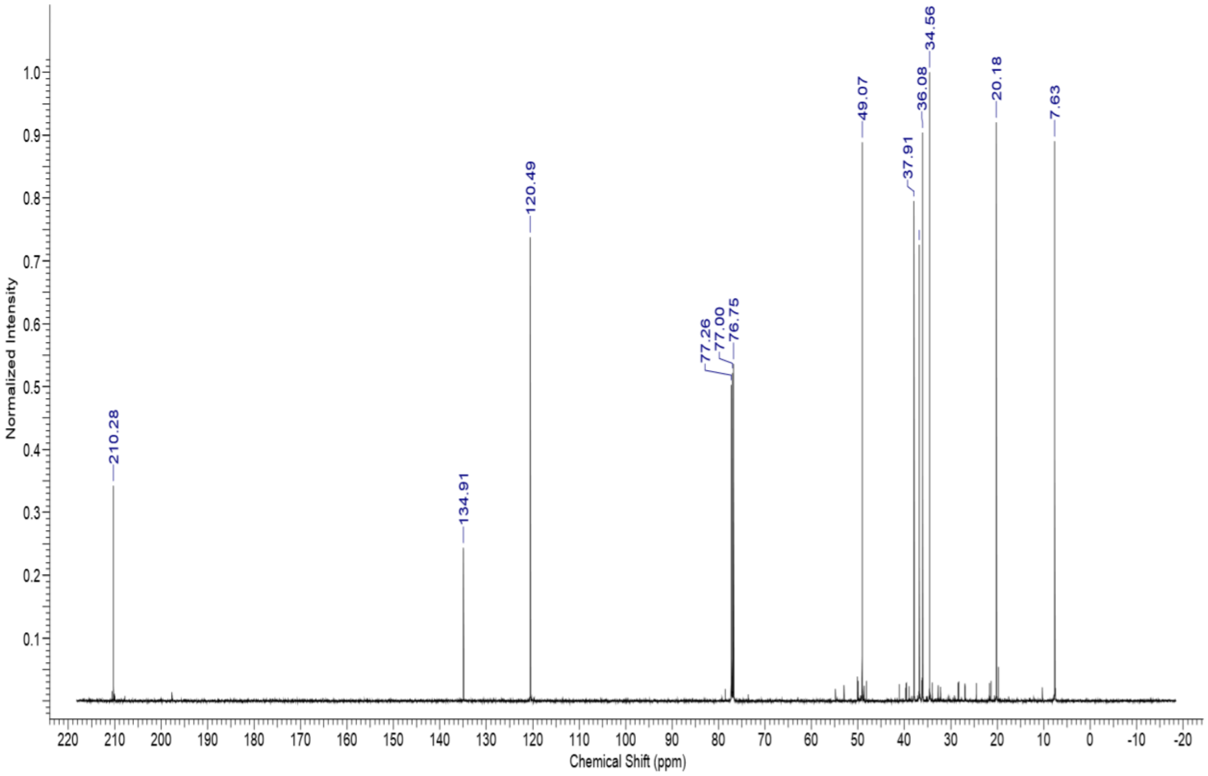

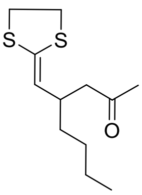

[4t] 4-((1,3-dithiolan-2-ylidene)methyl)octan-2-one

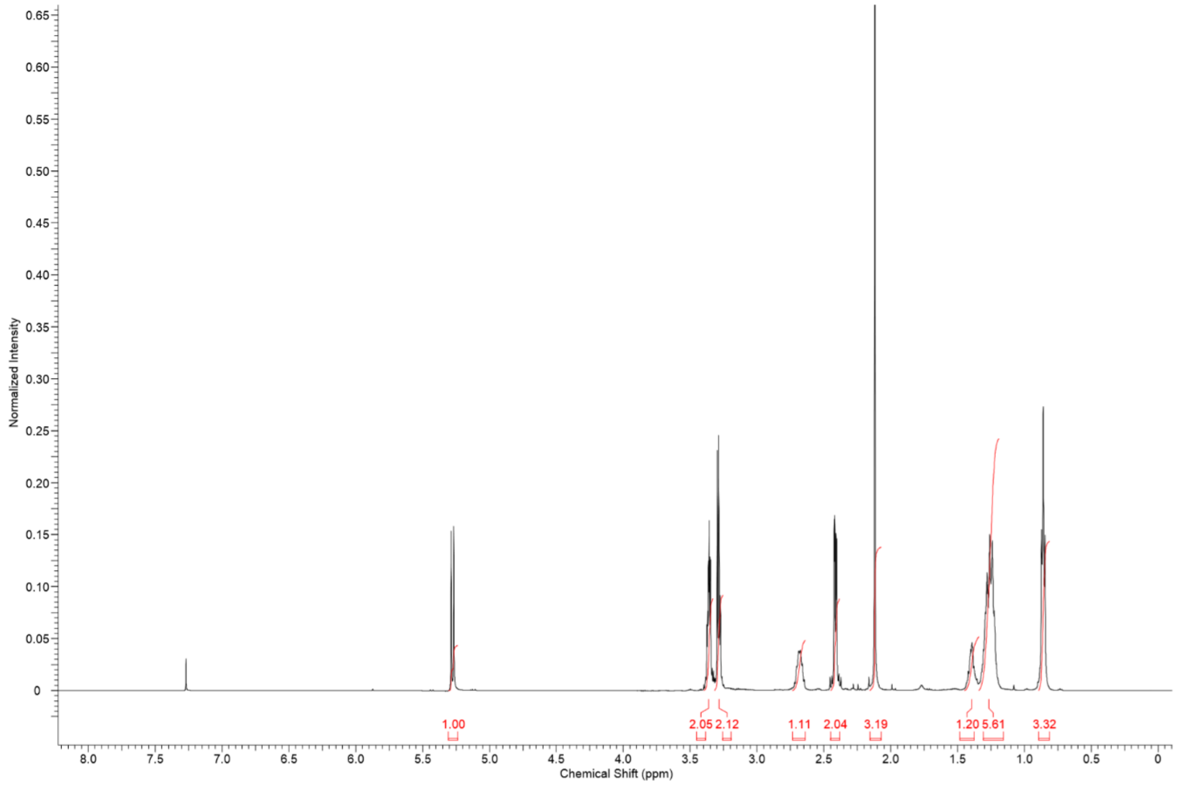

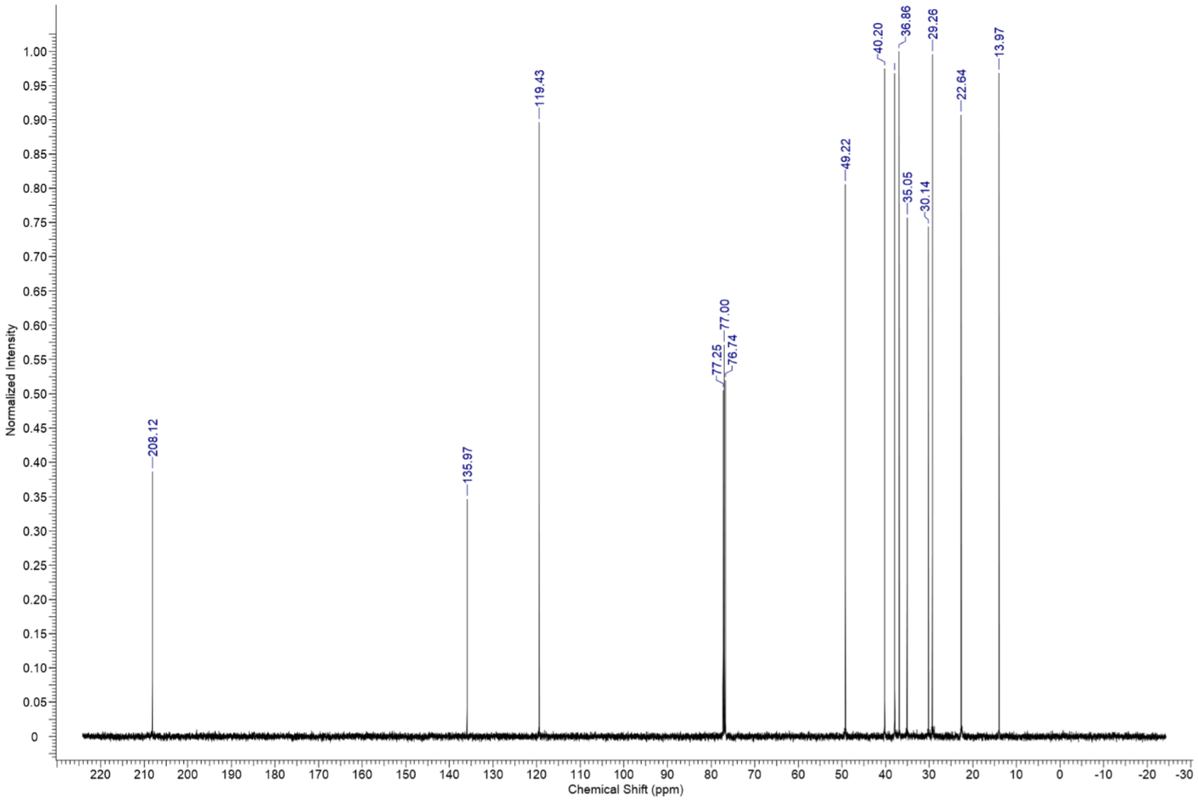

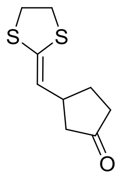

[4u] 3-((1,3-Dithiolan-2-ylidene)methyl)cyclopentan-1-one

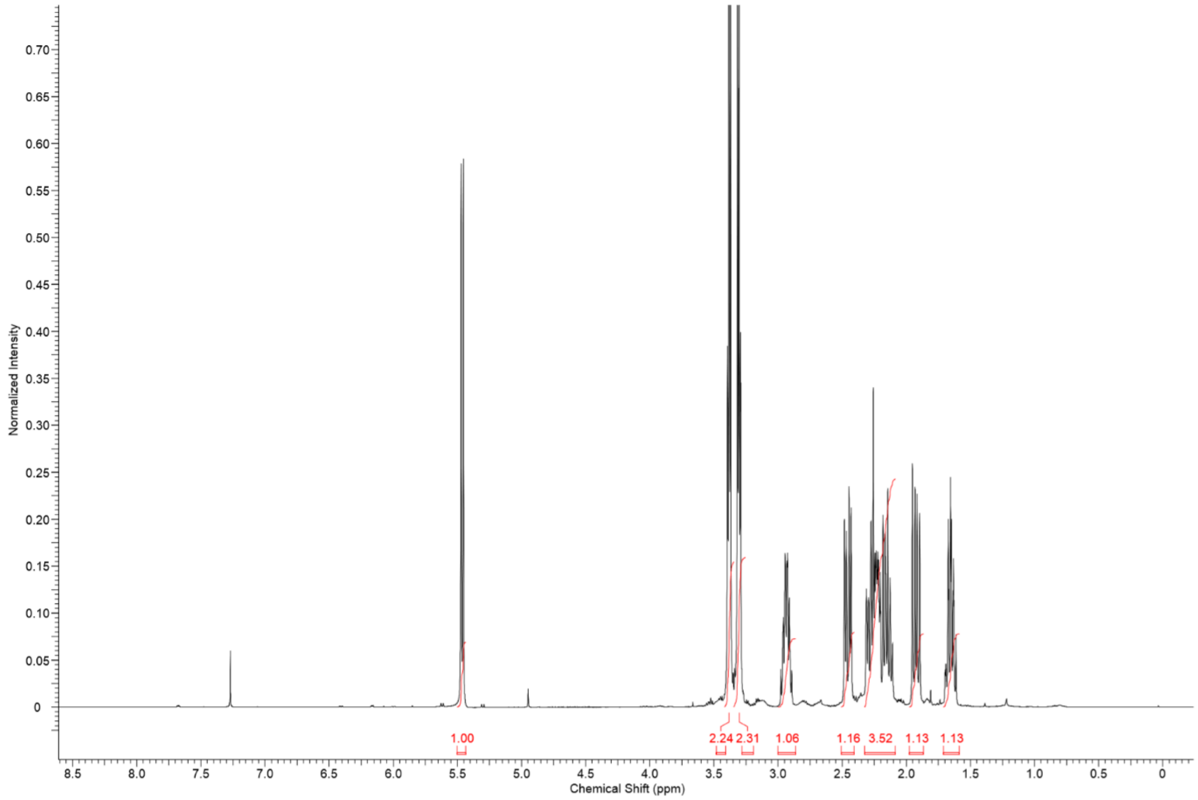

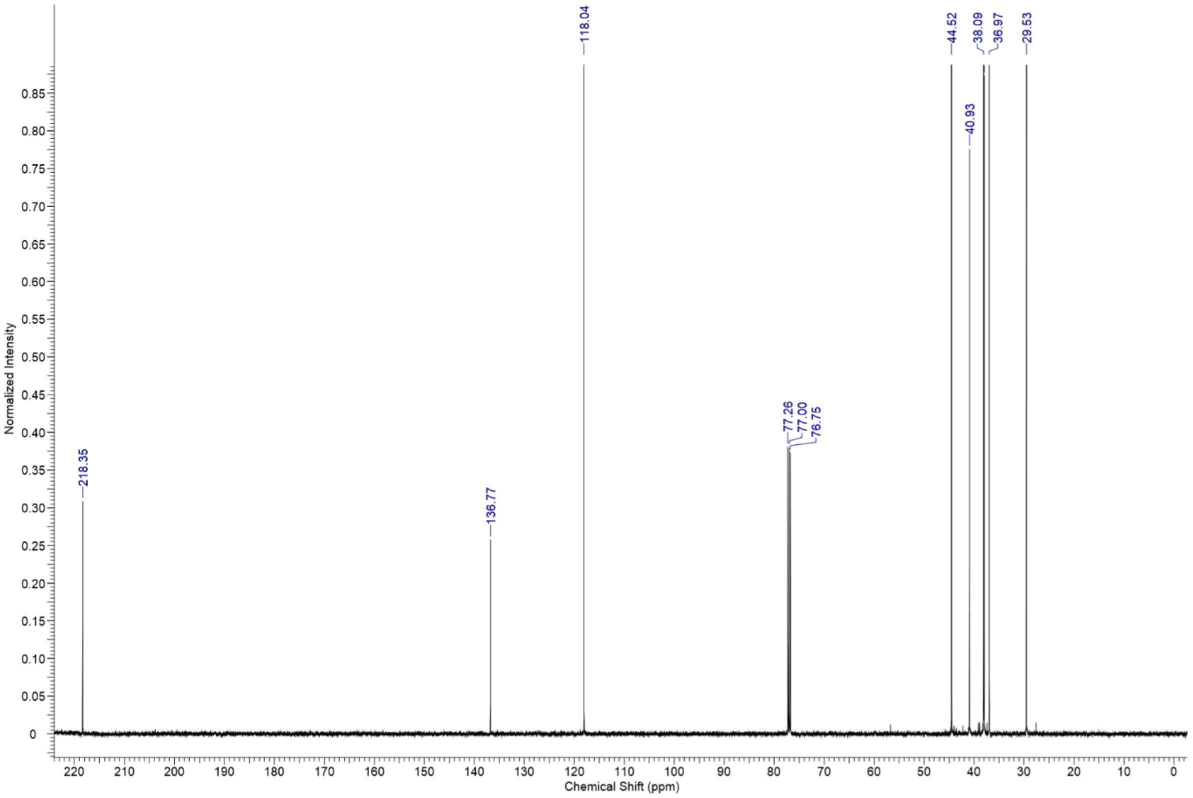

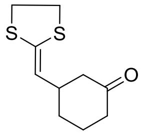

[4v] 3-((1,3-dithiolan-2-ylidene)methyl)cyclohexan-1-one

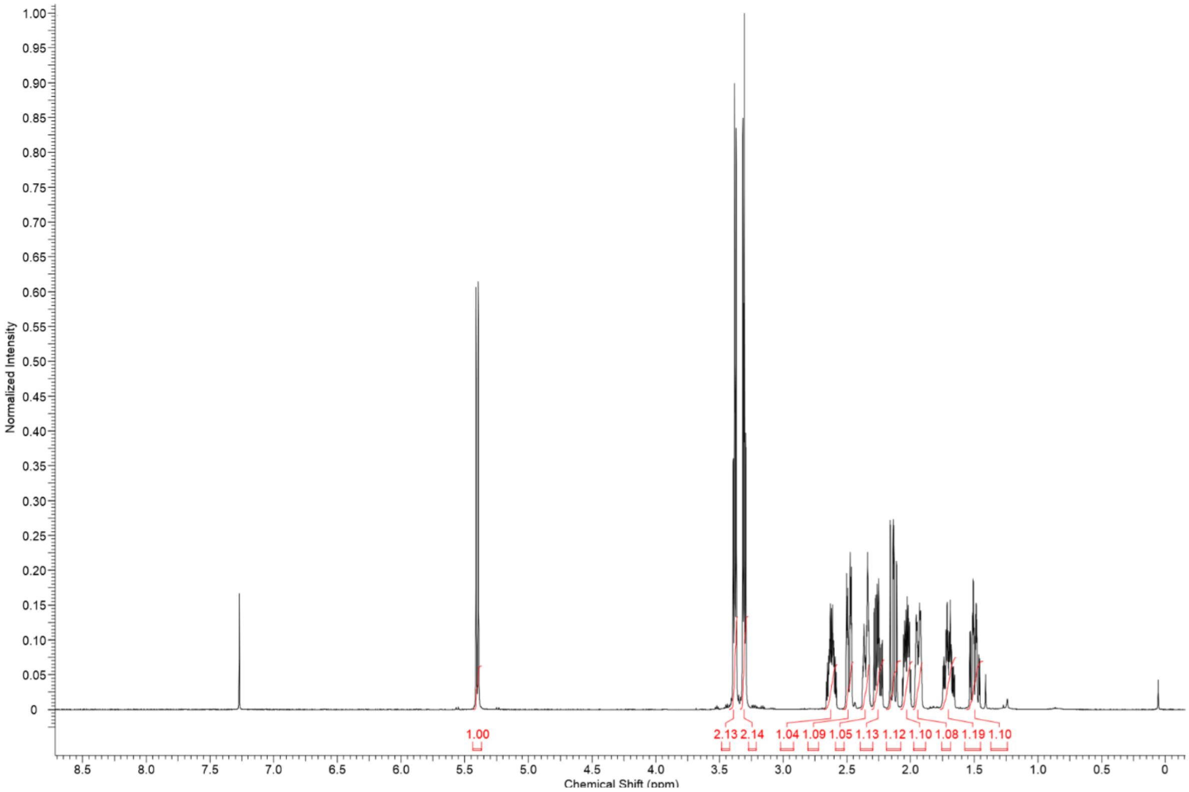

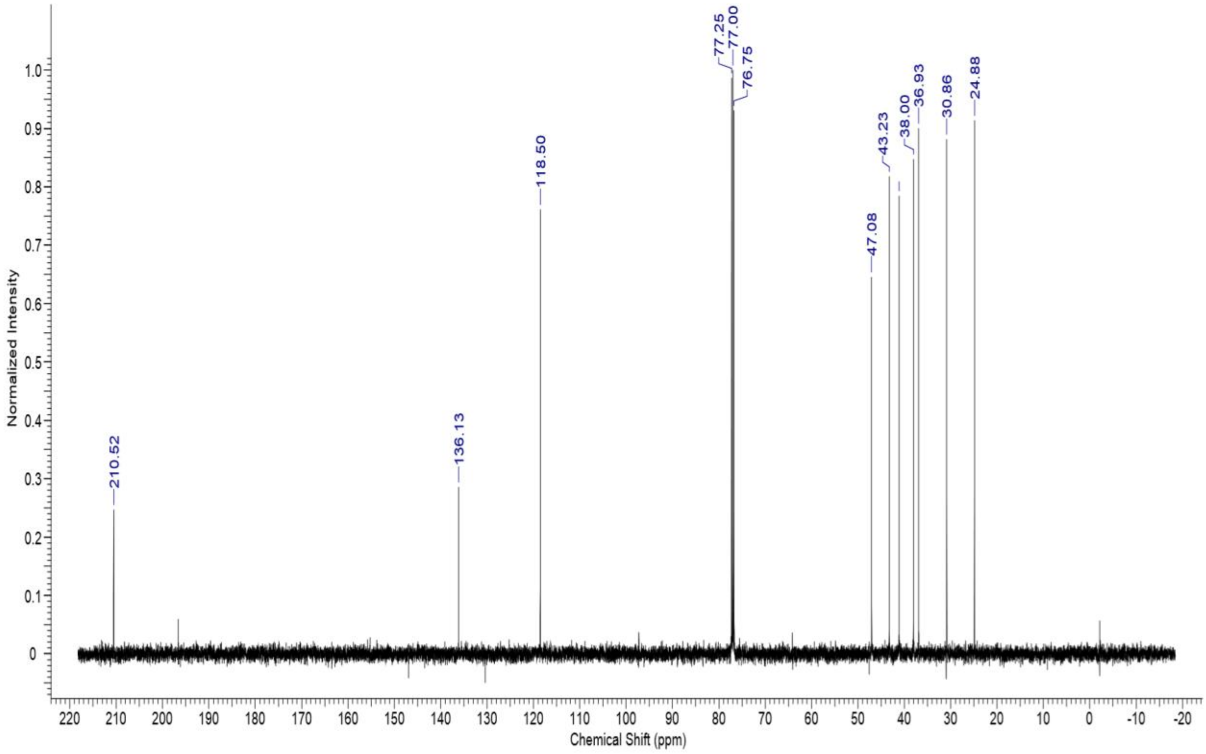

Spectra of compounds type **9** and **10**

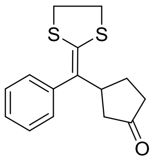

[9a] 3-((1,3-dithiolan-2-ylidene)(phenyl)methyl)cyclopentan-1-one

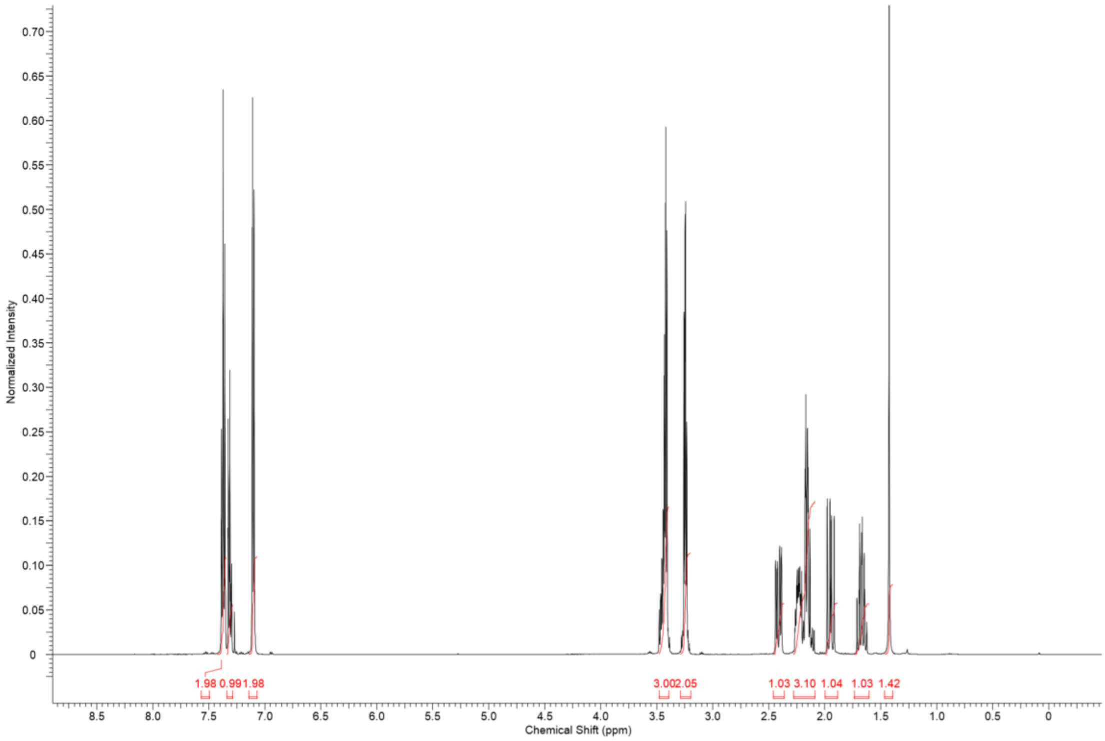

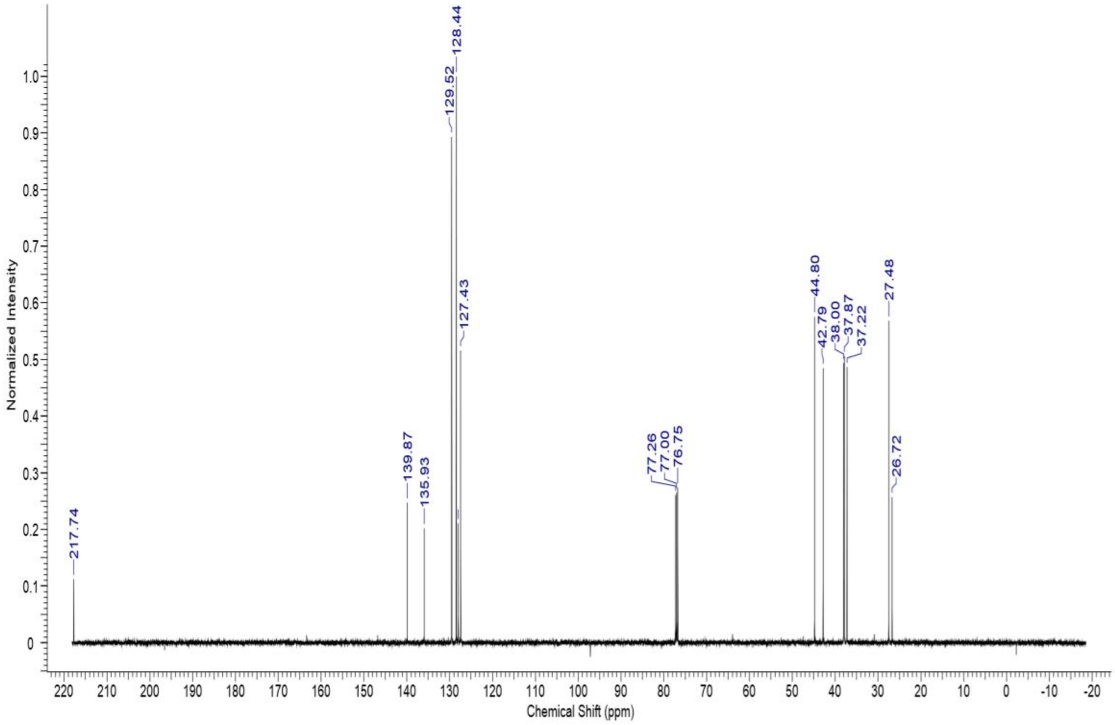

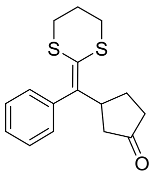

[10a] 3-((1,3-dithian-2-ylidene)(phenyl)methyl)cyclopentan-1-one

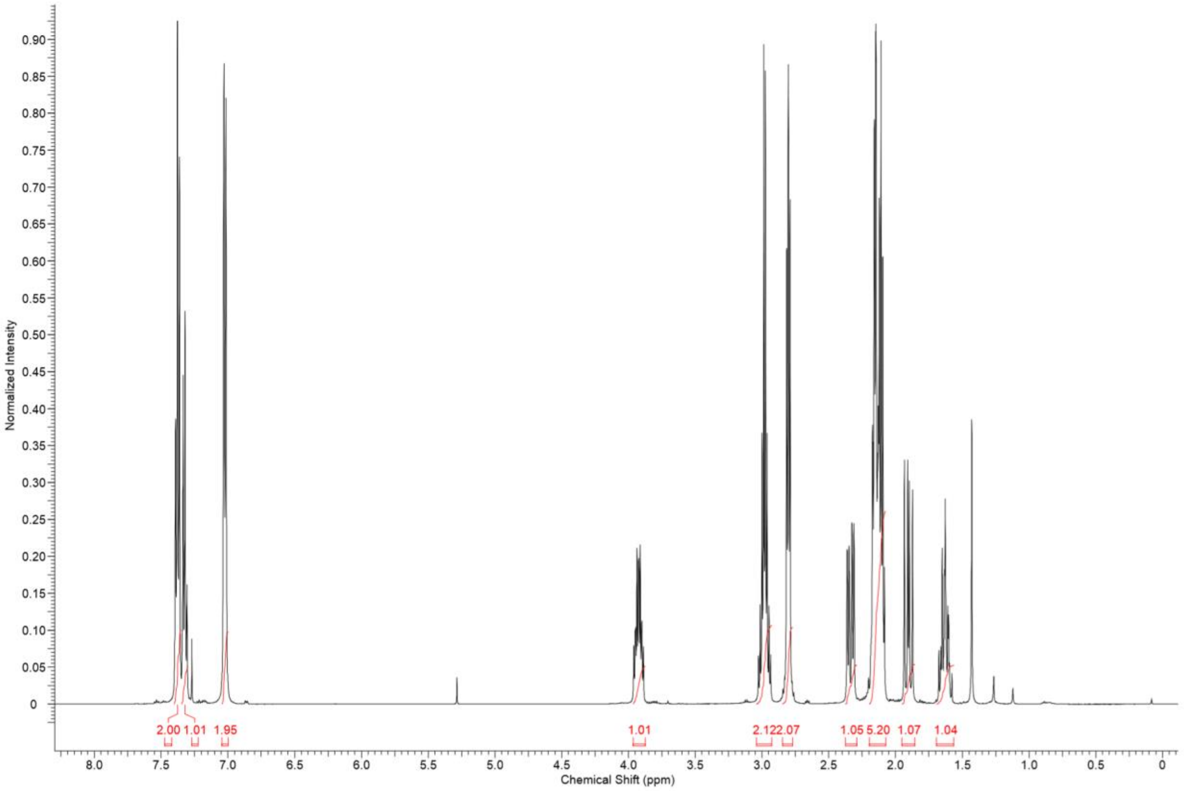

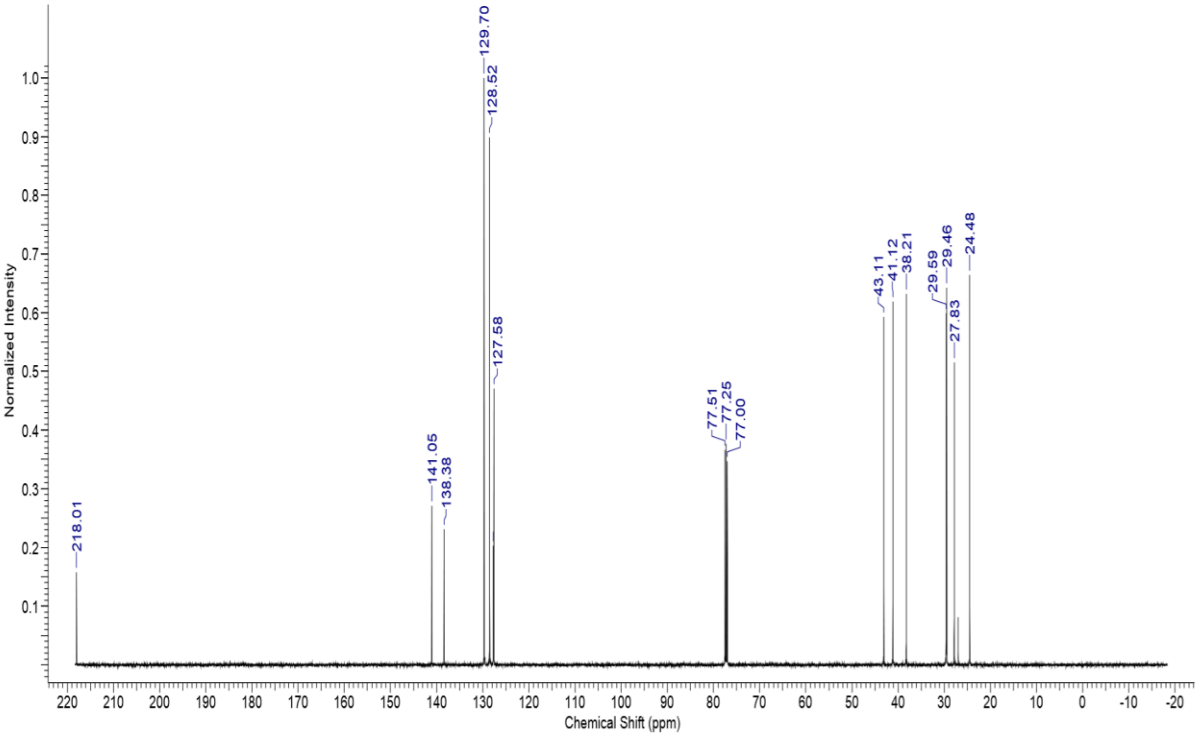

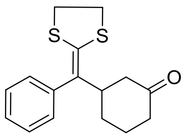

[9b] 3-((1,3-dithiolan-2-ylidene)(phenyl)methyl)cyclohexan-1-one

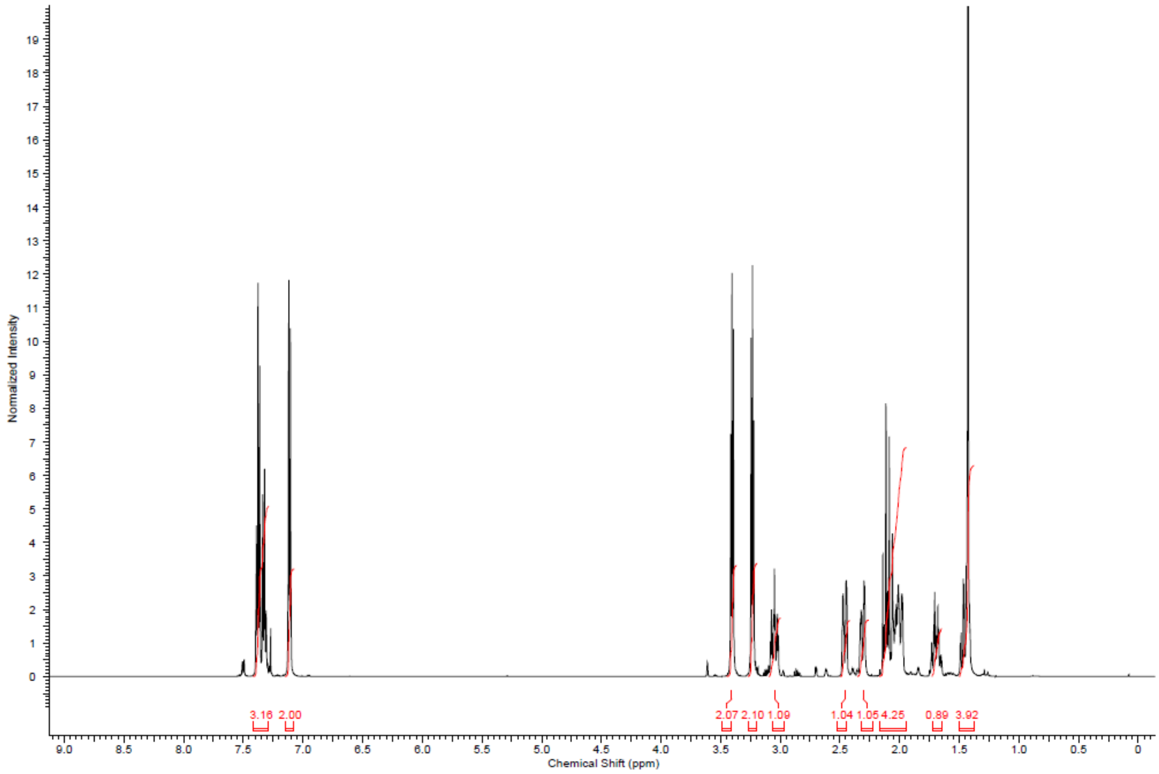

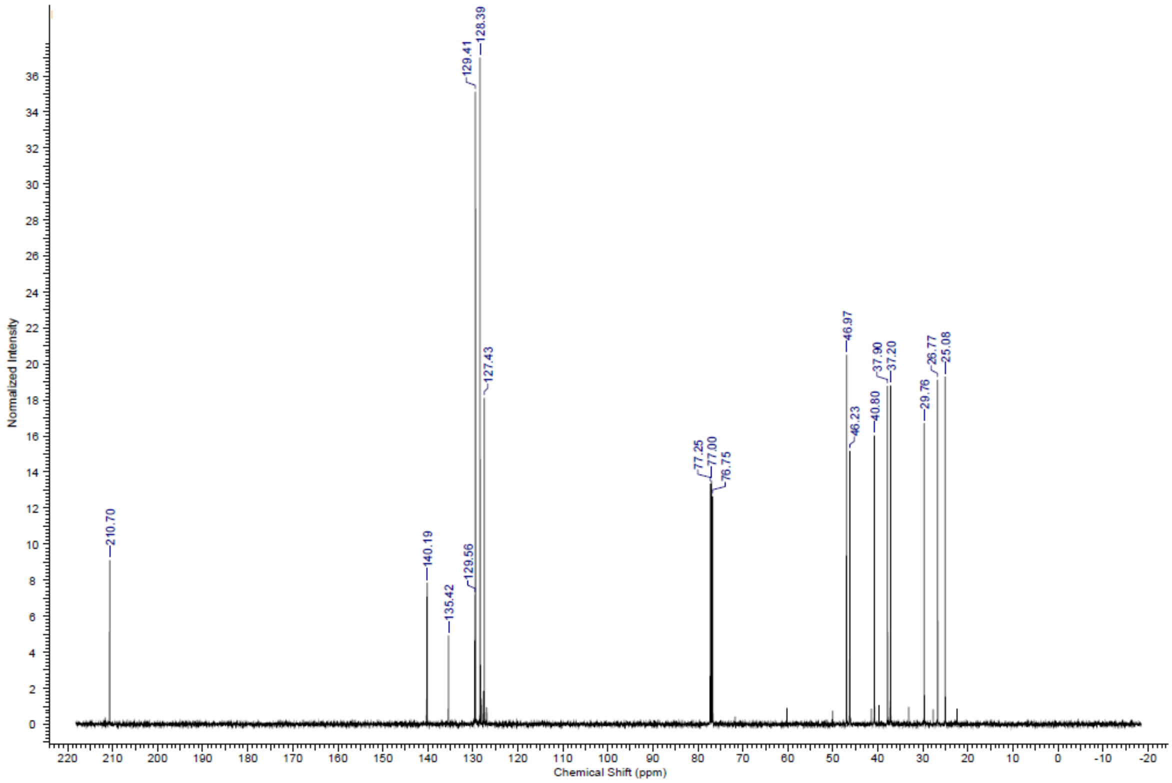

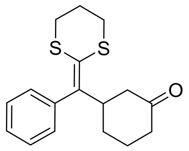

[10b] 3-((1,3-dithian-2-ylidene)(phenyl)methyl)cyclohexan-1-one

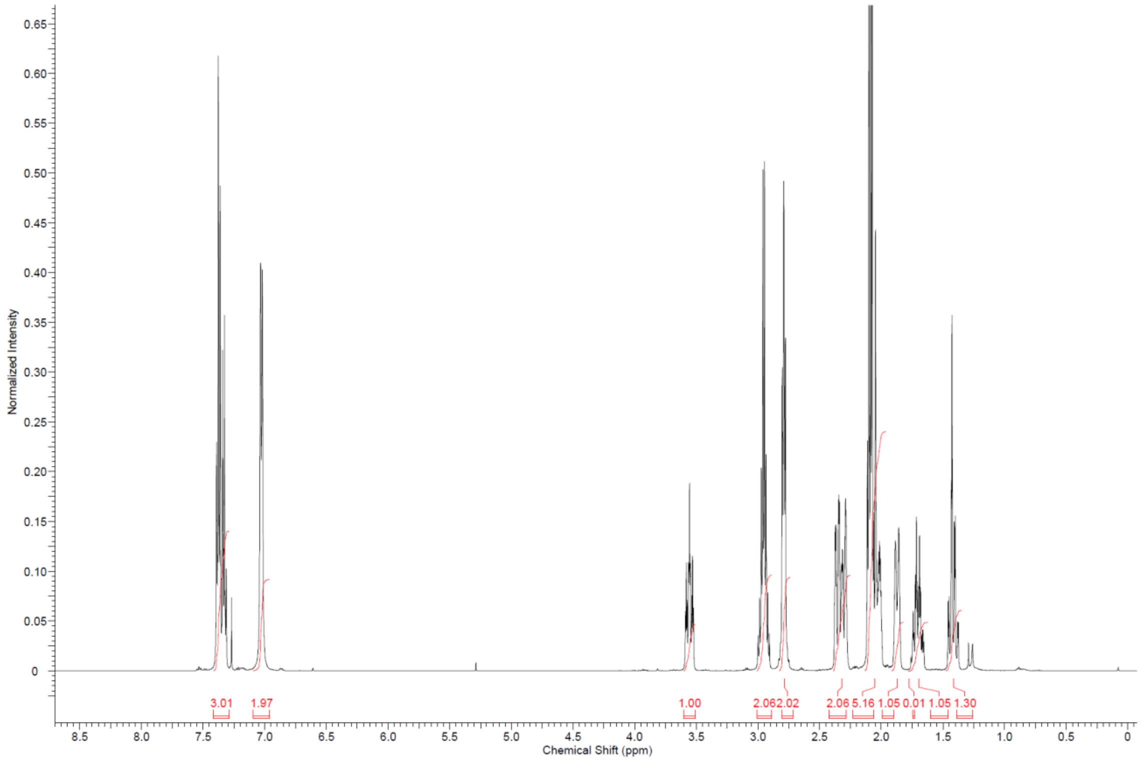

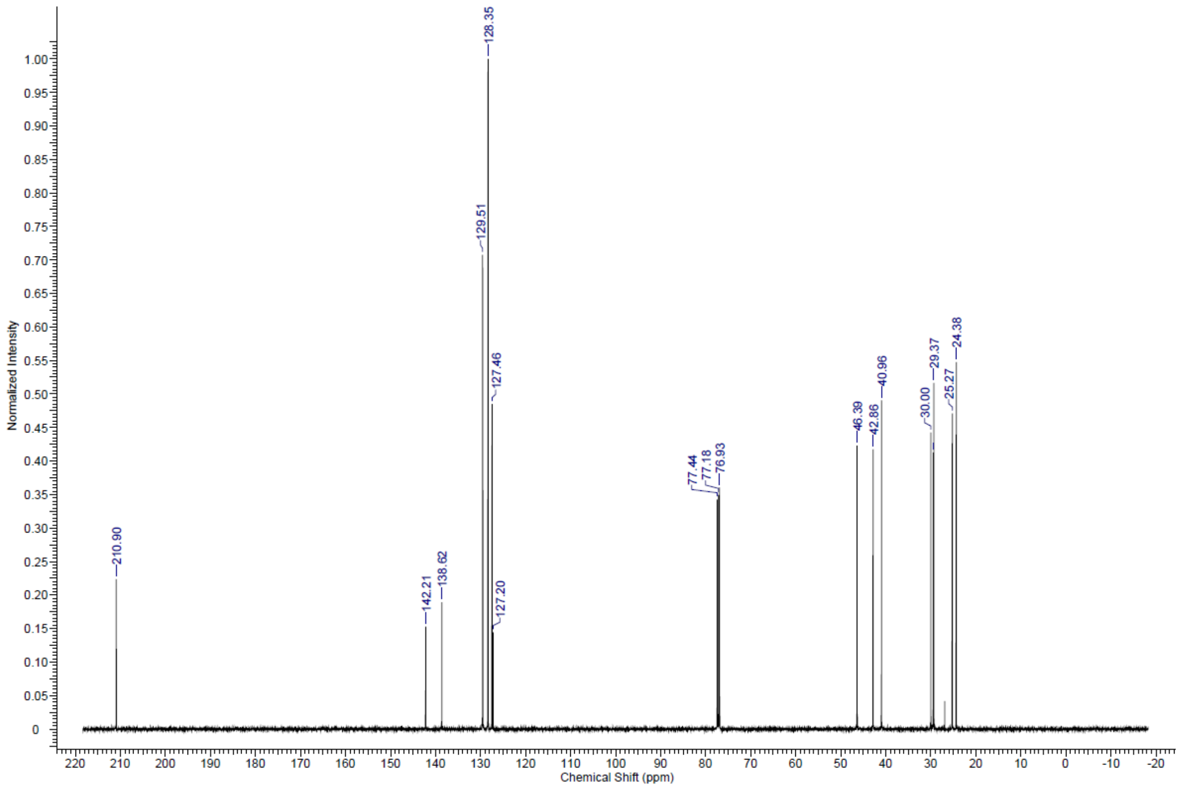

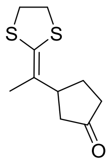

[9c] 3-(1-(1,3-dithiolan-2-ylidene)ethyl)cyclopentan-1-one

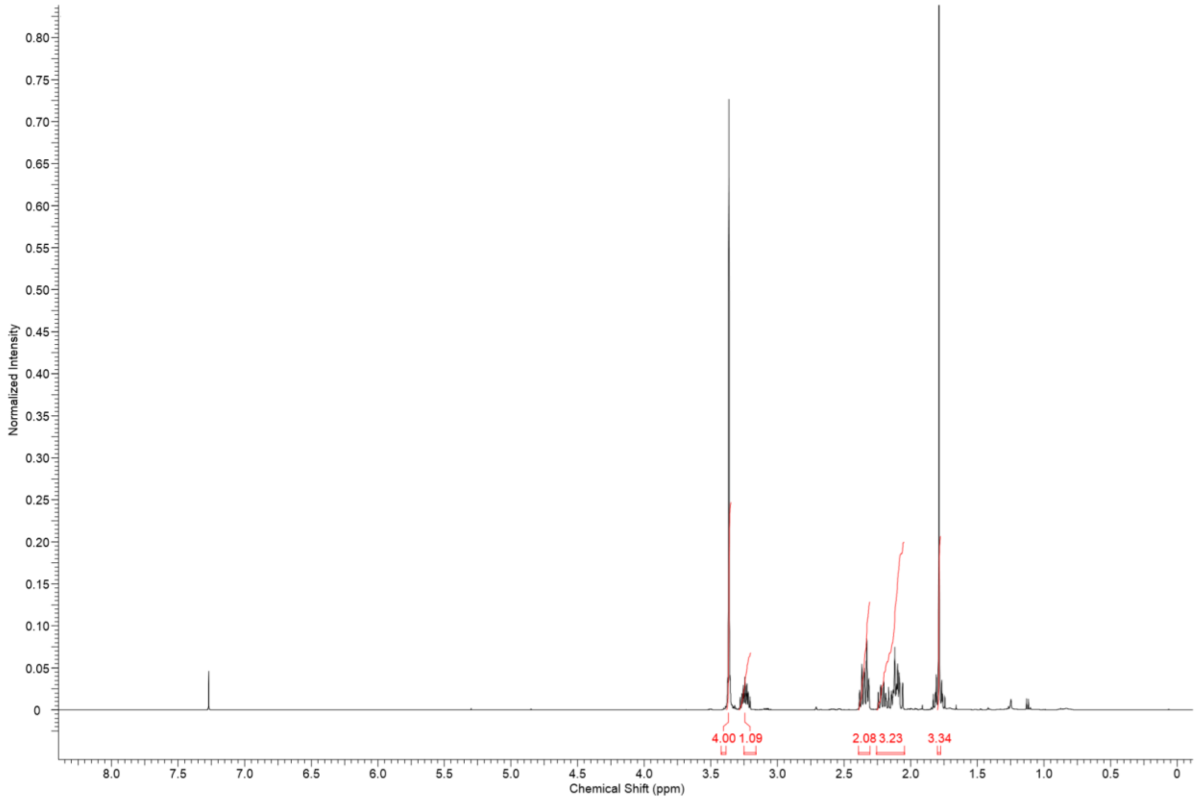

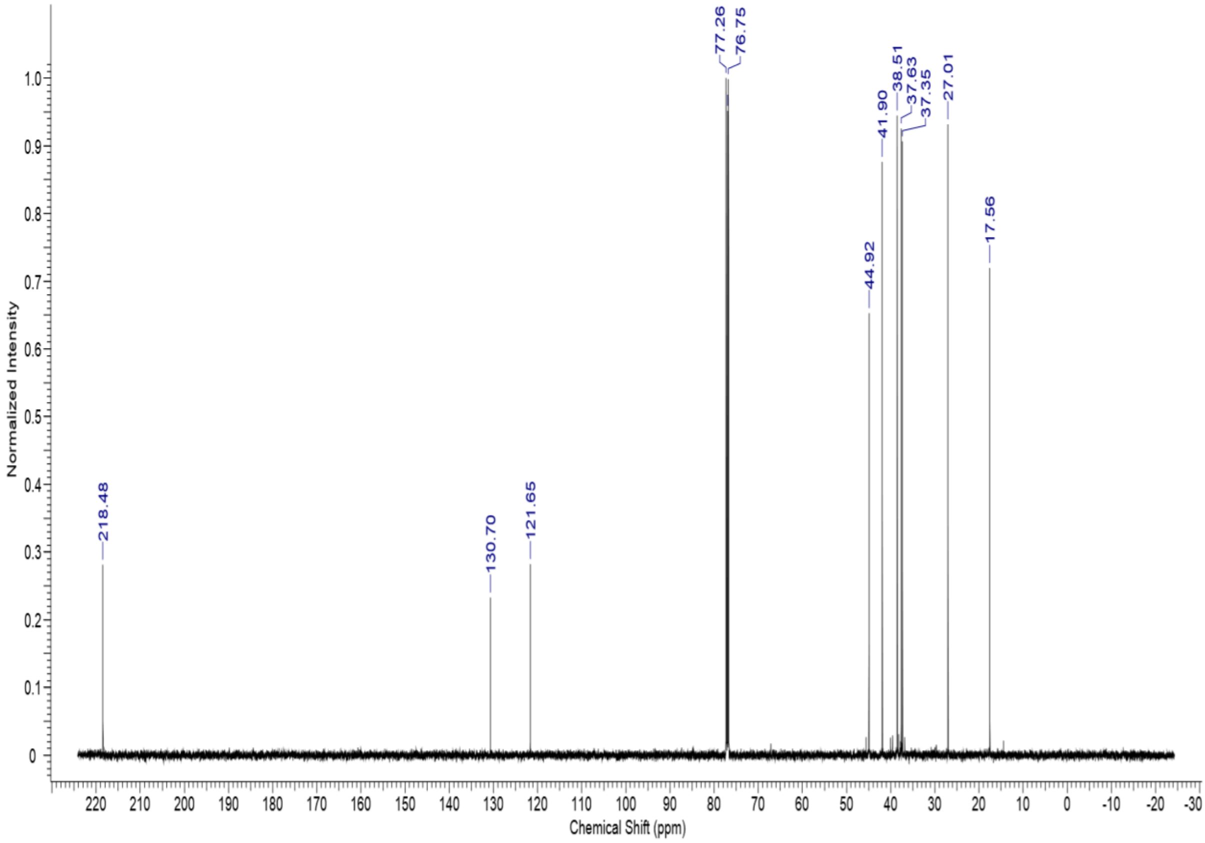

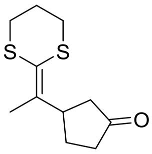

[10c] 3-(1-(1,3-Dithian-2-ylidene)ethyl)cyclopentan-1-one

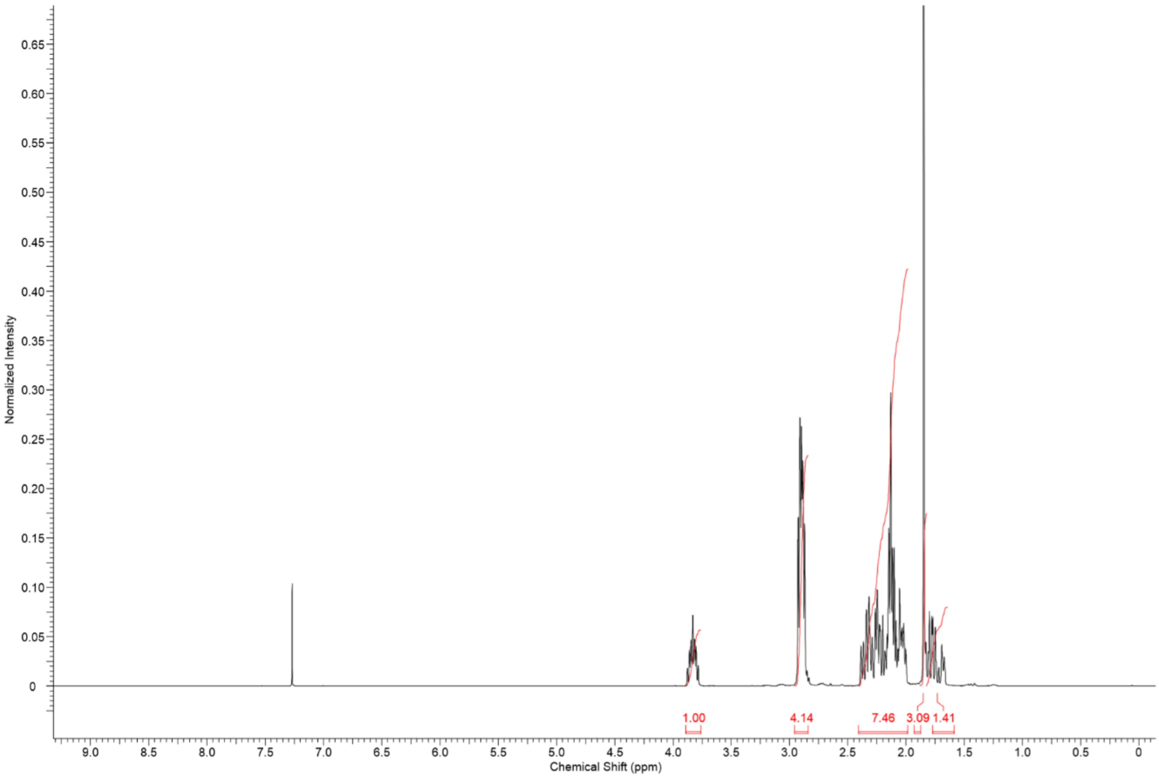

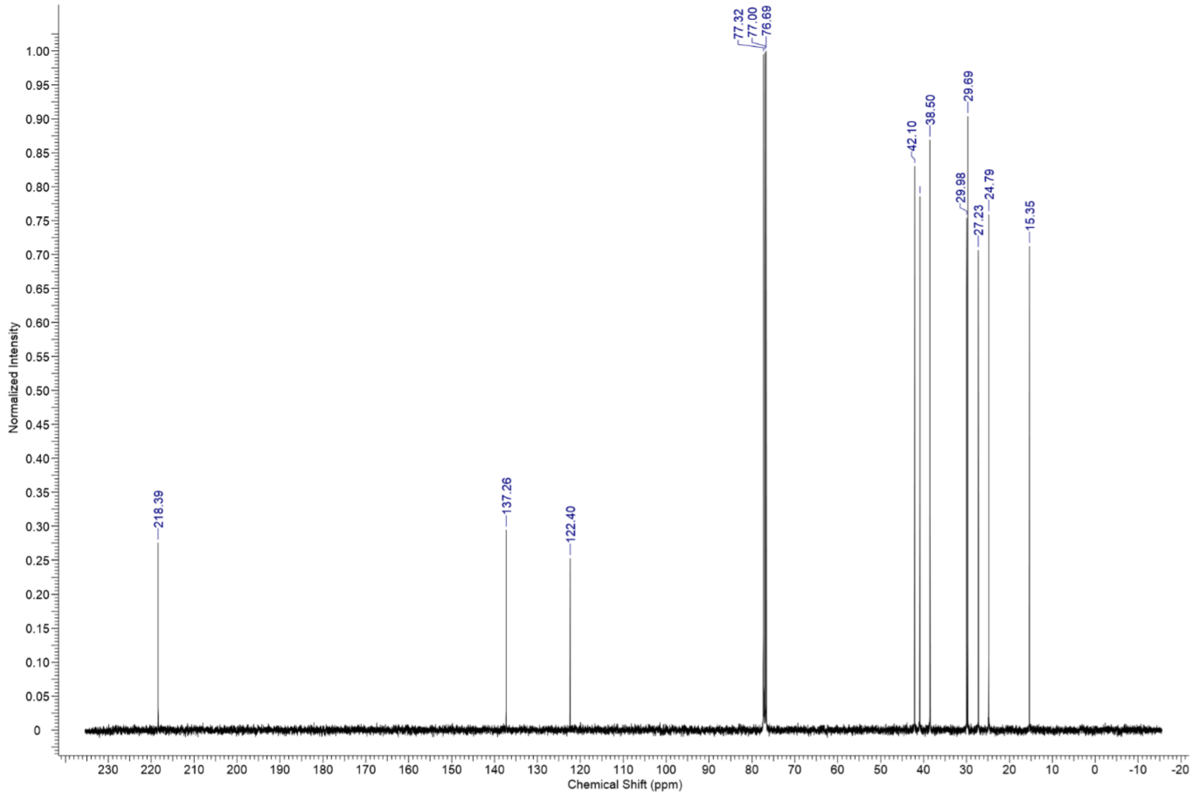

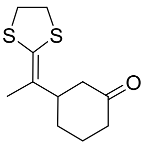

[9d] 3-(1-(1,3-dithiolan-2-ylidene)ethyl)cyclohexan-1-one

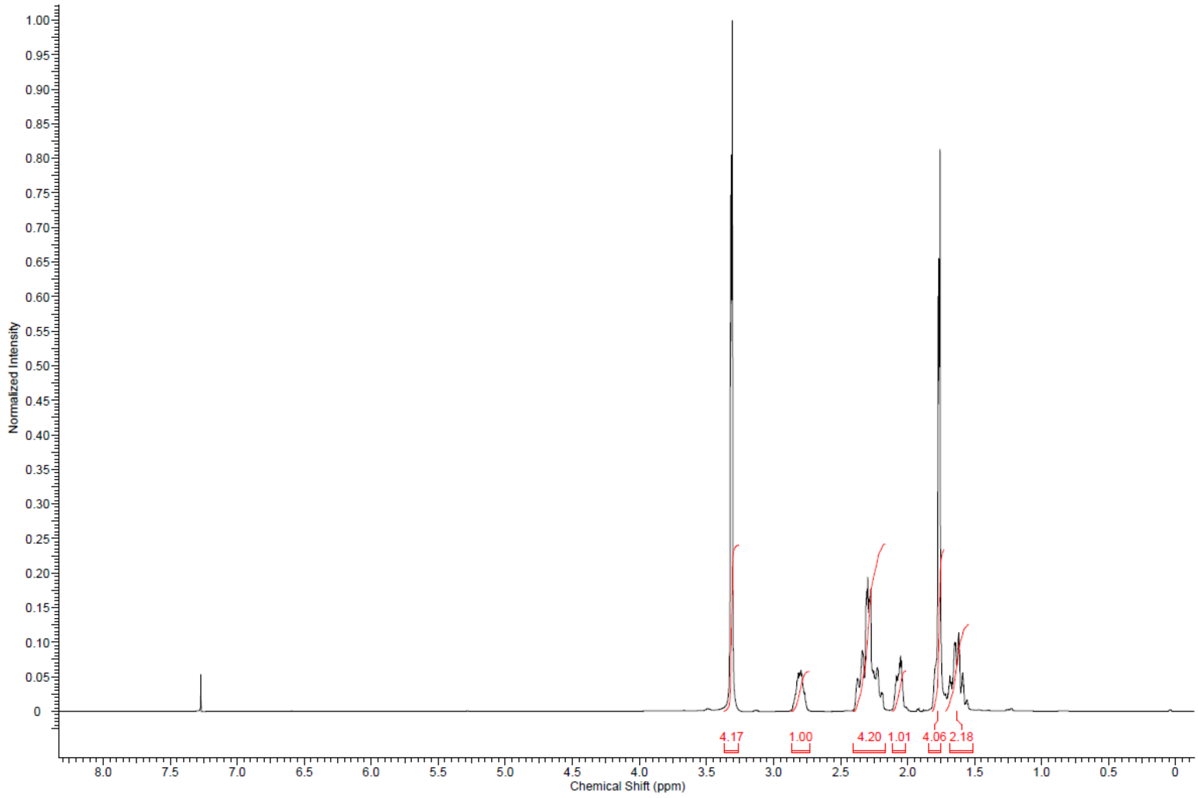

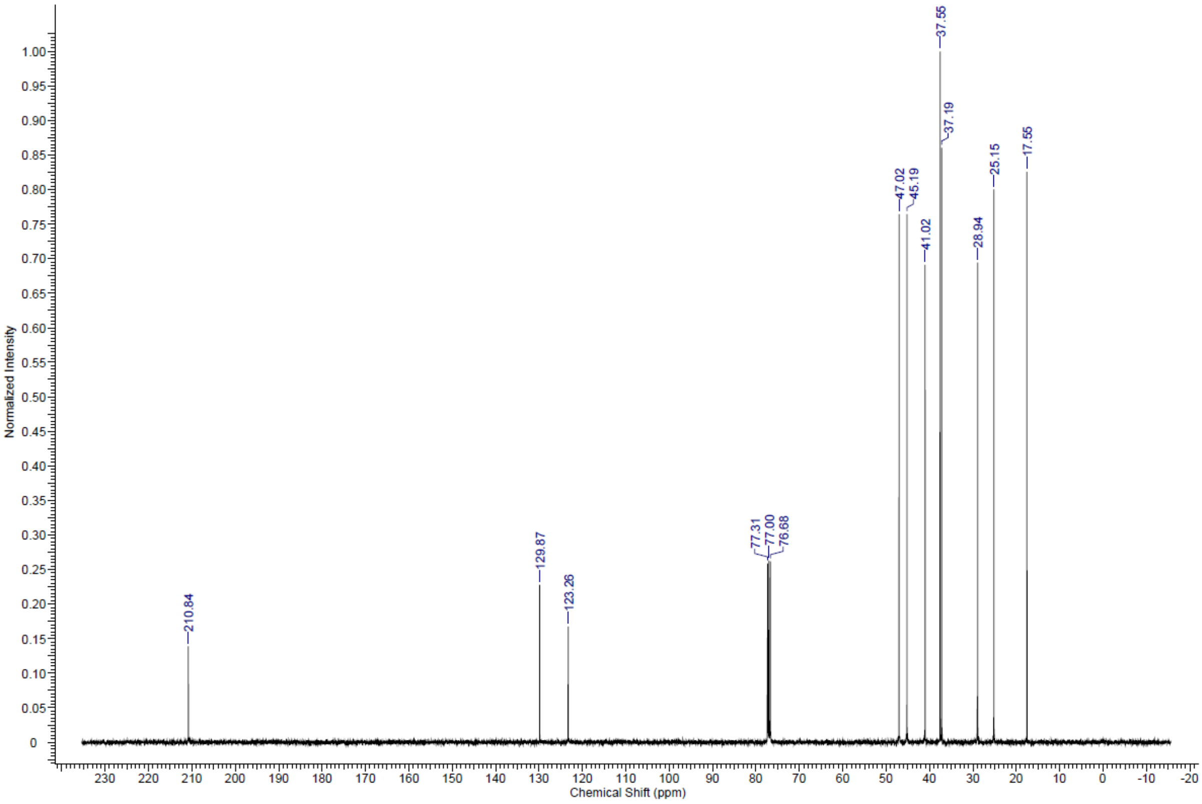

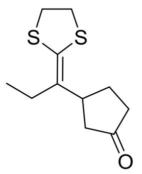

[9e] 3-(1-(1,3-dithiolan-2-ylidene)propyl)cyclopentan-1-one

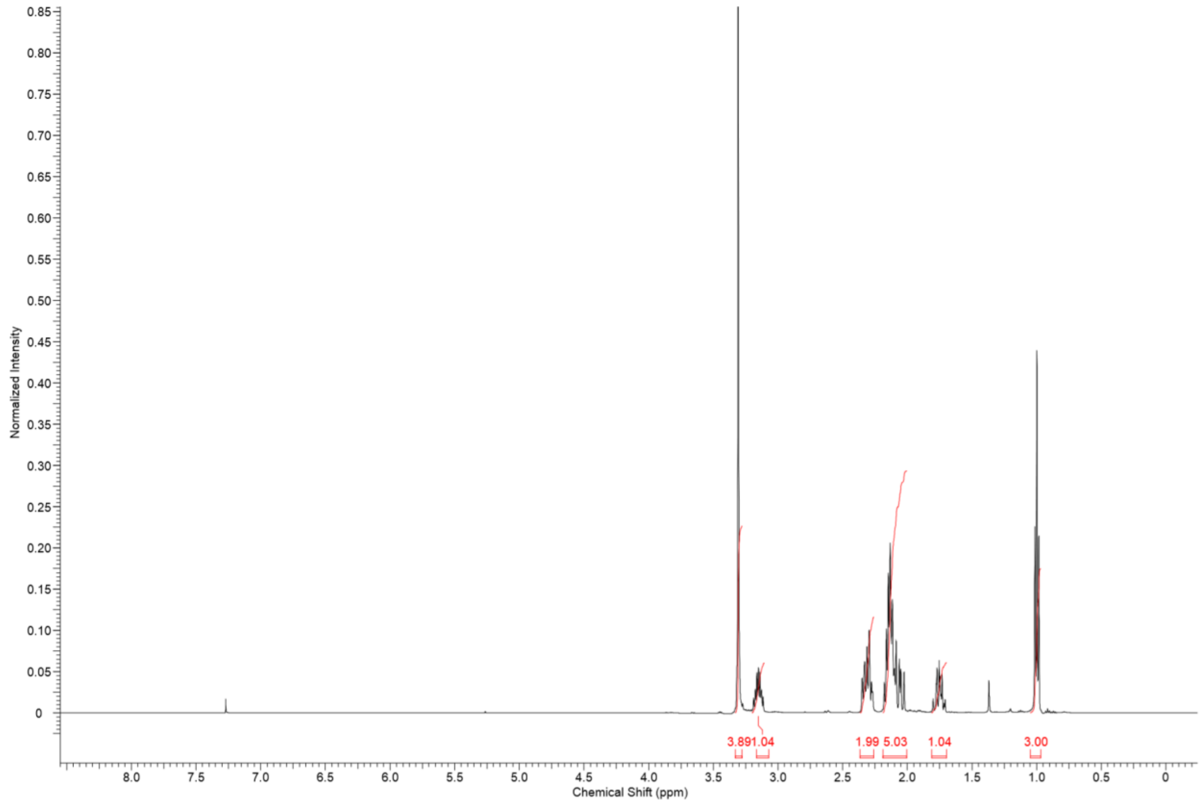

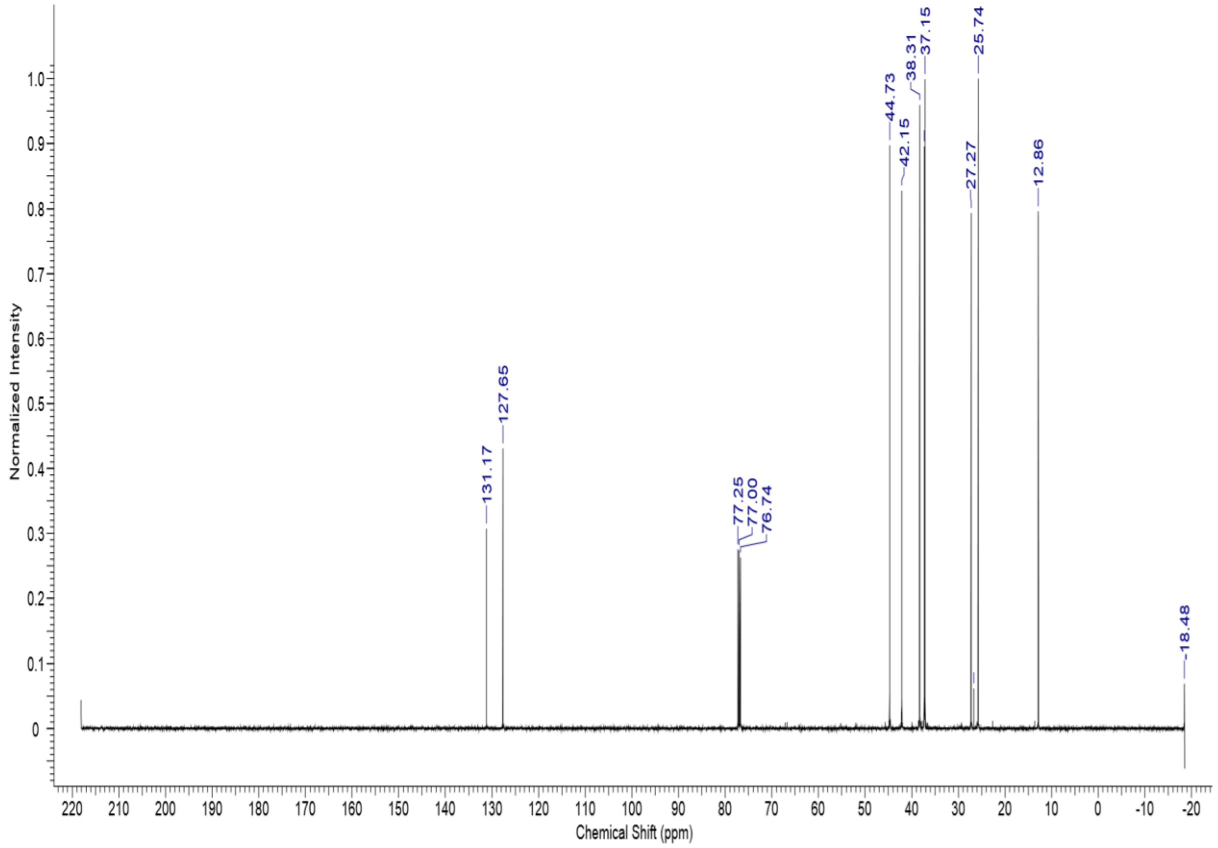

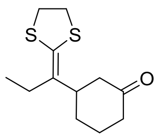

[9f] 3-(1-(1,3-dithiolan-2-ylidene)propyl)cyclohexan-1-one

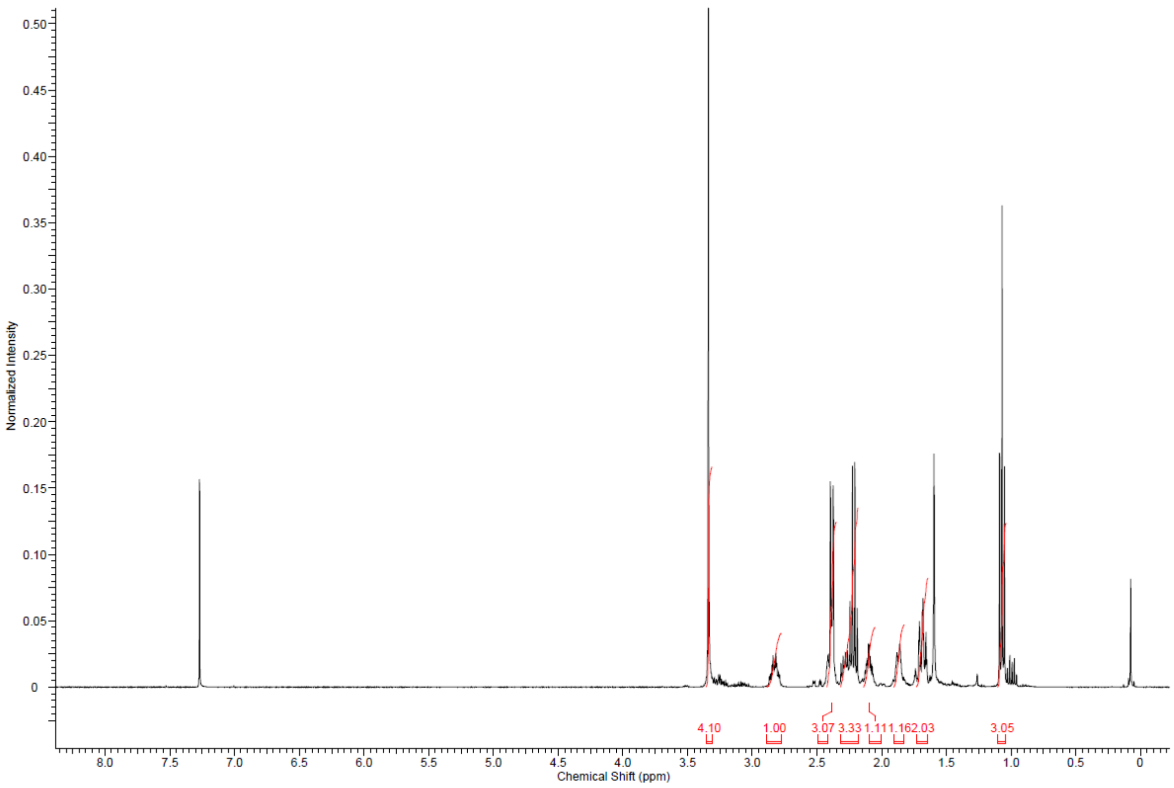

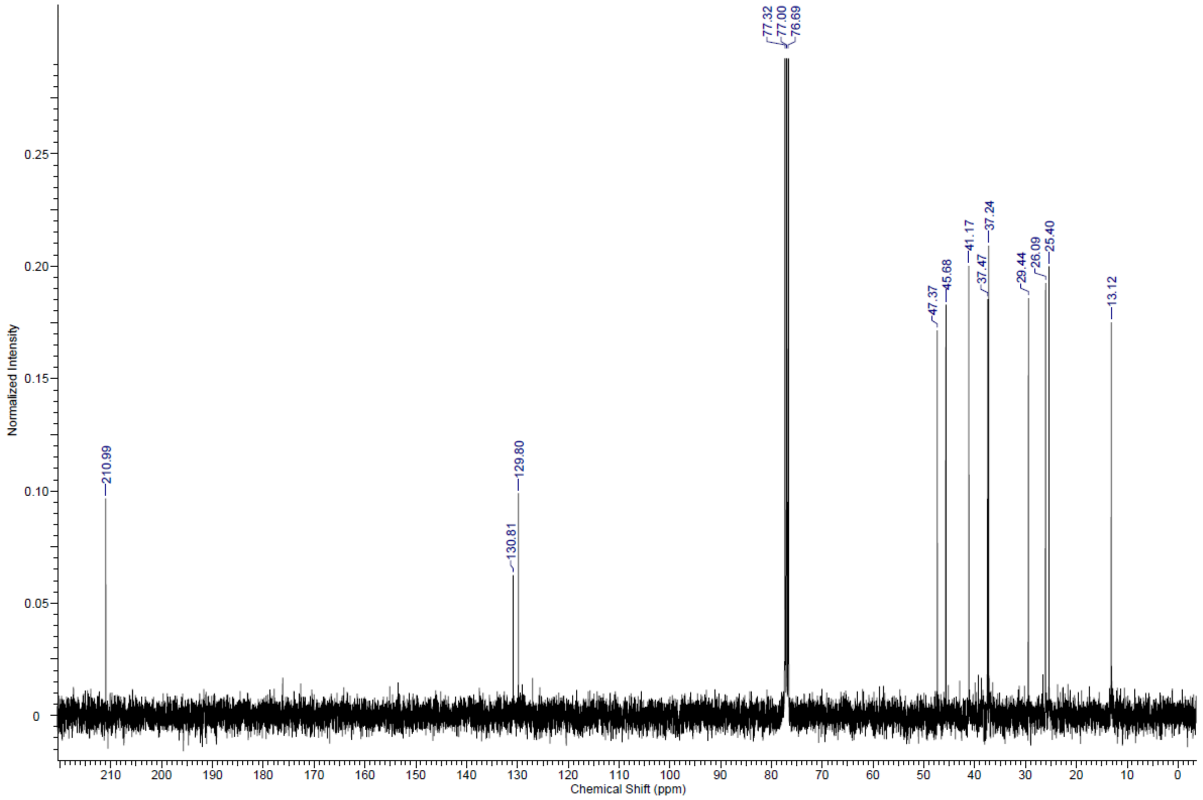

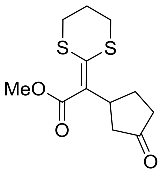

[10g] Methyl 2-(1,3-dithian-2-ylidene)-2-(3-oxocyclopentyl)acetate

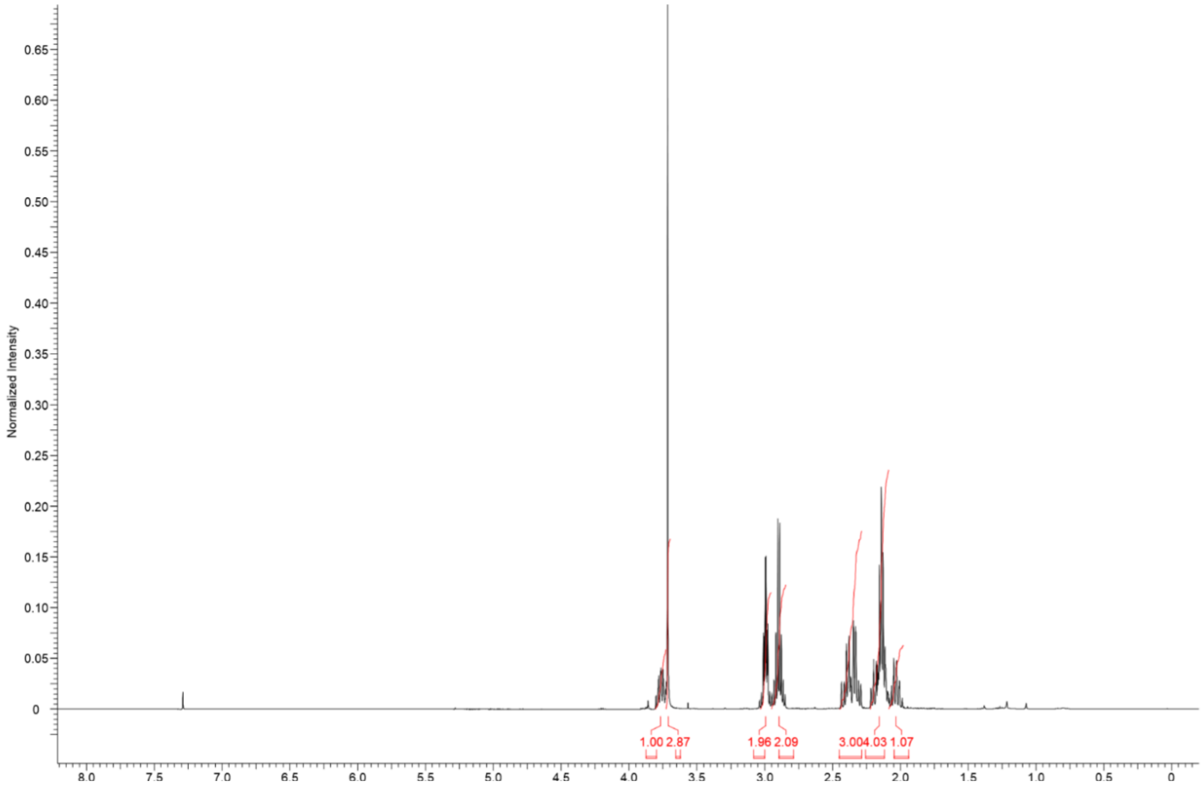

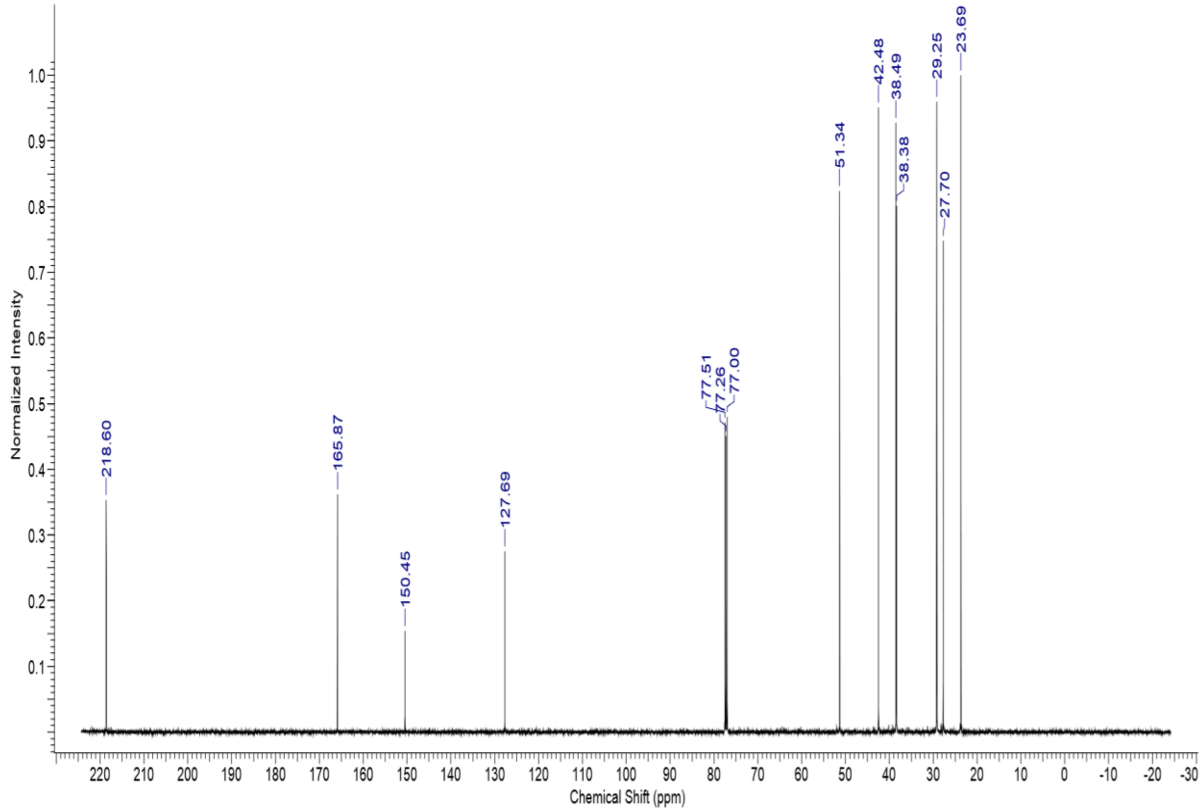

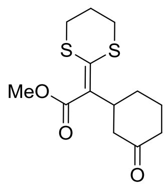

[10h] Methyl 2-(1,3-dithian-2-ylidene)-2-(3-oxocyclohexyl)acetate

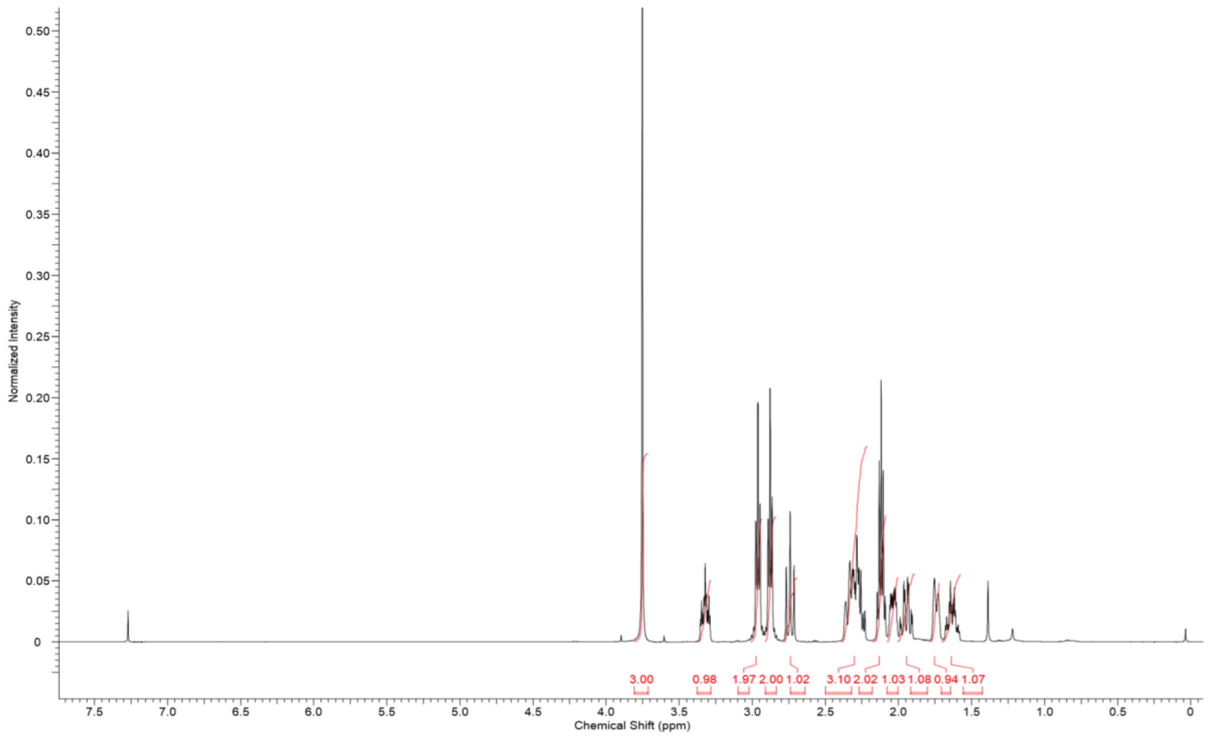

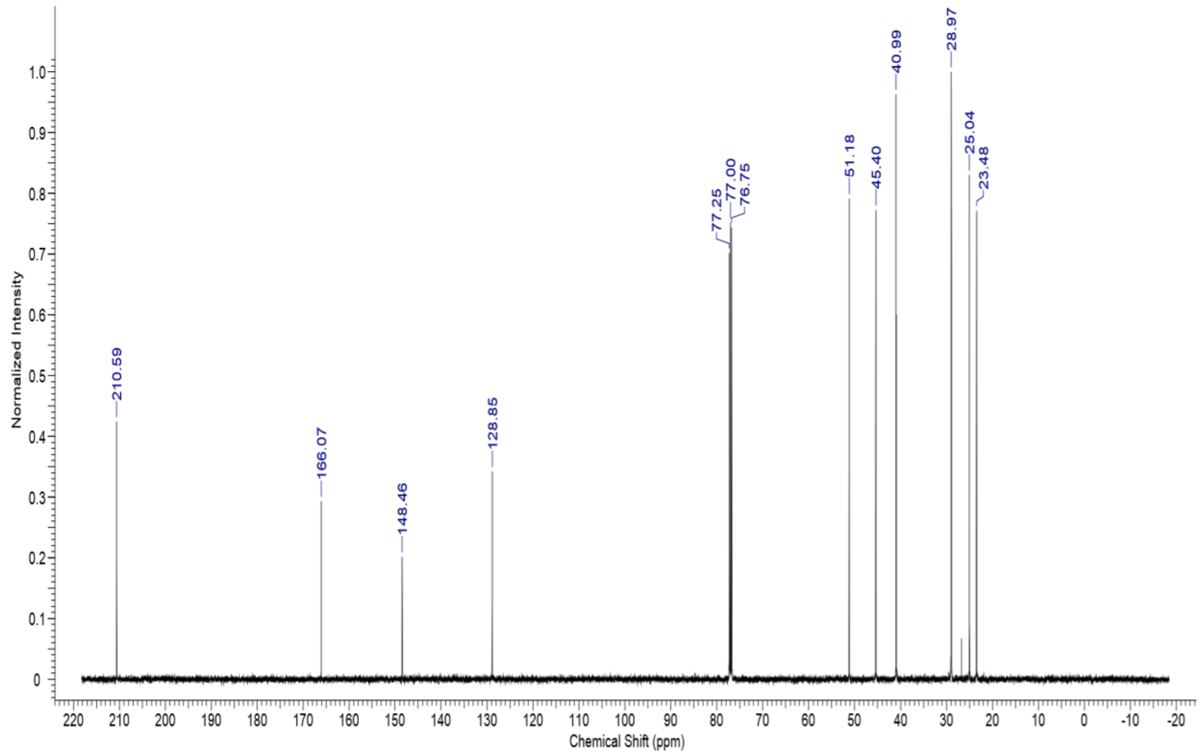

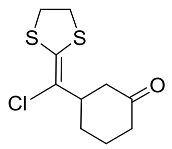

[9i] 3-(Chloro(1,3-dithiolan-2-ylidene)methyl)cyclohexan-1-one

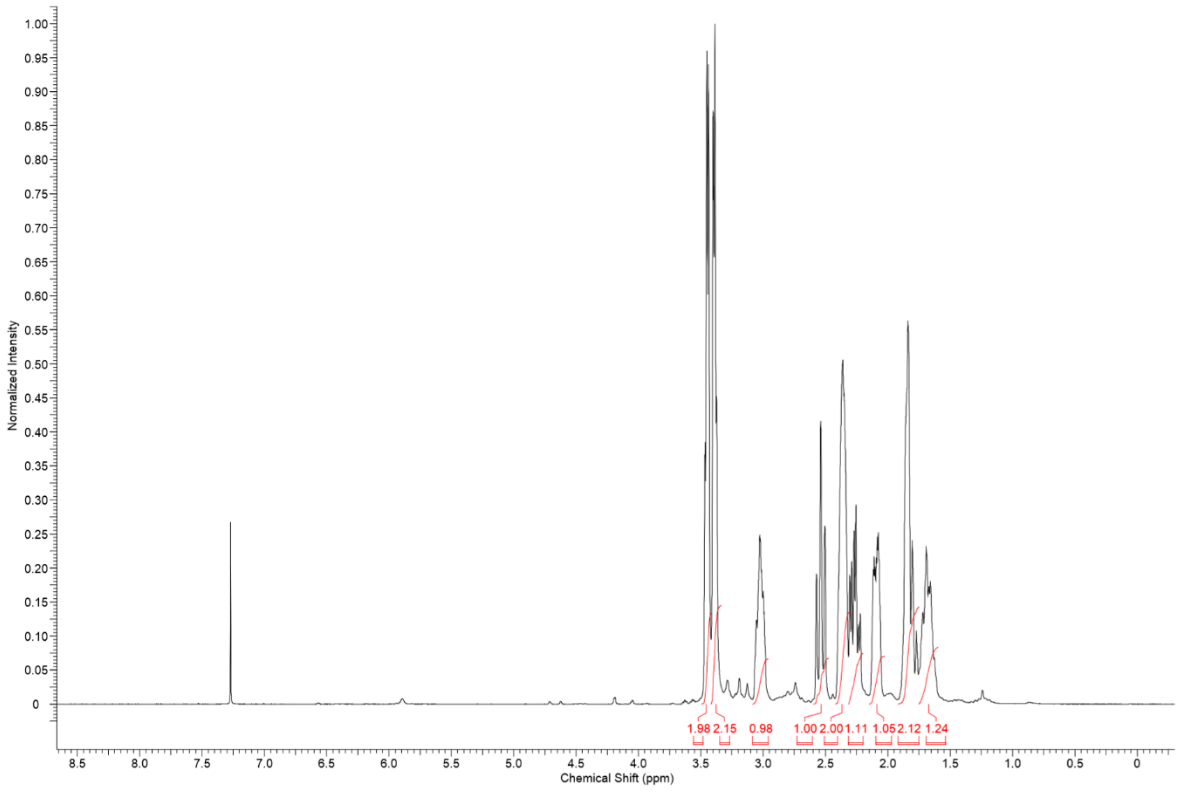

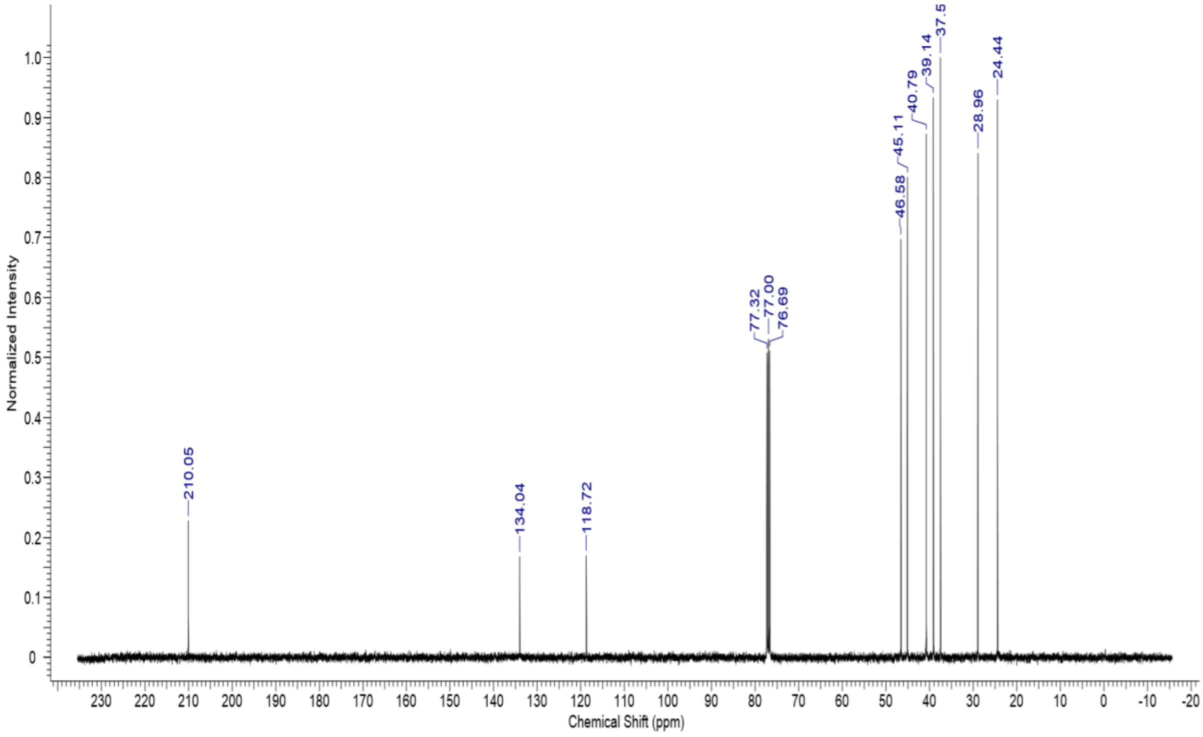

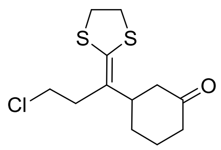

[9j] 3-(3-Chloro-1-(1,3-dithiolan-2-ylidene)propyl)cyclohexan-1-one

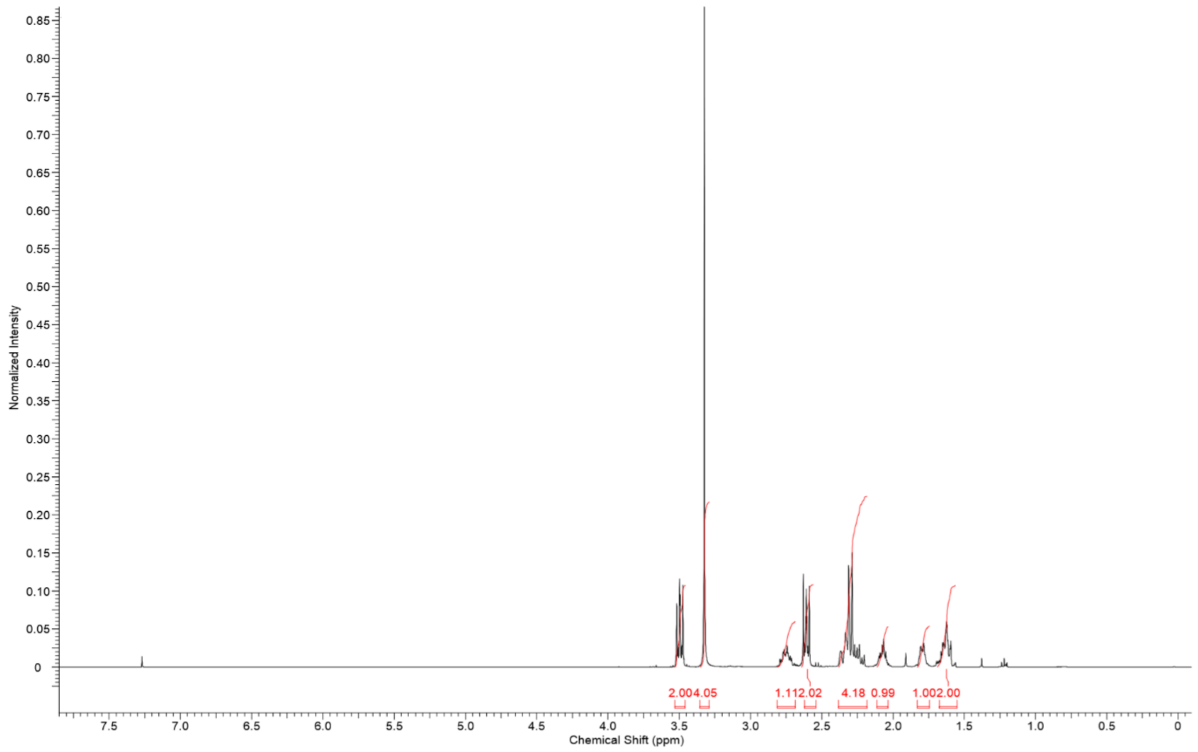

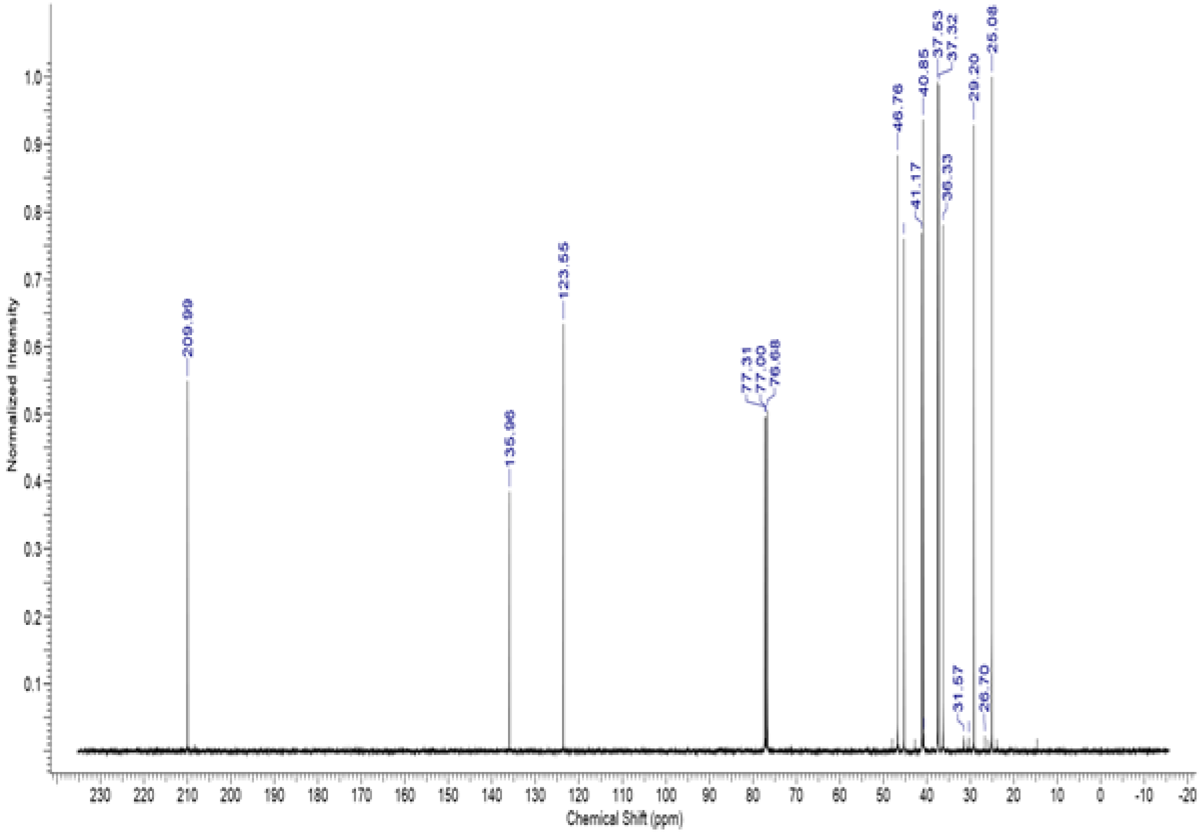

Spectra of compounds type **11a** and **11b**

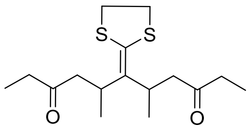

[11a] 6-(1,3-dithiolan-2-ylidene)-5,7-dimethylundecane-3,9-dione

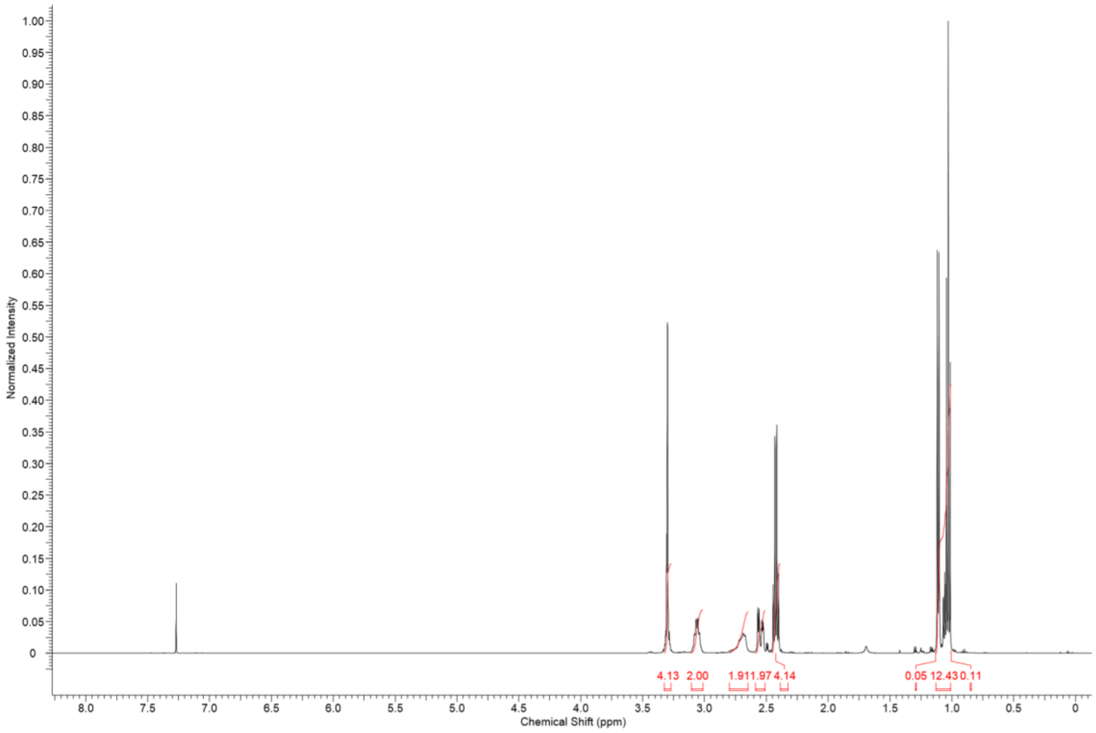

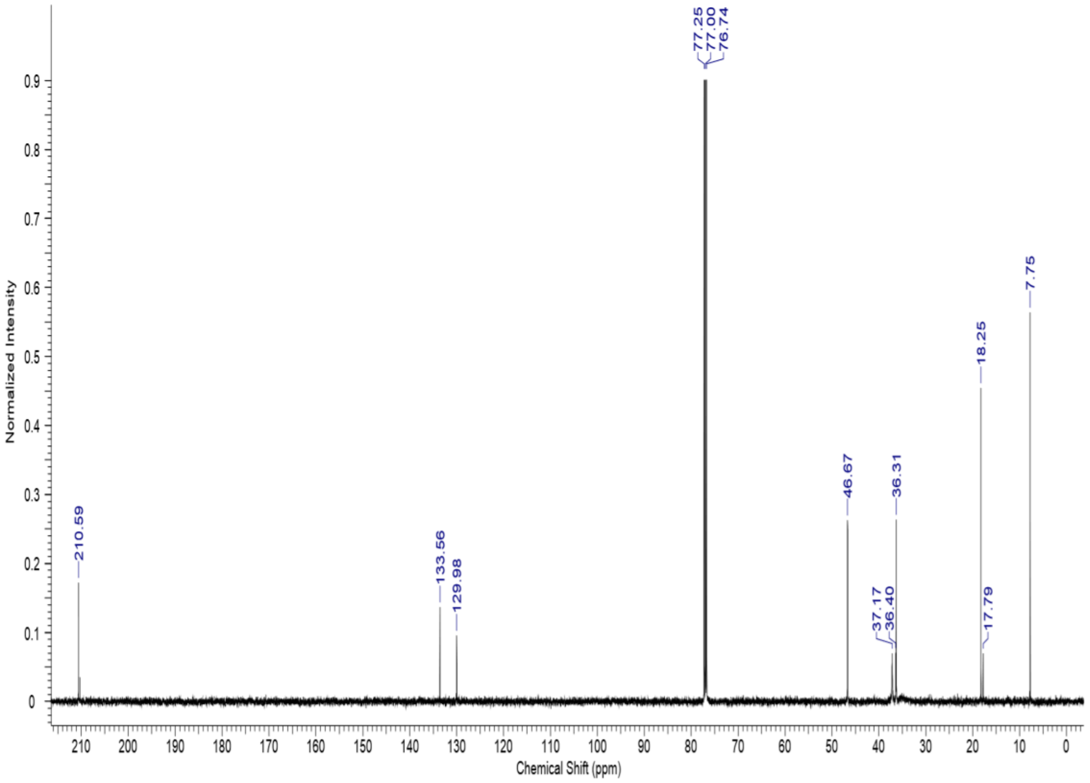

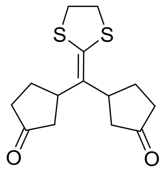

[11b] 3,3'-((1,3-dithiolan-2-ylidene)methylene)bis(cyclopentan-1-one)

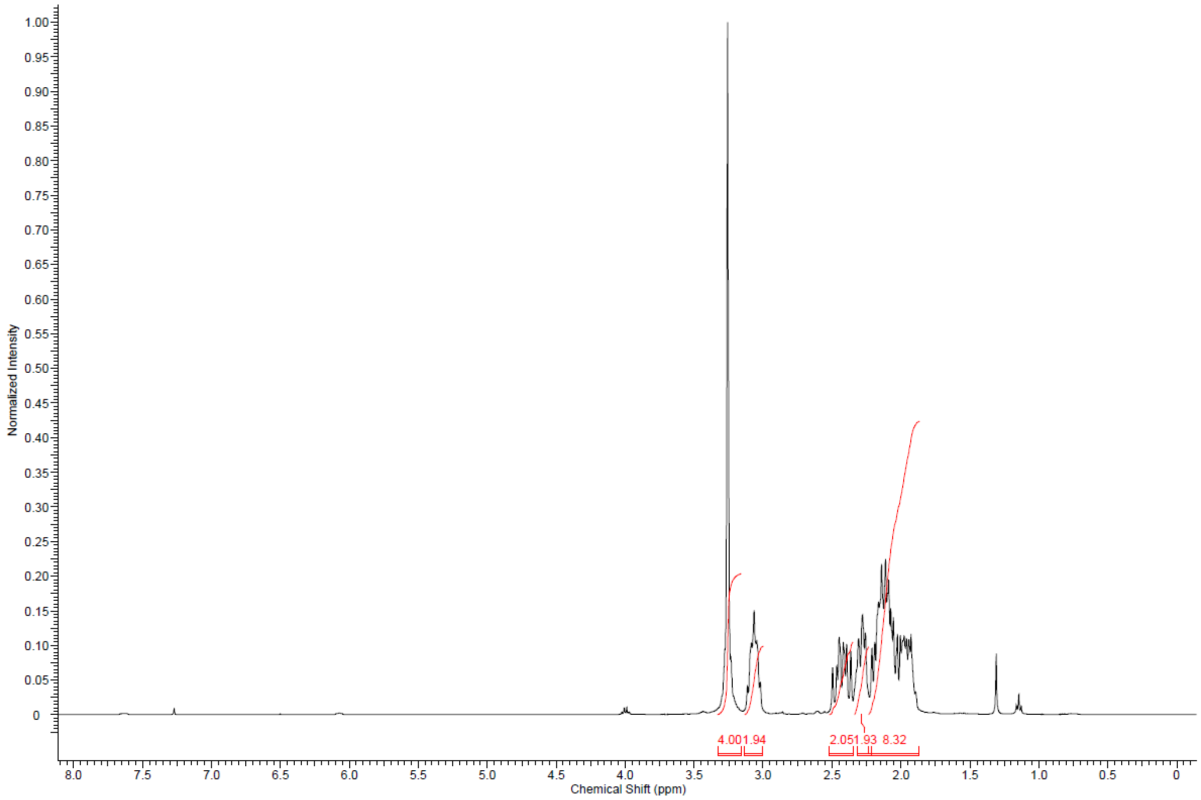

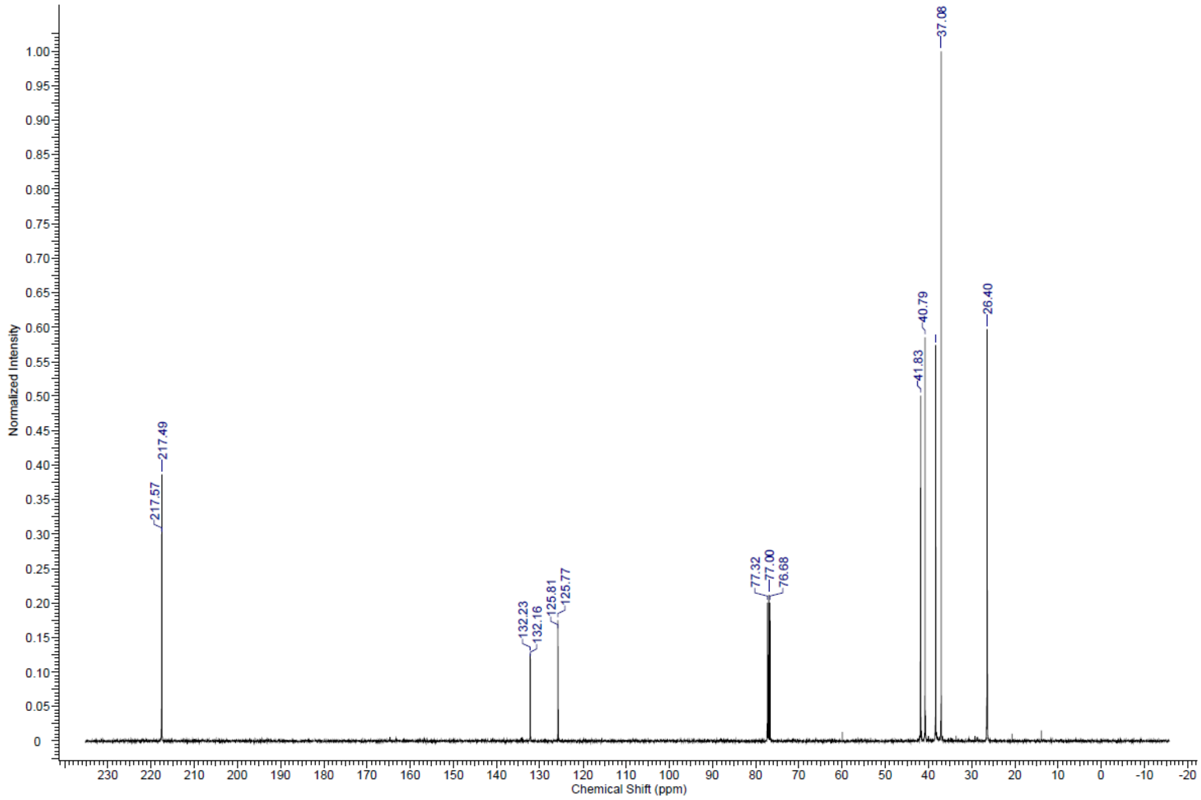

Spectra of compounds **13a**, **13b**, **15**, **16a**, **16b** and **18a** and **18b**

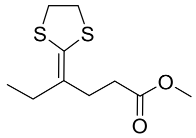

[13a] Methyl 4-(1,3-dithiolan-2-ylidene)hexanoate

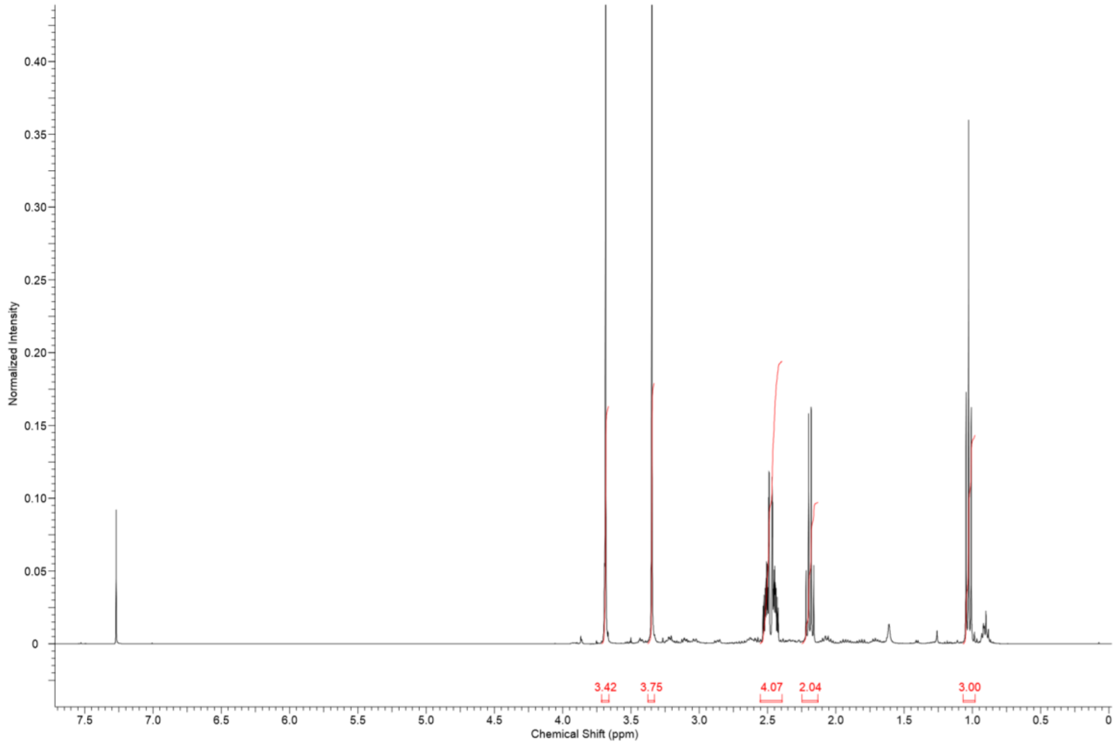

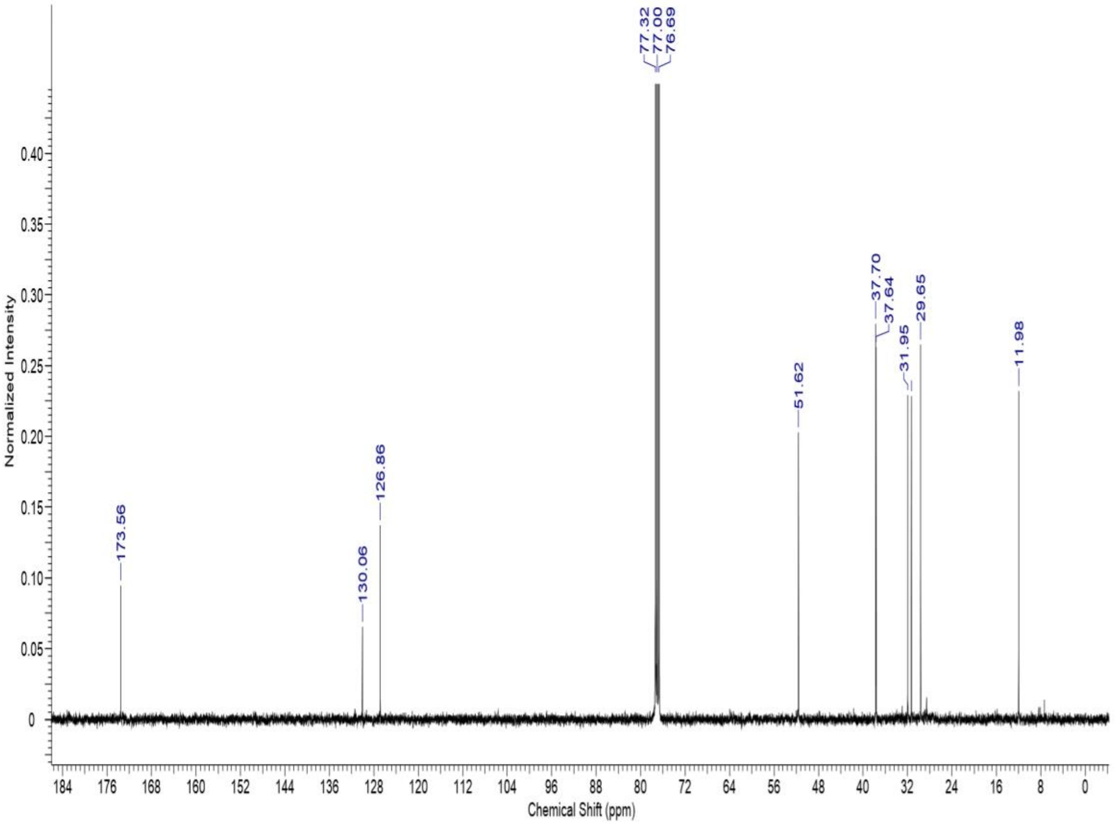

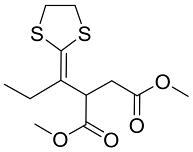

[13b] Dimethyl 2-(1-(1,3-dithiolan-2-ylidene)propyl)succinate

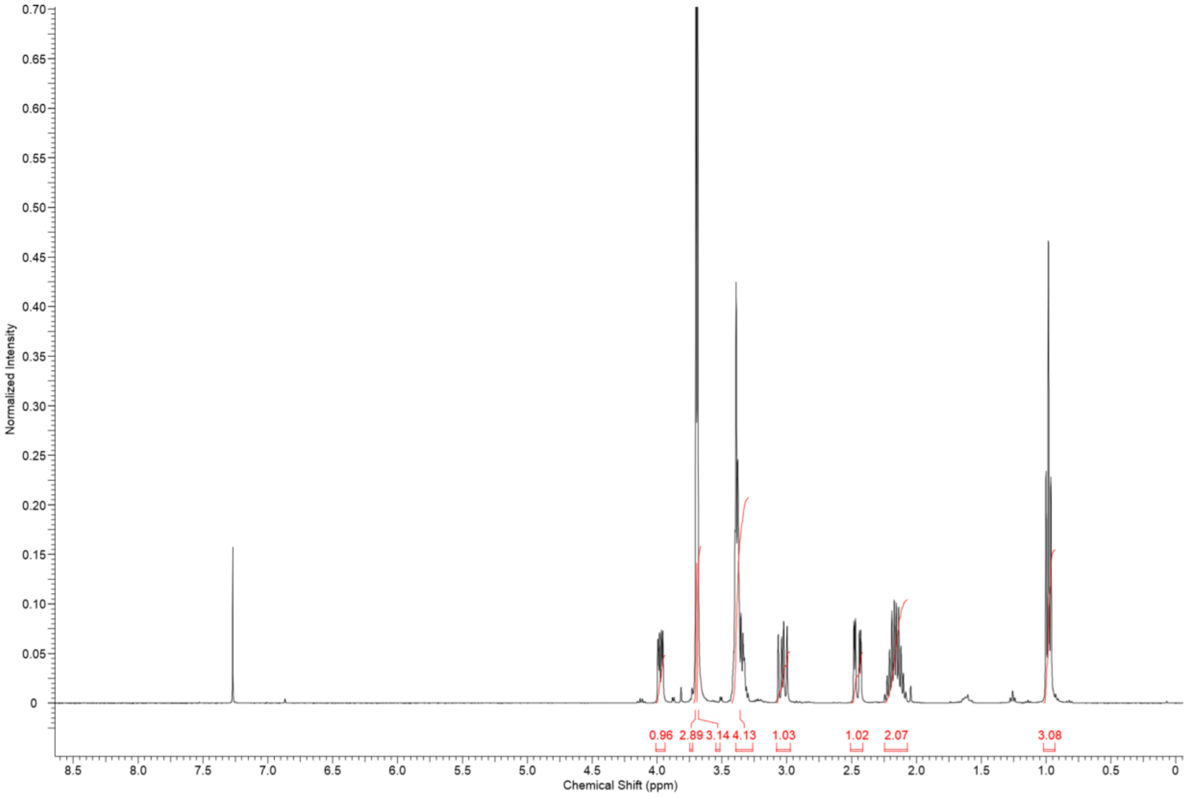

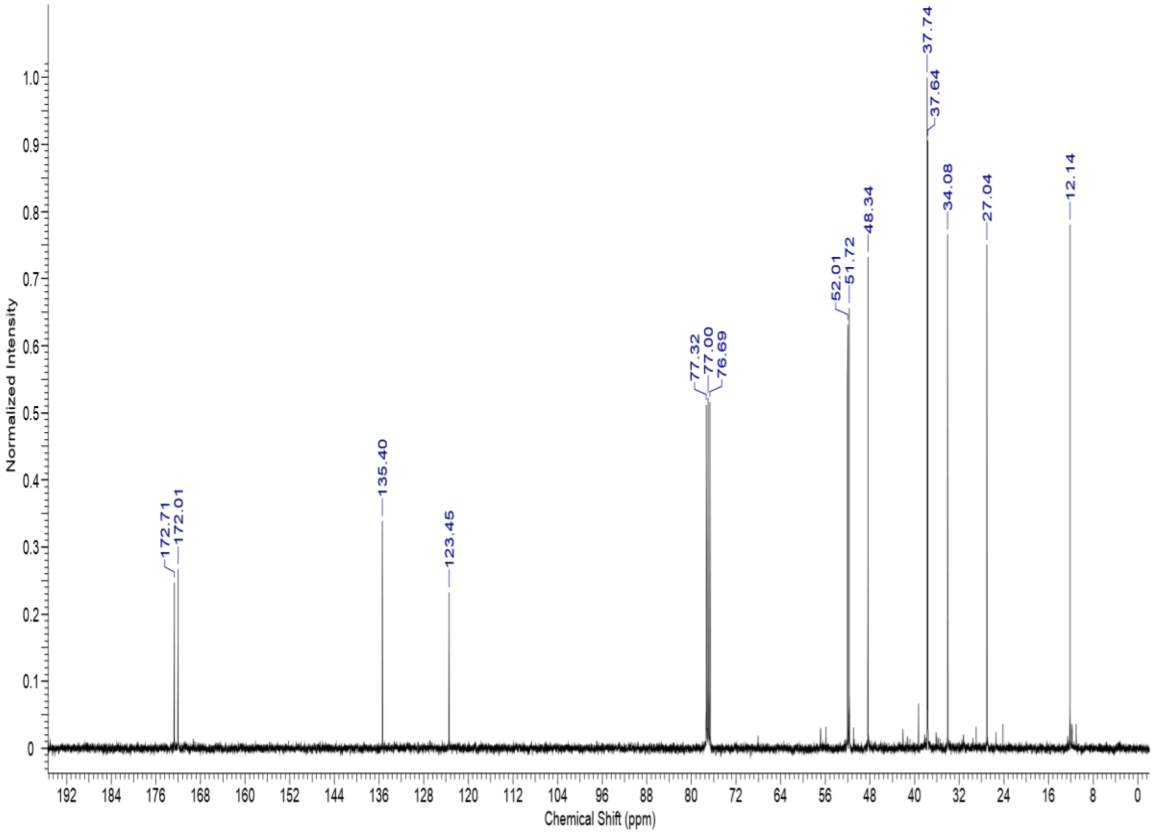

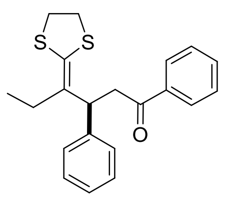

[15] 4-(1,3-dithiolan-2-ylidene)-1,3-diphenylhexan-1-one

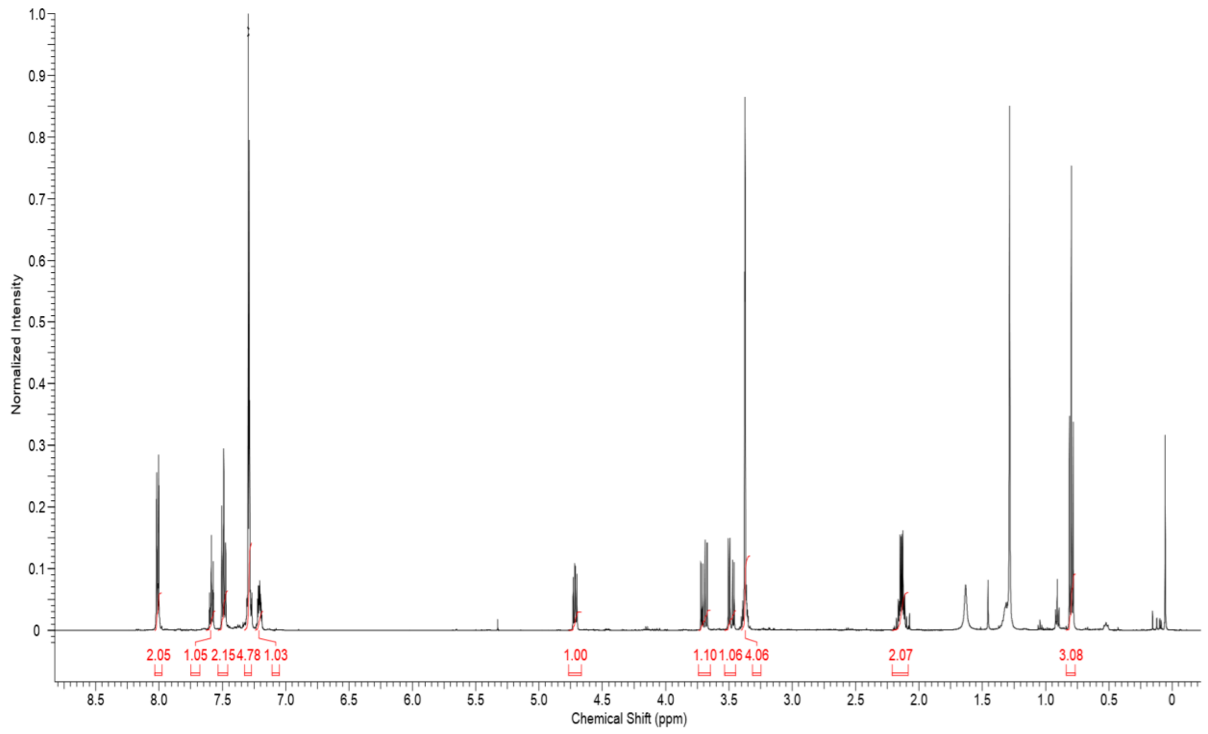

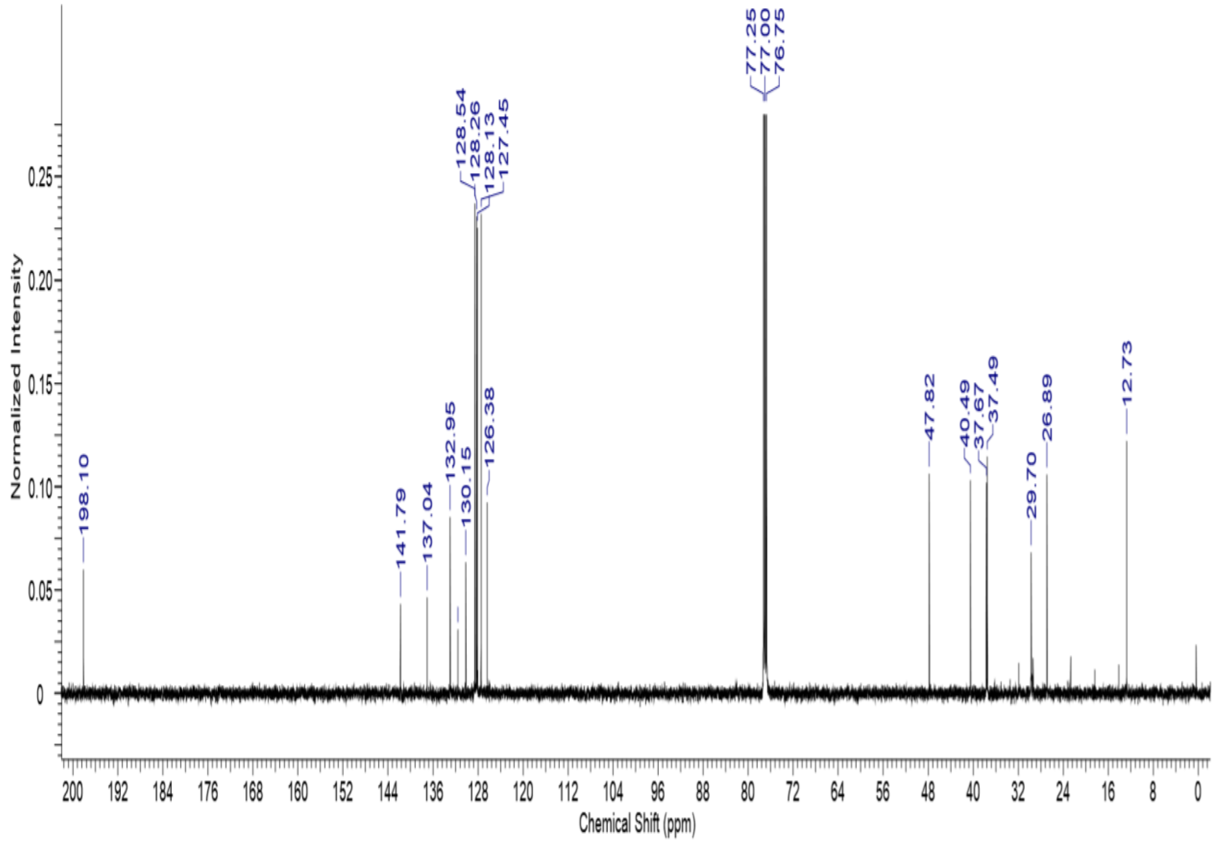

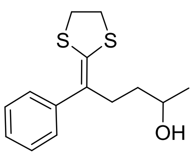

[16a] 5-(1,3-Dithiolan-2-ylidene)-5-phenylpentan-2-ol

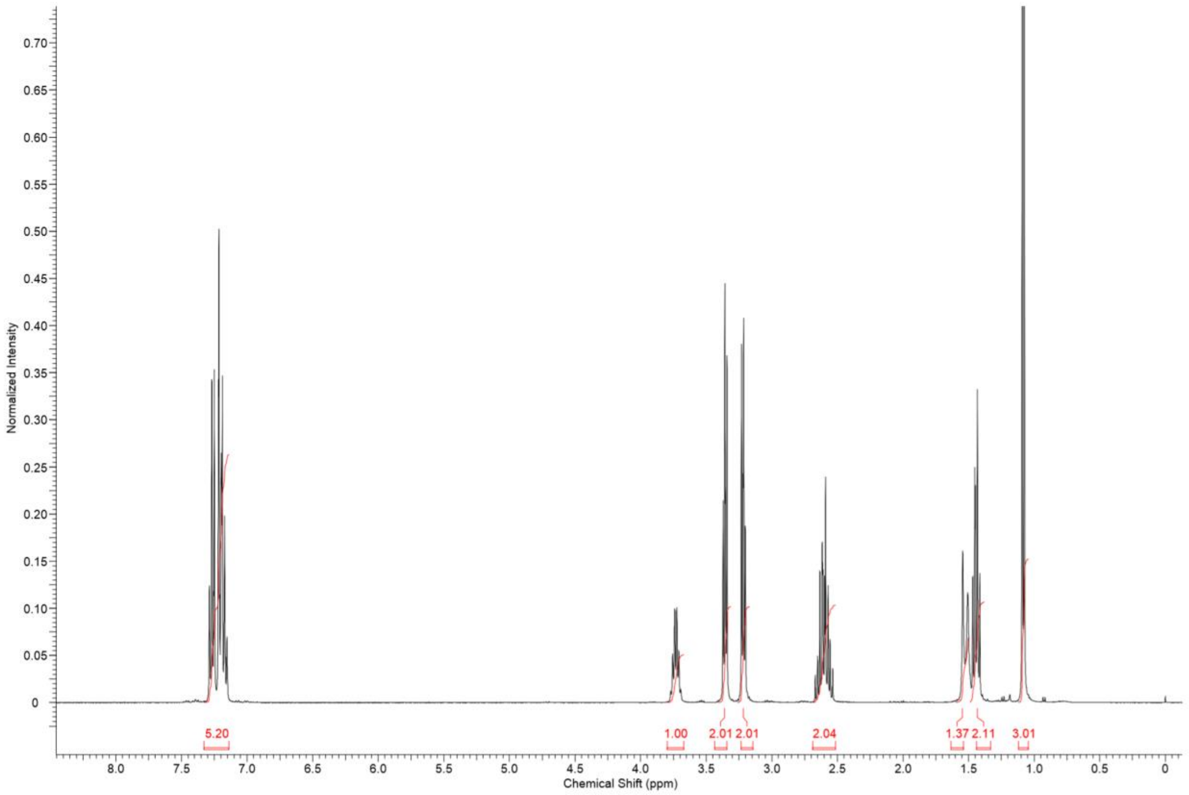

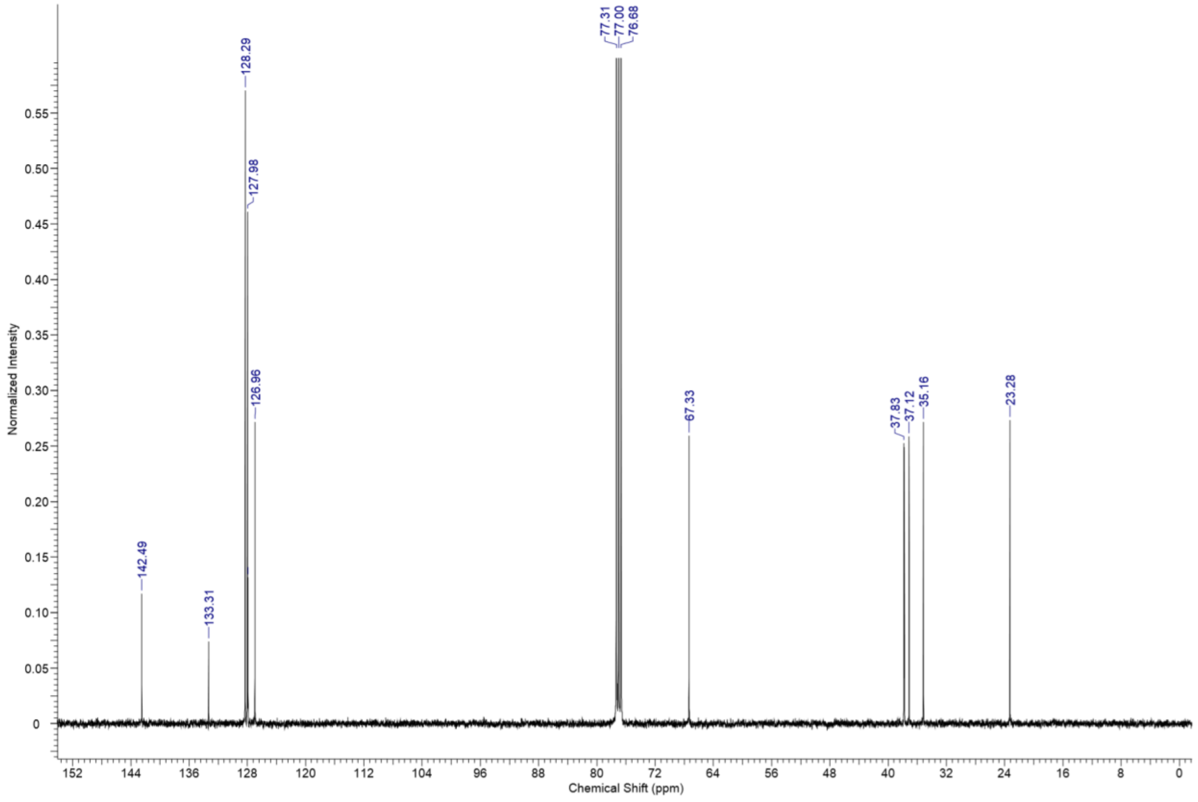

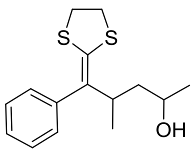

[16b] 5-(1,3-dithiolan-2-ylidene)-4-methyl-5-phenylpentan-2-ol, 2 diastereomers

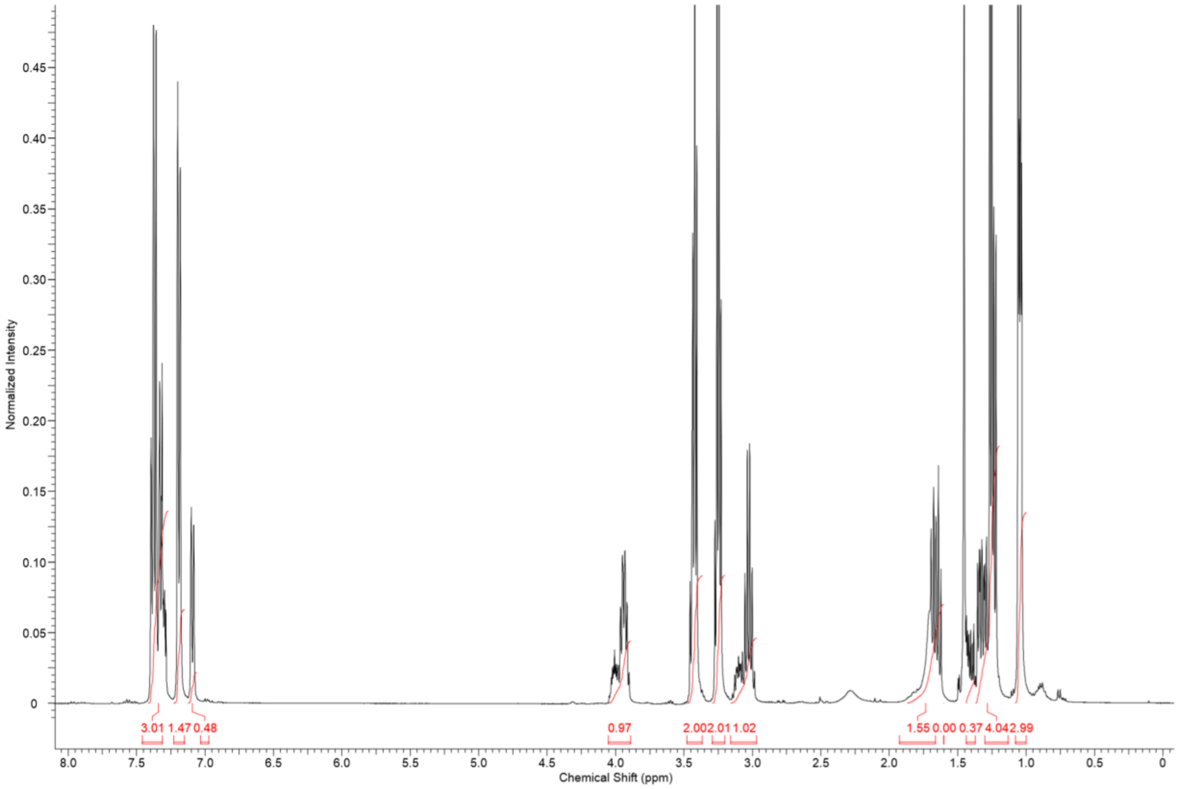

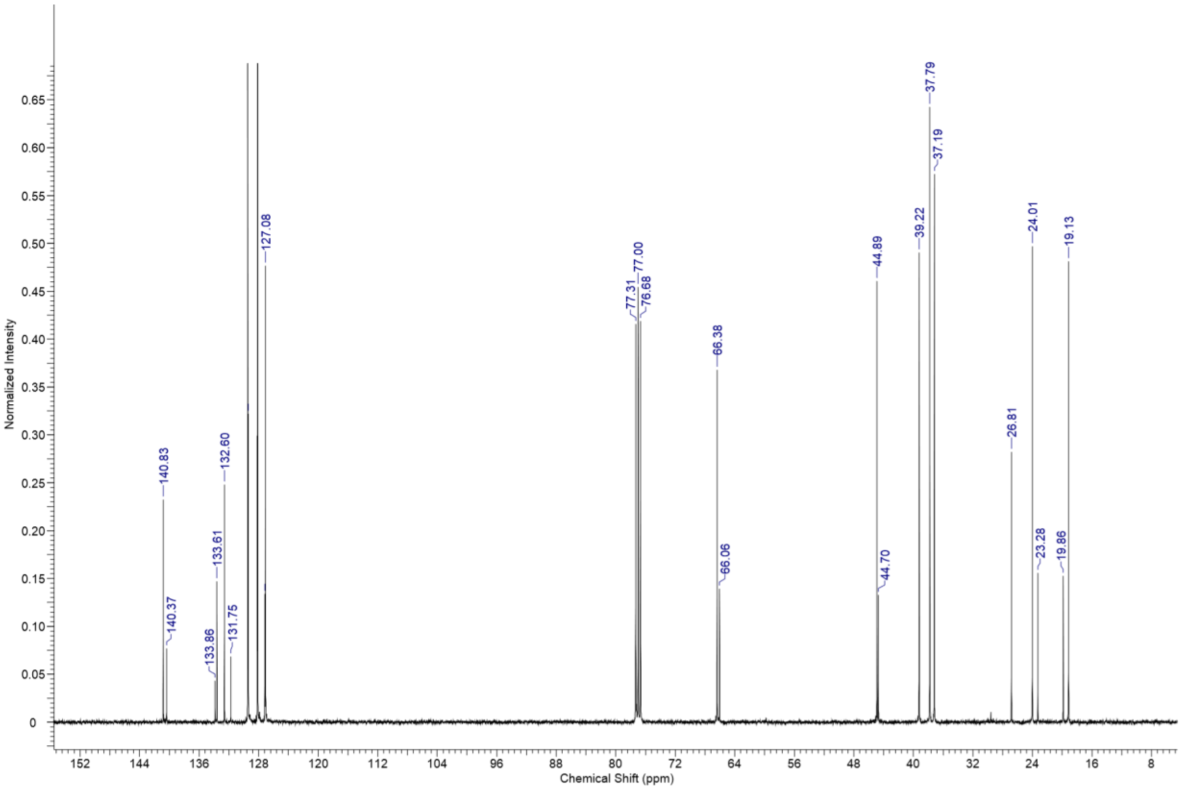

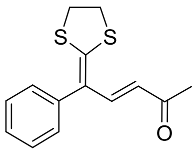

[**18a**] (E)- 5-(1,3-Dithiolan-2-ylidene)-5-phenylpent-3-en-2-one

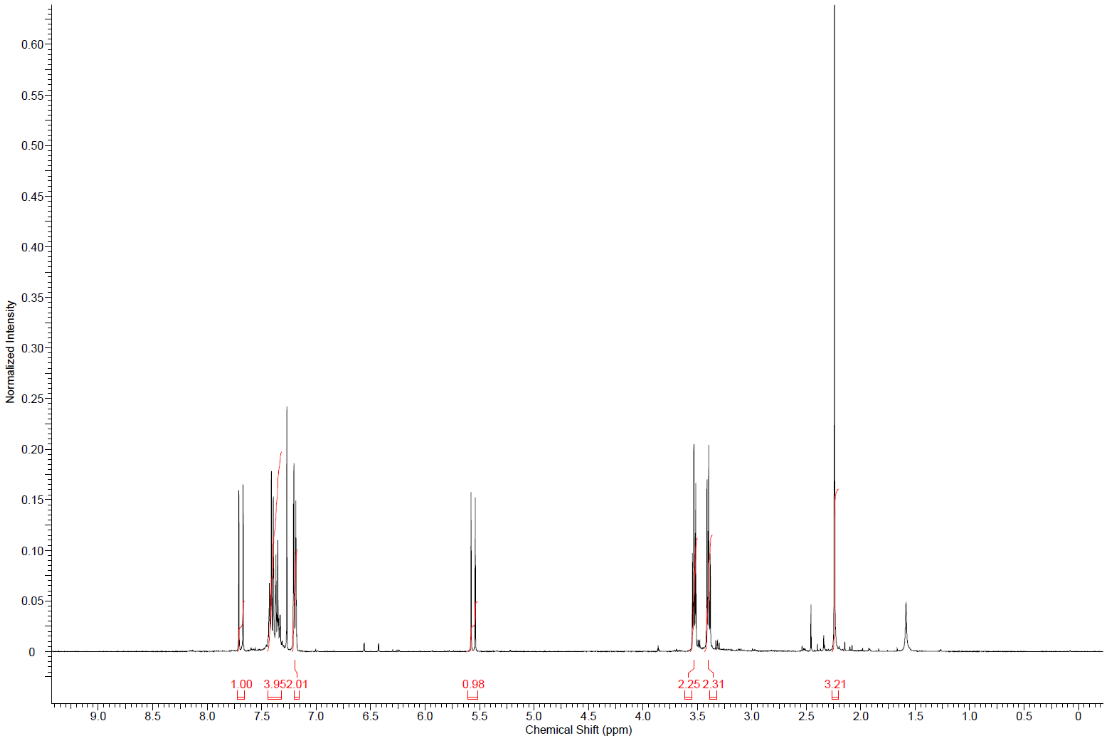

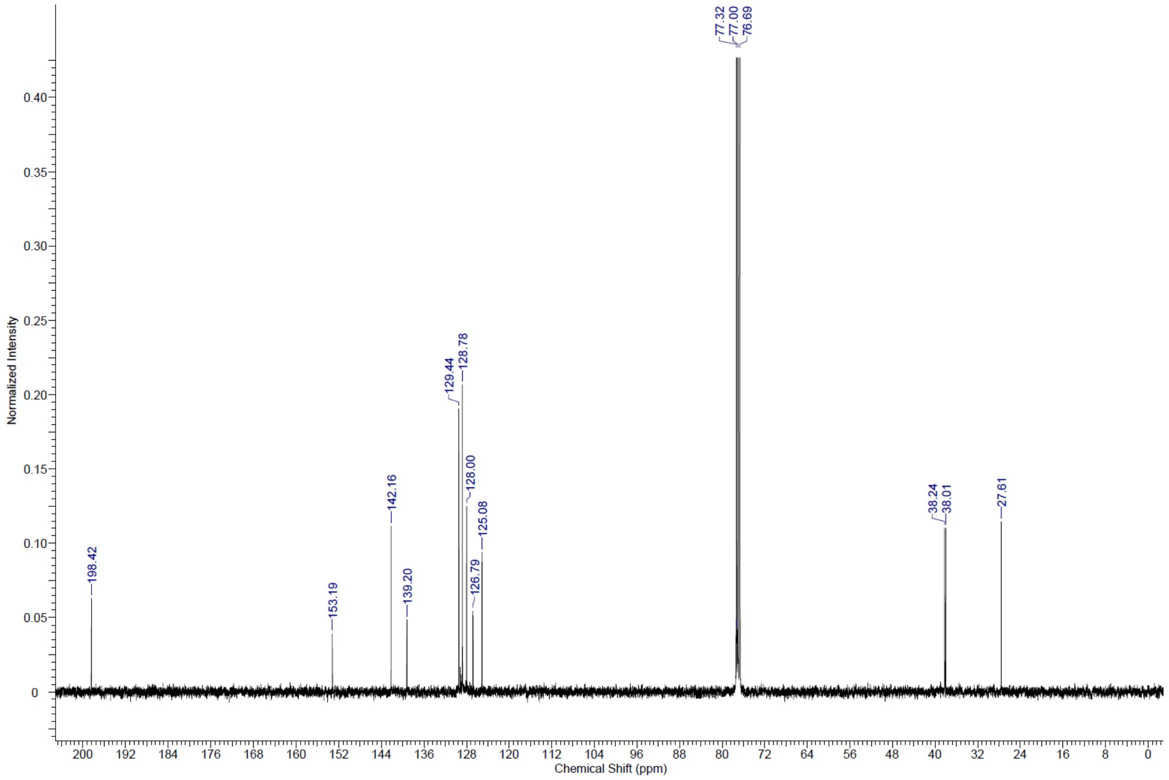

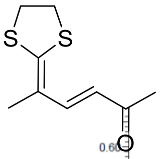

[**18b**] (E)-5-(1,3-Dithiolan-2-ylidene)hex-3-en-2-one

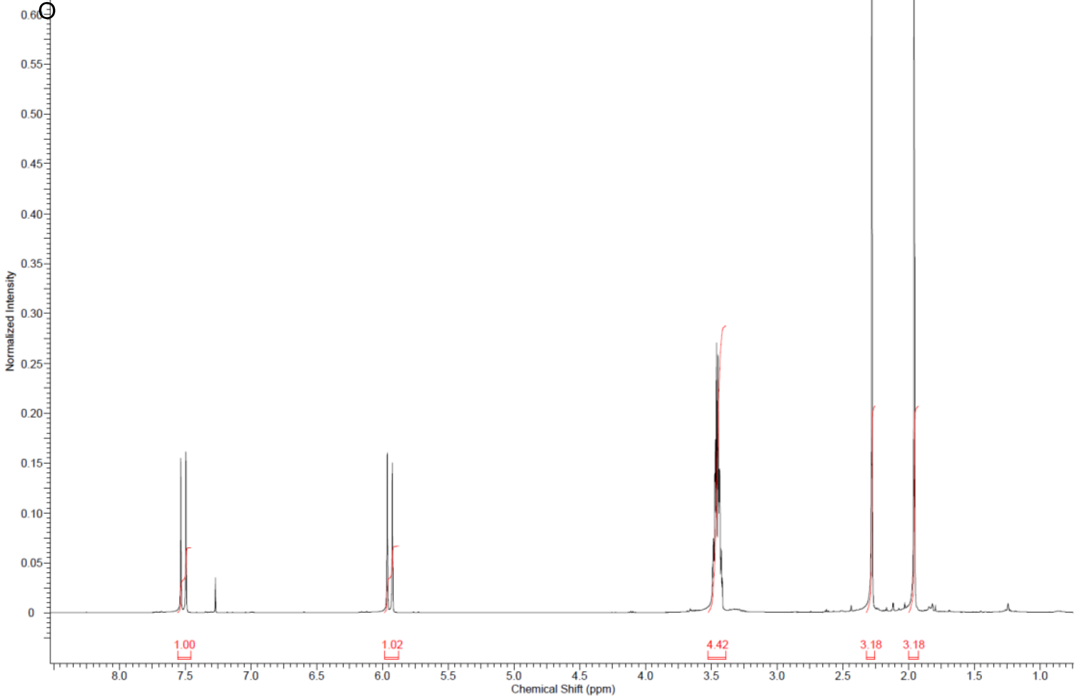

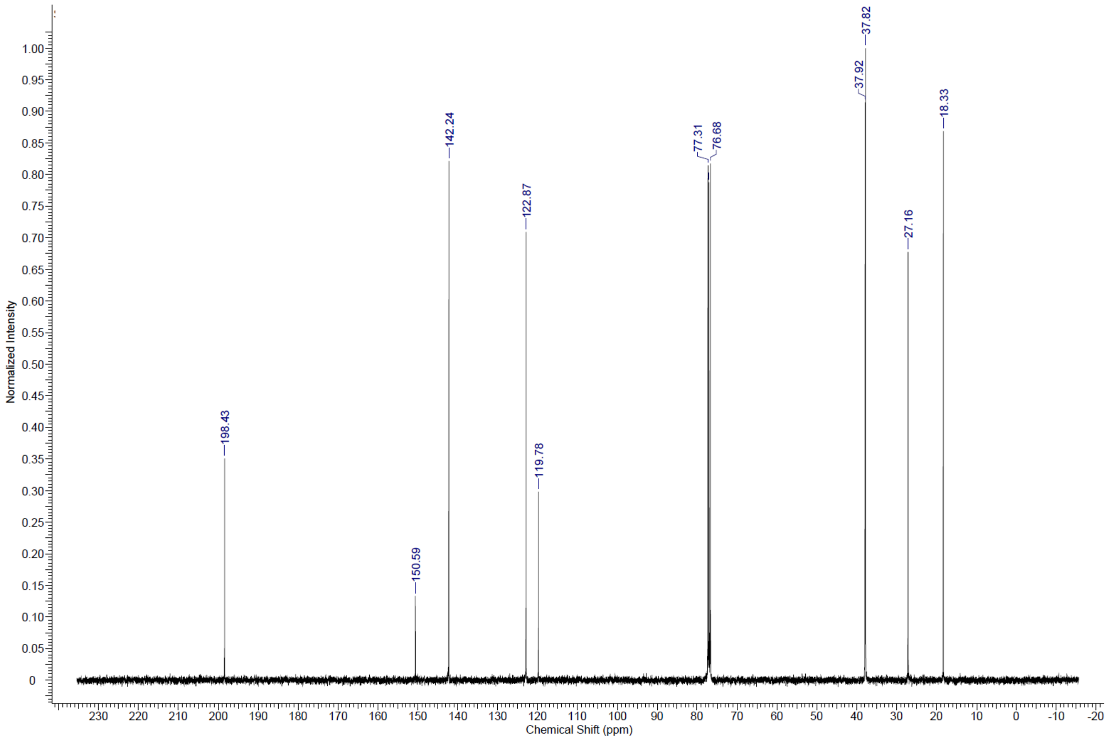

Products of type **7**: ring opening by hydrolysis

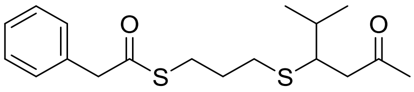

[7e] S-(3-((2-methyl-5-oxohexan-3-yl)thio)propyl) 2-phenylethanethioate

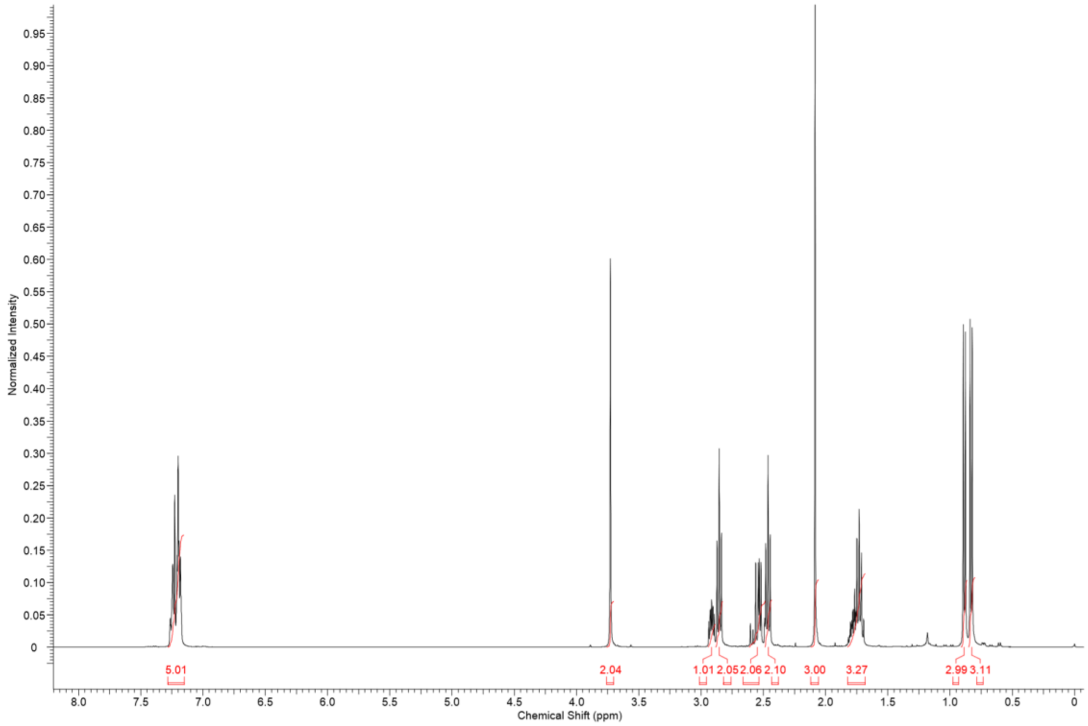

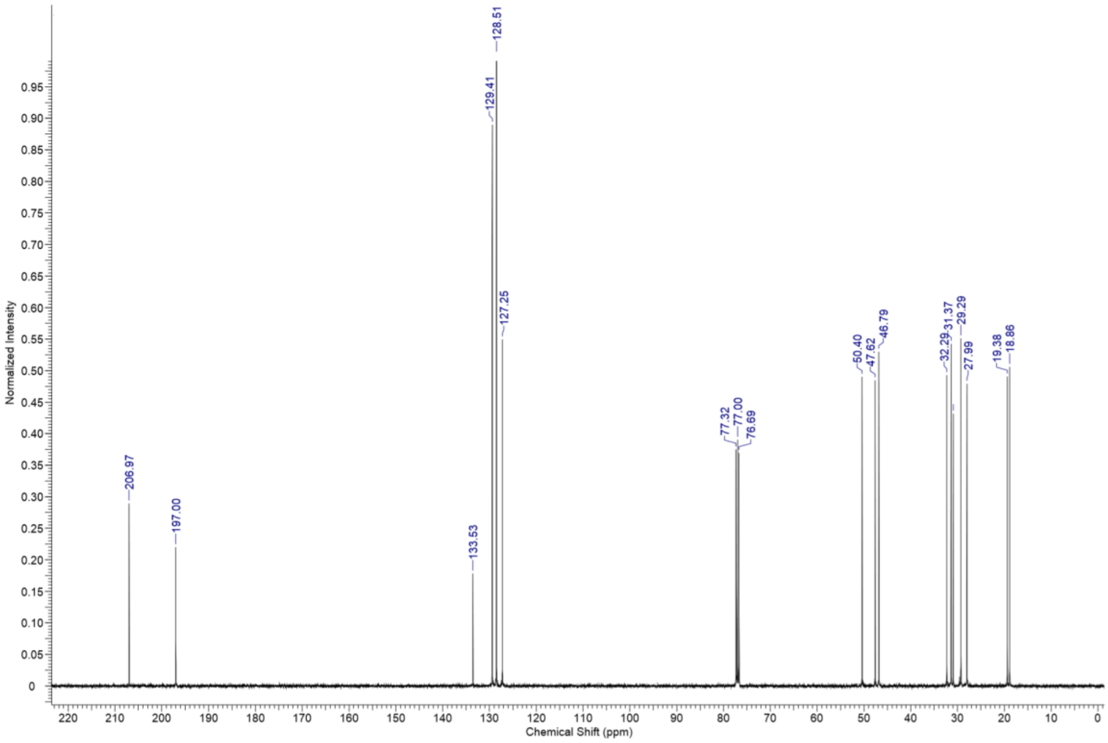

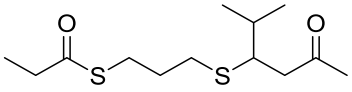

[7j] S-(3-((2-methyl-5-oxohexan-3-yl)thio)propyl) propanethioate

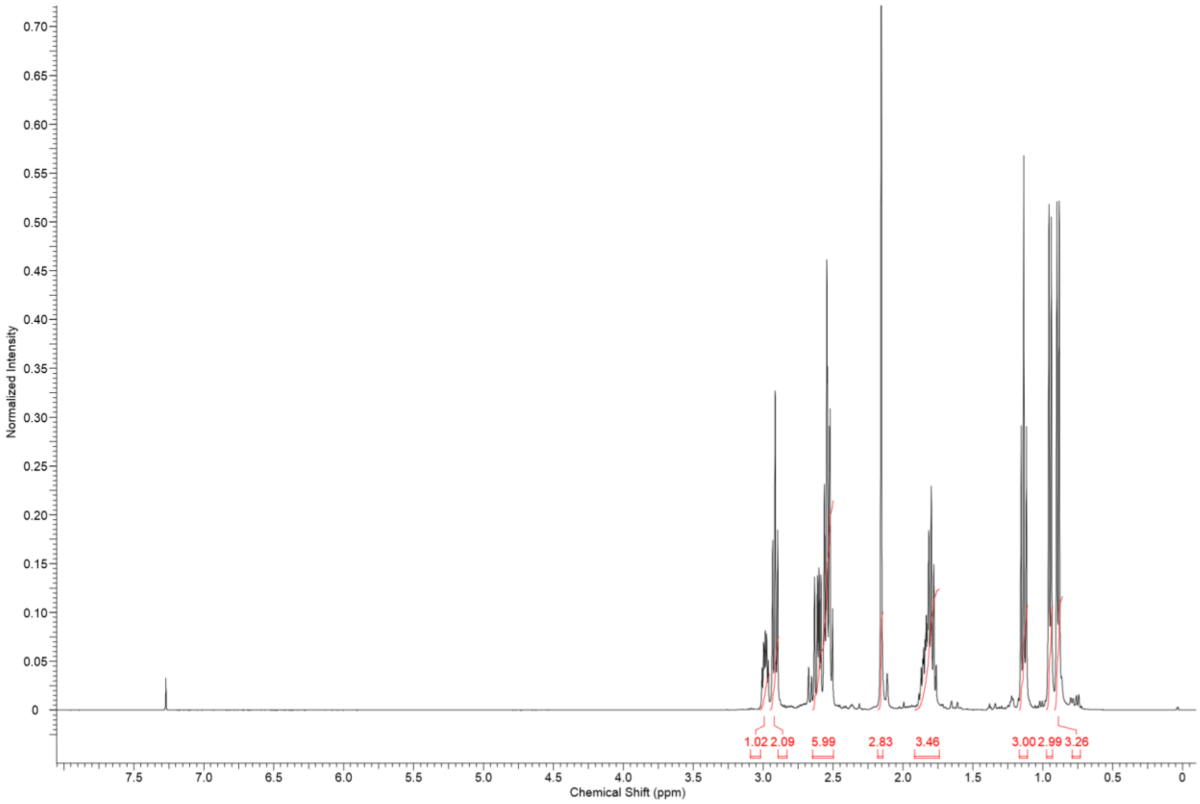

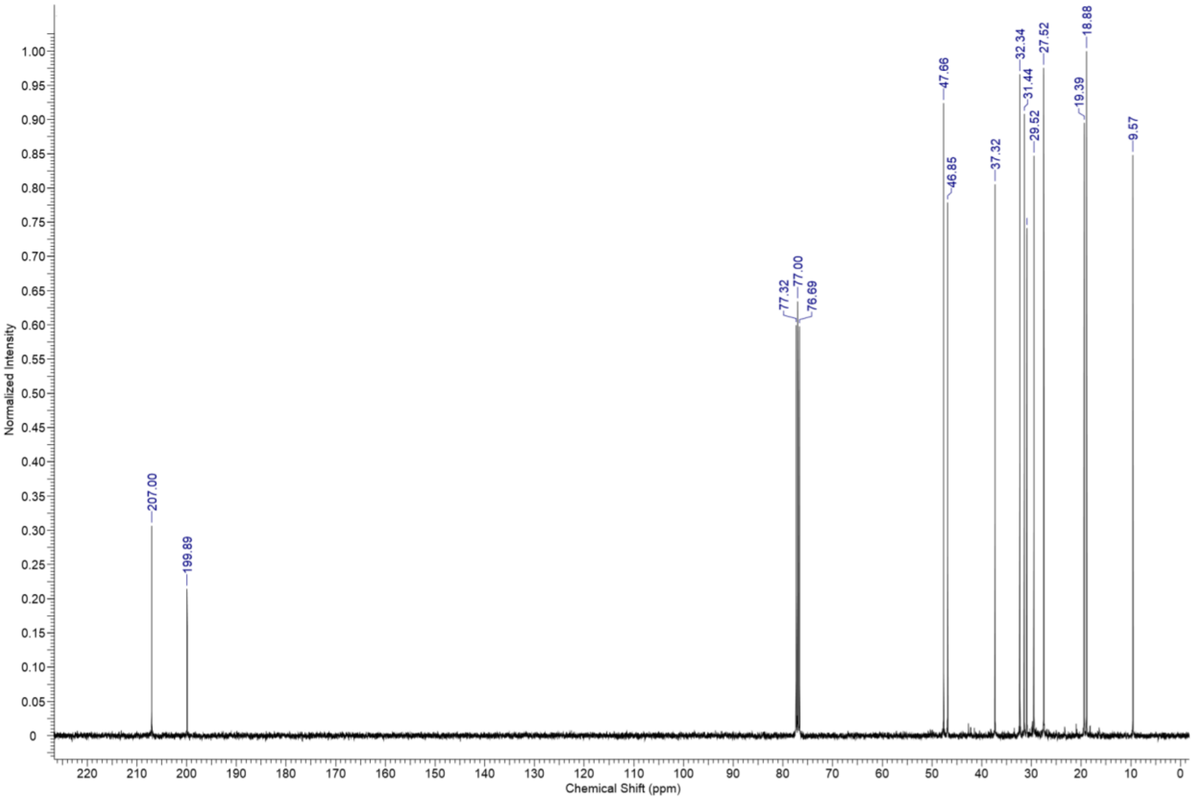

[7m] S-(3-((3-oxopentyl)thio)propyl) butanethioate

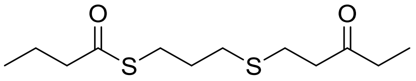

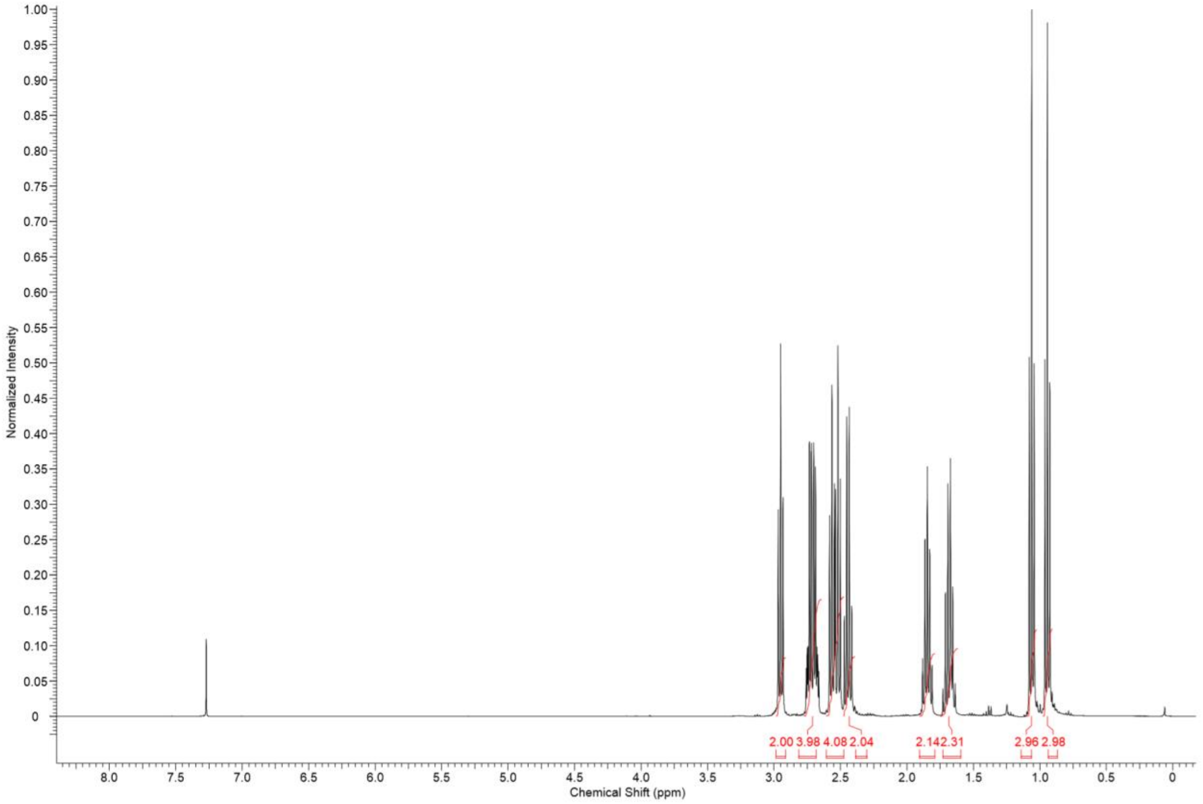

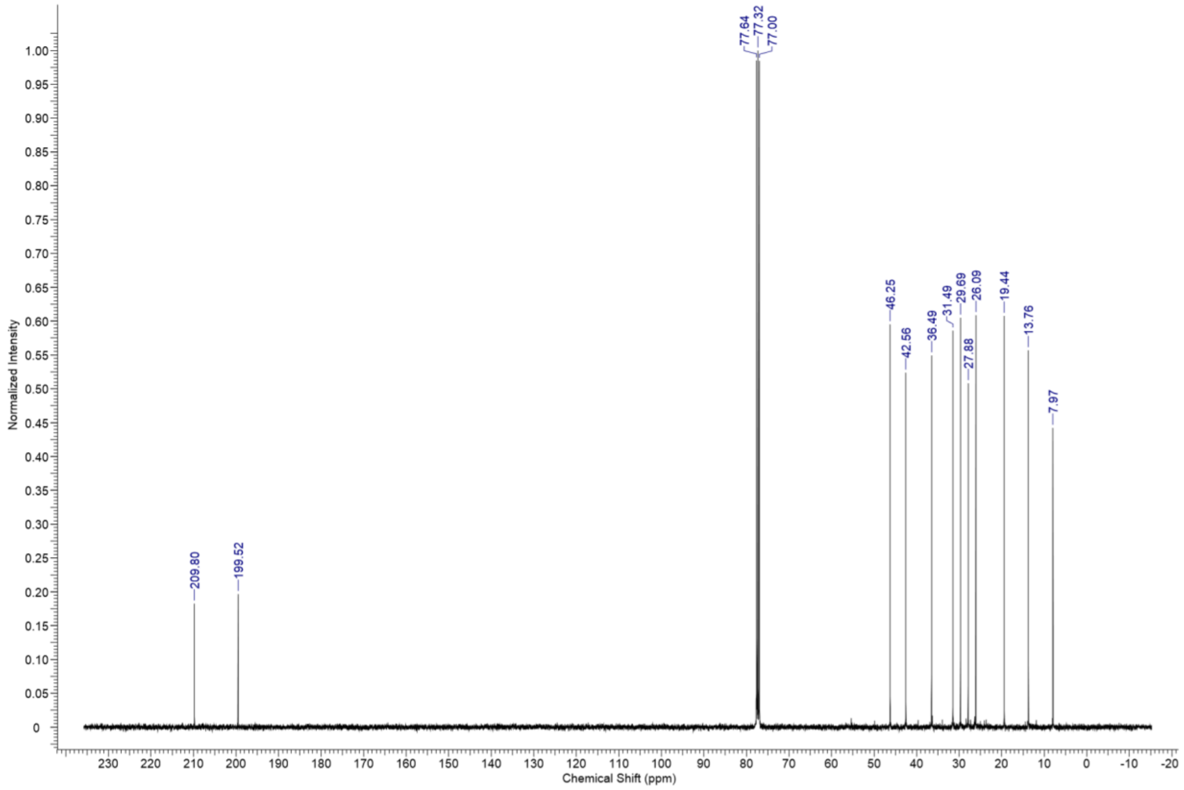

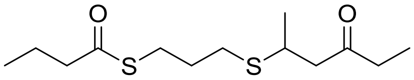

[7n] S-(3-((4-oxohexan-2-yl)thio)propyl) butanethioate

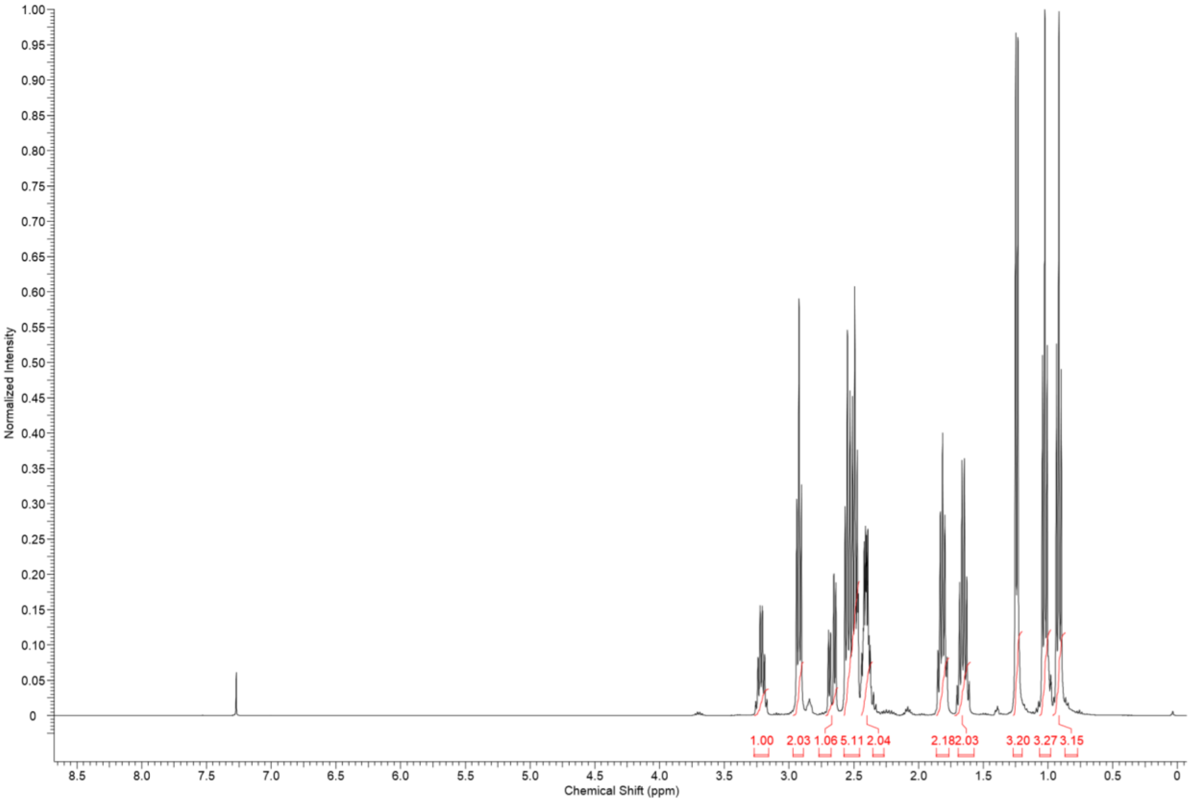

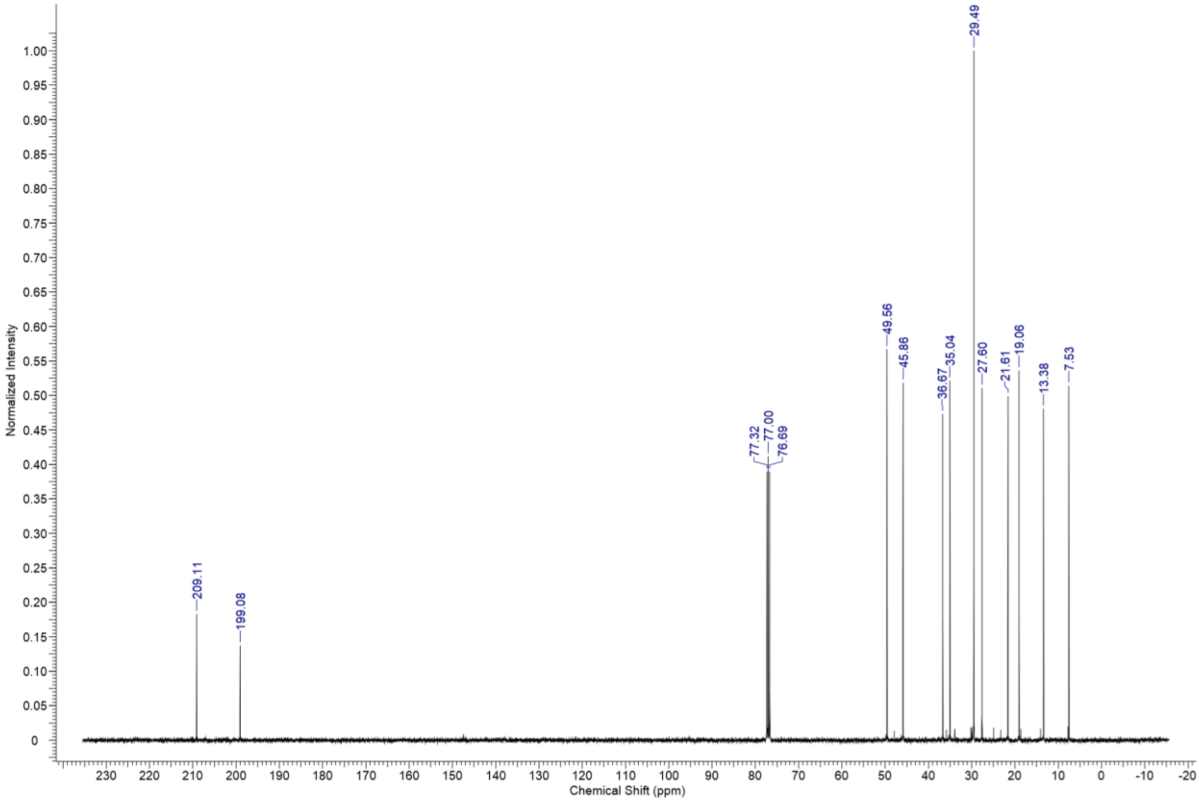

Properties and IR (Raman) spectra of compounds **1** and 2

Table S2: Properties and IR spectra of starting materials **1a-e** and **2a-e**.

No	Structure	properties	IR
**1a**	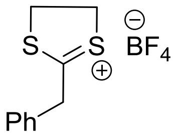	Colorless solid	IR (ATR, ṽ): 3025, 1493, 1456, 1420, 1397, 1286, 1122, 1095, 1024, 925, 809, 774, 701, 667, 632, 578, 520, 505, 445, 426 cm^{-1}.
**2a**	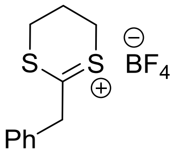	Colorless solid	IR (ATR, ṽ): 2947, 1685, 1596, 1494, 1446, 1433, 1420, 1289, 1246, 1181, 1024, 928, 909, 878, 823, 768, 708, 672, 628, 571, 520, 467 cm^{-1}.
**1b**	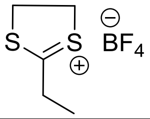	Orange oil	IR (ATR, ṽ): 2944, 1639, 1459, 1421, 1287, 1028, 945, 840, 763, 668, 519 cm^{-1}.
**2b**	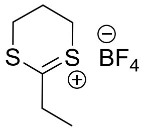	Orange oil	IR (ATR, ṽ): 2991, 1638, 1435, 1421, 1198, 1048, 977, 909, 763, 662, 578, 520 cm^{-1}.
**1c**	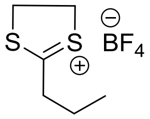	Yellow oil	IR (ATR, ṽ): 3532, 2971, 1637, 1462, 1420, 1287, 1049, 970, 764, 668, 520 cm^{-1}.
**2c**	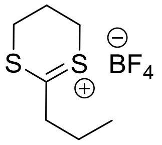	Yellow oil	IR (ATR, ṽ): 3531, 2972, 1632, 1436, 1422, 1245, 1051, 992, 909, 764, 662, 578, 520 cm^{-1}.
**1d**	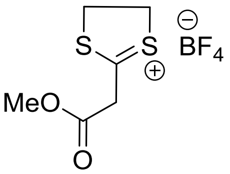	Orange oil	Raman (1064 nm, 150 mW, ṽ) =3004, 2956, 2700, 2637, 1463, 1423, 1187, 1423, 1187, 1115, 1024, 997, 888, 766, 662, 501, 478, 364, 247, 81 cm^{-1}.
**2d**	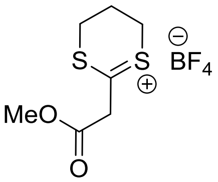	Orange solid	IR (ATR, ṽ): 2937, 1737, 1458, 1435, 1420, 1322, 1258, 1210, 1181, 1025, 980, 886, 730, 682, 661, 574, 521, 418 cm^{-1}.
**1e**	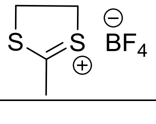	Yellow to red solid	IR (ATR, ṽ): 3021, 1421, 1290, 1153, 1121, 1088, 1025, 874, 839, 668, 520, 446 cm^{-1}.
**2e**	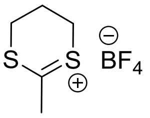	Yellow solid	IR (ATR, ṽ): 3000, 2926, 1688, 1427, 1361, 1287, 1239, 1209, 1155, 1118, 1094, 1021, 905, 880, 824, 765, 660, 566, 519, 448 cm^{-1}.

**1f**	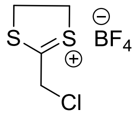	Colorless to violet solid	Raman (1064 nm, 150 mW, ṽ) =2984, 2968, 2937, 2922, 2905, 1418, 1283, 1250, 816, 742, 717, 689, 672, 534, 463, 421, 369, 331, 289, 204, 126, 84 cm^{-1}.
**1g**	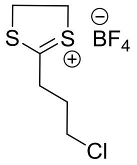	Orange oil	Raman (1064 nm, 150 mW, ṽ) = 3017, 2956, 1436, 1185, 995, 766, 661, 541, 476, 70 cm^{-1}.
